# COSMO-NET: fast and accurate machine learning surrogates for COSMO-based molecular descriptors

**DOI:** 10.1039/d6me00088f

**Published:** 2026-07-01

**Authors:** Saman Naseri Boroujeni, Amparo Galindo, George Jackson, Claire S. Adjiman

**Affiliations:** a Department of Chemical Engineering, Sargent Centre for Process Systems Engineering, Imperial College London London SW7 2AZ UK s.naseri-boroujeni@imperial.ac.uk c.adjiman@imperial.ac.uk

## Abstract

Accurate predictive thermodynamic models are essential tools for the computational design of new molecules. Models based on COnductor-like Screening Models (COSMO), such as COSMO-SAC and COSMO-RS, are well-suited for this purpose but require computationally expensive quantum-mechanical (QM) calculations to generate key properties – the surface charge density distribution (*p*(*σ*)), the surface area (*A*) and the cavity volume (*V*) – for each molecule. Here we develop graph-based neural network surrogate models, based on a directed message passing neural network (DMPNN) and a graph convolutional network (GCN), to predict these molecular properties rapidly and accurately. We train the models on a dataset of over 16 000 compounds generated with an automated QM calculation pipeline. The DMPNN model outperforms GCN for the prediction of the surface charge density, while the GCN provides a higher accuracy for surface area and cavity volume. We build on these strengths to propose a hybrid model: COSMO-NET. When applied to predict octanol–water partition coefficients and solid–liquid solubility across diverse solvents, COSMO-NET consistently outperforms individual models with the ranking: COSMO-NET > DMPNN > GCN > ECFP–MLP. COSMO-NET demonstrates remarkable robustness with minimal performance variation across different data splitting strategies. These results demonstrate that machine-learning surrogates can reliably replace costly QM calculations while maintaining accuracy, enabling the rapid discovery of new molecules and accurate evaluation of their thermodynamic properties.

## Abstract 2

COSMO-NET is a fast and accurate machine learning surrogate model that replaces computationally expensive quantum mechanics calculations for the generation of COSMO-based molecular descriptors.

## Graphical abstract

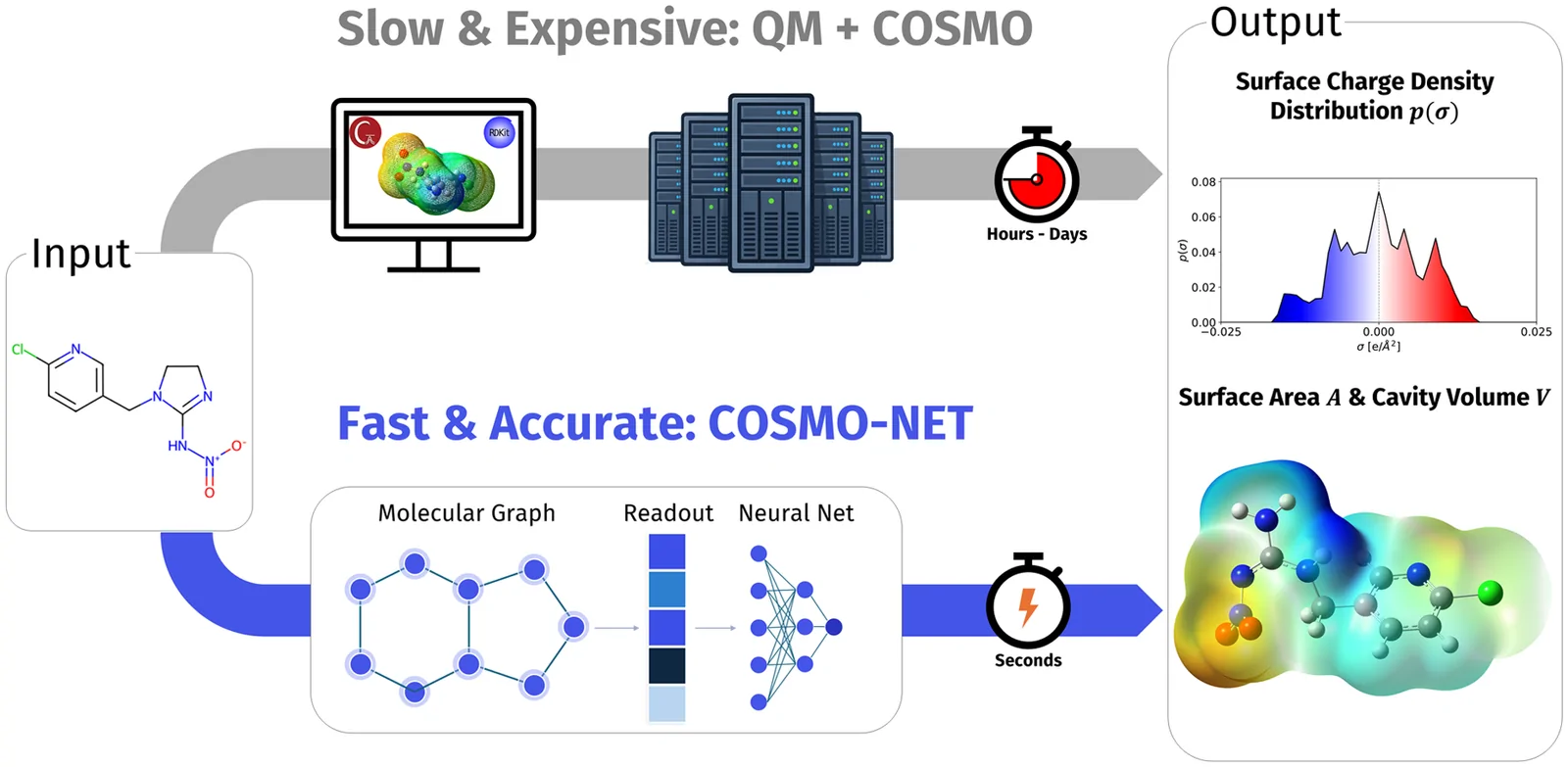

**Design, System, Application**

Predicting how molecules behave in solution is central to the rational design of new compounds across the chemical and pharmaceutical industries, yet the quantum-mechanical calculations required by widely used thermodynamic models remain a major computational bottleneck. In this work we address this challenge by replacing expensive quantum-mechanical calculations, typically requiring hours per molecule, with graph-based neural network surrogate models that learn directly from molecular connectivity encoded as SMILES strings. The hybrid model COSMO-NET combines the complementary strengths of two graph neural-network architectures: a directed message-passing network for predicting surface charge density distributions, and a graph-convolutional network for predicting molecular surface area and cavity volume. A key feature is full compatibility with both the COSMO-SAC 2002 and COSMO-SAC 2010 thermodynamic frameworks, requiring the surrogate to reproduce all descriptor types needed for activity coefficient prediction. Trained on a newly compiled database of over 16 000 compounds generated through an automated quantum-chemical pipeline, COSMO-NET achieves accuracy comparable to the underlying quantum-mechanical reference while delivering predictions in a fraction of the time. This capability directly enables high-throughput screening of large molecular libraries and integration into computer-aided molecular design workflows, with immediate relevance to solvent selection, drug formulation, and sustainable process design.

## 1. Introduction

Computational molecular design and discovery^1,2^ play a vital role in advancing product and process development in the chemical and pharmaceutical industries by providing an efficient approach to search a vast chemical space and find promising chemical structures for specific objectives. These objectives can range from physical properties to functional targets such as the performance of a device or chemical process, *e.g.* crystallization of drug compounds.^3–6^ Phase equilibrium is central to the evaluation of process performance, necessitating reliable predictive thermodynamic models. These include molecular-based approaches, group-contribution methods, and thermodynamic models derived from the conductor-like screening model (COSMO).^7^ The statistical associating fluid theory (SAFT)^8,9^ and its variants^10–12^ are examples of molecular-thermodynamic models that describe fluids in terms of intermolecular interactions and association effects. By contrast, group-contribution methods such as the universal quasi-chemical functional group activity coefficient (UNIFAC)^13^ estimate mixture behavior based on predefined functional groups rather than explicit molecular interactions. Notably, SAFT has also been reformulated as a group-contribution framework in the SAFT-γ Mie approach.^14^

Thermodynamic models based on COSMO, including COSMO-RS^15^ and COSMO-SAC,^16^ combine quantum-chemically derived surface charge densities with statistical thermodynamics to predict activity coefficients and phase equilibria. Among the available approaches, group-contribution and COSMO-based models are particularly attractive as they do not rely on extensive experimental fitting. While group-contribution methods^13,14,17–19^ often achieve high accuracy,^20,21^ their applicability is constrained by the availability of parameters for the functional groups present in the system of interest. By contrast, COSMO-based models such as COSMO-SAC^16,22,23^ and COSMO-RS,^7,15^ although typically slightly less accurate than group-contribution methods,^20,24^ offer significantly broader applicability as they do not depend on predefined group parameters.

However, these models rely on computationally intensive QM calculations to generate the required COSMO-based molecular descriptors, including the surface charge density distribution (*p*(*σ*) in COSMO-SAC 2002,^16^ and *p*^nhb^(*σ*), *p*^oh^(*σ*), and *p*^ot^(*σ*) in COSMO-SAC 2010^22^), molecular surface area (*A*), and cavity volume (*V*). This creates a significant computational bottleneck, particularly for large molecules or when exploring new compounds within an optimization framework. Surrogate models that can replace the time-consuming QM COSMO calculations and that are capable of rapidly and accurately predicting COSMO-based molecular descriptors would help to overcome this challenge and enhance the utility of COSMO-based models in molecular design and discovery.

A few attempts have been made to use machine-learning approaches to develop surrogate models for the prediction of COSMO-based molecular descriptors. Liu *et al.*^25^ used 1120 compounds, taken as a subset of VT 2005, the Virginia Tech database,^26^ to train the machine-learning-based atom contribution (MLAC) method to predict both *p*(*σ*) × *A* and *V*. Chen *et al.*^27^ developed a transformer-convolutional neural network (CNN) model to predict *p*(*σ*) × *A* and *V*, enabling high-throughput solvent screening with the COSMO-SAC 2002 thermodynamic activity coefficient model.^16^ They used the large and diverse ChEMBL database, comprising 1.87 million molecules, to train a simplified molecular input line entry system (SMILES) transformer model, and they used the Virginia Tech database^26^ (1431 compounds) to train the CNN. Zhang *et al.*^28^ employed the message-passing neural network model implemented in Chemprop^29^ and developed by Yang *et al.*^30^ to predict *p*(*σ*) × *A* and *V* using the Virginia Tech database.^26^ Abranches *et al.*^31^ developed a graph convolutional network (GCN) model to predict *p*(*σ*) × *A* from molecular structure using three types of node-level features. They also used the Virginia Tech database^26^ for the training of their model.

Although these studies have demonstrated the potential of machine-learning models to serve as surrogates for computationally expensive QM COSMO calculations, significant gaps remain. A primary limitation is the small size of the available database (1431 compounds), which constrains the models' ability to generalize, particularly for compounds with multiple functional groups. This limitation reduces model accuracy and weakens the extrapolative power of the surrogate models. Furthermore, despite achieving the highest reported coefficient of determination, the GCN model proposed by Abranches *et al.*^31^ does not predict cavity volume, which is an essential property for calculating activity coefficients in the liquid phase. As a result, it cannot be used as a surrogate model in computer-aided molecular design (CAMD) and many other applications. In addition, none of the aforementioned studies report the COSMO-based molecular descriptors required for the more recent and more accurate version of the COSMO-SAC model,^22^ in which *p*(*σ*) is split into non-hydrogen bonding (*p*^nhb^(*σ*)) and hydrogen bonding contributions and where the latter is further divided into OH (*p*^oh^(*σ*)) and OT (*p*^ot^(*σ*)) components. There is, therefore, a clear need: (i) to develop a more comprehensive database of sigma files (containing COSMO-based molecular descriptors), covering a wider range of compounds; and (ii) to train new machine-learning models applicable to both COSMO-SAC 2002^16^ and COSMO-SAC 2010^22^ on this enriched dataset to improve predictive performance and utility.

Consequently, in our current work, we develop a computational pipeline to perform QM COSMO calculations and use it to obtain the sigma files of more than 16 000 compounds, containing atom types H, C, N, O, F, P, S, Cl, and Br. Using this database, two widely used types of chemistry-aware GNN architectures, namely the directed message passing neural network (DMPNN)^30^ and the graph convolutional network (GCN),^32^ are used to train five models that generate a sigma file from a SMILES string. These chemistry-aware architectures are selected over alternative approaches such as transformer-based models and equivariant neural networks (*e.g.*, SchNet^33^ and PaiNN^34^) for three key reasons: (i) computational efficiency which is critical for high-throughput molecular design applications where inference speed is essential; (ii) data efficiency – our dataset of 16 130 compounds is modest by transformer standards, and GNNs generalize better in data-limited regimes due to chemistry-aligned inductive biases; and (iii) task suitability, since COSMO descriptors are normalized probability distributions decoupled from 3D molecular geometry, the additional representational capacity of equivariant networks provides no advantage while incurring higher computational cost. To provide a frame of reference, we evaluate all models against a baseline approach using Morgan fingerprints^35^ as molecular descriptors coupled with a multilayer perceptron (MLP) for prediction.

The remainder of the article is structured as follows: in section 2, the data generation pipeline and the GNN architectures are briefly explained. In section 3, the developed database of COSMO-based molecular descriptors is summarized, and our model development (data splitting and model training) is described. The accuracy of the DMPNN and GCN models is also evaluated leading to the identification of a hybrid model, COSMO-NET, as a high-performance alternative. The impact of using ML-based surrogate models on the prediction of two key thermodynamic properties, the octanol–water partition coefficient and the solid–liquid solubility, is assessed. Finally, in section 4, our findings are summarized and conclusions are drawn.

## 2. Methods

In our current work, a database of sigma files of compounds is first generated by conducting QM COSMO calculations. Graph-based surrogate models are then trained and used to estimate the COSMO-based molecular descriptors of the molecules. In this section, the pipeline for performing COSMO calculations (the Data generation step) is first presented. Then, the machine-learning models are briefly explained.

### 2.1. Data generation

The collection or generation of a “reasonable” number of data points is a necessary step in the development of surrogate models. Although having an ample number of data points does not guarantee success in the development of surrogate models, lack of data assures failure or at least limits the usefulness of the models. Since our objective is to develop surrogate models that replace computationally expensive QM COSMO calculations, data generation requires performing QM calculations. In order to facilitate this step, we have developed an automated pipeline for which the canonical SMILES string of a molecule is inputted and the corresponding COSMO-based molecular descriptors are outputted.

In Fig. 1, we present the pipeline used for the calculations of the COSMO files and the calculated COSMO-based molecular descriptors (*p*(*σ*), *A*, *V*). The pipeline involves three stages: (i) molecular structure parsing and conformer generation from canonical SMILES, followed by conformer clustering and Gaussian input generation; (ii) QM geometry optimization and COSMO calculations; and (iii) post-processing of COSMO outputs to generate COSMO-based molecular descriptors.

**Fig. 1:**
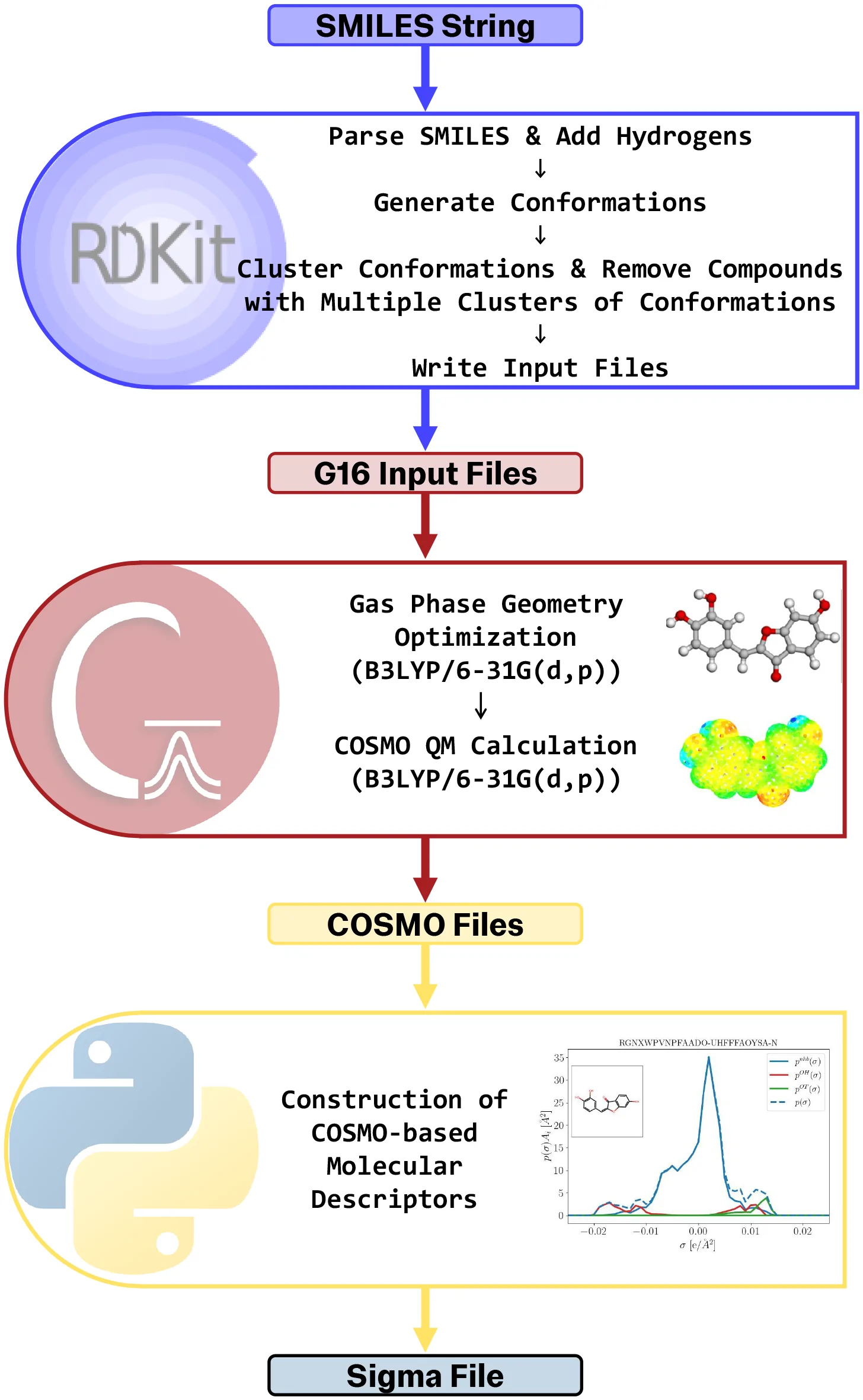
The pipeline for generating COSMO and sigma files from a SMILES string.

In the first stage, the canonical SMILES string is inputted and parsed using RDKit.^36^ After adding hydrogen atoms to the molecule, the rdkit.Chem.AllChem module is used to generate molecular conformations. These molecular conformations are then clustered using the clustering algorithm developed by Butina^37^ (rdkit.ML.Cluster.Butina module). Finally, input files for the Gaussian 16 (ref. 38) for both geometry optimization and single-point energy calculations are generated.

In the second stage, a geometry optimization in the gas phase using the B3LYP functional^39^ and the 6-31G(d,p) basis set is performed using Gaussian 16. The optimized geometry is then used to perform COSMO calculations at the same level of theory (LoT). The resulting COSMO file includes the surface charge density and surface area of the segments on the molecule.

In the third and final stage, using the open-source code COSMOSAC, developed by Bell *et al.*^40^ and the parameters^41^ summarized in Tables S1 and S2, the surface charge density of the segments on the molecule (in the COSMO file) is normalized and the corresponding sigma file is obtained. The resulting sigma file for COSMO-SAC 2002 (ref. 16) includes *p*(*σ*) ∈ ℝ^51^, *A*, and *V* and for COSMO-SAC 2010 (ref. 22), it includes *p*^nhb^(*σ*) ∈ ℝ^51^, *p*^oh^(*σ*) ∈ ℝ^51^, *p*^ot^(*σ*) ∈ ℝ^51^, *A*, and *V*. The computational pipeline is accessible in the Zenodo and GitHub repositories.

### 2.2. Machine learning models

Two graph-based neural network models are trained and used to predict COSMO-based molecular descriptors (*A*, *V*, *p*(*σ*), *p*^nhb^(*σ*), *p*^ot^(*σ*), or *p*^oh^(*σ*)). In Fig. 2, we present the overall end-to-end (SMILES string to target) architecture of the graph-based machine-learning models (graph convolutional network and directed message passing neural network). The model architectures used in our current work are the same as those presented by Yang *et al.*^30^ and Duvenaud *et al.*^32^ and implemented in the Deepchem^42^ Python library with a slight modification for the molecular featurization. Additionally, extended-connectivity fingerprints^35^ are used as the molecular encoding, combined with a multilayer perceptron baseline model (termed ECFP–MLP, abbreviated as ECFP in the figures).

**Fig. 2:**
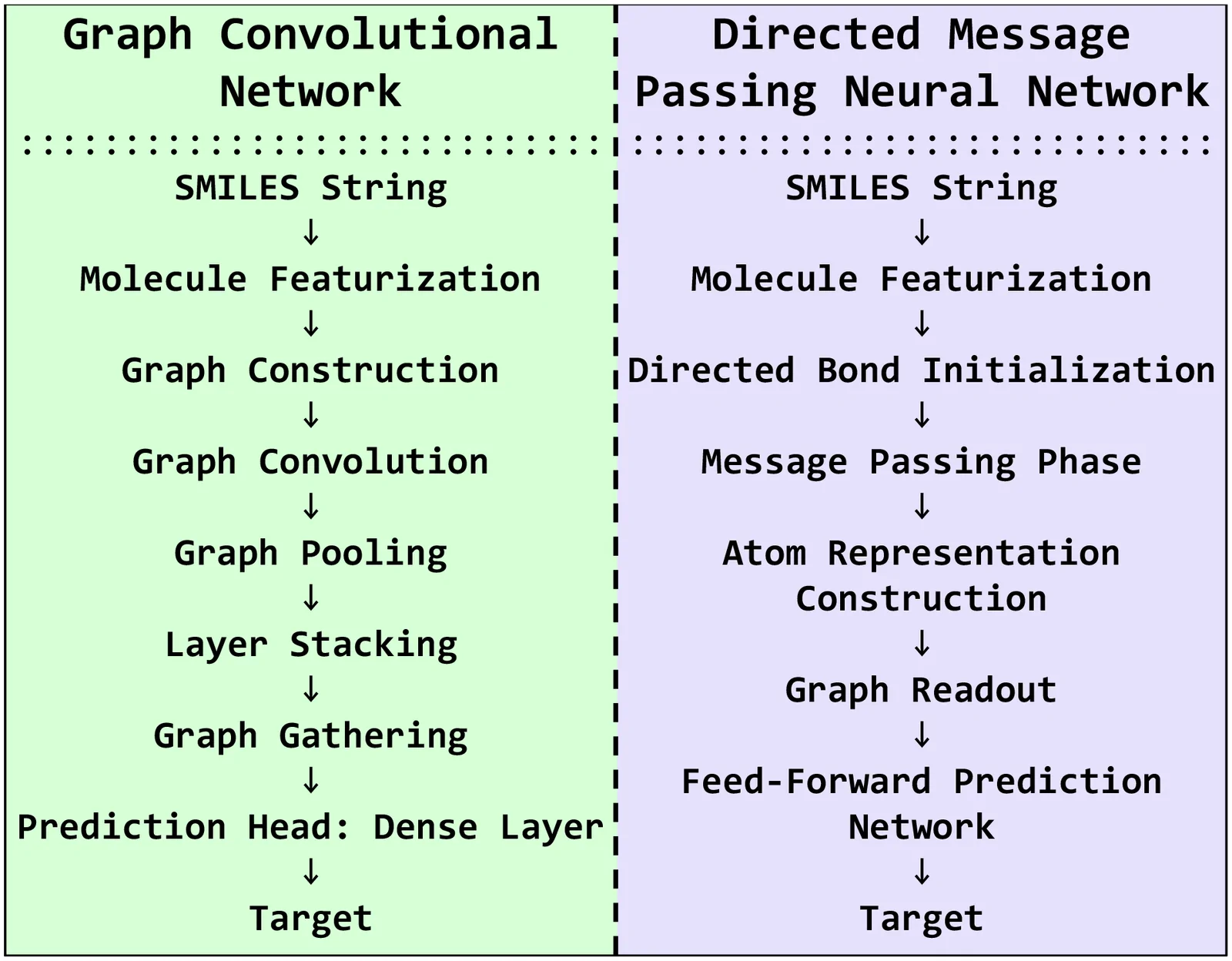
The architecture of the directed message passing neural network^30^ and graph convolutional network^32^ models used in the current work. Target could be [*A*, *V*] ∈ ℝ^2^, *p*(*σ*) ∈ ℝ^51^, *p*^nhb^(*σ*) ∈ ℝ^51^, *p*^ot^(*σ*) ∈ ℝ^51^, or *p*^oh^(*σ*) ∈ ℝ^51^.

#### 2.2.1. Graph convolutional network (GCN)

GCN is a class of neural networks that is operated directly on graph-structured data. Here, we adopt the neural fingerprint model introduced by Duvenaud *et al.*,^32^ which generalizes traditional circular molecular fingerprints into a differentiable, trainable architecture. The model is tailored for molecular property prediction tasks, where molecules are represented as graphs with atoms as nodes and chemical bonds as edges.

##### 2.2.1.1. Molecular featurization

We use a modified version of the ConvMolFeaturizer from the DeepChem library to convert molecular graphs into structured graph representations suitable for graph convolutional networks. The output of the featurization process is a node feature matrix **X** ∈ ℝ^*N*×*F*^, where *N* is the number of atoms and *F* is the feature dimension. The atom feature vector in the node feature matrix is summarized in Table 1. In addition, the output includes an adjacency list structure *𝒩*, representing the bonding topology of the molecular graph. The indices of neighbors of atom *v* are stored in list *𝒩*(*v*).

**Table 1:** Breakdown of the 35-dimensional atom feature vector used in the GCN and DMPNN models

Feature category	Encoding type	Size
Element type	One-hot: C, N, O, S, F, P, Cl, Br	8
Degree	One-hot: 0–5	6
Formal charge	One-hot: −2, −1, 0, 1, 2	5
Hybridization	One-hot: sp, sp2, sp3, sp3d, sp3d2	5
Aromaticity	Boolean	1
Total hydrogens	One-hot: 0–4	5
Chirality	One-hot: 0–3	4
Atomic mass	Atomic mass × 0.01	1
**Total**		**35**

##### 2.2.1.2. Graph construction

Let a molecular graph be denoted as 𝔾 = (𝕍, 𝔼), where 𝕍 is the set of atoms (nodes) and 𝔼 ⊆ 𝕍 × 𝕍 is the set of bonds (edges). Each atom *v* ∈ 𝕍 is associated with an initial feature vector **h**^(0)^_*v*_ ∈ ℝ^*F*^ that encodes atom-level properties as described in Table 1.

##### 2.2.1.3. Graph convolution

Each layer of the GCN performs a message-passing operation followed by an update step. For a given layer ℓ and atom *v* the information pertaining to all neighbors of *v*, 𝒩(*v*), is aggregated *via* summation as a vector **m**^(ℓ)^_*v*_:1
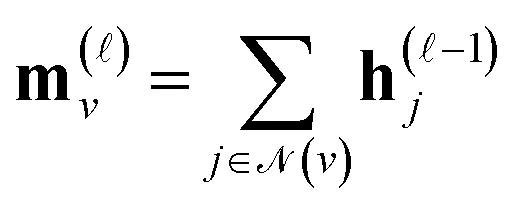


The aggregated message is then combined with the atom's current representation using a degree-specific affine transformation to form vectors **z**^(ℓ)^_*v*_ and **h**^(ℓ)^_*v*_:

2 **z**^(ℓ)^_*v*_ = **m**^(ℓ)^_*v*_**W**^(ℓ)^_*d*(*v*)_ + **h**^(ℓ−1)^_*v*_**W**^(ℓ)^_self,*d*(*v*)_ + **b**^(ℓ)^_*d*(*v*)_,

3 **h**^(ℓ)^_*v*_ = ReLU(**z**^(ℓ)^_*v*_),

where ReLU(·), *d*(*v*), **W**^(ℓ)^_*d*(*v*)_ and **W**^(ℓ)^_self, *d*(*v*)_, and **b**^(ℓ)^_*d*(*v*)_ are the rectified linear unit activation function, the degree of atom *v* (numbers of atoms bonded to atom *v*), learnable weight matrix for messages (which depends on the degree), learnable weight matrix for the self-term, and the bias, respectively.

##### 2.2.1.4. Graph pooling

After each convolution, a local pooling operation is applied to refine each node's representation. The pooling layer performs element-wise max pooling over each node's neighborhood, including itself:

4 **h̃**^(ℓ)^_*v*_ = max({**h**^(ℓ)^_*v*_}∪{**h**^(ℓ)^_*j*_:*j* ∈ *𝒩*(*v*)}).

The pooled representation **h̃**^(ℓ)^_*v*_ is then used as the input to the next layer.

##### 2.2.1.5. Graph gathering

Once *L* layers of message passing and pooling have been applied, the atom-level features are aggregated into a molecule-level embedding using global sum and max pooling:5
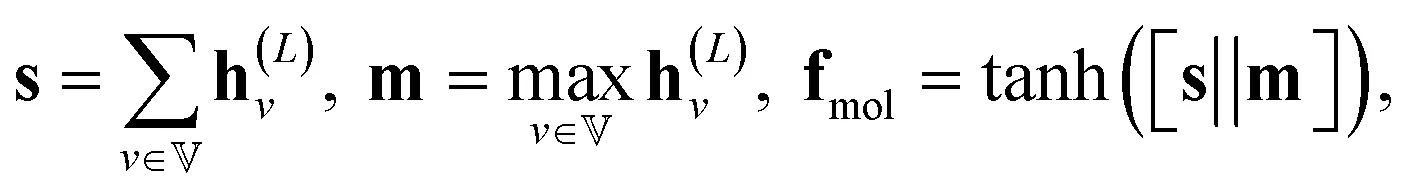
where **f**_mol_ ∈ ℝ^2*F*^ is the final fingerprint vector of the molecule and [·∥·] denotes vector concatenation.

##### 2.2.1.6. Prediction and loss

For regression tasks, a softplus activation function is used:

6 **ŷ** = softplus(**f**_mol_**W**_reg_ + **b**_reg_),

where **W**_reg_ and **b**_reg_ are a learnable weight matrix for the regression layer and a learnable bias vector, respectively. The model is trained end-to-end using the mean squared error (MSE) loss function ℒ_reg_ for regression:7
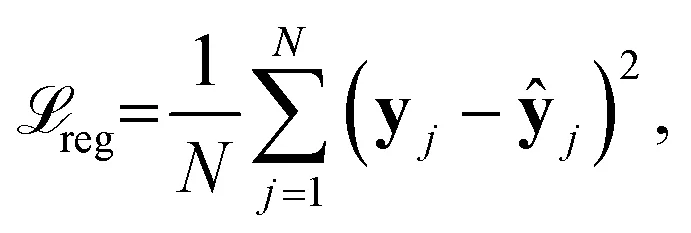
where **y**_*i*_, **ŷ**_*i*_ and *N* are the actual data points, the predicted data, and the number of data used for training.

#### 2.2.2. Directed message passing neural network (DMPNN)

DMPNN^30^ is a molecular graph neural network (GNN) that operates on directed bonds rather than atoms, improving representation learning by preventing unnecessary loops (totters) in the message passing trajectory. Similar to a GCN, a molecule is represented by a graph 𝔾 = (𝕍, 𝔼), where 𝕍 is the set of atoms (nodes) and 𝔼 is the set of directed bonds (edges). Each atom *v* ∈ 𝕍 has a feature vector **x**_*v*_ ∈ ℝ^*d*_A_^, and a set of directed bonds. A directed bond that includes atoms *v* and *w*, {*v*,*w*} ∈ 𝔼, has a feature vector **e**_*vw*_ ∈ ℝ^*d*_A_^. *d*_A_ and *d*_B_ are the dimensions of the atom and bond features, respectively.

##### 2.2.2.1. Molecular featurization

The SMILES string of each molecule is used as input and transformed into a molecular graph using RDKit, with atoms corresponding to nodes and bonds to edges.

The initial features vector **x**_*v*_ for atom *v* ∈ 𝕍 is obtained from a one-hot encoding of the element type, number of bonds linked to the atom, formal charge, chirality (if encoded in the SMILES), number of hydrogens, hybridization, and aromaticity of the atom, as well as the atomic mass (divided by 100 for scaling). This is summarized in Table 1.

The initial features vector **e**_*vw*_ for edge {*v*,*w*} ∈ 𝔼 comprises the bond type, Boolean variables describing whether the bond is conjugated or in a ring, and a one-hot encoding of whether it contains stereochemical information, such as a *cis*/*trans* double bond. The bond feature vector used for the DMPNN model is presented in Table 2.

**Table 2:** Breakdown of the 12-dimensional bond feature vector used in the DMPNN model

Feature category	Encoding type	Size
Bond type	One-hot: Single, double, triple, or aromatic	4
Conjugated	Boolean	1
In ring	Boolean	1
Stereo	One-hot: none, any, E/Z or *cis*/*trans*	6
**Total**		**12**

##### 2.2.2.2. Directed bond initialization

Each directed bond is initialized with a hidden state:

8 **h**^0^_*vw*_ = ReLU(**W**_Init_[**x**_*v*_||**e**_*vw*_]),

where **W**_Init_ ∈ ℝ^*d*_h_×(*d*_A_+*d*_B_)^ is a learnable weight matrix, and *d*_h_ is the dimension of the directed bond hidden state.

##### 2.2.2.3. Message-passing phase

Over *L* steps, where *L* is a hyperparameter, messages are passed along directed edges. For each directed bond {*v*,*w*}, messages from all other incoming edges to *v* are aggregated (excluding the reverse edge {*w*,*v*}):9
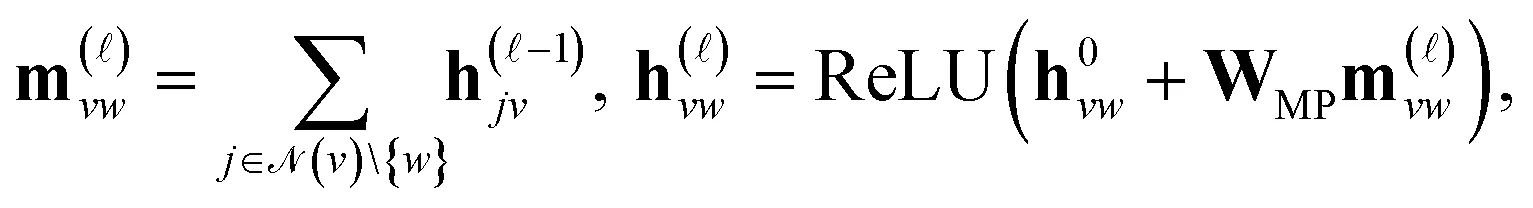
where **W**_MP_ ∈ ℝ^*d*_h_×*d*_h_^ is a learnable weight matrix, and ℓ = {1, 2, …, *L*} represents the message-passing step.

##### 2.2.2.4. Atom representation construction

After *L* steps, atom-level embeddings are derived by summing the incoming directed bond representations:10
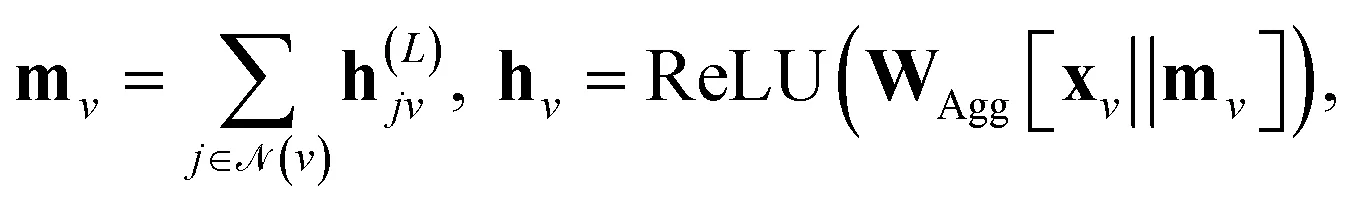
where **W**_Agg_ ∈ ℝ^*d*_h_×(*d*_A_+*d*_h_)^ is a learnable weight matrix.

##### 2.2.2.5. Graph readout

A graph-level embedding is constructed by summing over atom representations:11
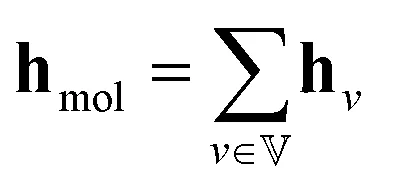


##### 2.2.2.6. Feed-forward prediction network

The final prediction is produced by a feed-forward neural network (FFN):

12 **ŷ** = FFN(**h**_mol_),

where FFN consists of one or more fully connected layers with dropout and nonlinearity.

##### 2.2.2.7. Training

The model is trained end-to-end using the MSE loss function (eqn (7)).

### 2.3. Error functions

To evaluate the performance of the models, MAE, MSE, the absolute average deviation in percent (AAD%), and the coefficient of determination (*R*^2^) are used:13
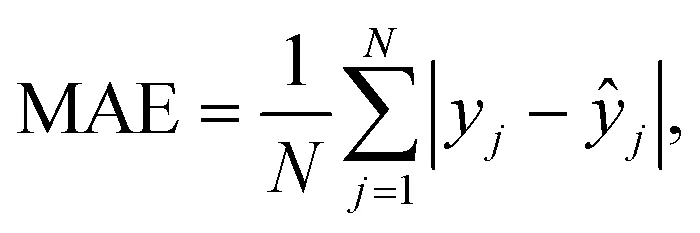
14
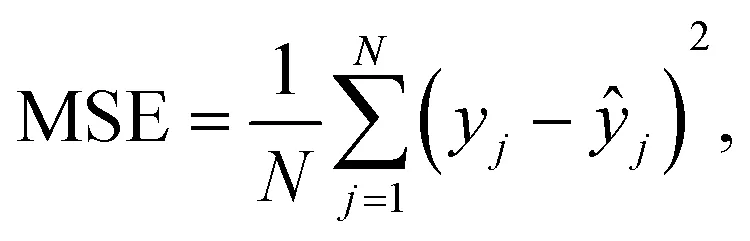
15
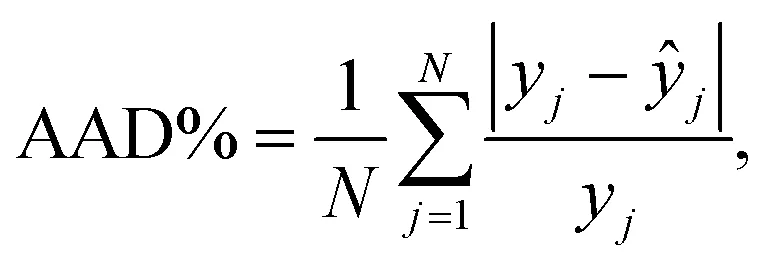
16
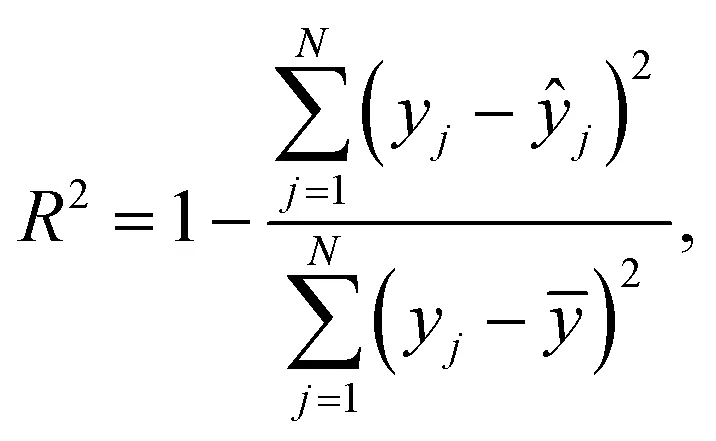
where *N* = *N*_set_*D*_target_ is the number of data points in the set (*N*_set_) multiplied by the dimension of the target (*D*_target_). Here, *ŷ*_*j*_ denotes the *j*-th element of the flattened target vector predicted by the ML models, while *y*_*j*_ denotes the corresponding *j*-th element of the flattened target vector obtained from the data generation pipeline. The flattened target vector is constructed by stacking all target vectors into a single vector of length *N*, and *ȳ* represents the mean of this vector.

## 3. Results and discussion

### 3.1. Database of COSMO and sigma files

All molecules in the DIPPR database and the Drug and Medication Information subcategory of the PubChem database with a molecular weight of less than 500 g mol^−1^ (49 959 molecules) are inputted to the pipeline introduced in section 2.1. Molecules with multiple 3D conformations which could not be clustered to one conformation with the clustering algorithm (*cf.* section 2.1) are eliminated from the SMILES list inputted to the pipeline. This reduces the dataset to 16 130 molecules. QM COSMO calculations are performed for all of these and the corresponding sigma files are generated. The full database of molecules including their CID, IUPAC names, SMILES, InChikeys and InChi as well as the COSMO and sigma files, is accessible in the Zenodo and the GitHub repositories associated with this work.

In Fig. 3, we present the histogram of molecular weights, complexity indices (a heuristic value that attempts to quantify the structural complexity of a molecule calculated with the Cactvs 3.4.8.18 software in the PubChem database), surface areas and cavity volumes of the molecules used in the data generation step. The mean, median, and standard deviation of the molecular weight of the compounds are 232.2, 213, and 98.2 g mol^−1^, respectively and the mean, median and standard deviation of their complexity score are 288, 226, 210, respectively. Most of the molecules have a complexity score of less than 500. The surface areas and volumes of the molecules calculated in the QM COSMO calculations, as shown in Fig. 3c and d, are between 0 to 700 Å^2^ (for surface area) and 700 Å^3^ (for volume). All distributions are skewed toward smaller molecules.

**Fig. 3:**
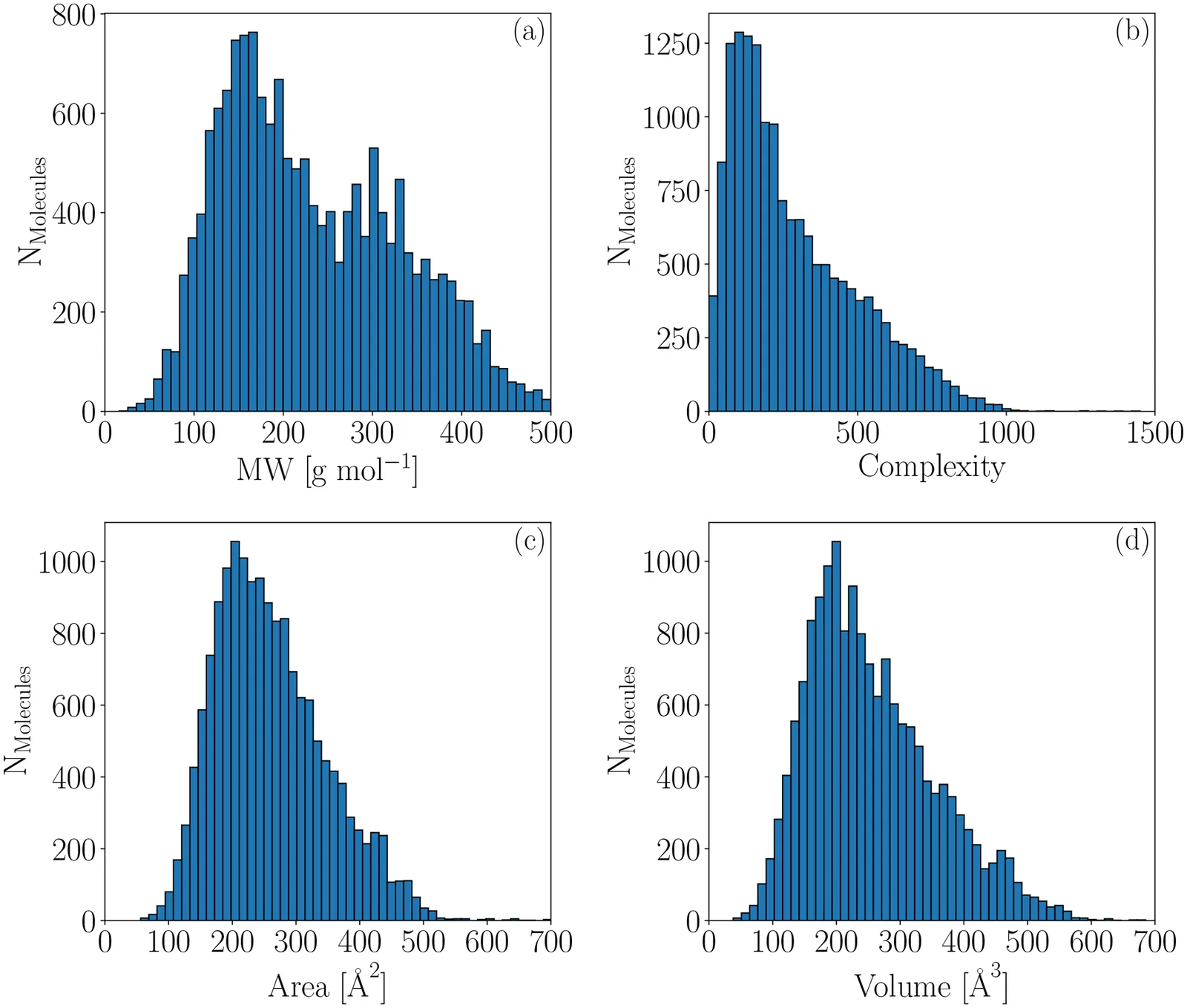
Histograms of (a) molecular weights, (b) complexity indices, (c) surface areas, and (d) cavity volumes for the compounds in the generated database.

The compounds used in the data generation step are composed of eight types of heavy atoms including carbon, oxygen, nitrogen, fluorine, phosphorous, sulfur, chlorine, and bromine, and of course include hydrogen. The molecule with the largest number of heavy atoms is 16,17-dihydroxyviolanthrone which includes 38 heavy atoms. The molecule with the smallest number of heavy atoms is methane.

In Fig. 4, we present a compact summary on the diversity of the compounds in terms of the prevalence of each atom pair. The number of molecules that contain a given pair of atoms is presented. The diagonal elements indicate the number of molecules that contain a given atom type. For instance, all molecules in the database contain at least one carbon atom, and 13 057 molecules contain at least one oxygen atom.

**Fig. 4:**
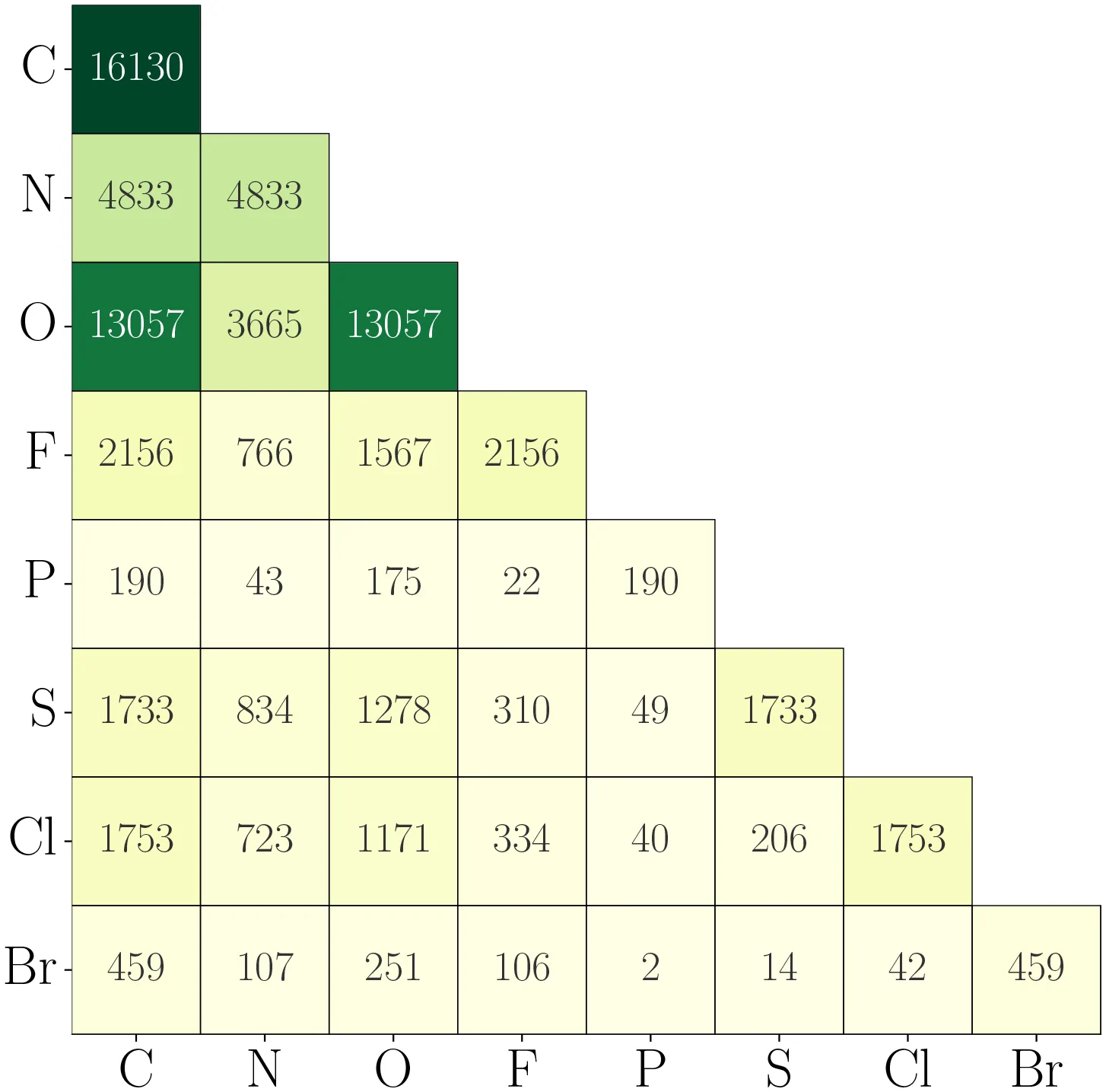
The number of molecules in the database that contain both atoms in the *x*- and *y*-axis.

As consistent with the nature of organic molecules, carbon-containing, oxygen-containing and nitrogen-containing compounds are dominant (as evident from Fig. 4). The numbers of molecules that contain other types of heavy atoms are also significant. For instance, the number of compounds that contain at least one fluorine atom (2156) in the current database is larger than the total number of molecules in the VT 2005 database that was previously used for the development of ML-based surrogate models of COSMO-based molecular descriptors.

### 3.2. Data splitting and training

#### 3.2.1. Data splitting

To comprehensively evaluate model generalization and robustness, we employ four complementary data-splitting strategies, each designed to test different aspects of model extrapolation capability. In the primary split, we use stratified sampling based on the complexity score of molecules using 10 bins. This approach ensures a representative distribution across chemical space while maintaining similar property distributions across sets (Fig. S1). It should, however, be noted that this method does not explicitly account for chemical similarity and may inadvertently place chemically similar molecules in both training and test sets.

To assess model extrapolation rigorously beyond the training domain, we additionally evaluate models using three alternative splitting strategies. First, the Fingerprint Splitter maximizes chemical dissimilarity between training and test sets by computing pairwise ECFP4 Tanimoto similarities, ensuring test molecules are chemically distant from training molecules, representing the most stringent extrapolation scenario. Second, the MaxMin Splitter selects test and validation sets as the most chemically diverse compounds using the MaxMin diversity algorithm,^43^ thereby ensuring the model encounters underrepresented regions of chemical space. Third, the Scaffold Splitter groups molecules by their Bemis-Murcko^44^ scaffold representation and splits the data such that different structural frameworks appear in training *versus* test sets, evaluating performance on structurally novel scaffolds. A uniform manifold approximation and projection (UMAP) visualization comparing the distribution of molecules across these four splitting strategies is presented in Fig. S3 in the SI, providing visual confirmation of the chemical and structural separation achieved. The consistency of the performance of the model across all splitting strategies, particularly the stringent Fingerprint and Scaffold splitters, provides strong evidence of robust generalization to chemically distinct and structurally novel compounds.

#### 3.2.2. Data normalization

Prior to training, the data are normalized using the following equation:17
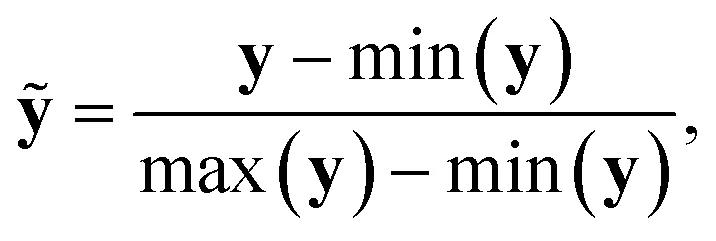
where **ỹ**, **y**, min(**y**), and max(**y**) denote the normalized value, the original value, the minimum, and the maximum of the training set, respectively. Per-task normalization is applied independently to each target property: surface area (*A*), cavity volume (*V*), and the probability distributions of *σ* (*p*(*σ*), and, where relevant, *p*^nhb^(*σ*), *p*^oh^(*σ*), and *p*^ot^(*σ*)).

#### 3.2.3. Training

To prevent overfitting on the training dataset, we adopt a unified hyperparameter strategy where all models employ fixed, architecture-specific hyperparameters across all prediction tasks. We use consistent model configurations: ECFP–MLP with 128-dimensional fingerprints and two layers of 51 neurons; GCN with two convolutional layers of 51 units and a single dense layer of 51 neurons; and DMPNN with three message-passing steps, 51-dimensional hidden states, and one FFN layer. All models employ dropout (0.1) for regularization. A batch size of 64 and 100 epochs are used for 5-fold cross-validation training.

In Fig. 5 and 6, we present parity plots comparing test set predictions of *p*(*σ*), *A*, and *V* from the GCN, DMPNN, and ECFP–MLP models against QM COSMO reference values across four train-test splitting strategies (Fingerprint, MaxMin, Scaffold, and Stratified). Parity plots for *p*^nhb^(*σ*), *p*^oh^(*σ*), and *p*^ot^(*σ*) are provided in the SI. Across all split methods, both the GCN and DMPNN models demonstrate superior predictive accuracy compared to the ECFP–MLP baseline model.

**Fig. 5:**
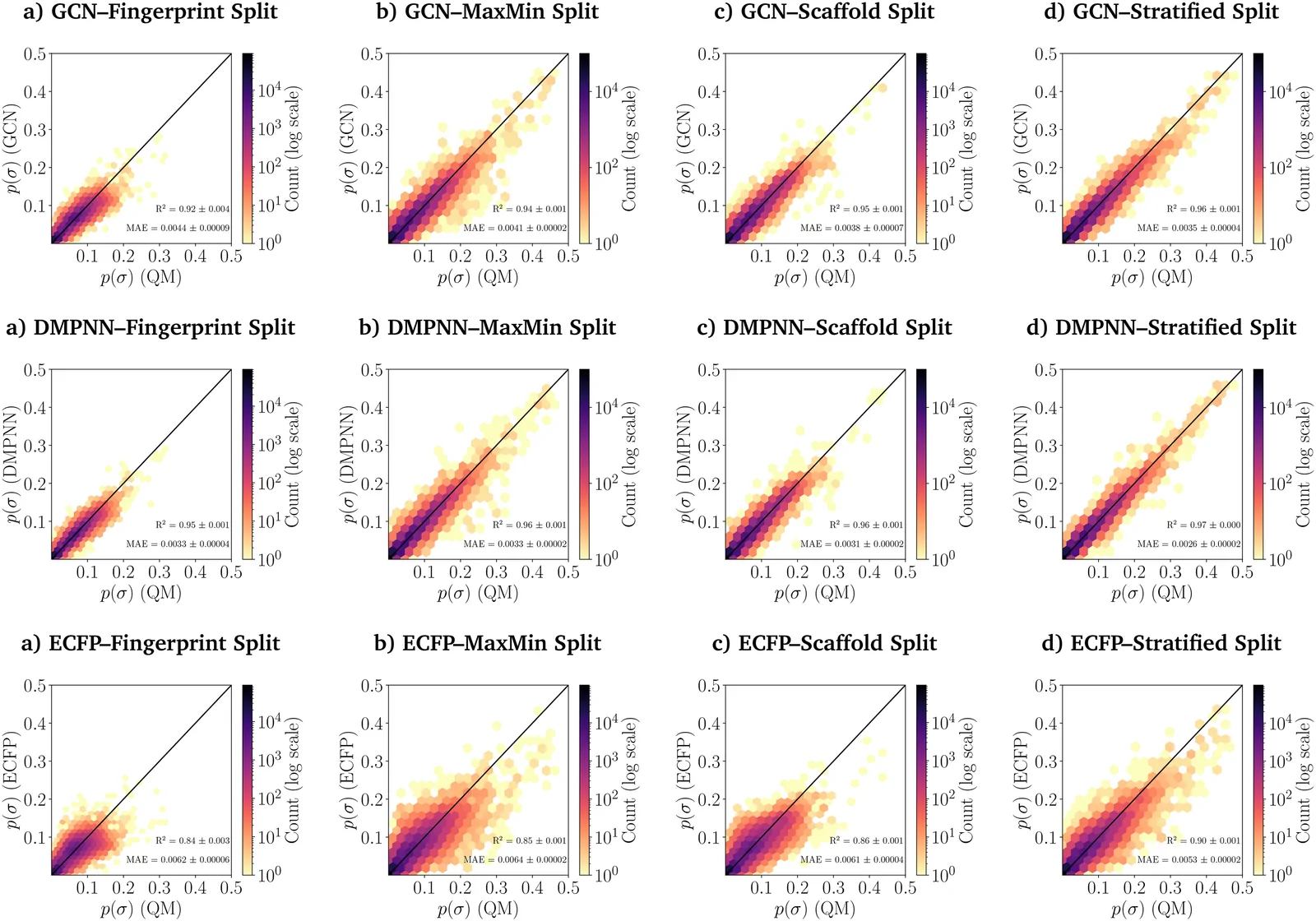
Parity plots for predictions of *p*(*σ*) across three machine learning models (GCN, DMPNN, ECFP–MLP) and four train-test splitting strategies (Fingerprint, MaxMin, Scaffold, and Stratified). Each subplot compares model predictions to QM COSMO reference values for the test set. Hexagonal bins indicate the density of predictions, with color intensity representing the number of data points (see color bar).

**Fig. 6:**
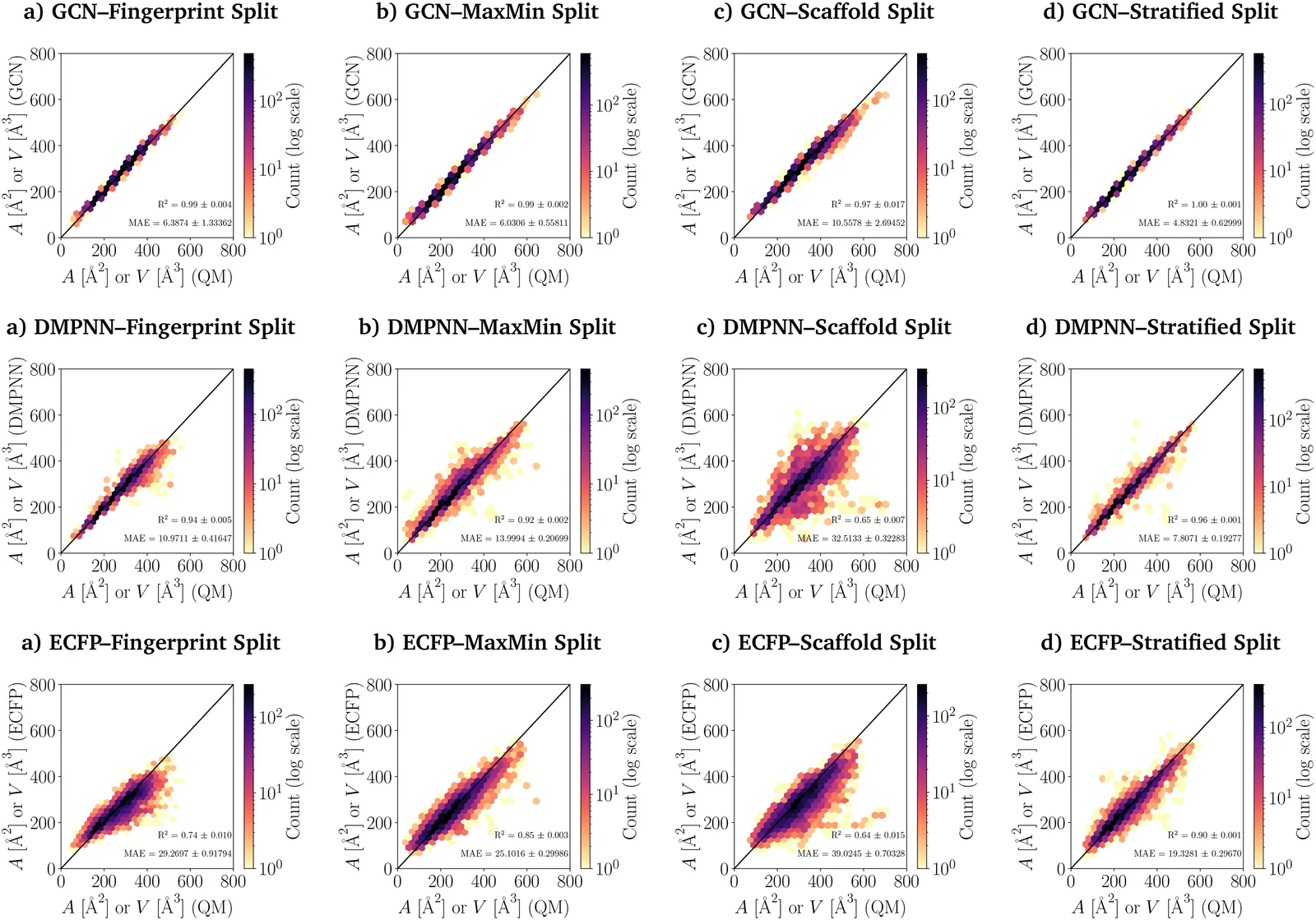
Parity plots for predictions of *V* and *A* across three machine learning models (GCN, DMPNN, ECFP–MLP) and four train-test splitting strategies (Fingerprint, MaxMin, Scaffold, and Stratified). Each subplot compares model predictions to QM COSMO reference values for the test set. Hexagonal bins indicate the density of predictions, with color intensity representing the number of data points (see color bar).

The Fingerprint splitting strategy represents the most challenging evaluation scenario, as expected, resulting in the lowest model accuracy across all three models. However, the performance degradation is modest, and the relative ranking of model performance remains consistent regardless of the splitting method, validating the robustness of our conclusions. The consistency of model performance trends across different splits demonstrates that the observed differences between models are genuine and not artifacts of a particular data partitioning strategy.

For the probability of the charge density properties (*p*(*σ*) and its contributions), the DMPNN model outperforms the GCN model across all splitting strategies. The superior performance of DMPNN for the probability of the surface charge density prediction stems from its directed bond-based message-passing architecture, which naturally captures the anisotropic and local nature of surface charge distributions. Surface charge densities are inherently heterogeneous, varying dramatically between polar (O, N) and non-polar (C) regions of molecules, and depend sensitively on local chemical environments, polarizability, and hydrogen-bonding propensity. By passing messages along directed bonds, DMPNN preserves information about bond asymmetry and can distinguish between different chemical contexts along individual bonds, enabling more nuanced representation of these complex local phenomena.

By contrast, the GCN model demonstrates superior predictive performance for the geometric properties, surface area (*A*) and cavity volume (*V*), across all splitting strategies. This performance advantage reflects the additive and global nature of these quantities. The surface area and volume are fundamentally bulk properties that scale with molecular size and result from the aggregation of atomic contributions. The node-based aggregation mechanism of GCNs, which pools information globally across all atoms *via* sum and max operations, is naturally aligned with this additive property. The simpler pooling operation proves sufficient because these properties lack the local chemical specificity required for charge distribution prediction.

A comprehensive evaluation of model performance across all six COSMO-based descriptors is provided in Fig. 7, in which *R*^2^ scores for test-set predictions are displayed. The *R*^2^ analysis confirms that DMPNN achieves the best performance for *p*(*σ*) and its contributions (*p*^nhb^(*σ*), *p*^oh^(*σ*), and *p*^ot^(*σ*)), while GCN achieves superior results for *A* and *V* across all splitting strategies. The small error bars in the bar plot, derived from the 5-fold cross-validation standard deviations, demonstrate the stability and consistency of model performance across different data folds, indicating that the observed performance differences are reliable and reproducible. The *R*^2^ values for *p*^oh^(*σ*) and *p*^ot^(*σ*) are lower than those for *p*^nhb^(*σ*). This is because many compounds have no OH or OT hydrogen-bonding interactions, meaning their *p*^oh^(*σ*) and *p*^ot^(*σ*) contributions are identically zero. The models handle these zero cases well, but struggle more with compounds that do have non-zero contributions – simply because such compounds are less common in the training data, leaving the models with fewer examples from which to learn these features. Despite these challenges, the GNN architectures consistently outperform the baseline ECFP–MLP model across all six properties and all splitting strategies, demonstrating the superior ability of GNN architectures to extract and learn generalizable molecular features compared to conventional fingerprint-based approaches.

**Fig. 7:**
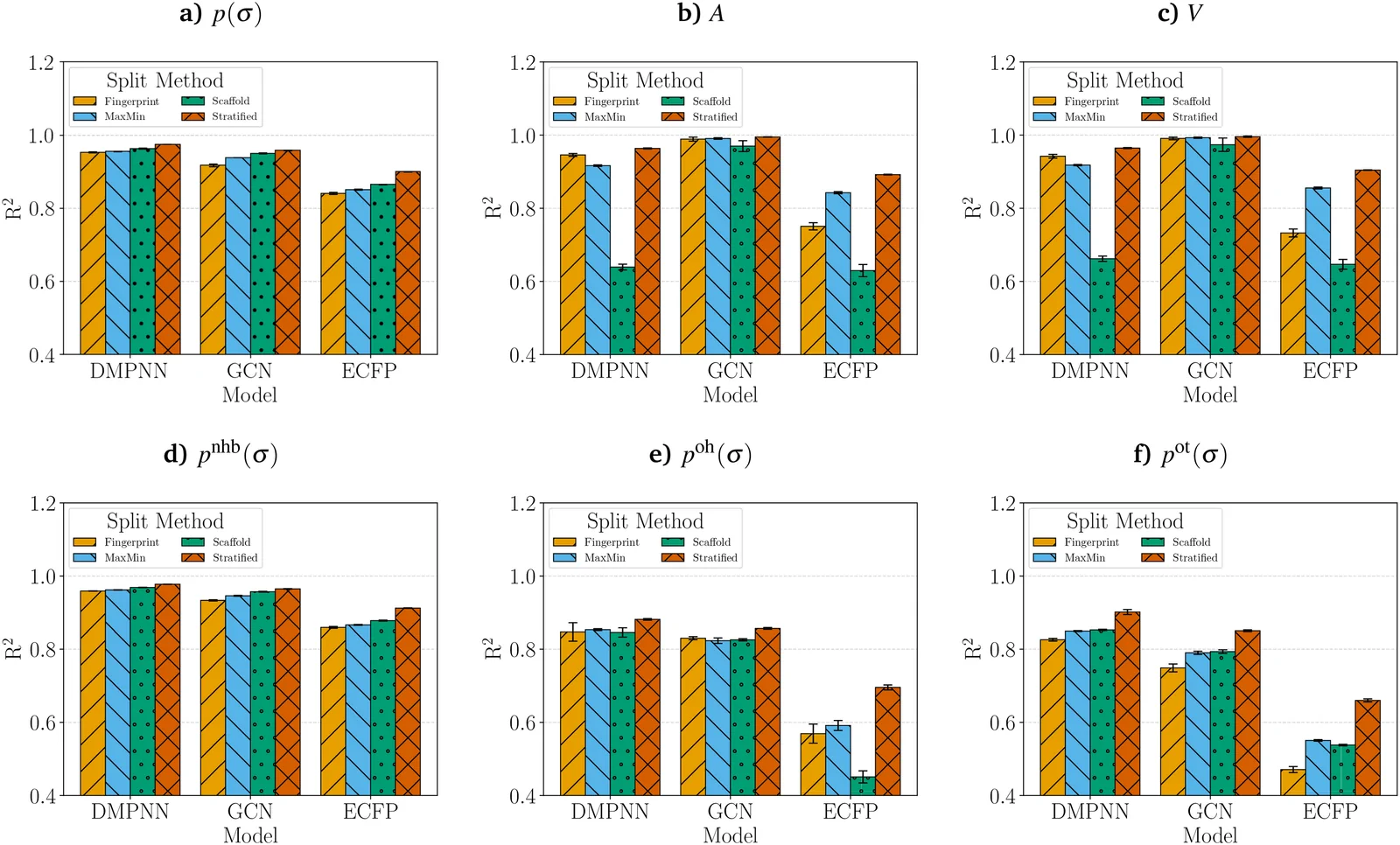
Comparison of test set *R*^2^ scores across GCN, DMPNN, and ECFP–MLP models for predicting six COSMO-based molecular descriptors: a) *p*(*σ*), b) *A* (surface area), c) *V* (volume), d) *p*^nhb^(*σ*) (non-hydrogen bonding), e) *p*^oh^(*σ*) (oh-type hydrogen bonding), and f) *p*^ot^(*σ*) (ot-type hydrogen bonding). Bar plots display model performance across different train-test splitting strategies, with bars representing mean *R*^2^ values and error bars indicating 5-fold cross-validation standard deviation.

### 3.3. Detailed evaluation of model accuracy

Although the differences in accuracy between the DMPNN and GCN models are relatively small, it is valuable to examine the predictions more closely. This closer inspection reveals both the strengths and limitations of each model and offers a more tangible understanding of the parity plots and of the significance of the reported MAEs.

To examine the transferability of the models beyond their training set, only compounds in the test set are considered. The compounds are categorized using two criteria and the error of each model is evaluated for each. The criteria considered are the number of heavy atoms and the types of atoms (similar to grouping of compounds in Fig. 4). For the assessment of the prediction of *p*(*σ*), *p*^nhb^(*σ*), *p*^ot^(*σ*), and *p*^oh^(*σ*), where all the data are between 0 to 0.494, the MAE is used as the error function. For the surface area and the cavity volume, where the range of data is 0 to 700 Å^2^ and 0 to 700 Å^3^, respectively, the AAD% is used as the error function.

In Fig. 8, we present a comparison of the error (the specific error function is shown in the figure) in the predictions of the GCN, DMPNN, and ECFP–MLP models against the number of heavy atoms in the structure of the molecules *via* a series of box plots. From this comparison further information on the accuracy and relative performance of the models as molecular size increases is revealed.

**Fig. 8:**
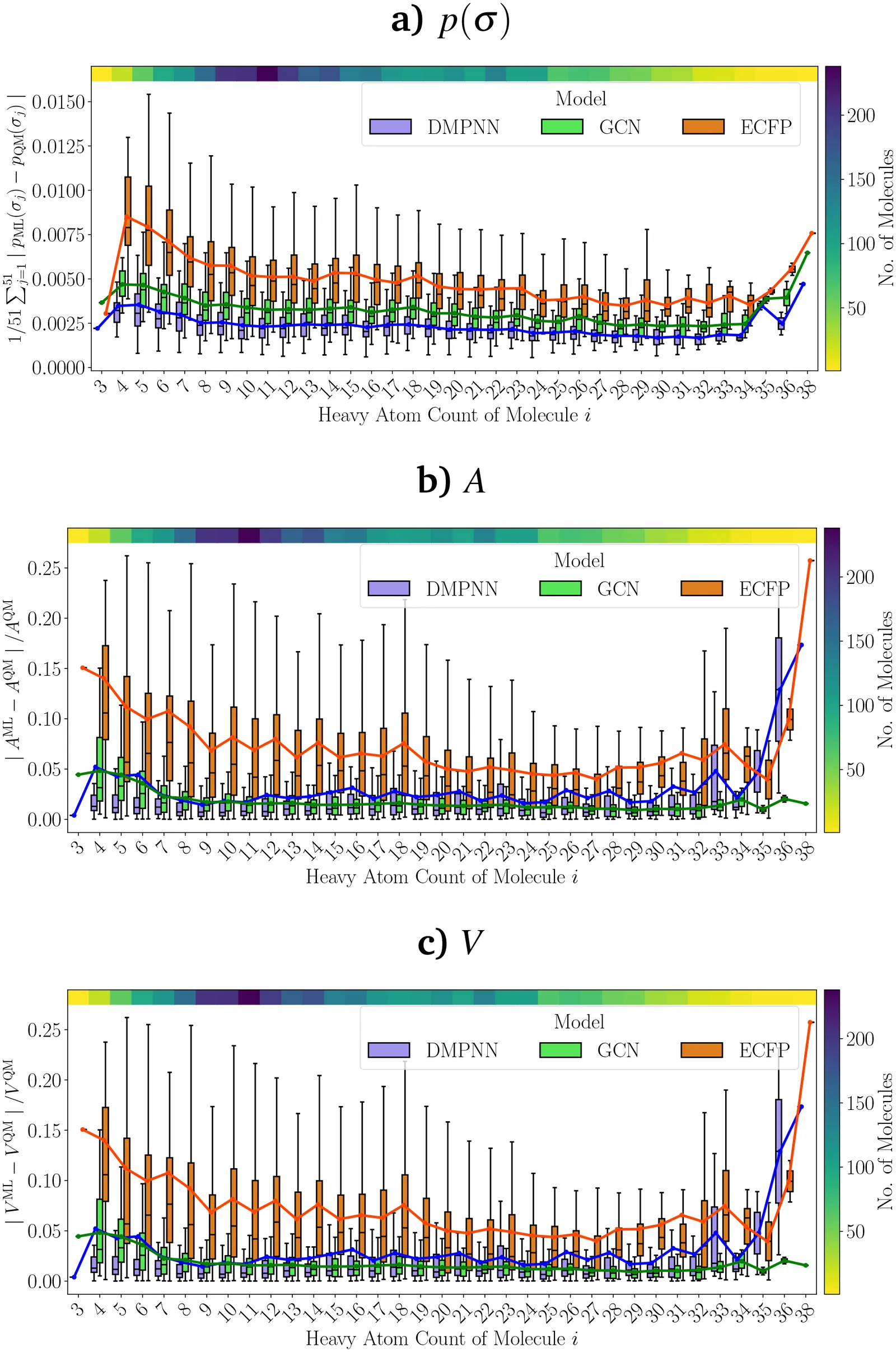
Prediction error comparison between GCN, DMPNN, and ECFP–MLP models for (a) *p*(*σ*), (b) *A*, and (c) *V* as a function of molecular heavy atom count. Results shown are for the test set using stratified train-test splitting; results for other splitting strategies are provided in the SI. Boxes show error distributions, with black horizontal lines indicating medians and colored points (green for GCN, blue for DMPNN, and orange for ECFP–MLP) showing means connected by lines. A heatmap above each plot displays the number of compounds in each heavy atom bin.

As shown in Fig. 8, the prediction errors of both models for *p*(*σ*) decrease when the number of heavy atoms in the structure of the molecule increases. In addition, both the mean and median errors of the DMPNN model are slightly lower than those of the GCN model for all the cases. The mean and median of error for the GCN and DMPNN models are significantly lower than the ECFP–MLP baseline model. The size of the box and also cap line decrease when the heavy atom count increases, indicating a narrower distribution of errors. This is not, however, a definite sign of increased accuracy for larger molecules since the number of compounds with a higher number of heavy atoms is relatively low, as shown in the colormap on the top of the figures. Similar trends are observed for *p*^nhb^(*σ*), *p*^oh^(*σ*), and *p*^ot^(*σ*), (see figures in the SI), with the DMPNN model consistently outperforming the GCN model.

The findings for the predictions of surface area and cavity volume show markedly different error patterns across models, as shown in Fig. 8. The GCN model exhibits low prediction errors across almost all molecular sizes, maintaining stable and narrow error distributions regardless of heavy atom count. This consistent accuracy is observed across all four splitting strategies, with results for the remaining splits provided in the SI.

By contrast, both the DMPNN and ECFP–MLP baseline models exhibit larger prediction errors for small and large molecules, with error curves displaying a characteristic “boat” shape, elevated errors at both extremes of the molecular size distribution. This behavior is reflected in the larger box heights for both models, indicating broader error distributions and reduced predictive accuracy compared to GCN. Across all splitting methods, a clear hierarchy in model performance emerges: GCN substantially outperforms DMPNN, which in turn significantly outperforms the ECFP–MLP baseline.

The large performance gap between the GNN architectures and the baseline model further demonstrates the superior capability of GNN architectures to capture the relationships governing geometric molecular properties. Notably, the better scalability observed for the GCN model, which concatenates sum and max pooling as its graph readout, compared to the DMPNN model, which relies solely on sum pooling, suggests that the choice of graph readout function is critically determines the model output. The scalability of these models with respect to molecular size is an important consideration studied in prior work; Chen *et al.*^45^ demonstrated that the choice of graph readout method is a critical source of information loss, as pooling atom-level representations into a single molecular vector can obscure size-dependent contributions to molecular properties.

Each readout strategy introduces a distinct form of information loss. Mean pooling averages all atom representations, discarding molecule size information and making it unsuitable for extensive properties that scale with atom count. Sum pooling preserves size information but causes the molecular representation to shift in latent space as molecule size grows, turning predictions on larger molecules into an extrapolation problem. Max pooling retains only the most activated feature across all atoms, ignoring the collective chemical context of the molecule. These trade-offs are consistent with the theoretical ranking of Xu *et al.*,^46^ who showed that sum pooling captures the full multiset of node features, mean pooling captures only the distribution of element types, and max pooling reduces the multiset to a simple set by ignoring multiplicities entirely – making sum pooling the most expressive of the three.

Concatenating sum and max pooling, as done in the GCN model, partially mitigates these shortcomings by combining size sensitivity with the retention of dominant structural features. This ensemble approach is supported by Binkowski *et al.*,^47^ who showed that composing multiple aggregators improves expressiveness beyond any single readout, and by Wang *et al.*,^48^ who identified concatenation of mean and max pooling as the most widely adopted cascade readout strategy in the GNN literature. More broadly, Schweidtmann *et al.*^49^ argued that the pooling selection should be guided by the physical nature of the target property, and that inappropriate choices can yield nonphysical models that generalize poorly – directly relevant to the performance differences observed here between extensive properties such as cavity volume and intensive ones such as surface charge distribution.

Several alternatives to global readout have also been proposed. Chen *et al.*^45^ predicted each atom's contribution independently using a shared feed-forward network, so that the molecular property emerges from the sum of atomic outputs rather than a single global aggregation, improving generalization to larger molecules without increasing parameter count. Buterez *et al.*^50^ further argued that fixed readout functions create a rigid coupling between node-level and graph-level representations, and proposed a learnable Set Transformer-based readout that adapts to the prediction task while preserving permutation invariance. Bianchi and Grattarola^51^ provided theoretical grounding for these findings, deriving sufficient conditions for a pooling operator to preserve the expressive power of the preceding message-passing layers, and empirically confirming that sum pooling outperforms max pooling in graph discriminability.

In agreement with our previous observations, both models demonstrate similar predictive accuracy for the probability densities (*p*(*σ*), *p*^nhb^(*σ*), *p*^oh^(*σ*), and *p*^ot^(*σ*)), with the DMPNN model showing a small systematic advantage. On the other hand, the predictions of the GCN model are more accurate for *A* and *V*. Both GNN architectures outperform the ECFP–MLP baseline model for all the properties. Further analysis of the performance of the model based on the types of atoms present in the molecule (see figures in the SI) confirms this behavior. However, it should be noted that the dataset exhibits heterogeneity; compounds containing C, N, and O atoms predominate, while those with less common heteroatoms, such as S and P, are underrepresented (*cf.* SI). In Fig. S18–S23, we study the impact of different atomic constituents on model performance, revealing that while not conclusively showing higher deviations for low-proportion heteroatoms, a rough trend can be observed where compounds containing S and P atoms present more challenging prediction tasks. These limitations could potentially be addressed through pre-training strategies, data augmentation techniques, or employing weighted loss functions during model training to improve the model's universality across diverse chemical compositions.

To explore the impact of molecular size variation further, we present a graphical comparison of predicted *vs.* actual *p*(*σ*) or *p*^nhb^(*σ*), *p*^oh^(*σ*), and *p*^ot^(*σ*) for the 16 130 compounds in the database (GitHub and Zenodo repositories). We show a sample of these in Fig. 9 by randomly selecting eight compounds of different complexity indices and displaying their *p*(*σ*) plots. The eight compounds are drawn from the test set using the stratified split method. In all cases, the predictions of the GCN and DMPNN models are acceptable. The main peaks of *p*(*σ*) are captured, although, in some cases, there is a quantitative disagreement on the height of the peaks. In contrast, the ECFP–MLP baseline model demonstrates substantial limitations, frequently failing to reproduce both the qualitative features and quantitative magnitudes of the predicted *p*(*σ*) across the complexity range examined.

**Fig. 9:**
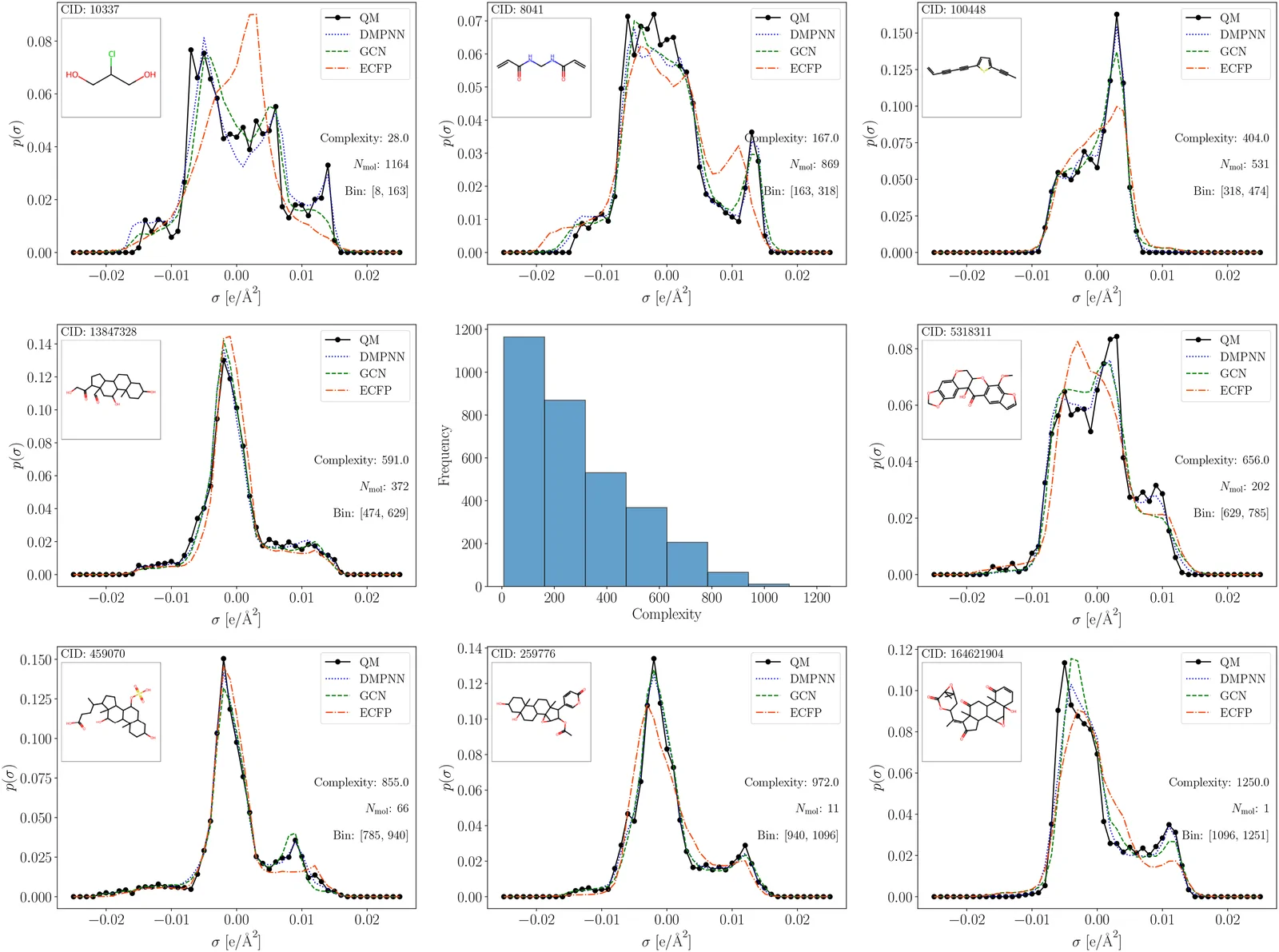
Comparison of actual (QM-calculated), GCN-predicted, DMPNN-predicted, and ECFP–MLP-predicted *p*(*σ*) values for eight randomly selected molecules, each from a different bin of the molecular complexity index distribution (the plot in the center of the figure). For each molecule (drawn randomly from each bin of the complexity index distribution), the chemical ID (on the top left of the plots), molecular structure (on the top lef of the plots), complexity index value (on the bottom right of the plots), bin size (*N*_mol_) (on the bottom right of the plots), and bin range (on the bottom right of the plots) are displayed.

Further profiles are presented in the SI, selecting molecules based on the MAE of *p*(*σ*), the molecular weight, and the heavy atom count. Profiles for the contributions *p*^nhb^(*σ*), *p*^oh^(*σ*), and *p*^ot^(*σ*) are shown in Fig. S29–S31. In all cases, a similar level of agreement is observed as in Fig. 9.

Consistent with our earlier findings, both the DMPNN and GCN models achieve comparable predictive accuracy for surface charge density properties (*p*(*σ*), *p*^nhb^(*σ*), *p*^oh^(*σ*), and *p*^ot^(*σ*)), with DMPNN providing a consistent yet modest performance advantage. Conversely, the GCN model delivers superior predictions for the geometric properties, surface area (*A*) and cavity volume (*V*). Both GNN architectures substantially outperform the ECFP–MLP baseline across all properties. A detailed analysis of model performance stratified by molecular atom types (provided in the SI) reinforces these findings, revealing that the performance differences are robust across different chemical compositions.

These results motivate the development of a hybrid model, COSMO-NET, that leverages the complementary strengths of both architectures: DMPNN is employed to predict surface charge density distributions (*p*(*σ*) and its individual contributions), while GCN is used to predict the geometric properties (*A* and *V*). This hybrid approach is designed to maximize predictive accuracy by assigning each model to the property class for which it demonstrates superior performance.

### 3.4. Evaluating the effect of using ML-generated COSMO descriptors on downstream thermodynamic modeling

Having presented our hybrid COSMO-NET model for the prediction of *p*(*σ*), *p*^nhb^(*σ*), *p*^oh^(*σ*), and *p*^ot^(*σ*) volume and area, we examine the impact of descriptor prediction errors on two thermodynamic properties of significant practical importance: the octanol–water partition coefficient and solid–liquid solubility.

#### 3.4.1. Octanol–water partition coefficient

At 298.15 K and 1 bar, the octanol–water partition coefficient is defined as:^52^18
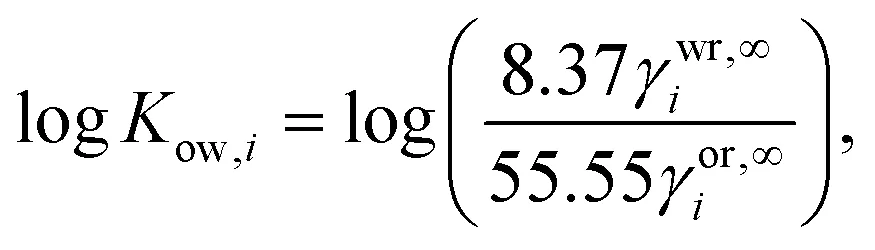
where *γ*^wr,∞^_*i*_ and *γ*^or,∞^_*i*_ are the activity coefficients of compound *i* at infinite dilution, in a water-rich phase, and octanol-rich phase, respectively, at specified conditions. To predict the activity coefficient in the liquid phase, the COSMO-SAC 2002^16^ and COSMO-SAC 2010^22^ models are used (the equations of COSMO-SAC 2002 and 2010 can be found in ref. 16, 22 and 40). The additional parameters of both COSMO-SAC models are taken as the B3LYP-6-31G(d,p)-COSMO parameters in the work of Chen *et al.*^41^ and summarized in Tables S1 and S2 in the SI.

As outlined in ref. 40, *p*(*σ*), *A*, and *V* are required for the prediction of the activity coefficient in the liquid phase using the COSMO-SAC 2002 model. For COSMO-SAC 2010, on the other hand, *p*^nhb^(*σ*), *p*^oh^(*σ*), *p*^ot^(*σ*), *A* and *V* are required. Predictions are performed for all test set compounds across each of the four train-test splitting strategies (Fingerprint, MaxMin, Scaffold, and Stratified). Detailed parity plots comparing COSMO-SAC predictions using ML-derived descriptors to those using QM COSMO reference values are provided in the SI. A summary comparison of model performance is presented in Fig. 10, which displays *R*^2^ and MAE scores across all splitting strategies.

**Fig. 10:**
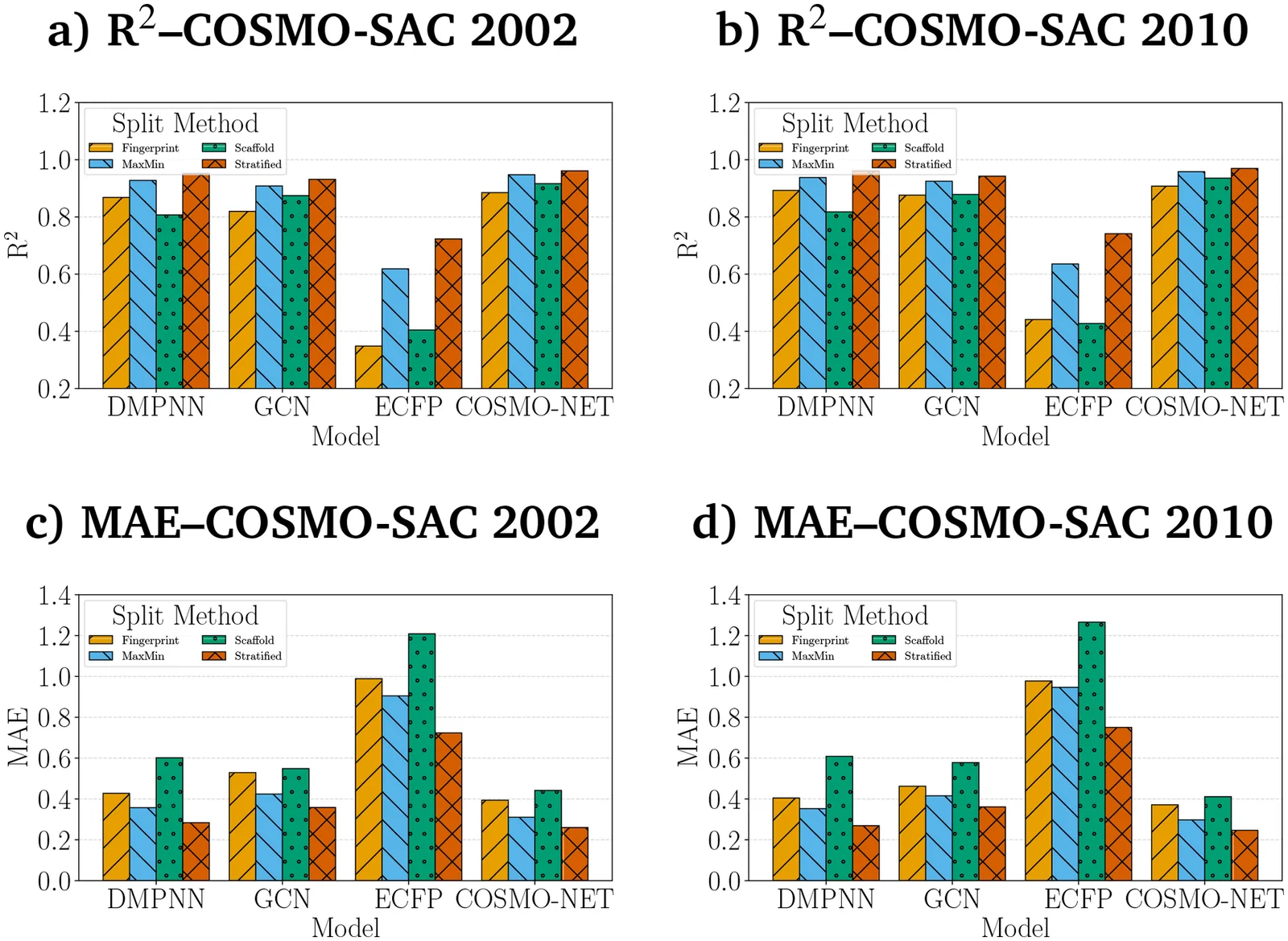
Comparison of test set (10% of the compounds) *R*^2^ and MAE scores across GCN, DMPNN, ECFP–MLP, and COSMO-NET models for predicting octanol–water partition coefficient of compounds using COSMO-SAC 2002 (a and c) and COSMO-SAC 2010 (b and d). Bar plots display model performance across different train–test splitting strategies, with bars representing *R*^2^ or MAE.

For both COSMO-SAC 2002 and 2010, COSMO-NET consistently achieves the best predictive accuracy across all splitting strategies, with the highest *R*^2^ and lowest MAE values. ECFP–MLP ranks last in all cases, reflecting the limitations of fixed fingerprint-based representations compared to end-to-end trained graph models. The only notable variation in rankings occurs under the Scaffold split, where GCN outperforms DMPNN for both activity coefficient models – suggesting that the GCN readout generalizes better to structurally dissimilar molecules, which are precisely the compounds isolated by scaffold-based splitting.

A notable observation is that although the Scaffold splitting strategy presents the most challenging evaluation scenario, exhibiting the highest prediction errors across all models, the COSMO-NET model provides remarkable robustness, showing negligible performance variation across splitting methods. In contrast, the standalone GCN, DMPNN, and baseline ECFP–MLP models exhibit larger performance fluctuations depending on the splitting strategy employed. The detailed parity plots provided in the ESI further confirm these observations, validating the superiority of the COSMO-NET hybrid approach. Further analysis of the predictive capabilities of the models as a function of heavy atom count (see figures in the SI) reveals that the relative accuracy of the models is preserved (COSMO-NET > DMPNN > GCN > ECFP–MLP) for both COSMO-SAC 2002 and 2010. These results demonstrate that COSMO-NET can be reliably trusted for accurate prediction of octanol–water partition coefficients across diverse molecular structures and data splitting scenarios.

#### 3.4.2. Solid–liquid solubility

Solid–liquid solubility predictions are performed using the COSMO-SAC 2002 and 2010 thermodynamic models. The solubility of a solid in a liquid solvent at a given temperature and pressure is described given by Prausnitz *et al.*:^53^19
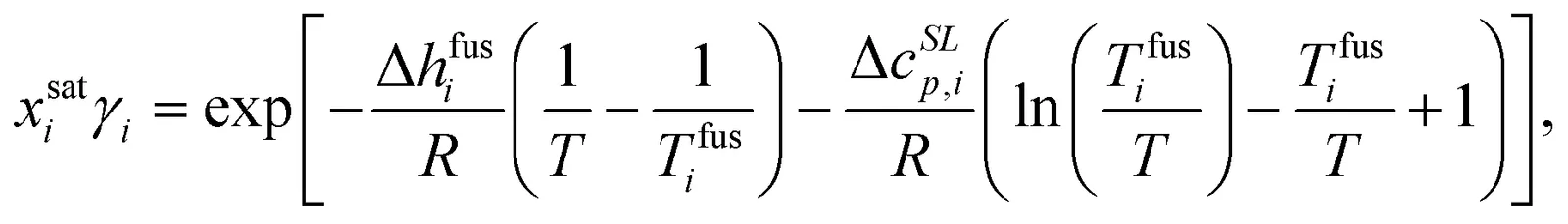
where *x*^sat^_*i*_ is the mole fraction solubility, *γ*_*i*_ is the activity coefficient of the solute at solubility composition, and Δ*h*^fus^_*i*_, *T*^fus^_*i*_, Δ*c*^*SL*^_*p*,*i*_ = *c*^L^_*p*,*i*_ − *c*^S^_*p*,*i*_ are the enthalpy of fusion, fusion temperature, and the difference between the isobaric heat capacity in the liquid and solid phases of compound *i*, respectively. In this work, the enthalpy of fusion and fusion temperature are obtained from Landolt-Börnstein,^54^ Acree and Chickos,^55–57^ and ThermoML,^58^ and due to the lack of data Δ*c*^*SL*^_*p*,*i*_ is neglected. The specific values are presented in the SI. Solubility predictions are computed for 1096 compounds (in which the corresponding fusion properties could be found) across 68 solvents including water, 12 alcohols, 4 ketones, 10 esters, 7 ethers, 8 hydrocarbons, 5 halogenated, 12 aprotic polar, 3 acids, 2 amines, and 4 miscellaneous (see SI for the list of solvents) at 298.15 K and 1 bar using COSMO descriptors (*p*(*σ*), *A*, and *V* for COSMO-SAC 2002 and *p*^nhb^(*σ*), *p*^oh^(*σ*), *p*^ot^(*σ*), *A*, and *V* for COSMO-SAC 2010) derived from the GCN, DMPNN, ECFP–MLP, and COSMO-NET models. Reference solubility values are calculated using *p*(*σ*), *A*, and *V* for COSMO-SAC 2002 and *p*^nhb^(*σ*), *p*^oh^(*σ*), *p*^ot^(*σ*), *A*, and *V* for COSMO-SAC 2010 using QM COSMO calculations.

The performance of each model in predicting solid–liquid solubility is summarized in Fig. 11, which displays *R*^2^ and MAE scores for test set predictions across all four train-test splitting strategies. Due to incomplete fusion property data in the database, the number of compounds available for solubility prediction varies across splitting methods: the Fingerprint split contains 406 compounds, MaxMin split 146 compounds, Scaffold split 221 compounds, and Stratified split 208 compounds, with a total of 1096 compounds analyzed across all splits. Predictions are performed for all available test set compounds within each splitting strategy. Detailed parity plots comparing solubility predictions from each model to reference QM COSMO calculations are provided in the SI.

**Fig. 11:**
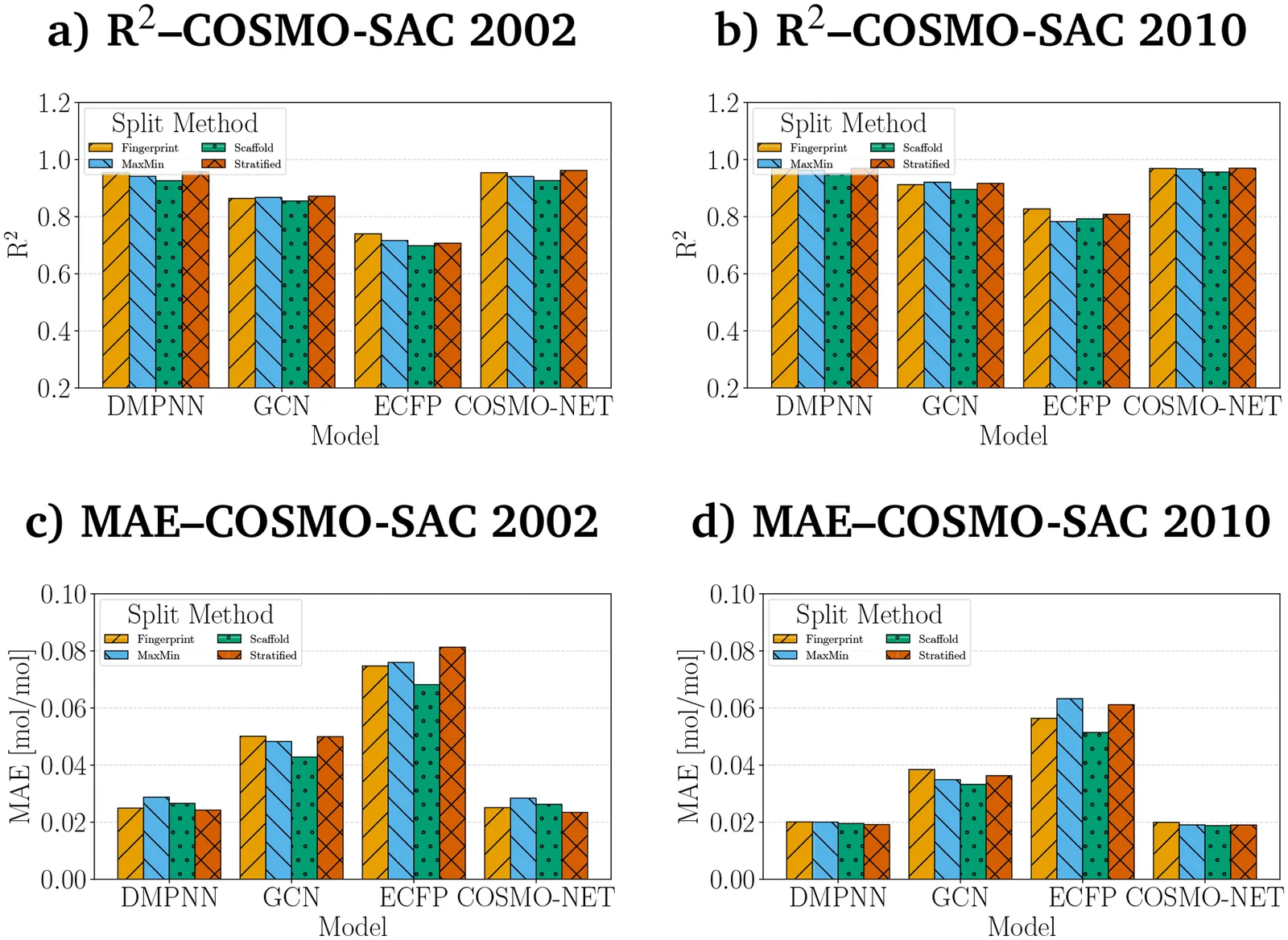
Comparison of test set *R*^2^ and MAE scores across GCN, DMPNN, ECFP–MLP, and COSMO-NET models for predicting solubility of compounds using COSMO-SAC 2002 (a and c) and COSMO-SAC 2010 (b and d). Bar plots display model performance across different train-test splitting strategies, with bars representing *R*^2^ or MAE.

The COSMO-NET hybrid model demonstrates superior predictive accuracy for solid–liquid solubility predictions both in the case of COSMO-2002 and of COSMO-2010. The DMPNN model achieves comparable performance with slightly lower *R*^2^ scores and marginally larger MAE values compared to COSMO-NET. The standalone GCN model exhibits prediction errors approximately two times higher than COSMO-NET, while the ECFP–MLP baseline model demonstrates substantially inferior performance with MAE values roughly three times those of the hybrid model. Notably, the ranking of model performance (COSMO-NET > DMPNN > GCN > ECFP-MLP) is consistent for both COSMO-SAC variants, demonstrating the generalizability of the hybrid approach to different thermodynamic models.

It is also interesting to note that the splitting method exerts minimal influence on the accuracy of the solubility prediction, with no significant performance variation observed across the four splitting strategies. The COSMO-NET and DMPNN models exhibit the least variation in performance across different splits, further highlighting their robustness for the prediction of solubility.

## 4. Conclusions

In the present work, we have developed and benchmarked machine-learning surrogate models for predicting COSMO-based molecular descriptors, including *p*(*σ*) (along with its *p*^nhb^(*σ*), *p*^oh^(*σ*), and *p*^ot^(*σ*) components), molecular surface area (*A*), and cavity volume (*V*), with the aim of replacing computationally expensive QM calculations. To train these models, we have compiled a database of COSMO files and corresponding sigma files for 16 130 compounds, generated through an automated quantum-chemical calculation pipeline. The dataset comprises molecules with molecular weights below 500 g mol^−1^, complexity scores under 1500, and up to 38 heavy atoms, selected from a pool of eight elements: C, N, O, P, S, F, Cl, and Br.

We have implemented two types of graph-based surrogate models, graph convolutional network and directed message passing neural network, to predict the COSMO-based molecular descriptors used in both the COSMO-SAC 2002 (*p*(*σ*), *A*, *V*) and COSMO-SAC 2010 (*p*^nhb^(*σ*), *p*^oh^(*σ*), *p*^ot^(*σ*), *A*, *V*) activity coefficient models. While both models achieve reasonable accuracy, the DMPNN model consistently outperforms GCN in predicting the various forms of *p*(*σ*), whereas GCN exhibited superior performance in predicting surface area and cavity volume. We have thus proposed a hybrid model, COSMO-NET, in which *p*(*σ*) (along with *p*^nhb^(*σ*), *p*^oh^(*σ*), *p*^ot^(*σ*)) is obtained from the DMPNN model and *A* and *V* from the GCN model.

To evaluate the practical impact of using surrogate models on thermodynamic property prediction, we have assessed model performance in terms of the prediction of the octanol–water partition coefficient and solid–liquid solubility. For the octanol–water partition coefficient predictions for all the compounds in the database, the hybrid COSMO-NET model is seen to outperform the others considered. Similarly, for solid–liquid solubility predictions across 1096 compounds in 68 different solvents, COSMO-NET demonstrated superior performance, while DMPNN achieved comparable results with marginally larger errors. Notably, COSMO-NET demonstrated robustness across different train-test splitting strategies and showed minimal performance variation in the solubility predictions regardless of the data partitioning method.

By replacing computationally expensive QM COSMO calculations with rapid ML predictions, the COSMO-NET model presented here substantially reduces computational time, enabling accelerated molecular design workflows and exploration of a large chemical spaces. We expect COSMO-NET to become an invaluable tool relevant for molecular and process design applications.

## Author contributions

Saman Naseri Boroujeni: conceptualization, methodology, software, validation, investigation, data curation, writing – original draft, visualization, funding acquisition. Amparo Galindo: conceptualization, methodology, validation, writing – review & editing, supervision, funding acquisition. George Jackson: conceptualization, methodology, validation, writing – review & editing, supervision, funding acquisition. Claire S. Adjiman: conceptualization, methodology, validation, writing – review & editing, supervision, project administration, funding acquisition.

## Conflicts of interest

There are no conflicts to declare.

## Supplementary Material

ME-011-D6ME00088F-s001

## Data Availability

The data supporting the findings of this study are available in three locations. The supplementary information (SI) provides the COSMO-SAC 2002 and COSMO-SAC 2010 parameter sets used in this work, together with extended accuracy analyses including parity plots, error probability density functions, boxplots, and heatmaps. The source code for COSMO-NET, including model architectures, training and inference scripts, is available in a GitHub repository (https://github.com/snaserib/COSMO-NET-Paper). The datasets and model artifacts required to reproduce the reported results are archived on Zenodo (https://doi.org/10.5281/zenodo.21265695). Supplementary information: full parameter sets for the COSMO-SAC 2002 and 2010 models; data-splitting diagnostics, including property distributions across the training, validation and test sets, atom-pair count heatmaps, and UMAP visualisations of the four splitting strategies; training results comprising error metrics and parity plots for the predicted sigma profiles (*p*(σ), *p*^nhb^(*σ*), *p*^oh^(*σ*), *p*^ot^(*σ*)) and molecular surface area and cavity volume across all models and splits; a detailed accuracy assessment with error distributions by heavy-atom count, spatial error heatmaps in chemical space, and representative molecule-level sigma profile comparisons; and an evaluation of the downstream effect of ML-generated COSMO descriptors on the prediction of octanol–water partition coefficients and solid–liquid solubilities using COSMO-SAC 2002 and 2010. See DOI: https://doi.org/10.1039/d6me00088f.
