# Supplementary material for: COSMO-NET: fast and accurate machine learning surrogates for COSMO-based molecular descriptors
Source: Mol Syst Des Eng. 2026 Jul 1;11(10):983–98. doi: 10.1039/d6me00088f (PMC13637804; doi:10.1039/d6me00088f)
Supplement: Supplementary file 1 [file ME-011-D6ME00088F-s001.pdf]

Supplementary Information (SI) for Molecular Systems Design & Engineering.
This journal is © The Royal Society of Chemistry 2026

Electronic Supporting Information for

COSMO-NET: Fast and Accurate Machine Learning Surrogates for COSMO-based Molecular Descriptors

Saman Naseri Boroujeni*[1], Amparo Galindo[1], George Jackson[1], and Claire S. Adjiman*[1]

[1]Department of Chemical Engineering, Sargent Centre for Process Systems Engineering, Institute for Molecular Science and Engineering, Imperial College London, South Kensington Campus, London SW7 2AZ, United Kingdom.

In this document, we present further details required in the reproduction of the data in the main manuscript and further analysis to support the main findings.

1 Parameters of the COSMO-SAC models

In Tables S1 and S2, we present the parameters of COSMO-SAC 2002 [1] and 2010 [2] models required for the prediction of the activity coefficient in the liquid phase.

Table S1: Parameters for the COSMO-SAC 2002 model [3].

Parameter	Value
q_0	$79.53\,\text{Å}^2$
r_0	$66.69\,\text{Å}^3$
z	10
r_{av}	$0.8176\,\text{Å}$
a_{eff}	$6.4547\,\text{Å}^2$
c_{hb}	$55\,946\,\text{kcal}\,\text{Å}^4\,\text{mol}^{-1}\,\text{e}^{-2}$
σ_{hb}	$0.0084\,\text{e}\text{Å}^{-2}$
f_{pol}	0.7217
R	$0.001\,987\,\text{kcal}\,\text{mol}^{-1}\,\text{K}^{-1}$

Table S2: Parameters for the COSMO-SAC 2010 model [3].

Parameter	Value
a_{eff}	$6.4547\,\text{Å}^2$
A_{ES}	$5501.09\,\text{kcal}\,\text{Å}^4\,\text{mol}^{-1}\,\text{e}^{-2}$
B_{ES}	$1.8822 \times 10^8\,\text{kcal}\,\text{Å}^4\,\text{mol}^{-1}\,\text{e}^{-2}\,\text{K}^{-2}$
$C_{\text{OH-OH}}$	$4900.06\,\text{kcal}\,\text{Å}^4\,\text{mol}^{-1}\,\text{e}^{-2}$
$C_{\text{OT-OT}}$	$1378.62\,\text{kcal}\,\text{Å}^4\,\text{mol}^{-1}\,\text{e}^{-2}$
$C_{\text{OH-OT}}$	$4211.67\,\text{kcal}\,\text{Å}^4\,\text{mol}^{-1}\,\text{e}^{-2}$

*Emails: s.naseri-boroujeni@imperial.ac.uk & c.adjiman@imperial.ac.uk

2 Data splitting

In Figure S1, we present PDFs of molecular weights, complexity indices, A, and V for the training, validation, and test sets. As can be seen, the stratified split resulted in a good distribution of compounds in the training, validation, and test sets. This can be further confirmed in Figure S2.

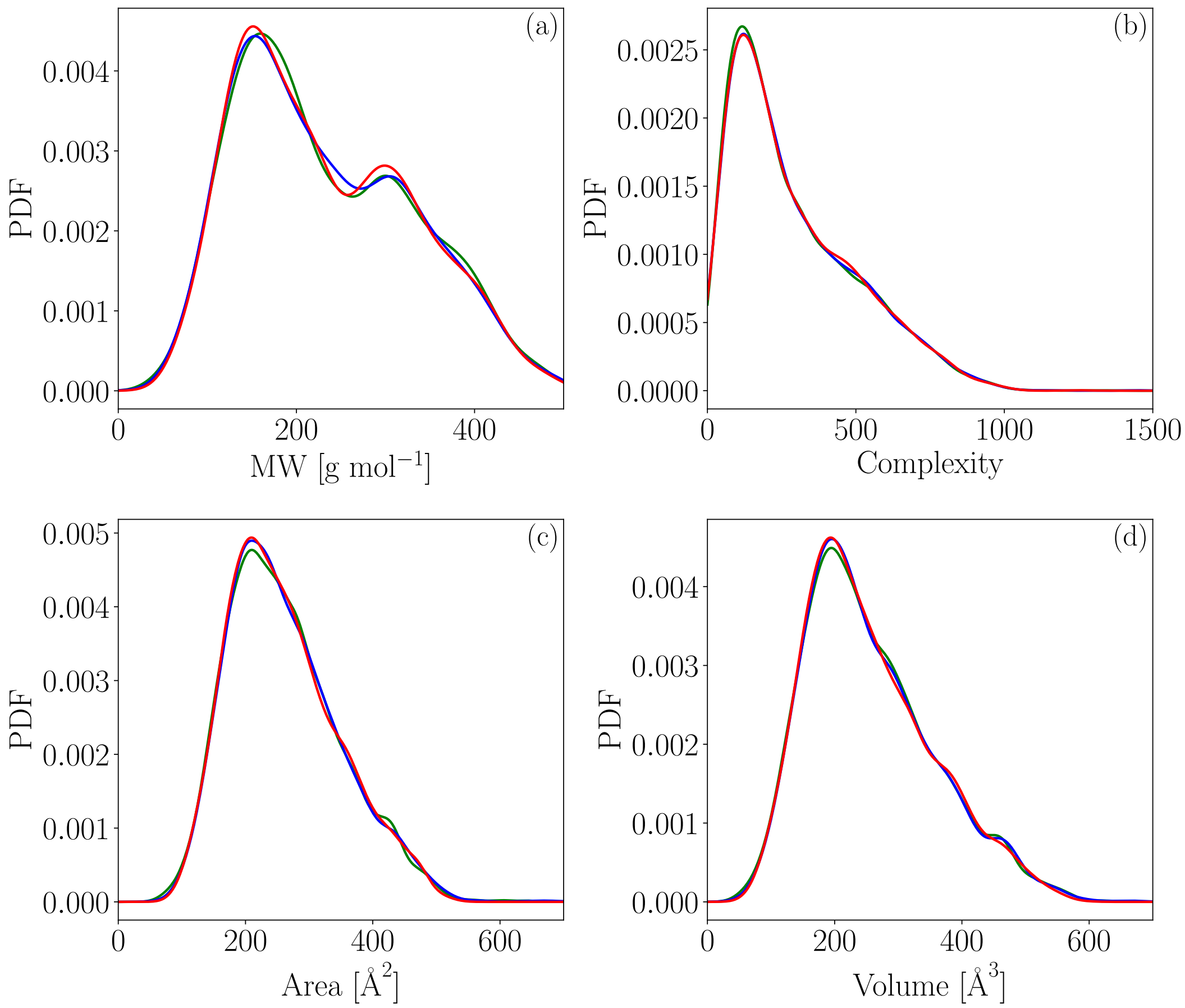

Figure S1: Probability density functions (PDF) of (a) molecular weights, (b) complexity score indices, (c) area, and (d) volume of molecules in the training (green curves), validation (blue curves), and test sets (red curves).

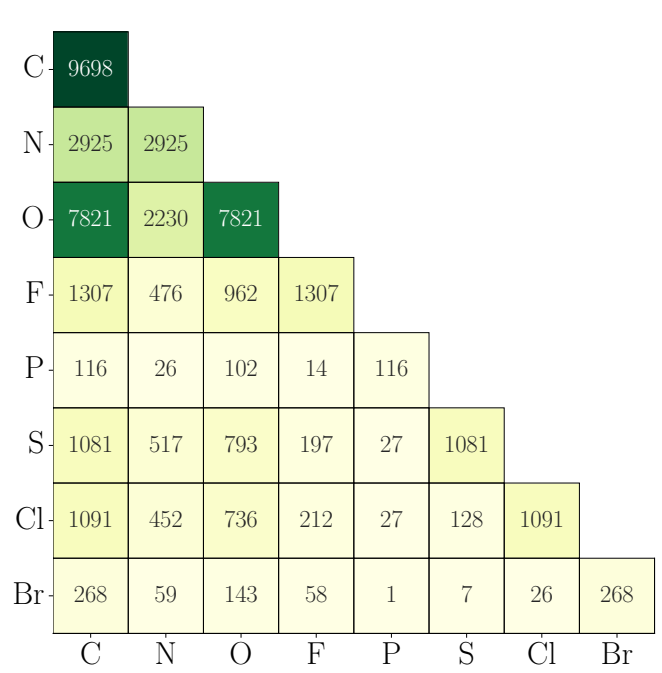

(a) Training Set

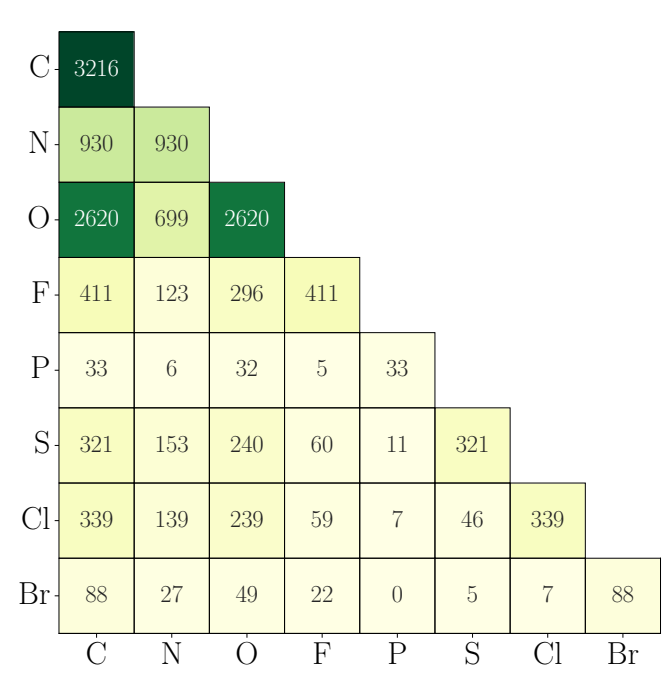

(b) Validation Set

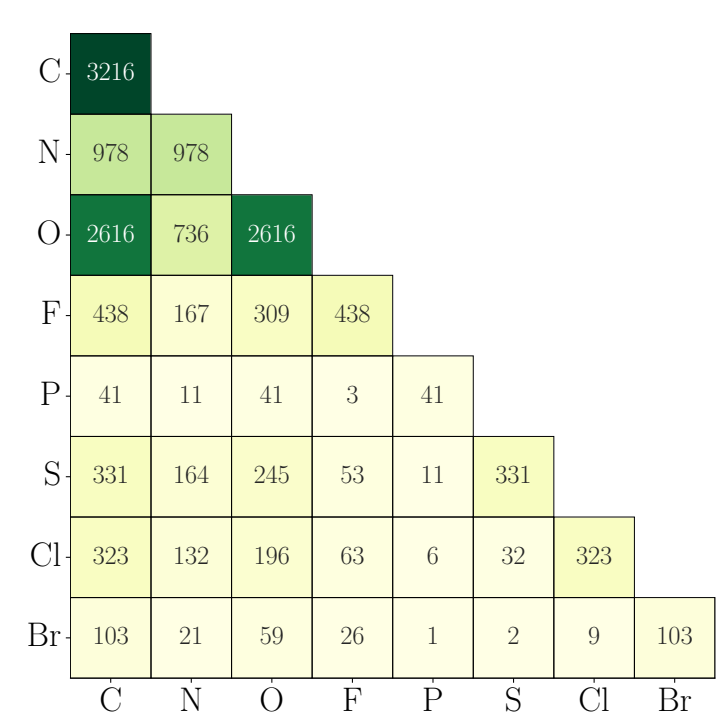

(c) Test Set

Figure S2: Number of molecules that contains both atoms shown in the x- and y-axis (in addition to other atoms) for the (a) training, (b) validation set, and (c) test sets.

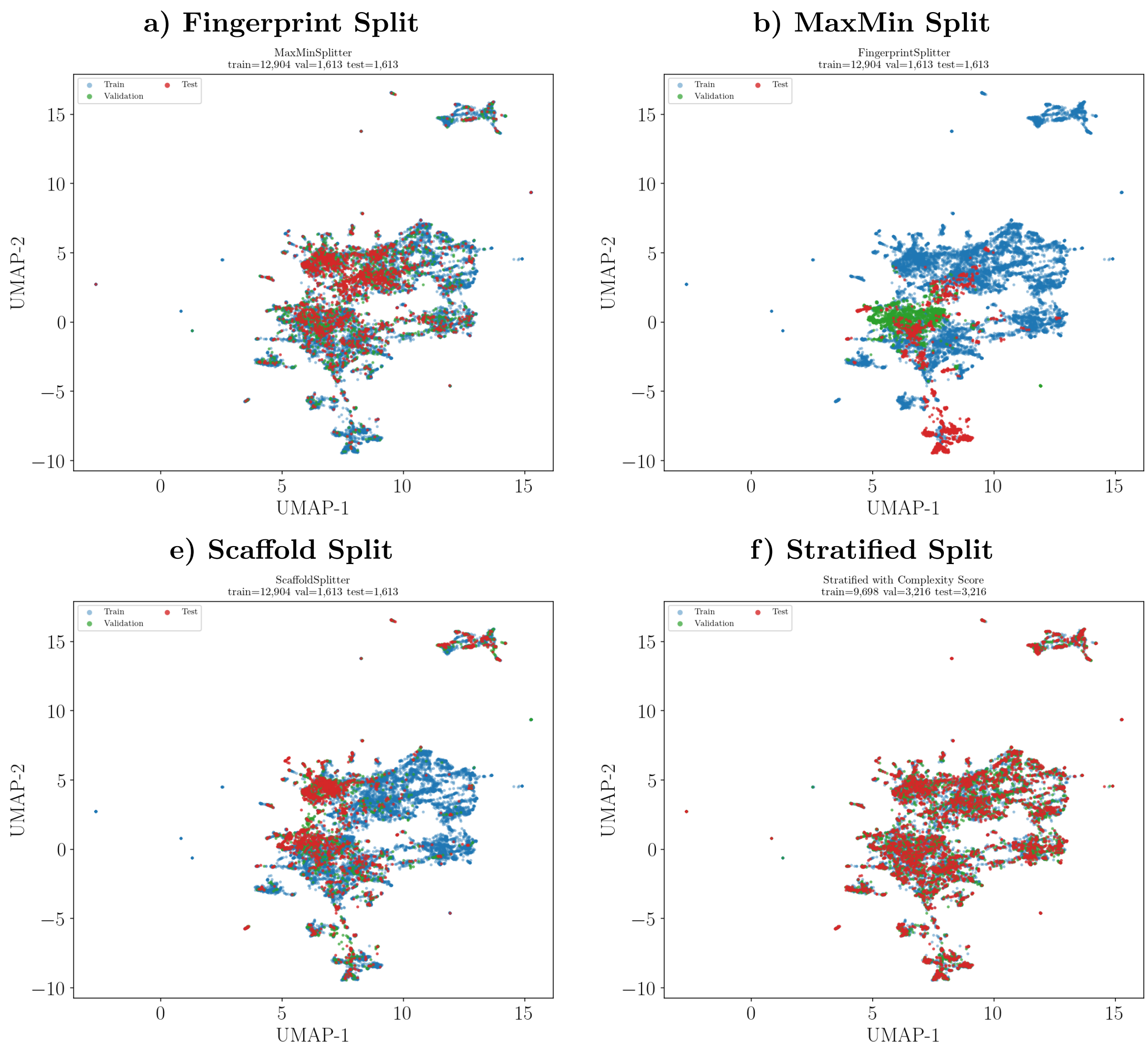

Figure S3: Two-dimensional UMAP visualizations of molecular datasets partitioned using different train/validation/test splitting strategies: **a)** fingerprint-based splitting, **b)** MaxMin diversity-based splitting, **c)** scaffold-based splitting, and **d)** stratified random splitting. The visualizations illustrate how each splitting method distributes molecules in chemical space, with implications for model generalization.

3 Training

Table S3: Error scores of the of the GCN, DMPNN, and ECFP–MLP models for each target property and each split method. MAEs and standard deviations for A and V are in $Å^2$ and $Å^3$ units, respectively, while for probabilities are unitless.

**Split set**	**Model**	**Split method**	**Property**	**MAE**	**AAD%**	$\mathbf{R^2}$
Training	DMPNN	Fingerprint	A	3.13 ± 0.21	1.291 ± 0.069	0.996 ± 0.001
Test	DMPNN	Fingerprint	A	10.34 ± 0.35	3.534 ± 0.078	0.946 ± 0.004
Training	ECFP	Fingerprint	A	8.48 ± 0.77	3.390 ± 0.278	0.968 ± 0.002
Test	ECFP	Fingerprint	A	27.47 ± 0.91	10.139 ± 0.351	0.751 ± 0.010
Training	GCN	Fingerprint	A	4.32 ± 0.50	1.800 ± 0.250	0.996 ± 0.001
Test	GCN	Fingerprint	A	6.20 ± 1.70	2.395 ± 0.686	0.989 ± 0.005
Training	DMPNN	MaxMin	A	3.26 ± 0.29	1.328 ± 0.142	0.996 ± 0.000
Test	DMPNN	MaxMin	A	13.21 ± 0.23	5.391 ± 0.096	0.917 ± 0.002
Training	ECFP	MaxMin	A	8.15 ± 0.40	3.171 ± 0.090	0.969 ± 0.002
Test	ECFP	MaxMin	A	23.96 ± 0.28	10.250 ± 0.055	0.843 ± 0.003
Training	GCN	MaxMin	A	4.33 ± 0.51	1.808 ± 0.222	0.996 ± 0.001
Test	GCN	MaxMin	A	5.97 ± 0.67	2.740 ± 0.295	0.991 ± 0.002
Training	DMPNN	Scaffold	A	2.26 ± 0.21	0.949 ± 0.088	0.998 ± 0.000
Test	DMPNN	Scaffold	A	30.38 ± 0.33	9.749 ± 0.131	0.639 ± 0.008
Training	ECFP	Scaffold	A	7.34 ± 0.37	3.022 ± 0.108	0.971 ± 0.001
Test	ECFP	Scaffold	A	36.31 ± 0.80	11.745 ± 0.208	0.630 ± 0.017
Training	GCN	Scaffold	A	4.20 ± 0.63	1.818 ± 0.274	0.995 ± 0.001
Test	GCN	Scaffold	A	10.40 ± 2.21	3.392 ± 0.677	0.970 ± 0.015
Training	DMPNN	Stratified	A	3.21 ± 0.21	1.303 ± 0.080	0.996 ± 0.000
Test	DMPNN	Stratified	A	7.44 ± 0.15	2.902 ± 0.054	0.964 ± 0.001
Training	ECFP	Stratified	A	8.11 ± 0.34	3.167 ± 0.148	0.971 ± 0.003
Test	ECFP	Stratified	A	18.51 ± 0.28	7.604 ± 0.140	0.892 ± 0.001
Training	GCN	Stratified	A	4.53 ± 0.24	1.897 ± 0.112	0.995 ± 0.001
Test	GCN	Stratified	A	4.64 ± 0.24	1.935 ± 0.117	0.995 ± 0.001
Training	DMPNN	Fingerprint	V	3.18 ± 0.27	1.389 ± 0.103	0.997 ± 0.000
Test	DMPNN	Fingerprint	V	11.60 ± 0.50	3.961 ± 0.108	0.942 ± 0.005
Training	ECFP	Fingerprint	V	9.02 ± 0.80	3.818 ± 0.281	0.975 ± 0.002
Test	ECFP	Fingerprint	V	31.07 ± 0.96	11.443 ± 0.351	0.732 ± 0.011
Training	GCN	Fingerprint	V	4.32 ± 0.48	1.992 ± 0.174	0.997 ± 0.001
Test	GCN	Fingerprint	V	6.57 ± 1.54	2.587 ± 0.521	0.991 ± 0.004
Training	DMPNN	MaxMin	V	3.29 ± 0.17	1.421 ± 0.102	0.997 ± 0.000
Test	DMPNN	MaxMin	V	14.79 ± 0.19	6.312 ± 0.106	0.918 ± 0.002
Training	ECFP	MaxMin	V	8.81 ± 0.32	3.590 ± 0.069	0.975 ± 0.001
Test	ECFP	MaxMin	V	26.25 ± 0.33	11.829 ± 0.059	0.855 ± 0.003
Training	GCN	MaxMin	V	4.79 ± 1.02	2.100 ± 0.339	0.996 ± 0.001
Test	GCN	MaxMin	V	6.09 ± 0.79	3.074 ± 0.266	0.993 ± 0.002
Training	DMPNN	Scaffold	V	2.30 ± 0.25	1.022 ± 0.109	0.998 ± 0.000
Test	DMPNN	Scaffold	V	34.64 ± 0.32	11.082 ± 0.120	0.662 ± 0.007
Training	ECFP	Scaffold	V	7.82 ± 0.26	3.435 ± 0.092	0.976 ± 0.001
Test	ECFP	Scaffold	V	41.73 ± 0.62	13.360 ± 0.177	0.647 ± 0.013
Training	GCN	Scaffold	V	4.38 ± 0.77	2.025 ± 0.309	0.996 ± 0.002
Test	GCN	Scaffold	V	10.72 ± 3.19	3.419 ± 0.886	0.974 ± 0.018
Training	DMPNN	Stratified	V	3.33 ± 0.25	1.411 ± 0.105	0.997 ± 0.000
Test	DMPNN	Stratified	V	8.17 ± 0.24	3.267 ± 0.090	0.964 ± 0.001
Training	ECFP	Stratified	V	8.62 ± 0.38	3.547 ± 0.167	0.977 ± 0.002
Test	ECFP	Stratified	V	20.15 ± 0.32	8.712 ± 0.155	0.904 ± 0.001
Training	GCN	Stratified	V	4.93 ± 1.25	2.233 ± 0.537	0.996 ± 0.002
Test	GCN	Stratified	V	5.02 ± 1.25	2.241 ± 0.523	0.996 ± 0.002
Training	DMPNN	Fingerprint	$p(\sigma)$	0.0020 ± 0.00005		0.987 ± 0.001
Test	DMPNN	Fingerprint	$p(\sigma)$	0.0033 ± 0.00004		0.953 ± 0.001
Training	ECFP	Fingerprint	$p(\sigma)$	0.0047 ± 0.00002		0.928 ± 0.001
Test	ECFP	Fingerprint	$p(\sigma)$	0.0062 ± 0.00006		0.840 ± 0.003
Training	GCN	Fingerprint	$p(\sigma)$	0.0033 ± 0.00003		0.966 ± 0.001
Test	GCN	Fingerprint	$p(\sigma)$	0.0044 ± 0.00009		0.918 ± 0.004

Continued on next page

Continued from previous page

Split set	Model	Split method	Property	MAE	AAD%	R^2
Training	DMPNN	MaxMin	$p(\sigma)$	0.0021 ± 0.00005		0.986 ± 0.001
Test	DMPNN	MaxMin	$p(\sigma)$	0.0033 ± 0.00002		0.956 ± 0.001
Training	ECFP	MaxMin	$p(\sigma)$	0.0045 ± 0.00002		0.931 ± 0.001
Test	ECFP	MaxMin	$p(\sigma)$	0.0064 ± 0.00002		0.850 ± 0.001
Training	GCN	MaxMin	$p(\sigma)$	0.0033 ± 0.00003		0.966 ± 0.001
Test	GCN	MaxMin	$p(\sigma)$	0.0041 ± 0.00002		0.938 ± 0.001
Training	DMPNN	Scaffold	$p(\sigma)$	0.0021 ± 0.00003		0.986 ± 0.000
Test	DMPNN	Scaffold	$p(\sigma)$	0.0031 ± 0.00002		0.963 ± 0.001
Training	ECFP	Scaffold	$p(\sigma)$	0.0046 ± 0.00004		0.926 ± 0.001
Test	ECFP	Scaffold	$p(\sigma)$	0.0061 ± 0.00004		0.865 ± 0.001
Training	GCN	Scaffold	$p(\sigma)$	0.0034 ± 0.00006		0.964 ± 0.001
Test	GCN	Scaffold	$p(\sigma)$	0.0038 ± 0.00007		0.950 ± 0.001
Training	DMPNN	Stratified	$p(\sigma)$	0.0021 ± 0.00004		0.985 ± 0.001
Test	DMPNN	Stratified	$p(\sigma)$	0.0026 ± 0.00002		0.975 ± 0.000
Training	ECFP	Stratified	$p(\sigma)$	0.0047 ± 0.00002		0.924 ± 0.001
Test	ECFP	Stratified	$p(\sigma)$	0.0053 ± 0.00002		0.900 ± 0.001
Training	GCN	Stratified	$p(\sigma)$	0.0034 ± 0.00003		0.963 ± 0.001
Test	GCN	Stratified	$p(\sigma)$	0.0035 ± 0.00004		0.958 ± 0.001
Training	DMPNN	Fingerprint	$p^{\mathrm{nhb}}(\sigma)$	0.0020 ± 0.00003		0.985 ± 0.000
Test	DMPNN	Fingerprint	$p^{\mathrm{nhb}}(\sigma)$	0.0028 ± 0.00001		0.959 ± 0.000
Training	ECFP	Fingerprint	$p^{\mathrm{nhb}}(\sigma)$	0.0038 ± 0.00004		0.940 ± 0.001
Test	ECFP	Fingerprint	$p^{\mathrm{nhb}}(\sigma)$	0.0052 ± 0.00005		0.860 ± 0.002
Training	GCN	Fingerprint	$p^{\mathrm{nhb}}(\sigma)$	0.0027 ± 0.00003		0.972 ± 0.001
Test	GCN	Fingerprint	$p^{\mathrm{nhb}}(\sigma)$	0.0036 ± 0.00009		0.933 ± 0.002
Training	DMPNN	MaxMin	$p^{\mathrm{nhb}}(\sigma)$	0.0019 ± 0.00003		0.985 ± 0.000
Test	DMPNN	MaxMin	$p^{\mathrm{nhb}}(\sigma)$	0.0028 ± 0.00002		0.962 ± 0.001
Training	ECFP	MaxMin	$p^{\mathrm{nhb}}(\sigma)$	0.0037 ± 0.00002		0.943 ± 0.001
Test	ECFP	MaxMin	$p^{\mathrm{nhb}}(\sigma)$	0.0054 ± 0.00003		0.866 ± 0.001
Training	GCN	MaxMin	$p^{\mathrm{nhb}}(\sigma)$	0.0027 ± 0.00003		0.971 ± 0.001
Test	GCN	MaxMin	$p^{\mathrm{nhb}}(\sigma)$	0.0034 ± 0.00003		0.946 ± 0.001
Training	DMPNN	Scaffold	$p^{\mathrm{nhb}}(\sigma)$	0.0019 ± 0.00001		0.985 ± 0.000
Test	DMPNN	Scaffold	$p^{\mathrm{nhb}}(\sigma)$	0.0026 ± 0.00002		0.969 ± 0.001
Training	ECFP	Scaffold	$p^{\mathrm{nhb}}(\sigma)$	0.0038 ± 0.00002		0.940 ± 0.000
Test	ECFP	Scaffold	$p^{\mathrm{nhb}}(\sigma)$	0.0052 ± 0.00003		0.878 ± 0.001
Training	GCN	Scaffold	$p^{\mathrm{nhb}}(\sigma)$	0.0027 ± 0.00006		0.971 ± 0.001
Test	GCN	Scaffold	$p^{\mathrm{nhb}}(\sigma)$	0.0031 ± 0.00005		0.957 ± 0.001
Training	DMPNN	Stratified	$p^{\mathrm{nhb}}(\sigma)$	0.0020 ± 0.00003		0.984 ± 0.000
Test	DMPNN	Stratified	$p^{\mathrm{nhb}}(\sigma)$	0.0022 ± 0.00002		0.978 ± 0.000
Training	ECFP	Stratified	$p^{\mathrm{nhb}}(\sigma)$	0.0039 ± 0.00003		0.937 ± 0.001
Test	ECFP	Stratified	$p^{\mathrm{nhb}}(\sigma)$	0.0044 ± 0.00002		0.912 ± 0.001
Training	GCN	Stratified	$p^{\mathrm{nhb}}(\sigma)$	0.0028 ± 0.00003		0.970 ± 0.001
Test	GCN	Stratified	$p^{\mathrm{nhb}}(\sigma)$	0.0029 ± 0.00003		0.965 ± 0.001
Training	DMPNN	Fingerprint	$p^{\mathrm{oh}}(\sigma)$	0.0002 ± 0.00003		0.897 ± 0.027
Test	DMPNN	Fingerprint	$p^{\mathrm{oh}}(\sigma)$	0.0002 ± 0.00002		0.847 ± 0.025
Training	ECFP	Fingerprint	$p^{\mathrm{oh}}(\sigma)$	0.0003 ± 0.00000		0.820 ± 0.002
Test	ECFP	Fingerprint	$p^{\mathrm{oh}}(\sigma)$	0.0004 ± 0.00001		0.569 ± 0.026
Training	GCN	Fingerprint	$p^{\mathrm{oh}}(\sigma)$	0.0002 ± 0.00001		0.877 ± 0.003
Test	GCN	Fingerprint	$p^{\mathrm{oh}}(\sigma)$	0.0003 ± 0.00001		0.830 ± 0.004
Training	DMPNN	MaxMin	$p^{\mathrm{oh}}(\sigma)$	0.0002 ± 0.00000		0.915 ± 0.002
Test	DMPNN	MaxMin	$p^{\mathrm{oh}}(\sigma)$	0.0002 ± 0.00000		0.854 ± 0.003
Training	ECFP	MaxMin	$p^{\mathrm{oh}}(\sigma)$	0.0003 ± 0.00000		0.830 ± 0.002
Test	ECFP	MaxMin	$p^{\mathrm{oh}}(\sigma)$	0.0003 ± 0.00001		0.591 ± 0.014
Training	GCN	MaxMin	$p^{\mathrm{oh}}(\sigma)$	0.0002 ± 0.00001		0.879 ± 0.005
Test	GCN	MaxMin	$p^{\mathrm{oh}}(\sigma)$	0.0002 ± 0.00001		0.823 ± 0.008
Training	DMPNN	Scaffold	$p^{\mathrm{oh}}(\sigma)$	0.0002 ± 0.00002		0.891 ± 0.025
Test	DMPNN	Scaffold	$p^{\mathrm{oh}}(\sigma)$	0.0002 ± 0.00001		0.846 ± 0.013
Training	ECFP	Scaffold	$p^{\mathrm{oh}}(\sigma)$	0.0003 ± 0.00000		0.829 ± 0.002

Continued on next page

Continued from previous page

Split set	Model	Split method	Property	MAE	AAD%	R^2
Test	ECFP	Scaffold	$p^{oh}(\sigma)$	0.0004 ± 0.00001		0.451 ± 0.016
Training	GCN	Scaffold	$p^{oh}(\sigma)$	0.0002 ± 0.00000		0.878 ± 0.002
Test	GCN	Scaffold	$p^{oh}(\sigma)$	0.0002 ± 0.00000		0.826 ± 0.003
Training	DMPNN	Stratified	$p^{oh}(\sigma)$	0.0002 ± 0.00000		0.911 ± 0.005
Test	DMPNN	Stratified	$p^{oh}(\sigma)$	0.0002 ± 0.00000		0.882 ± 0.002
Training	ECFP	Stratified	$p^{oh}(\sigma)$	0.0003 ± 0.00001		0.812 ± 0.007
Test	ECFP	Stratified	$p^{oh}(\sigma)$	0.0003 ± 0.00001		0.696 ± 0.007
Training	GCN	Stratified	$p^{oh}(\sigma)$	0.0002 ± 0.00000		0.877 ± 0.002
Test	GCN	Stratified	$p^{oh}(\sigma)$	0.0002 ± 0.00000		0.857 ± 0.002
Training	DMPNN	Fingerprint	$p^{ot}(\sigma)$	0.0002 ± 0.00001		0.957 ± 0.004
Test	DMPNN	Fingerprint	$p^{ot}(\sigma)$	0.0004 ± 0.00001		0.826 ± 0.004
Training	ECFP	Fingerprint	$p^{ot}(\sigma)$	0.0006 ± 0.00001		0.769 ± 0.006
Test	ECFP	Fingerprint	$p^{ot}(\sigma)$	0.0008 ± 0.00002		0.471 ± 0.008
Training	GCN	Fingerprint	$p^{ot}(\sigma)$	0.0004 ± 0.00001		0.896 ± 0.002
Test	GCN	Fingerprint	$p^{ot}(\sigma)$	0.0006 ± 0.00002		0.749 ± 0.011
Training	DMPNN	MaxMin	$p^{ot}(\sigma)$	0.0002 ± 0.00000		0.958 ± 0.001
Test	DMPNN	MaxMin	$p^{ot}(\sigma)$	0.0004 ± 0.00000		0.850 ± 0.001
Training	ECFP	MaxMin	$p^{ot}(\sigma)$	0.0005 ± 0.00000		0.781 ± 0.003
Test	ECFP	MaxMin	$p^{ot}(\sigma)$	0.0009 ± 0.00000		0.551 ± 0.002
Training	GCN	MaxMin	$p^{ot}(\sigma)$	0.0004 ± 0.00001		0.894 ± 0.003
Test	GCN	MaxMin	$p^{ot}(\sigma)$	0.0006 ± 0.00001		0.790 ± 0.004
Training	DMPNN	Scaffold	$p^{ot}(\sigma)$	0.0003 ± 0.00002		0.953 ± 0.011
Test	DMPNN	Scaffold	$p^{ot}(\sigma)$	0.0004 ± 0.00001		0.853 ± 0.002
Training	ECFP	Scaffold	$p^{ot}(\sigma)$	0.0006 ± 0.00001		0.770 ± 0.006
Test	ECFP	Scaffold	$p^{ot}(\sigma)$	0.0008 ± 0.00000		0.538 ± 0.002
Training	GCN	Scaffold	$p^{ot}(\sigma)$	0.0004 ± 0.00000		0.891 ± 0.002
Test	GCN	Scaffold	$p^{ot}(\sigma)$	0.0006 ± 0.00000		0.793 ± 0.005
Training	DMPNN	Stratified	$p^{ot}(\sigma)$	0.0003 ± 0.00004		0.943 ± 0.015
Test	DMPNN	Stratified	$p^{ot}(\sigma)$	0.0003 ± 0.00002		0.902 ± 0.007
Training	ECFP	Stratified	$p^{ot}(\sigma)$	0.0006 ± 0.00001		0.755 ± 0.007
Test	ECFP	Stratified	$p^{ot}(\sigma)$	0.0007 ± 0.00000		0.660 ± 0.004
Training	GCN	Stratified	$p^{ot}(\sigma)$	0.0004 ± 0.00001		0.883 ± 0.004
Test	GCN	Stratified	$p^{ot}(\sigma)$	0.0005 ± 0.00001		0.851 ± 0.003

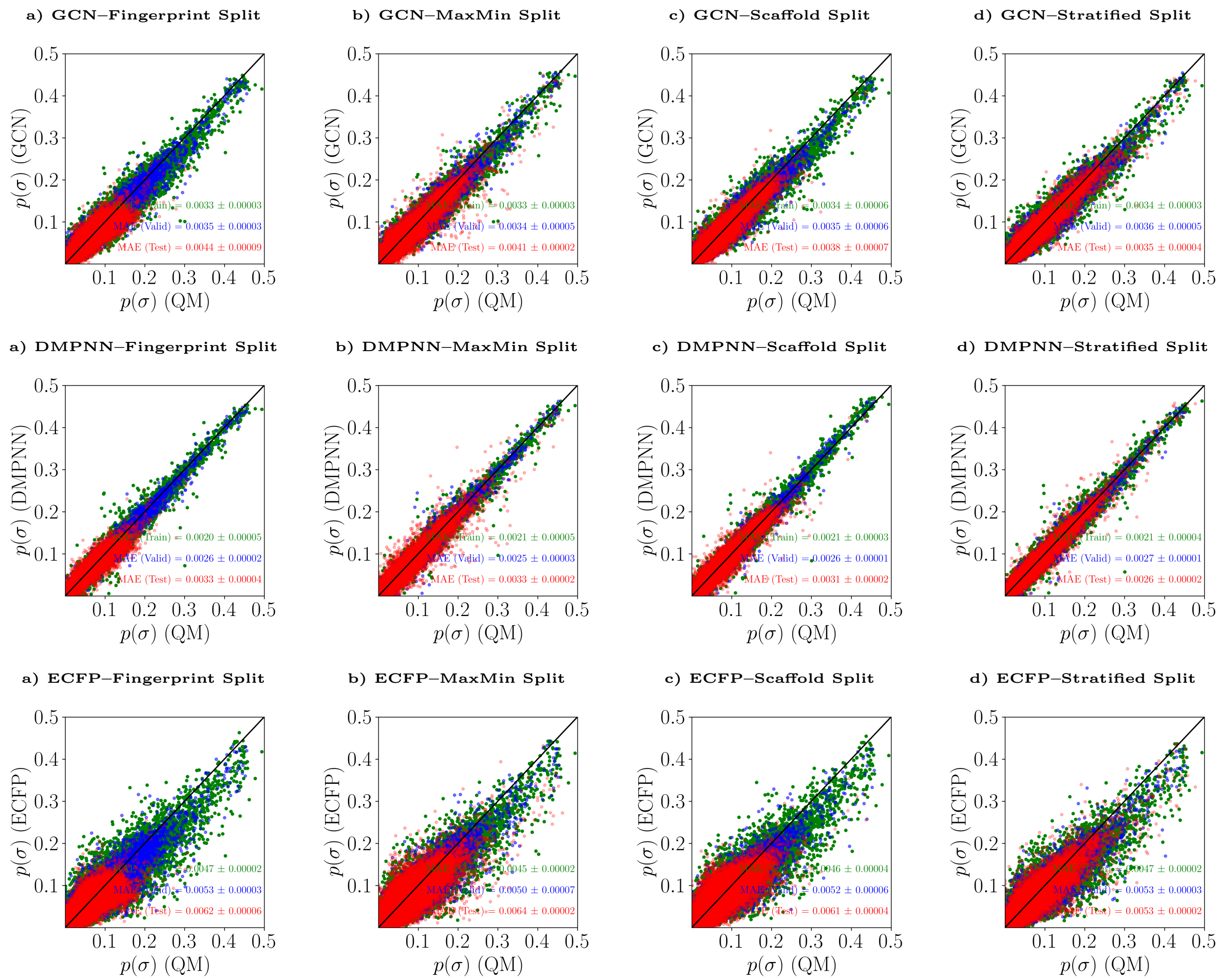

Figure S4: Parity plots for predictions of $p(\sigma)$ across three machine learning models (GCN, DMPNN, ECFP-MLP) and four train-test splitting strategies (Fingerprint, MaxMin, Scaffold, and Stratified). Each subplot compares model predictions to QM COSMO reference values across all data sets: green points indicate training set, blue points validation set, and red points test set.

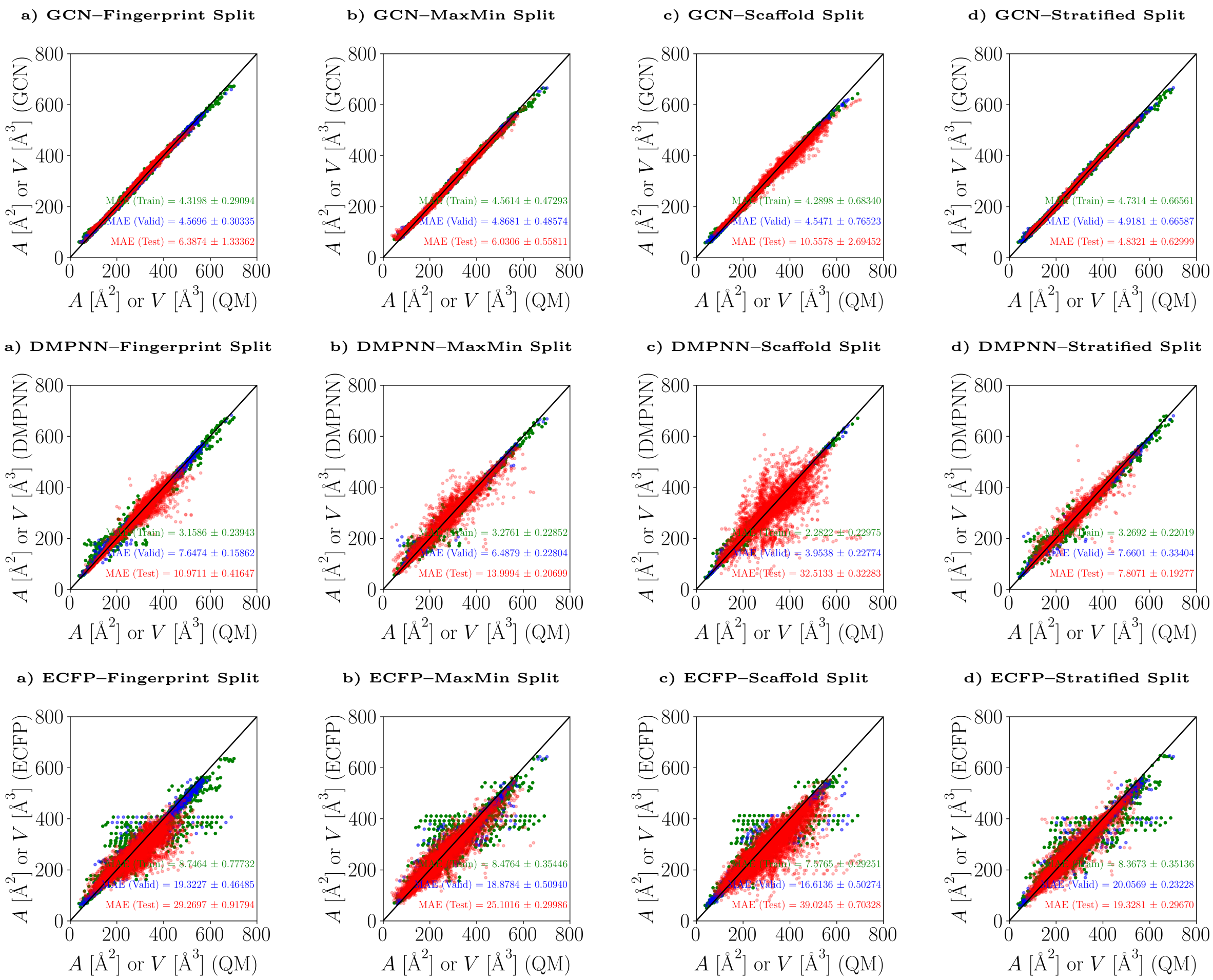

Figure S5: Parity plots for predictions of V and A across three machine learning models (GCN, DMPNN, ECFP-MLP) and four train-test splitting strategies (Fingerprint, MaxMin, Scaffold, and Stratified). Each subplot compares model predictions to QM COSMO reference values across all data sets: green points indicate training set, blue points validation set, and red points test set.

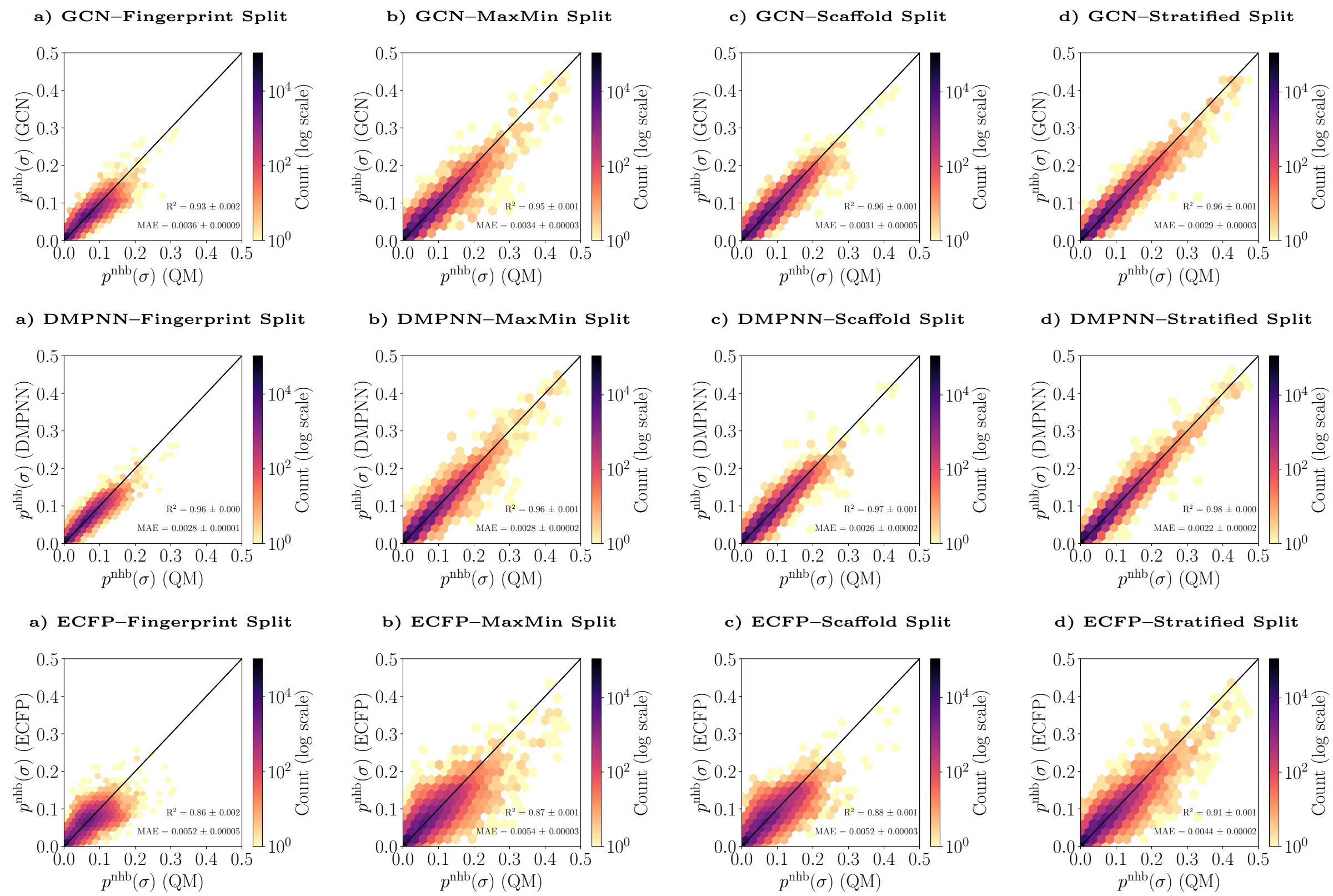

Figure S6: Parity plots for predictions of $p^{\mathrm{nhb}}(\sigma)$ across three machine learning models (GCN, DMPNN, ECFP-MLP) and four train-test splitting strategies (Fingerprint, MaxMin, Scaffold, and Stratified). Each subplot compares model predictions to QM COSMO reference values for the test set. Hexagonal bins indicate the density of predictions, with color intensity representing the number of data points (see color bar).

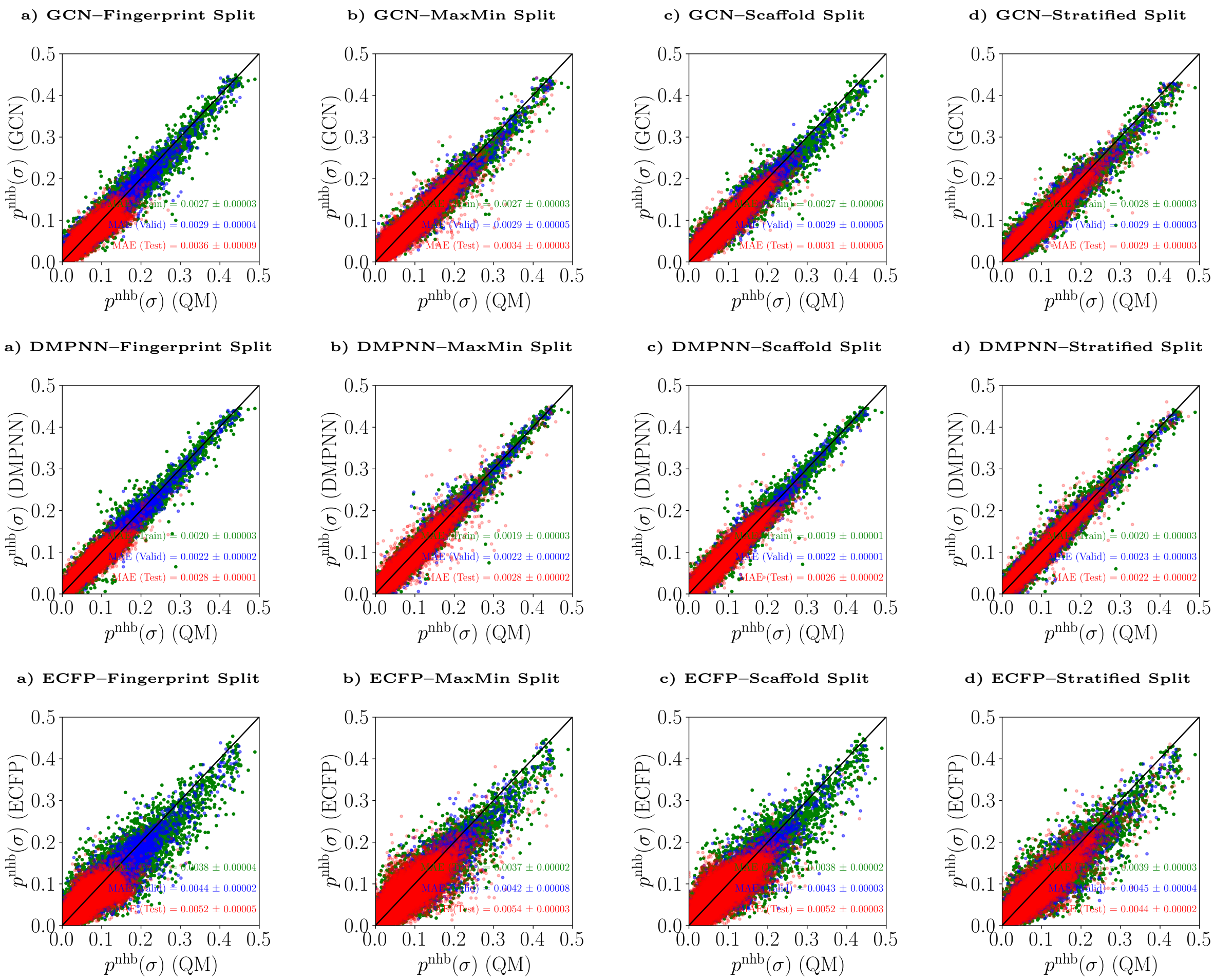

Figure S7: Parity plots for predictions of $p^{\mathrm{nhb}}(\sigma)$ across three machine learning models (GCN, DMPNN, ECFP-MLP) and four train-test splitting strategies (Fingerprint, MaxMin, Scaffold, and Stratified). Each subplot compares model predictions to QM COSMO reference values across all data sets: green points indicate training set, blue points validation set, and red points test set.

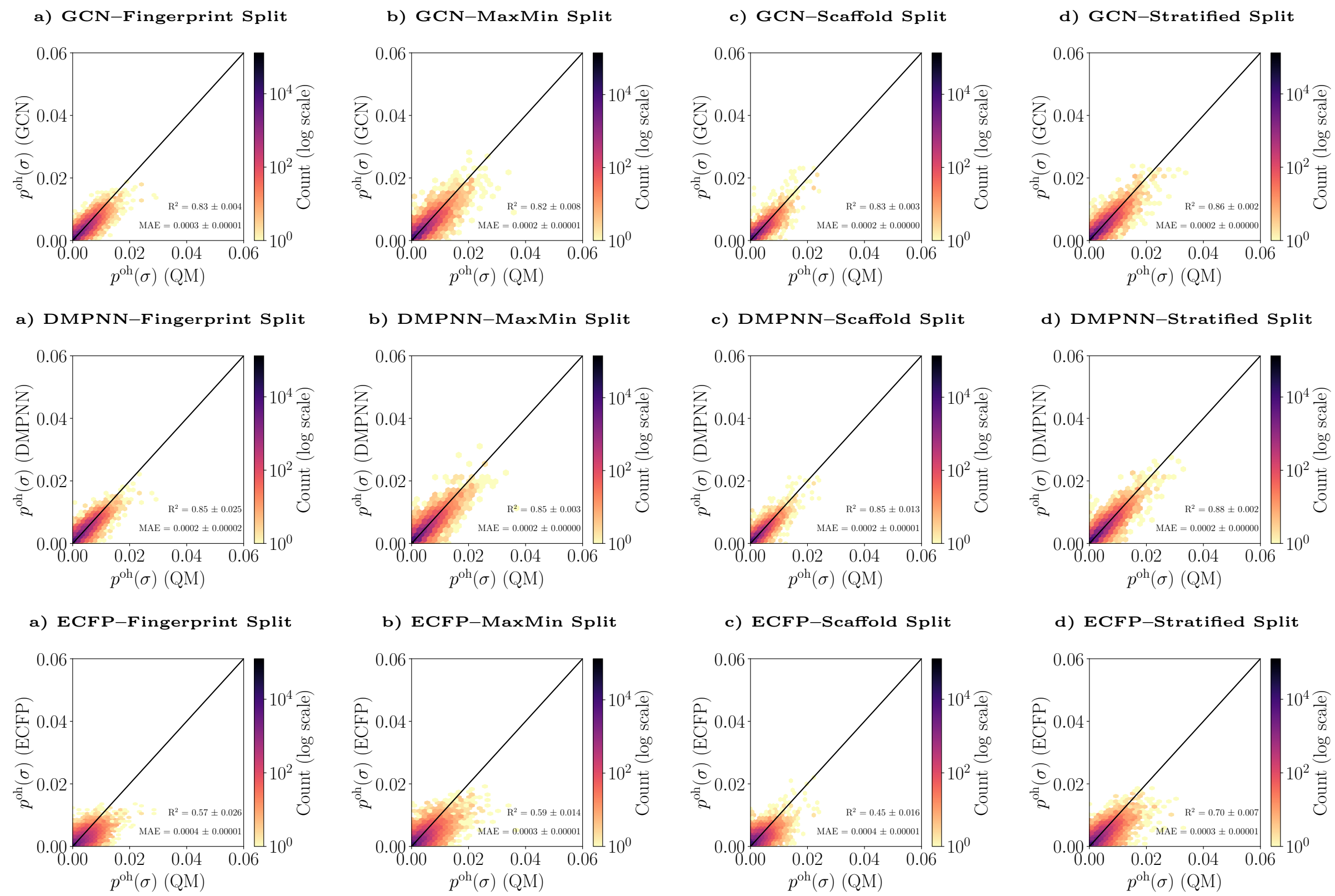

Figure S8: Parity plots for predictions of $p^{\text{oh}}(\sigma)$ across three machine learning models (GCN, DMPNN, ECFP-MLP) and four train-test splitting strategies (Fingerprint, MaxMin, Scaffold, and Stratified). Each subplot compares model predictions to QM COSMO reference values for the test set. Hexagonal bins indicate the density of predictions, with color intensity representing the number of data points (see color bar).

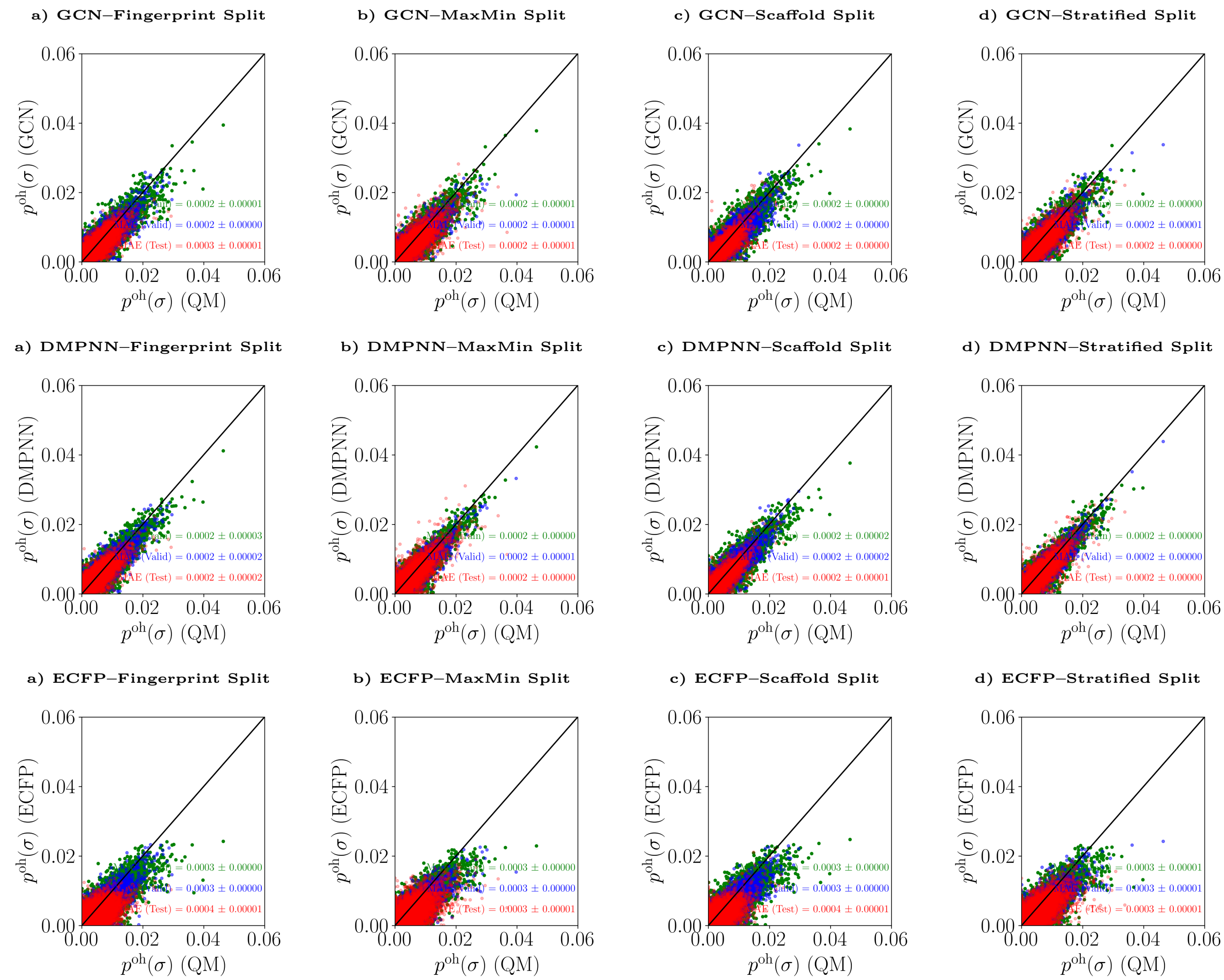

Figure S9: Parity plots for predictions of $p^{\mathrm{oh}}(\sigma)$ across three machine learning models (GCN, DMPNN, ECFP-MLP) and four train-test splitting strategies (Fingerprint, MaxMin, Scaffold, and Stratified). Each subplot compares model predictions to QM COSMO reference values across all data sets: green points indicate training set, blue points validation set, and red points test set.

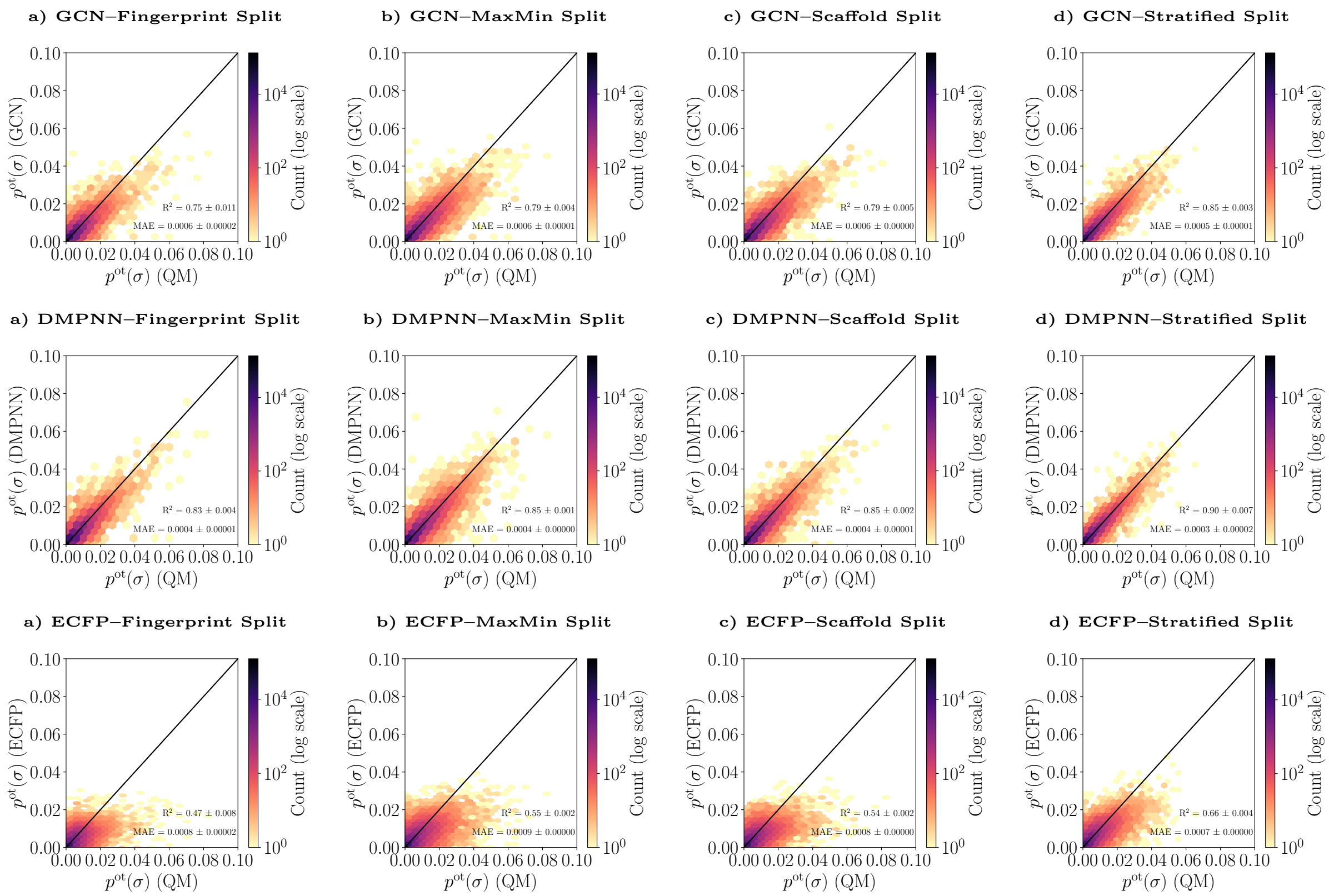

Figure S10: Parity plots for predictions of $p^{ot}(\sigma)$ across three machine learning models (GCN, DMPNN, ECFP-MLP) and four train-test splitting strategies (Fingerprint, MaxMin, Scaffold, and Stratified). Each subplot compares model predictions to QM COSMO reference values for the test set. Hexagonal bins indicate the density of predictions, with color intensity representing the number of data points (see color bar).

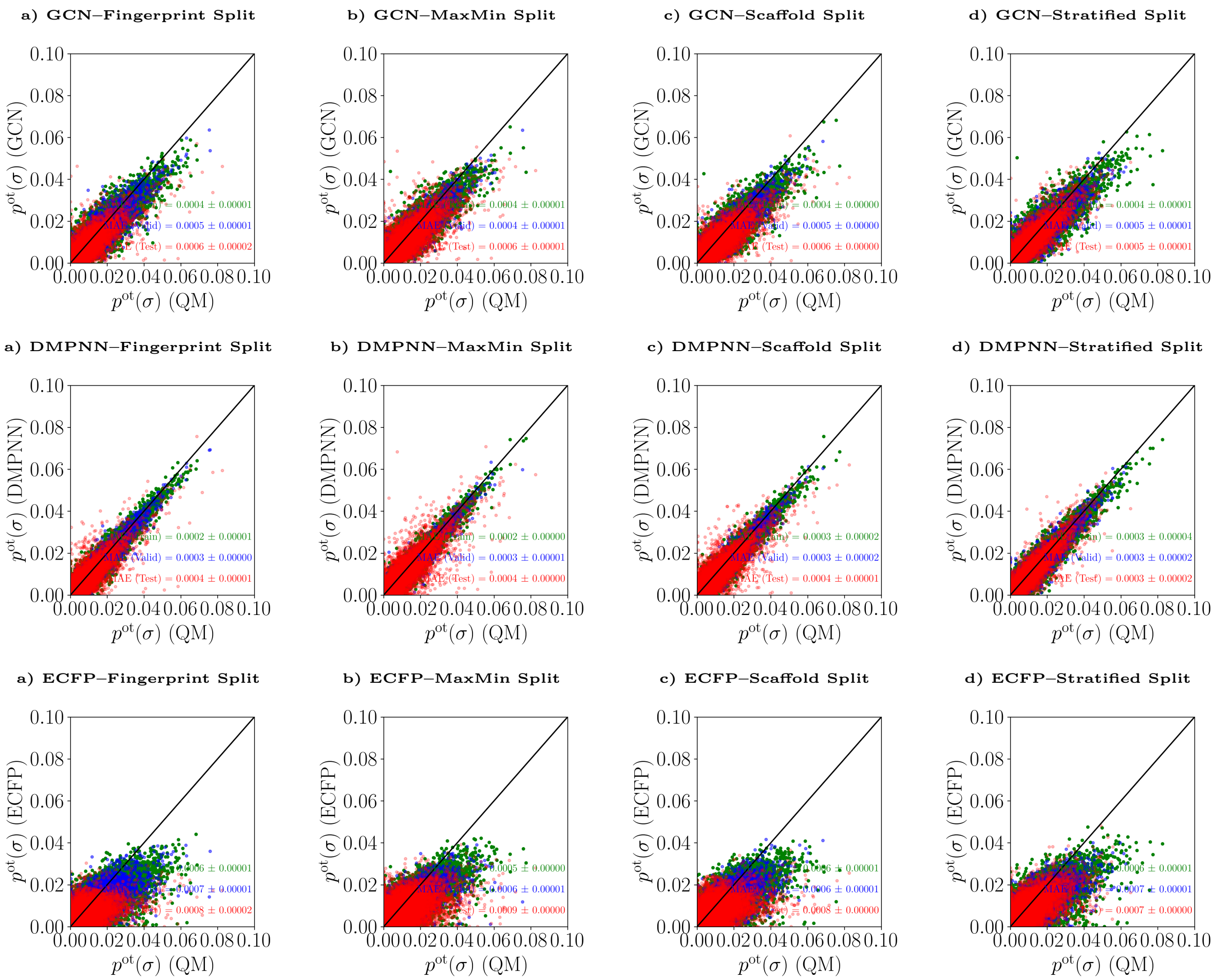

Figure S11: Parity plots for predictions of $p^{\mathrm{ot}}(\sigma)$ across three machine learning models (GCN, DMPNN, ECFP-MLP) and four train-test splitting strategies (Fingerprint, MaxMin, Scaffold, and Stratified). Each subplot compares model predictions to QM COSMO reference values across all data sets: green points indicate training set, blue points validation set, and red points test set.

4 Detailed evaluation of model accuracy

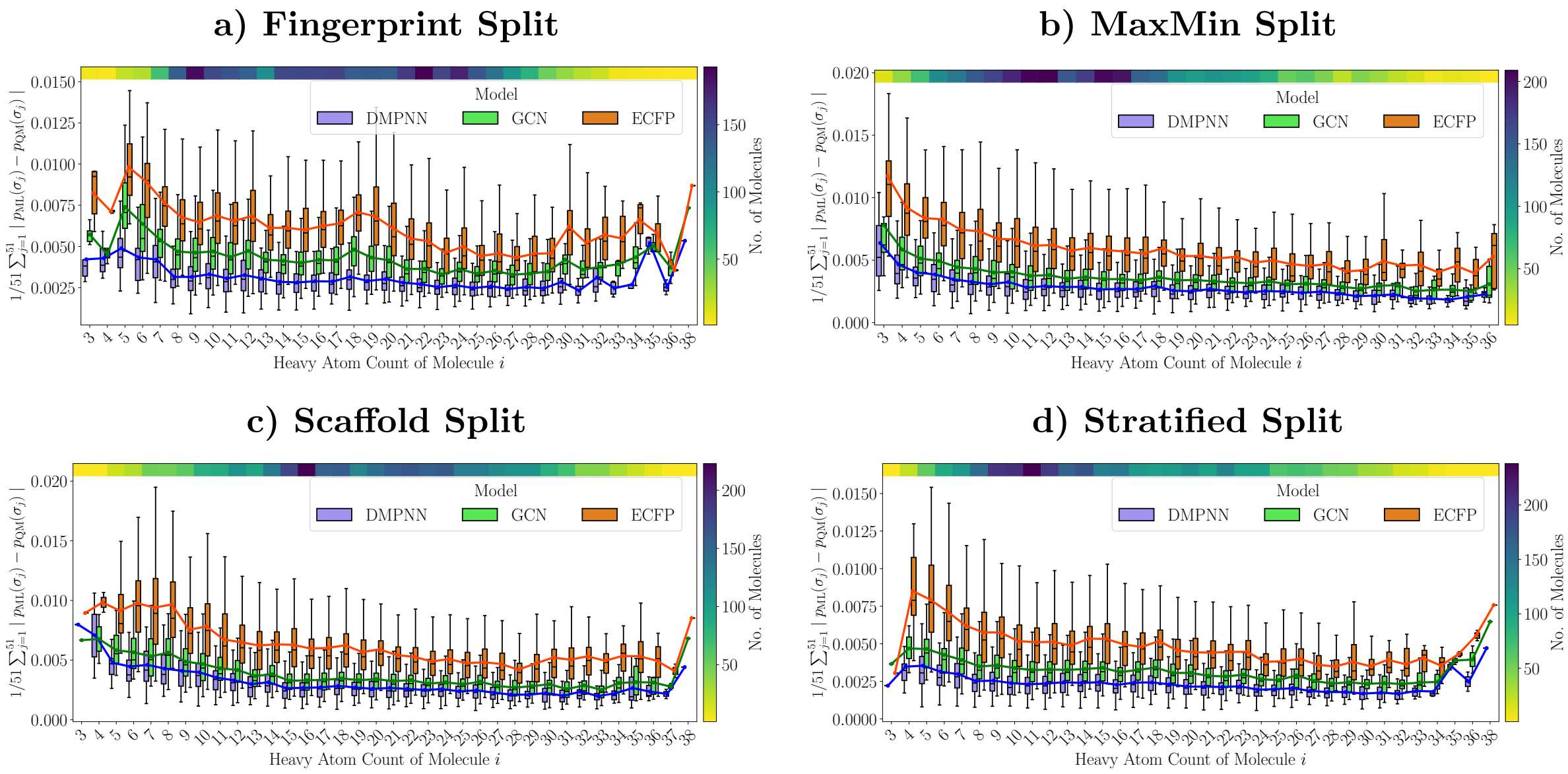

Figure S12: Prediction error distributions for $p(\sigma)$ from GCN, DMPNN, and ECFP-MLP models as a function of molecular heavy atom count across four train-test splitting strategies: **a)** Fingerprint, **b)** MaxMin, **c)** Scaffold, and **d)** Stratified. Only test set predictions are shown. Each box plot displays the median error (black line) and mean error (colored points: green for GCN, blue for DMPNN, orange for ECFP-MLP). The heatmap above each subplot indicates the number of test compounds in each heavy atom count bin.

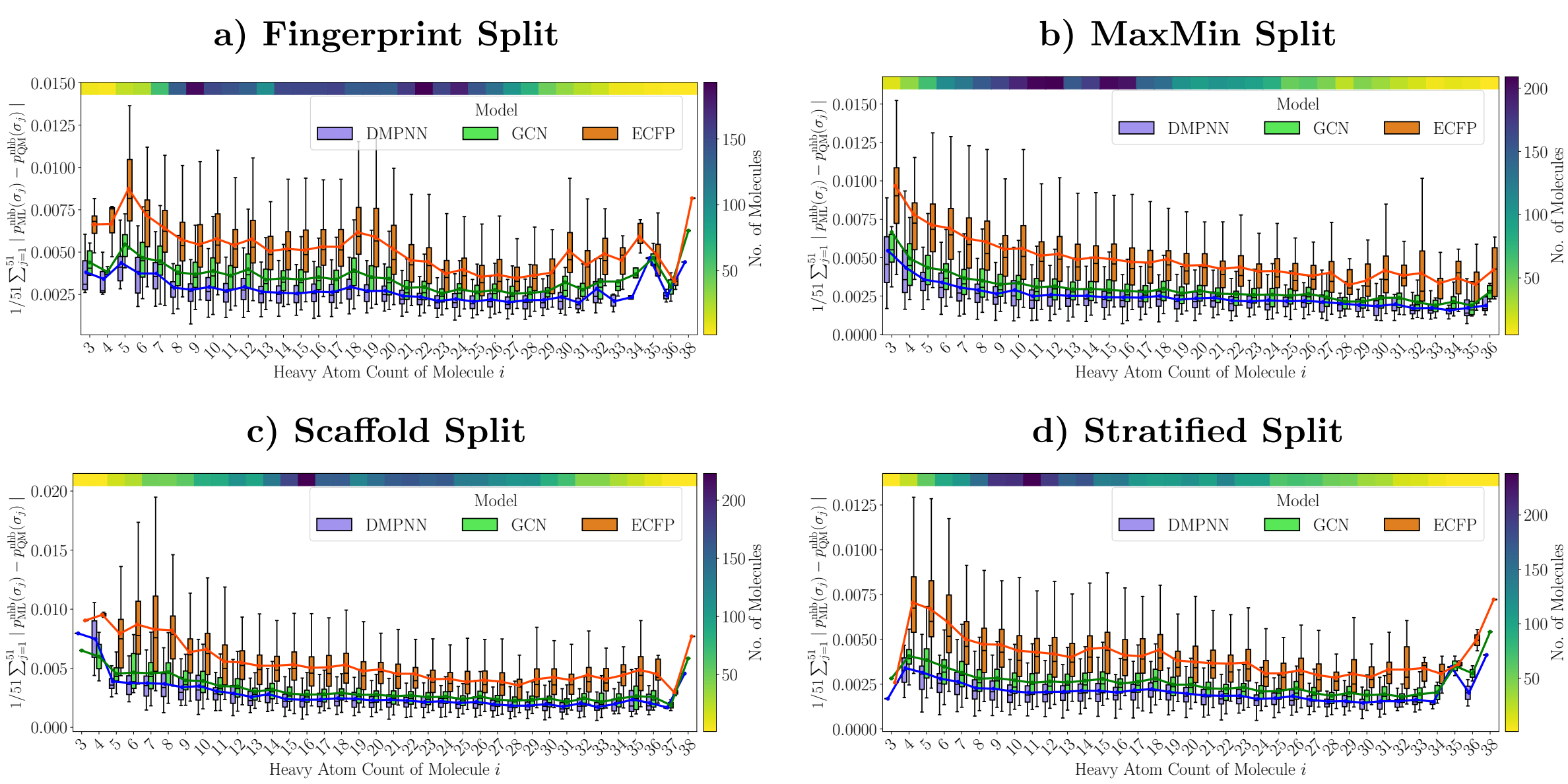

Figure S13: Prediction error distributions for $p^{\mathrm{nhb}}(\sigma)$ from GCN, DMPNN, and ECFP-MLP models as a function of molecular heavy atom count across four train-test splitting strategies: **a)** Fingerprint, **b)** MaxMin, **c)** Scaffold, and **d)** Stratified. Only test set predictions are shown. Each box plot displays the median error (black line) and mean error (colored points: green for GCN, blue for DMPNN, orange for ECFP-MLP). The heatmap above each subplot indicates the number of test compounds in each heavy atom count bin.

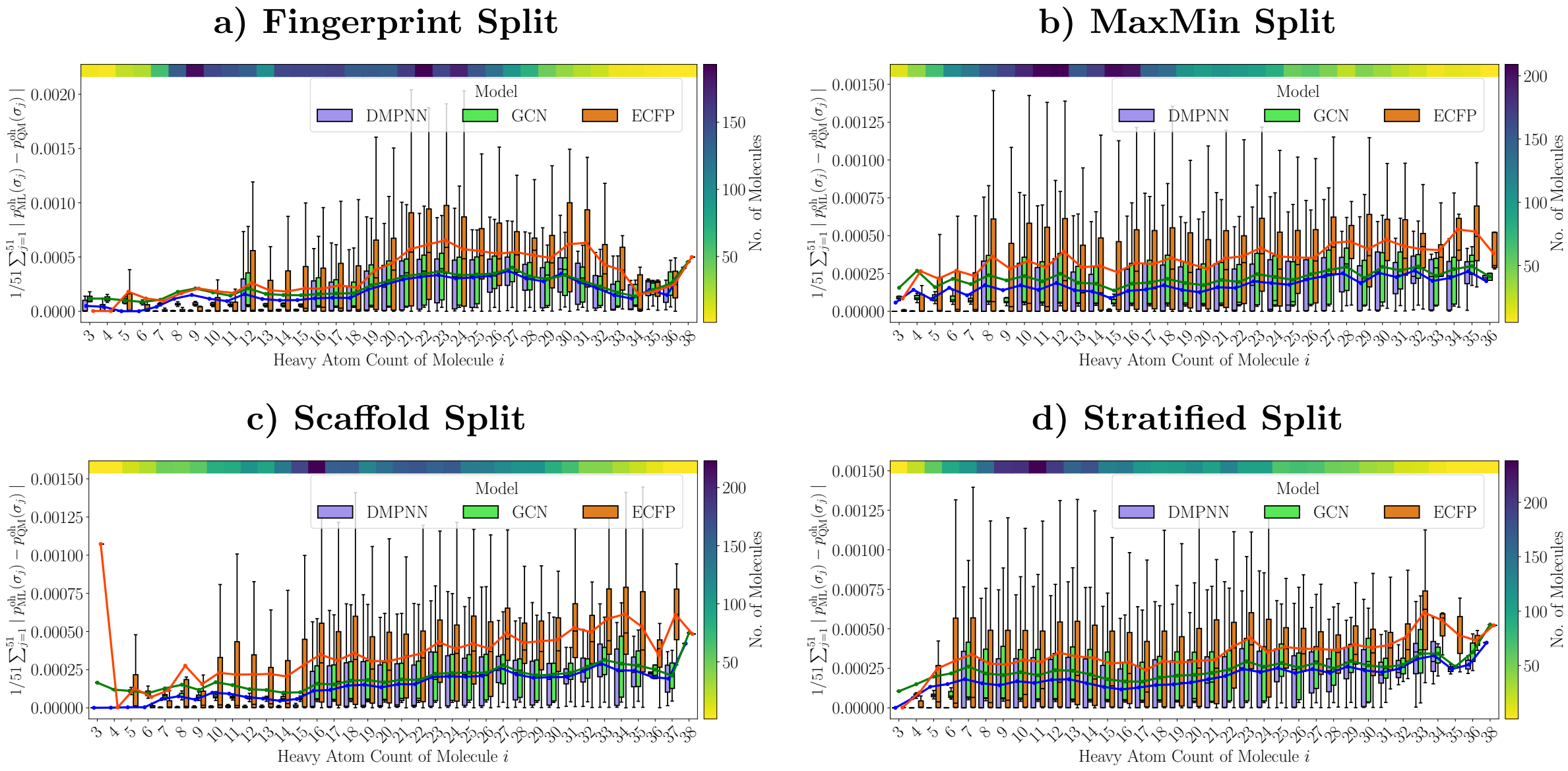

Figure S14: Prediction error distributions for $p^{\mathrm{oh}}(\sigma)$ from GCN, DMPNN, and ECFP-MLP models as a function of molecular heavy atom count across four train-test splitting strategies: **a)** Fingerprint, **b)** MaxMin, **c)** Scaffold, and **d)** Stratified. Only test set predictions are shown. Each box plot displays the median error (black line) and mean error (colored points: green for GCN, blue for DMPNN, orange for ECFP-MLP). The heatmap above each subplot indicates the number of test compounds in each heavy atom count bin.

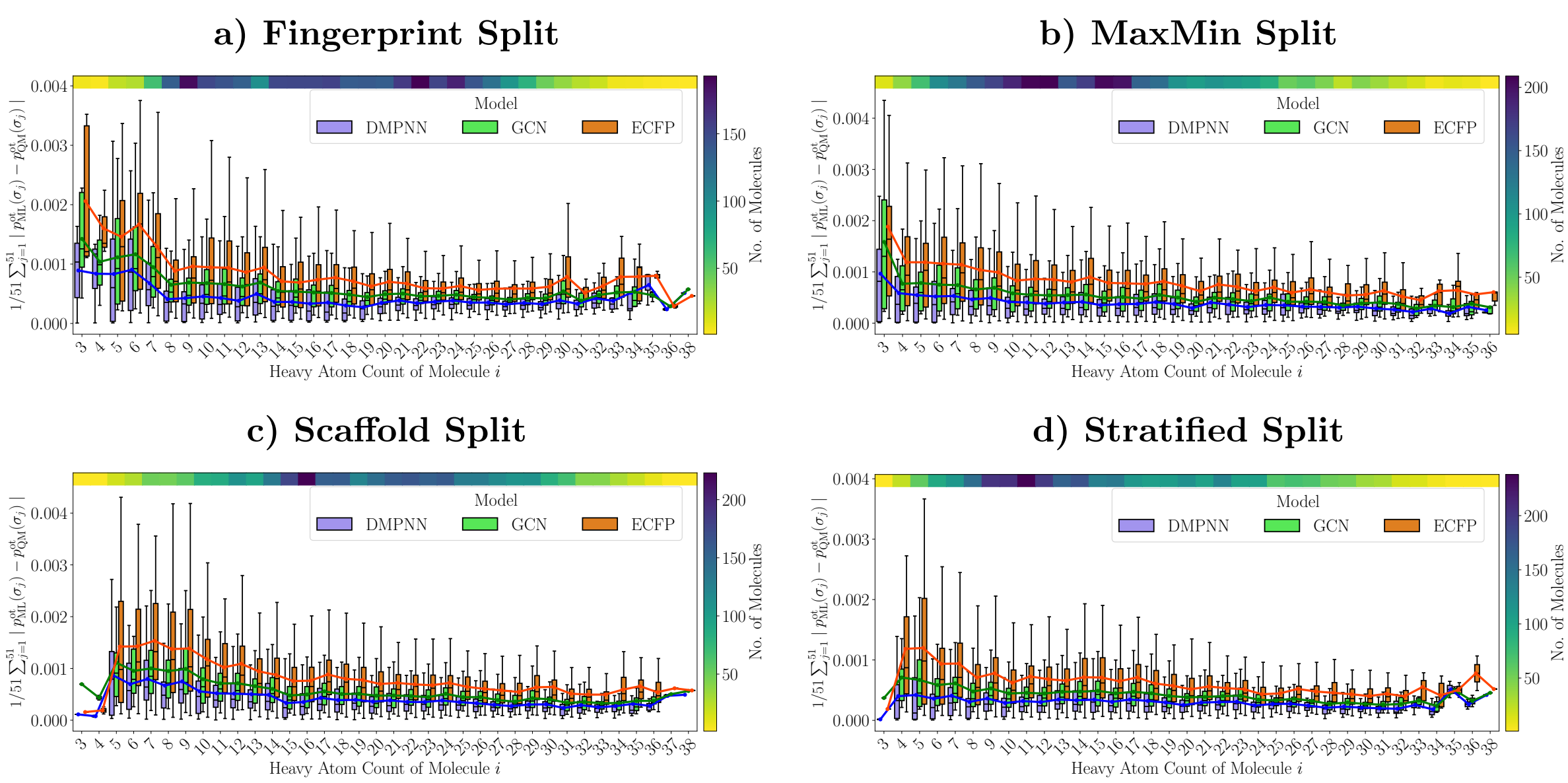

Figure S15: Prediction error distributions for $p^{\mathrm{ot}}(\sigma)$ from GCN, DMPNN, and ECFP-MLP models as a function of molecular heavy atom count across four train-test splitting strategies: **a)** Fingerprint, **b)** MaxMin, **c)** Scaffold, and **d)** Stratified. Only test set predictions are shown. Each box plot displays the median error (black line) and mean error (colored points: green for GCN, blue for DMPNN, orange for ECFP-MLP). The heatmap above each subplot indicates the number of test compounds in each heavy atom count bin.

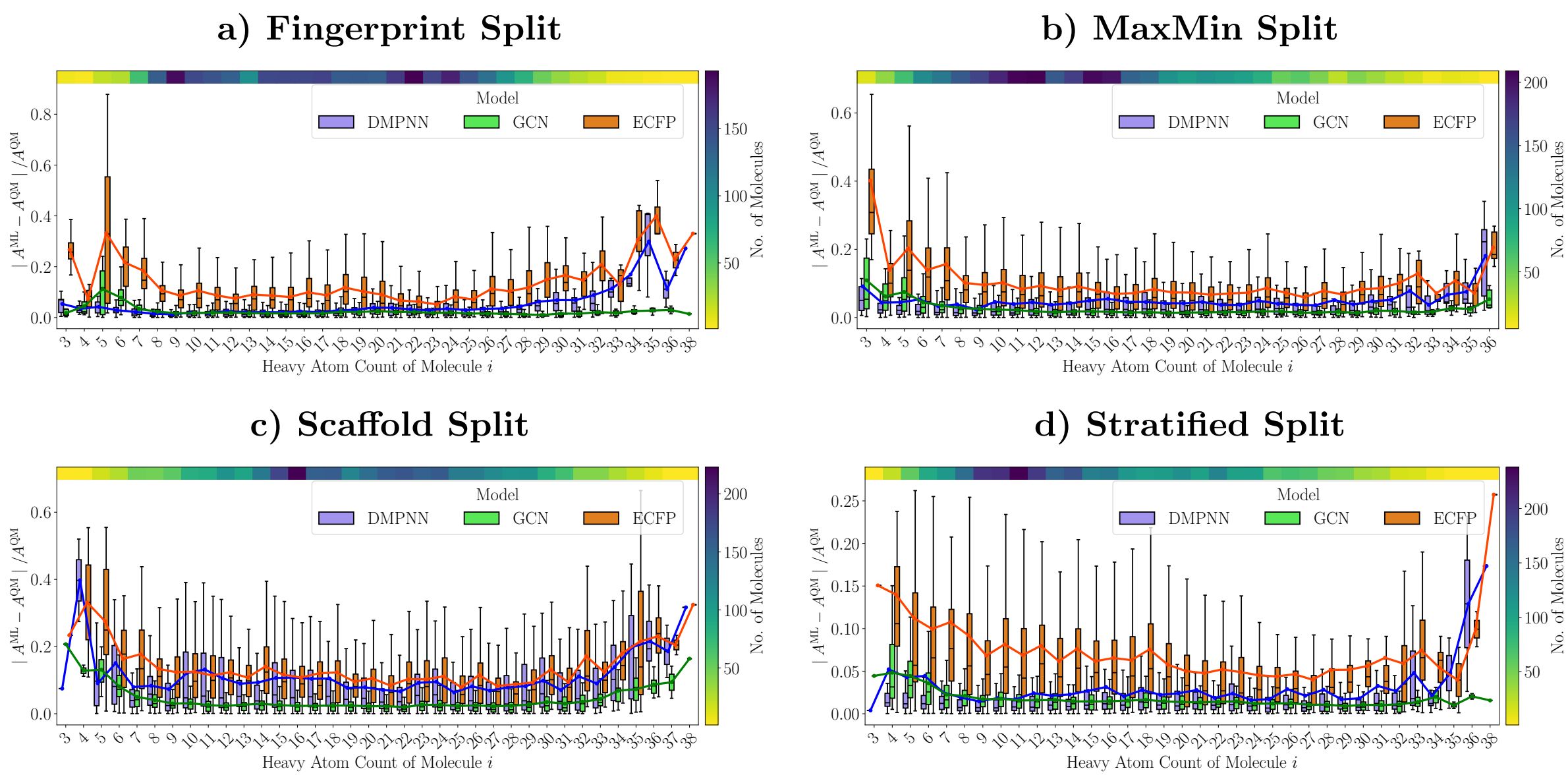

Figure S16: Prediction error distributions for A from GCN, DMPNN, and ECFP-MLP models as a function of molecular heavy atom count across four train-test splitting strategies: **a)** Fingerprint, **b)** MaxMin, **c)** Scaffold, and **d)** Stratified. Only test set predictions are shown. Each box plot displays the median error (black line) and mean error (colored points: green for GCN, blue for DMPNN, orange for ECFP-MLP). The heatmap above each subplot indicates the number of test compounds in each heavy atom count bin.

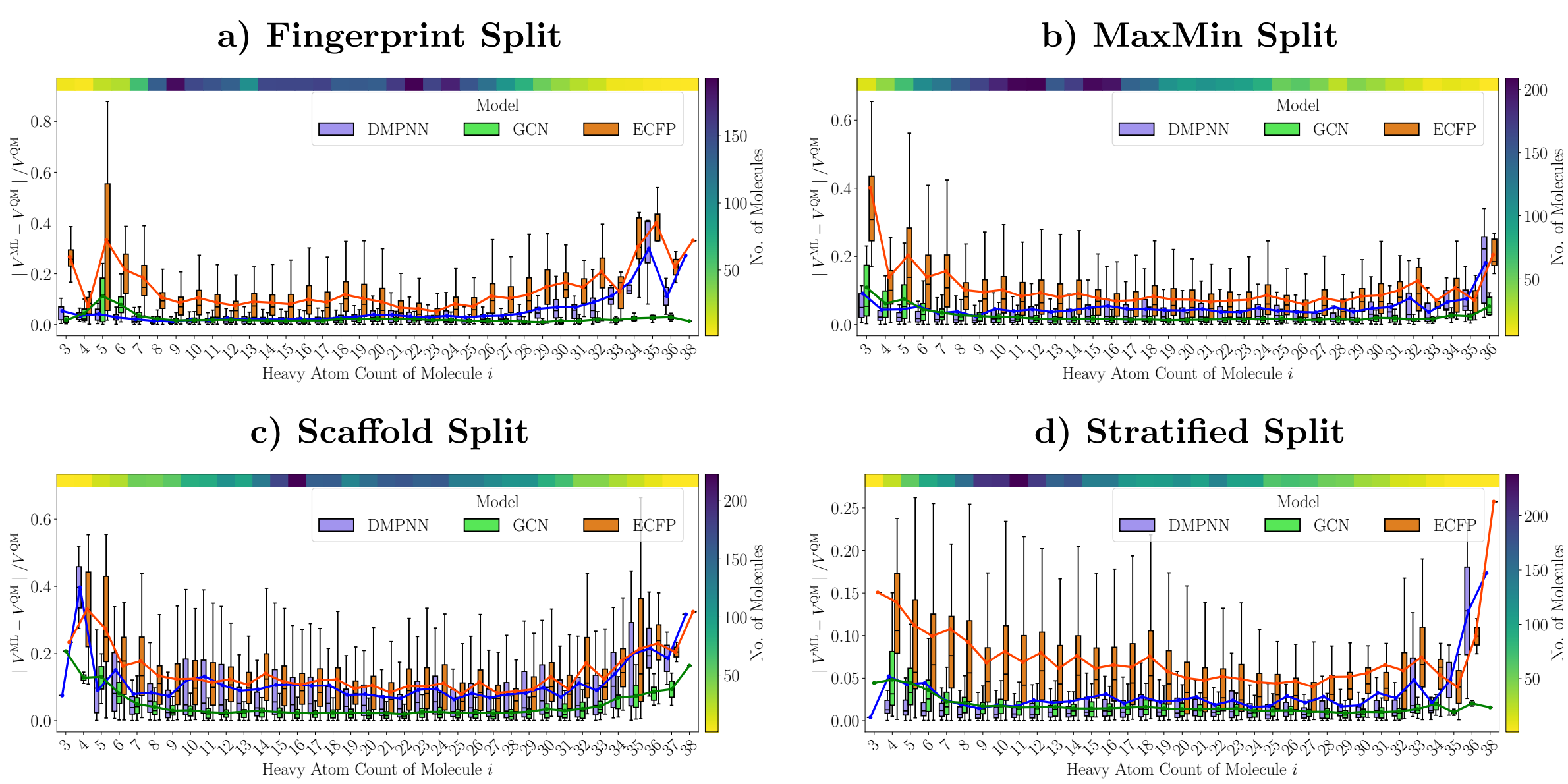

Figure S17: Prediction error distributions for V from GCN, DMPNN, and ECFP-MLP models as a function of molecular heavy atom count across four train-test splitting strategies: **a)** Fingerprint, **b)** MaxMin, **c)** Scaffold, and **d)** Stratified. Only test set predictions are shown. Each box plot displays the median error (black line) and mean error (colored points: green for GCN, blue for DMPNN, orange for ECFP-MLP). The heatmap above each subplot indicates the number of test compounds in each heavy atom count bin.

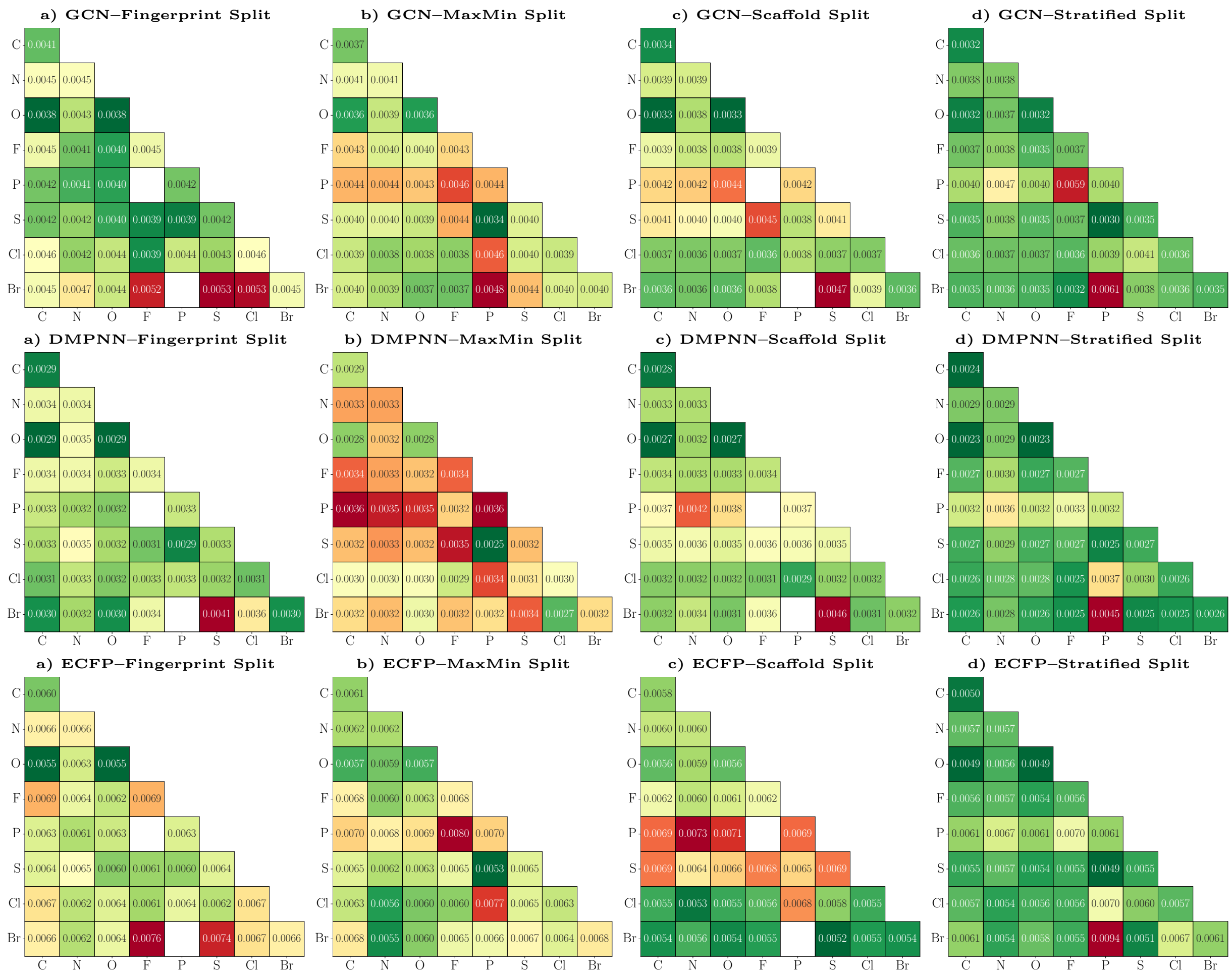

Figure S18: Prediction-error heatmaps for $p(\sigma)$ generated by the GCN, DMPNN, and ECFP-MLP models under four train–test splitting strategies: Fingerprint, MaxMin, Scaffold, and Stratified. Rows correspond to model type, while columns correspond to the splitting strategy. The heatmaps illustrate the spatial distribution of error across the chemical space, enabling comparison of how model performance and generalization depend on both architecture and data partitioning.

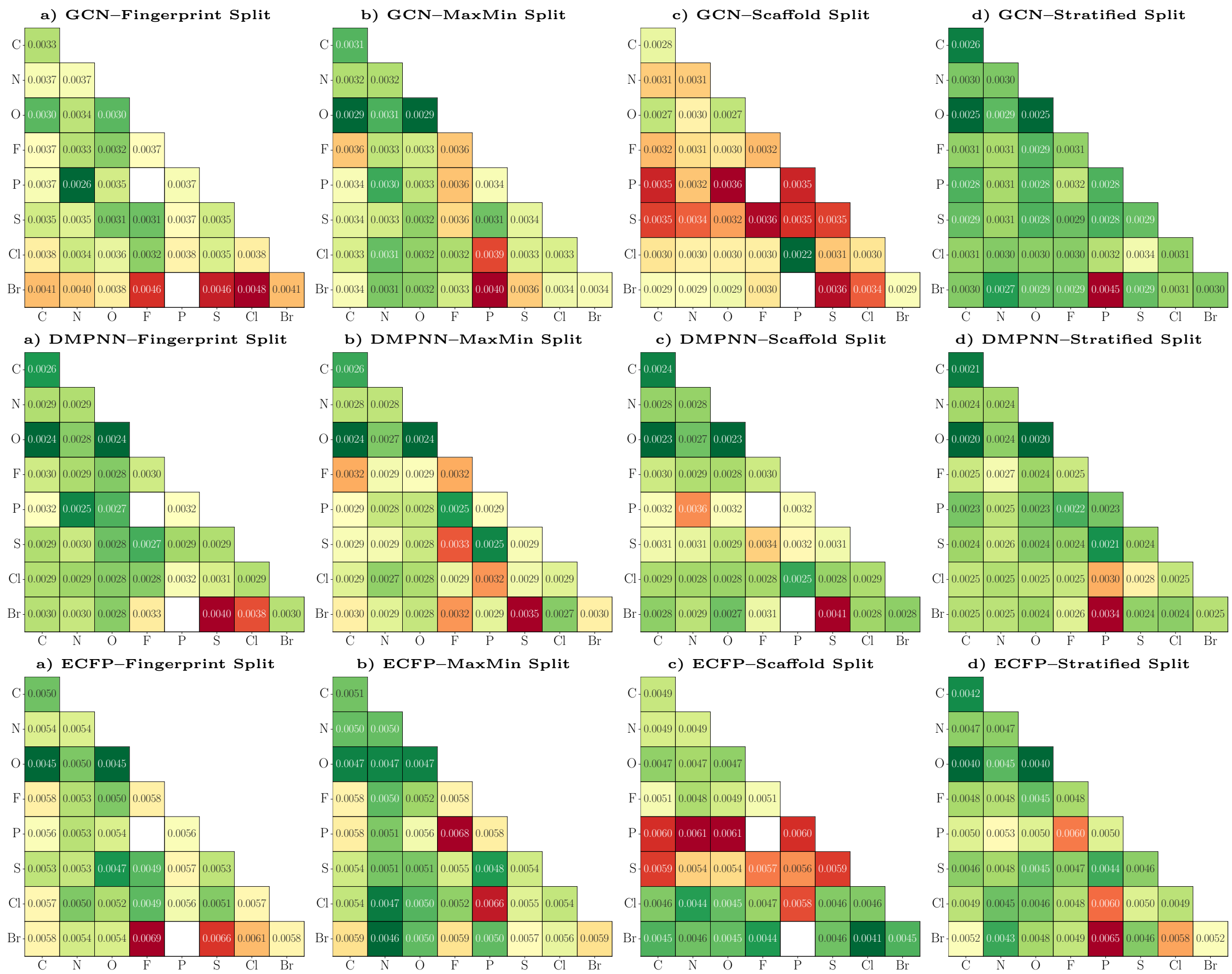

Figure S19: Prediction-error heatmaps for $p^{\mathrm{nhb}}(\sigma)$ generated by the GCN, DMPNN, and ECFP-MLP models under four train–test splitting strategies: Fingerprint, MaxMin, Scaffold, and Stratified. Rows correspond to model type, while columns correspond to the splitting strategy. The heatmaps illustrate the spatial distribution of error across the chemical space, enabling comparison of how model performance and generalization depend on both architecture and data partitioning.

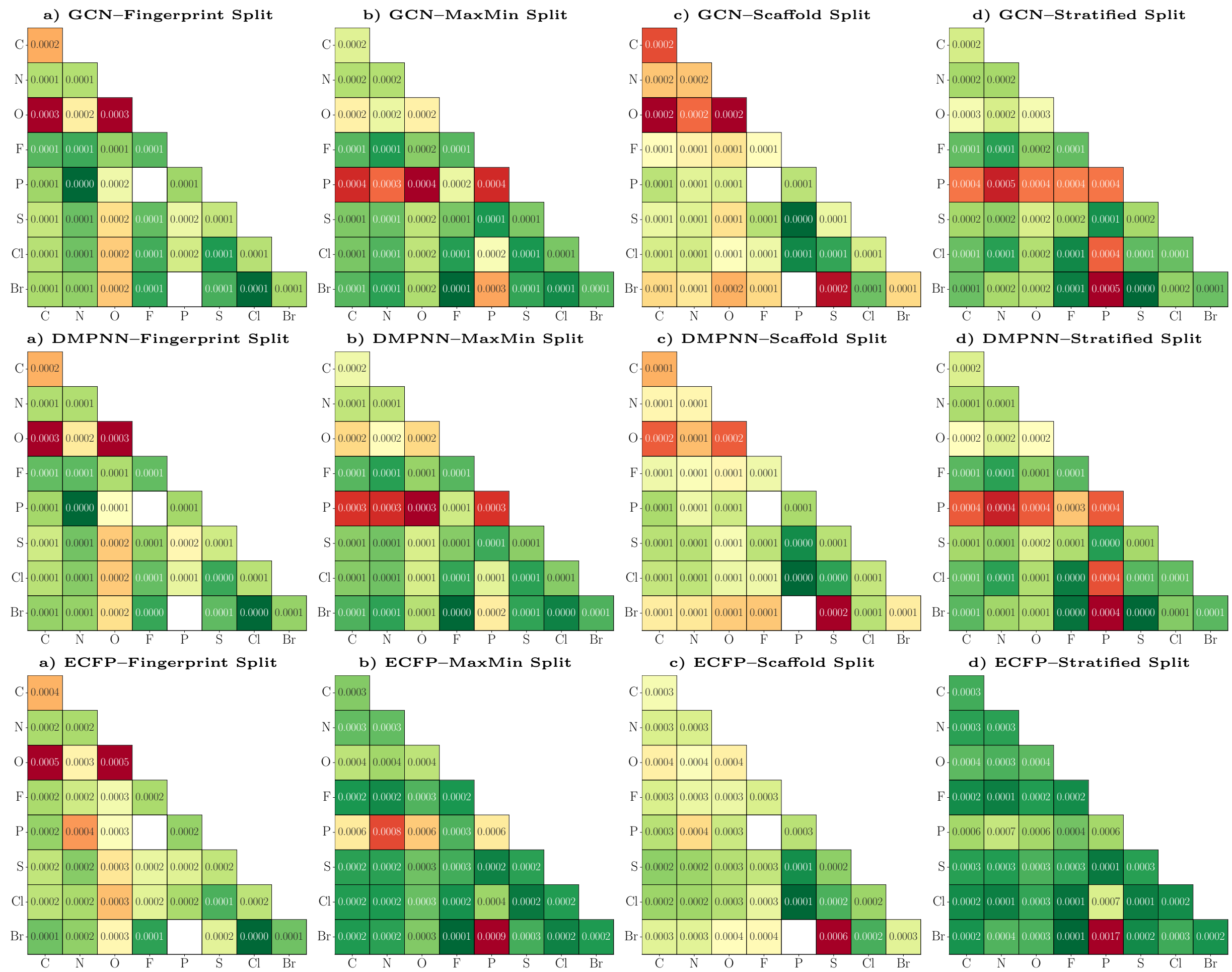

Figure S20: Prediction-error heatmaps for $p^{\mathrm{oh}}(\sigma)$ generated by the GCN, DMPNN, and ECFP-MLP models under four train–test splitting strategies: Fingerprint, MaxMin, Scaffold, and Stratified. Rows correspond to model type, while columns correspond to the splitting strategy. The heatmaps illustrate the spatial distribution of error across the chemical space, enabling comparison of how model performance and generalization depend on both architecture and data partitioning.

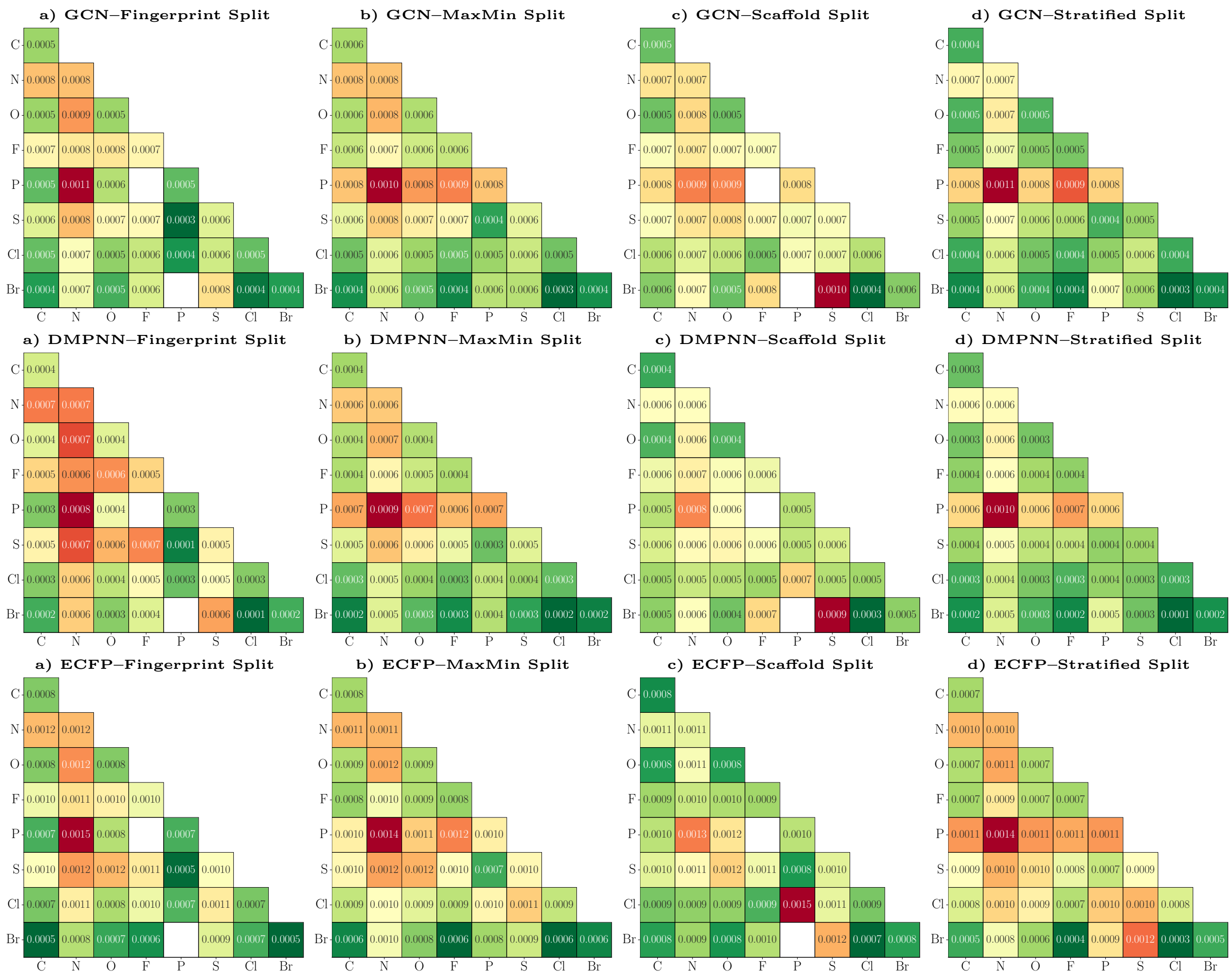

Figure S21: Prediction-error heatmaps for $p^{\mathrm{oh}}(\sigma)$ generated by the GCN, DMPNN, and ECFP-MLP models under four train–test splitting strategies: Fingerprint, MaxMin, Scaffold, and Stratified. Rows correspond to model type, while columns correspond to the splitting strategy. The heatmaps illustrate the spatial distribution of error across the chemical space, enabling comparison of how model performance and generalization depend on both architecture and data partitioning.

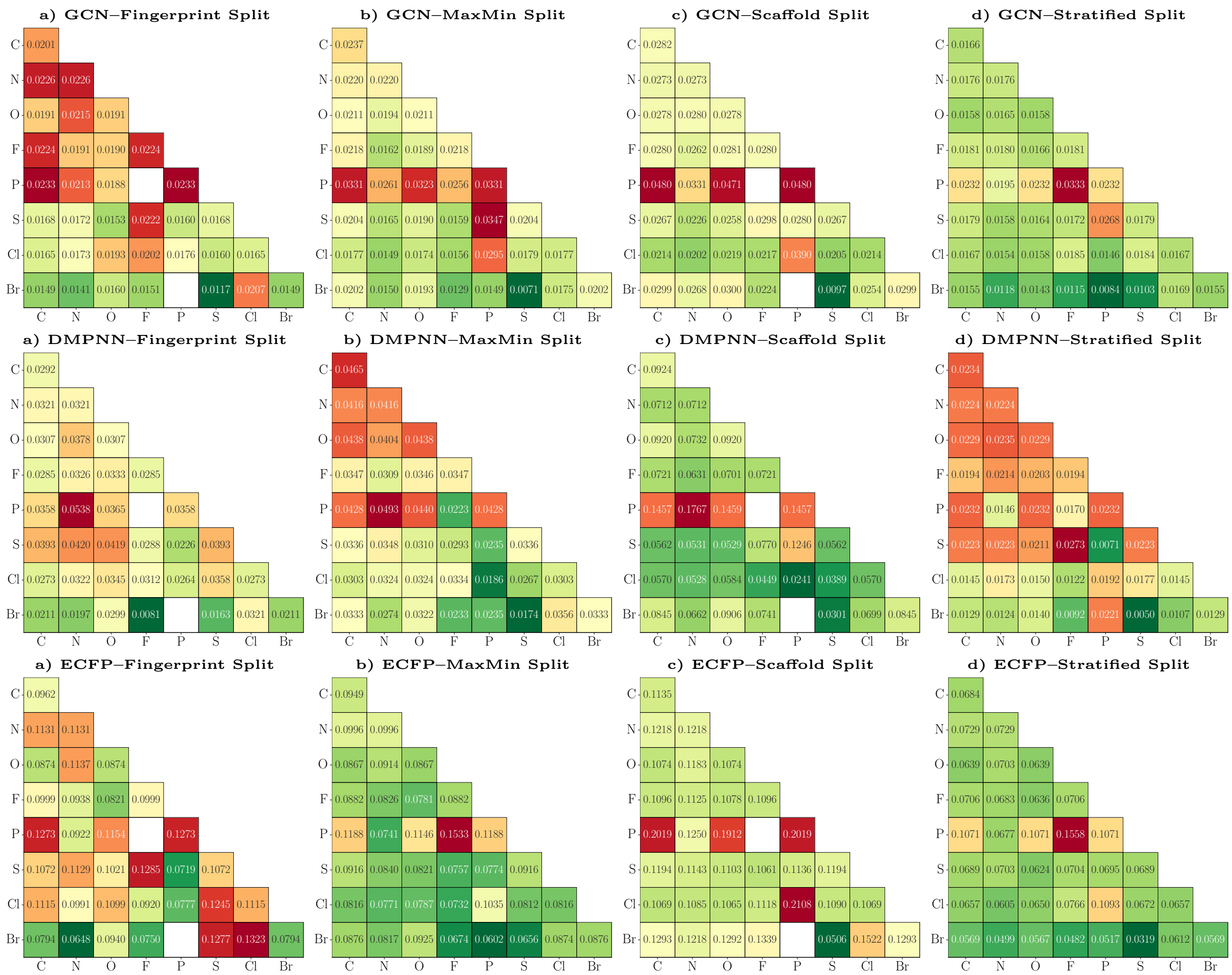

Figure S22: Prediction-error heatmaps for A generated by the GCN, DMPNN, and ECFP-MLP models under four train–test splitting strategies: Fingerprint, MaxMin, Scaffold, and Stratified. Rows correspond to model type, while columns correspond to the splitting strategy. The heatmaps illustrate the spatial distribution of error across the chemical space, enabling comparison of how model performance and generalization depend on both architecture and data partitioning.

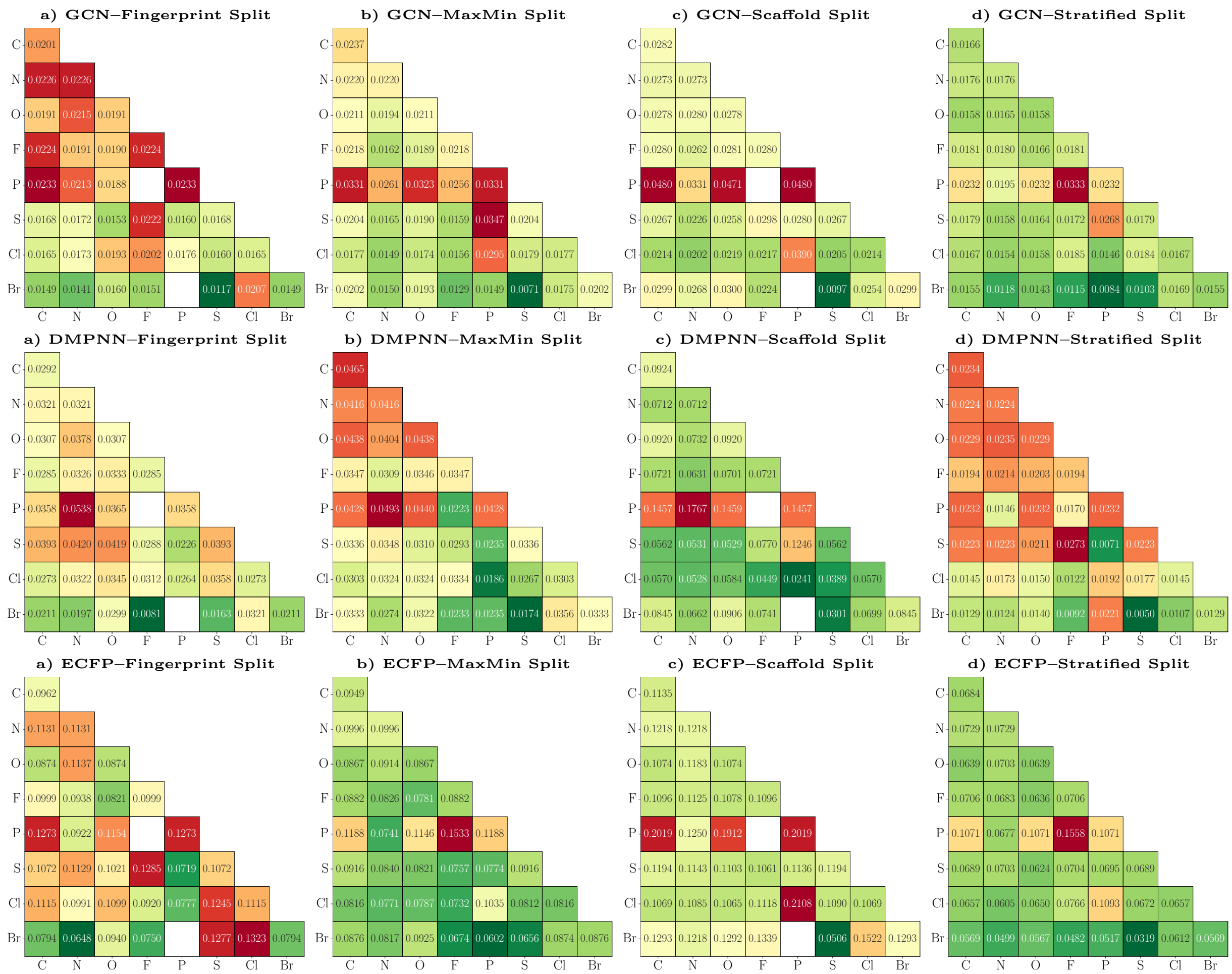

Figure S23: Prediction-error heatmaps for V generated by the GCN, DMPNN, and ECFP-MLP models under four train–test splitting strategies: Fingerprint, MaxMin, Scaffold, and Stratified. Rows correspond to model type, while columns correspond to the splitting strategy. The heatmaps illustrate the spatial distribution of error across the chemical space, enabling comparison of how model performance and generalization depend on both architecture and data partitioning.

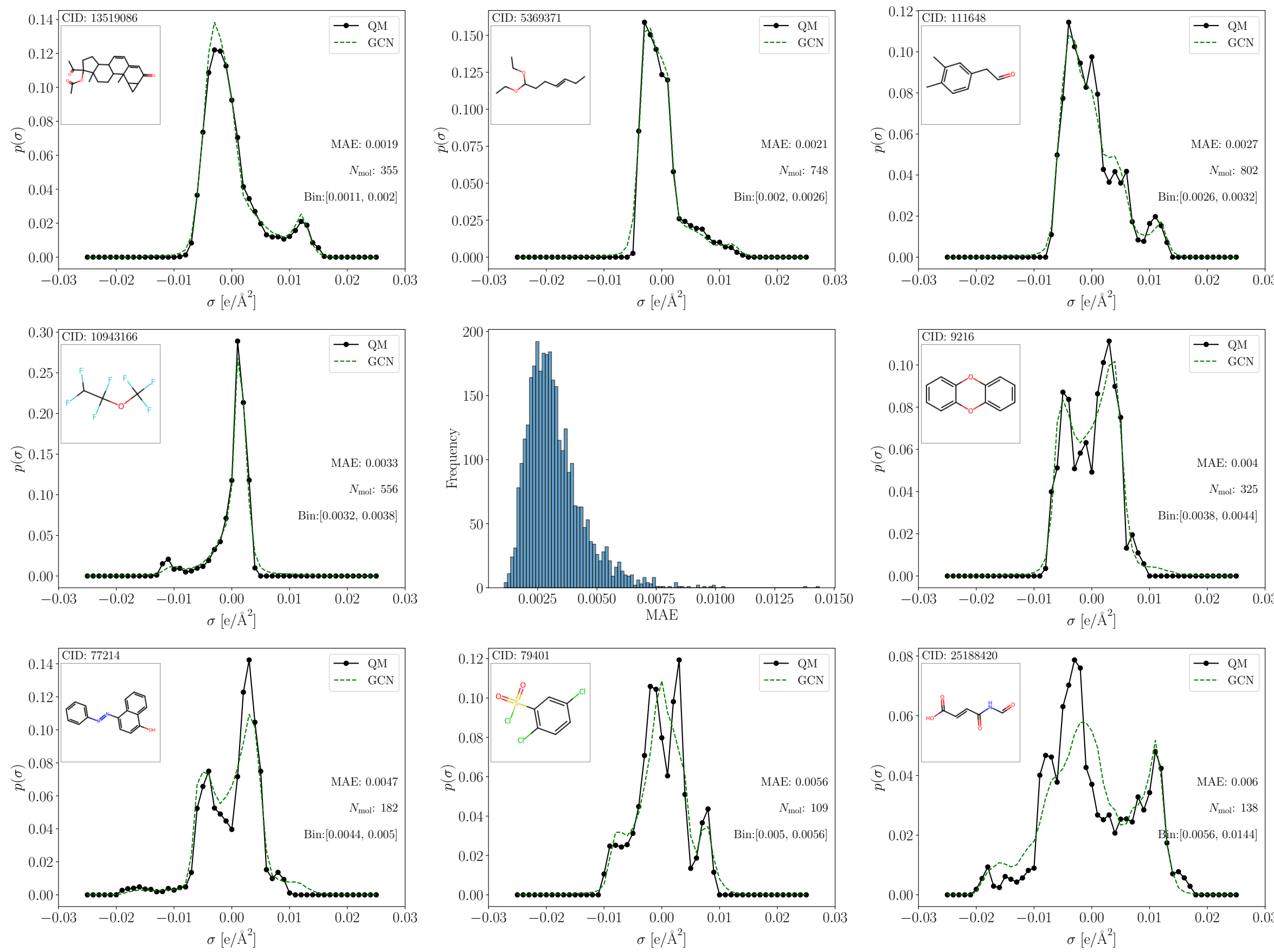

Figure S24: Comparison of the actual and GCN-predicted $p(\sigma)$ for eight randomly chosen molecules, each drawn from a different bin in the central MAE histogram. The CID, molecular diagram, MAE of the GCN model predictions, count of the molecules in the corresponding bin (N_{mol}), and bin range are shown on each $p(\sigma)$ plot.

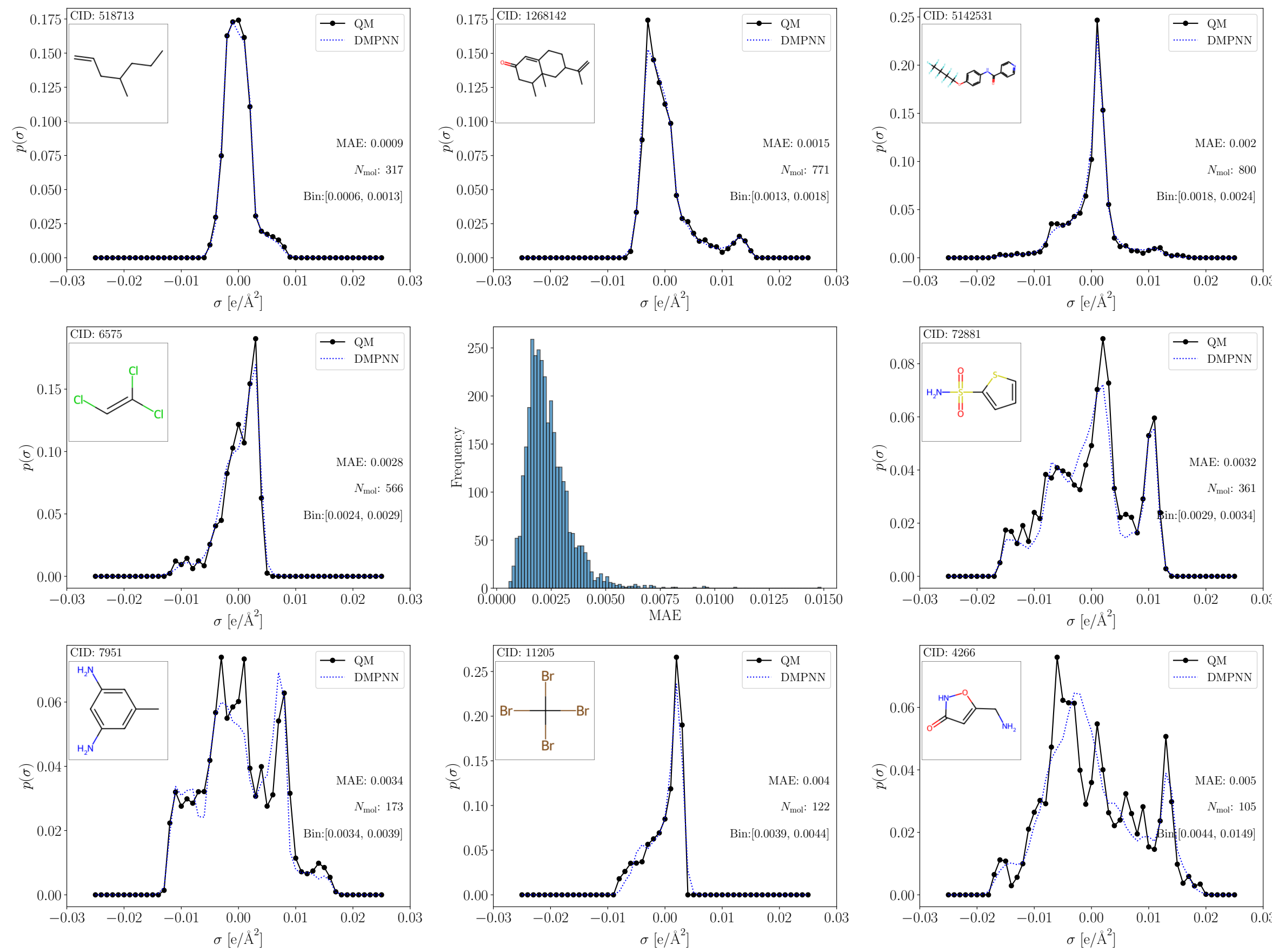

Figure S25: Comparison of the actual and DMPNN-predicted $p(\sigma)$ for eight randomly chosen molecules, each drawn from a different bin in the central MAE histogram. The CID, molecular diagram, MAE of the DMPNN model predictions, count of the molecules in the corresponding bin (N_{mol}), and bin range are shown on each $p(\sigma)$ plot.

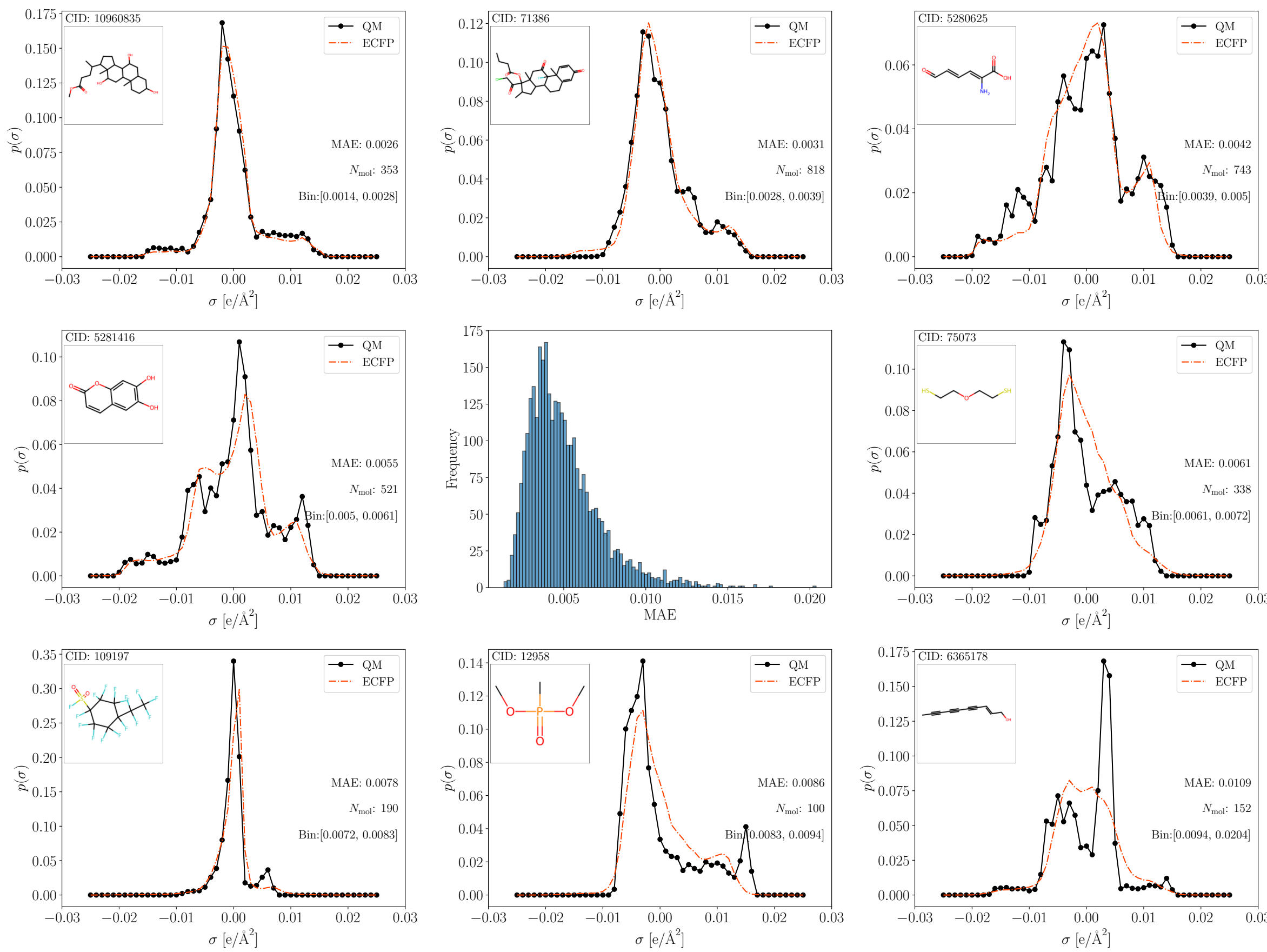

Figure S26: Comparison of the actual and ECFP-MLP-predicted $p(\sigma)$ for eight randomly chosen molecules, each drawn from a different bin in the central MAE histogram. The CID, molecular diagram, MAE of the DMPNN model predictions, count of the molecules in the corresponding bin (N_{mol}), and bin range are shown on each $p(\sigma)$ plot.

In Figures S27 and S28, we present $p(\sigma)$ profile of randomly selected compounds from the bins of histograms for the molecular weights and numbers of heavy atoms, respectively. It can be observed that either the models' predictions are in quantitative agreement with the data or at least the trend including the main peaks are captured. In agreement with our previous discussion, a significant difference between models cannot be observed in these figures, too.

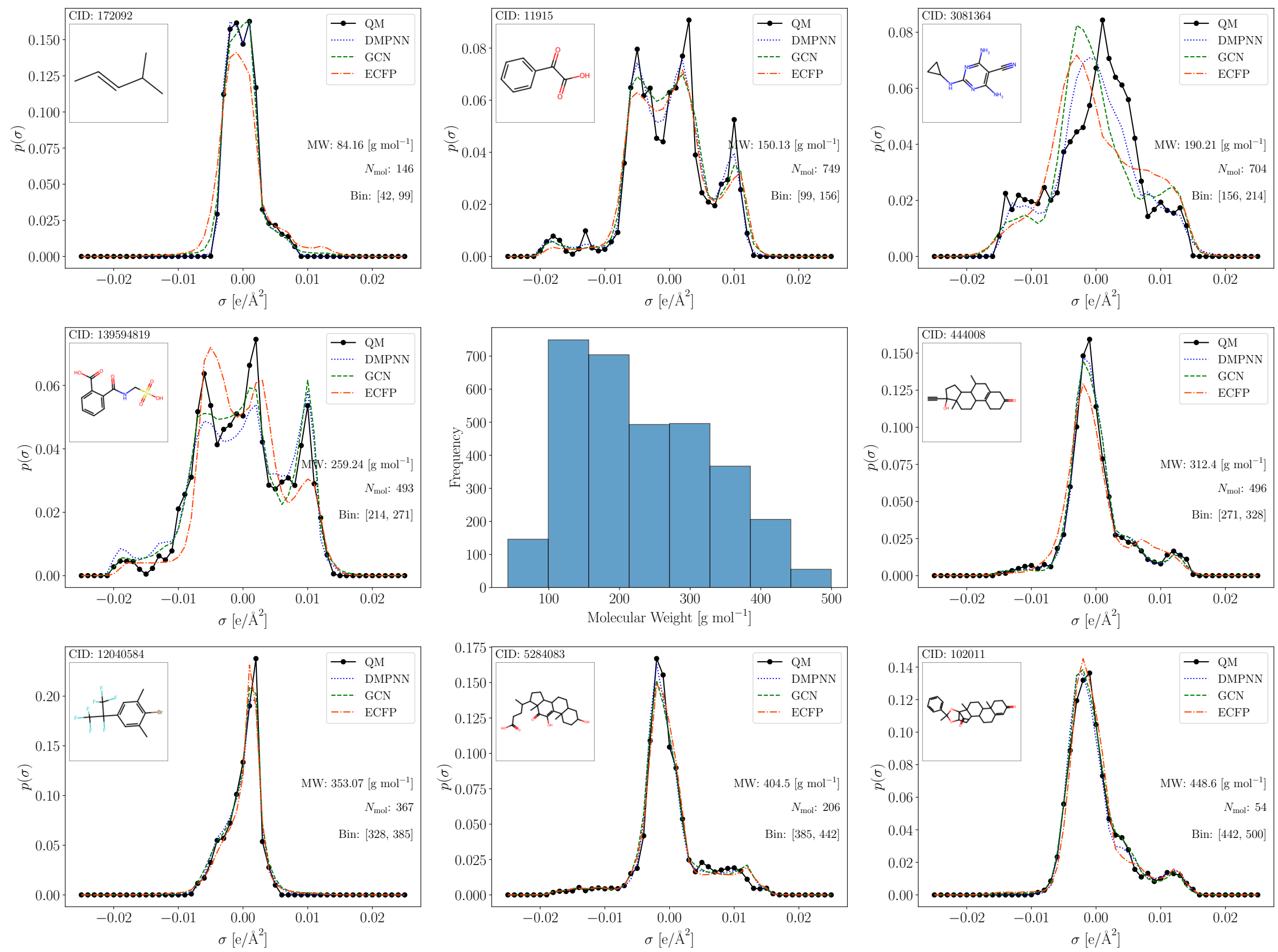

Figure S27: Comparison of the actual, GCN-predicted, and DMPNN-predicted $p(\sigma)$ for eight randomly chosen molecules, each drawn from a different bin in the central molecular weight histogram. The CID, molecular diagram, molecular weight, count of the molecules in the corresponding bin (N_{mol}), and bin range are shown on each $p(\sigma)$ plot.

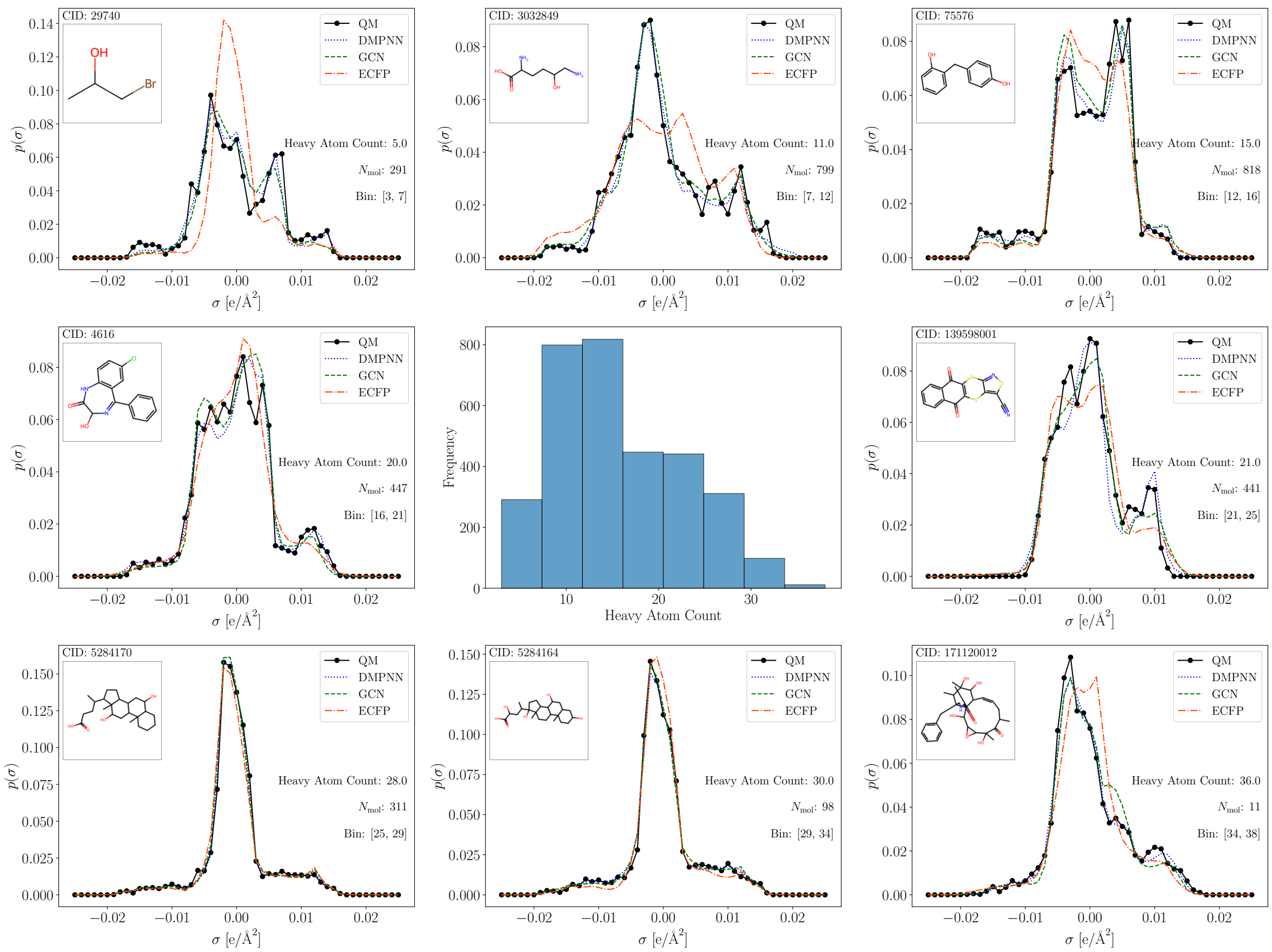

Figure S28: Comparison of the actual, GCN-predicted, and DMPNN-predicted $p(\sigma)$ for eight randomly chosen molecules, each drawn from a different bin in the central heavy atom count histogram. The CID, molecular diagram, heavy atom count, count of the molecules in the corresponding bin (N_{mol}), and bin range are shown on each $p(\sigma)$ plot.

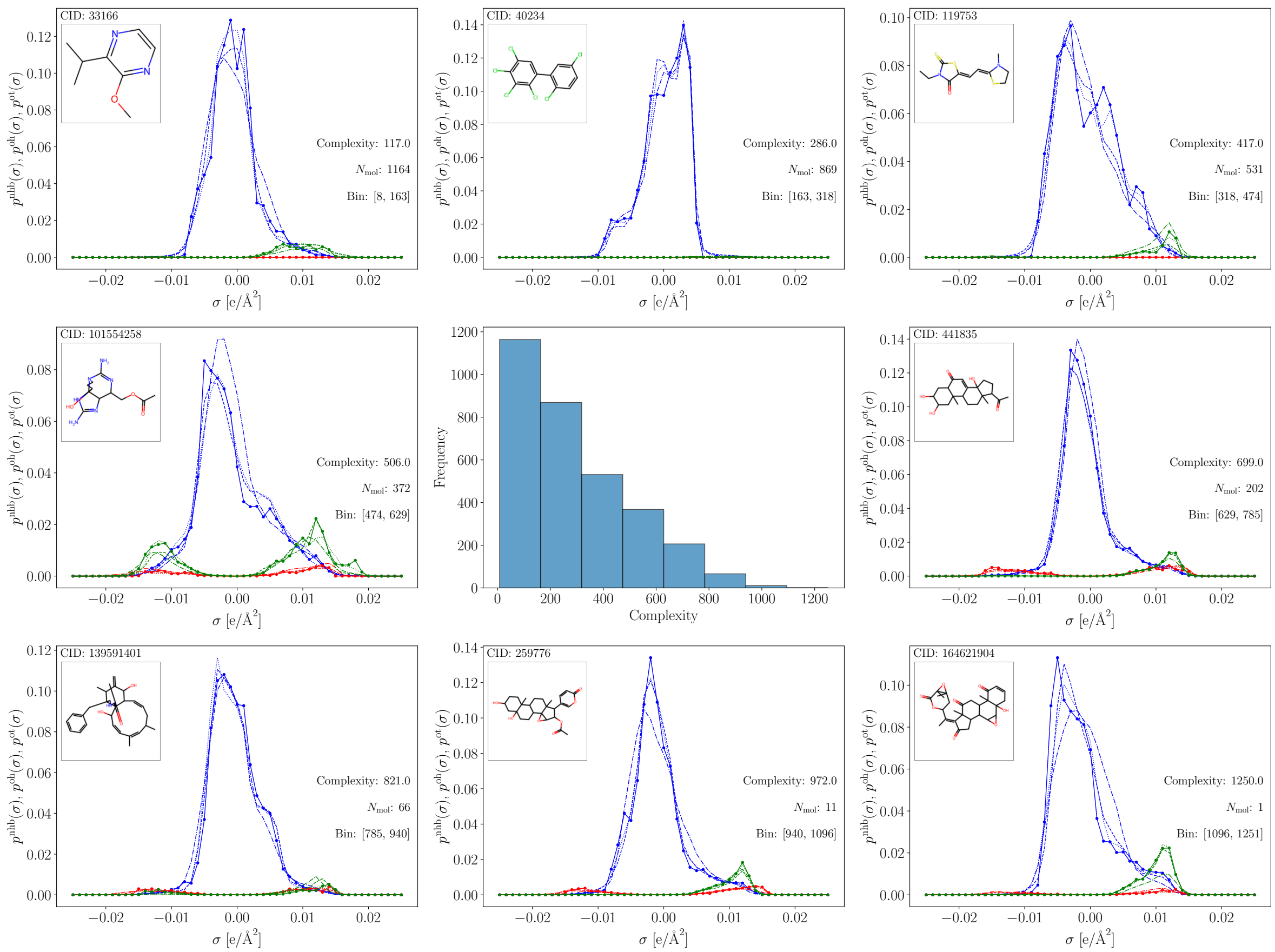

Figure S29: Comparison of the actual, GCN-predicted (dashed lines), DMPNN-predicted (dotted lines), and ECFP-MLP-predicted (dashed-dotted lines) $p^{\mathrm{nhb}}(\sigma)$ (blue lines), $p^{\mathrm{oh}}(\sigma)$ (red lines), and $p^{\mathrm{ot}}(\sigma)$ (green lines) for eight randomly chosen molecules, each drawn from a different bin in the central complexity index histogram. The CID, molecular diagram, complexity index, count of the molecules in the corresponding bin (N_{mol}), and bin range are shown on each $p^{\mathrm{nhb}}(\sigma)$, $p^{\mathrm{oh}}(\sigma)$, $p^{\mathrm{ot}}(\sigma)$ plot.

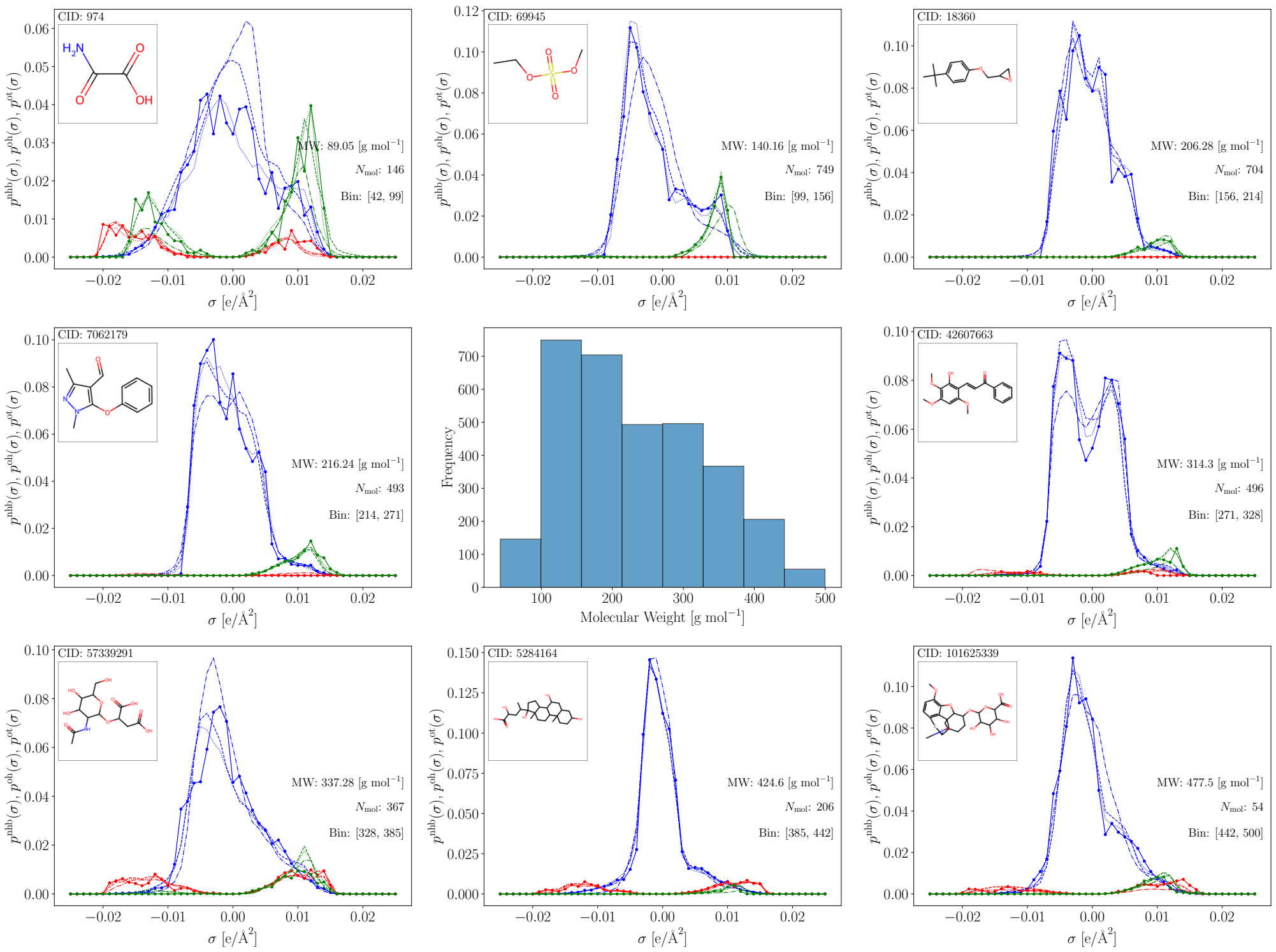

Figure S30: Comparison of the actual, GCN-predicted (dashed lines), DMPNN-predicted (dotted lines), and ECFP-MLP-predicted (dashed-dotted lines) $p^{\mathrm{nhb}}(\sigma)$ (blue lines), $p^{\mathrm{oh}}(\sigma)$ (red lines), and $p^{\mathrm{ot}}(\sigma)$ (green lines) for eight randomly chosen molecules, each drawn from a different bin in the central molecular weight histogram. The CID, molecular diagram, molecular weight, count of the molecules in the corresponding bin (N_{mol}), and bin range are shown on each $p^{\mathrm{nhb}}(\sigma)$, $p^{\mathrm{oh}}(\sigma)$, $p^{\mathrm{ot}}(\sigma)$ plot.

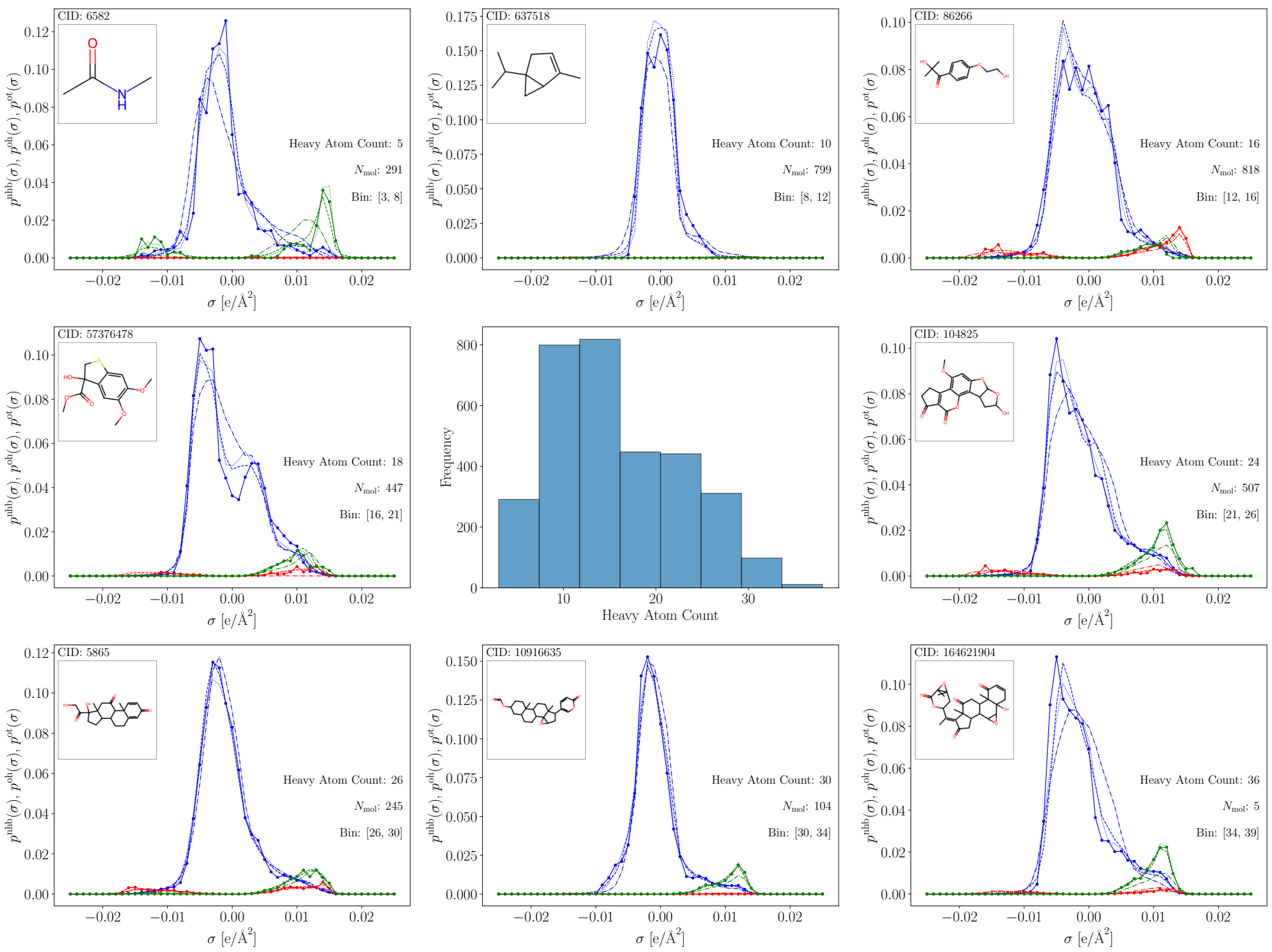

Figure S31: Comparison of the actual, GCN-predicted (dashed lines), DMPNN-predicted (dotted lines), and ECFP-MLP-predicted (dashed-dotted lines) $p^{\mathrm{nhb}}(\sigma)$ (blue lines), $p^{\mathrm{oh}}(\sigma)$ (red lines), and $p^{\mathrm{ot}}(\sigma)$ (green lines) for eight randomly chosen molecules, each drawn from a different bin in the central heavy atom count histogram. The CID, molecular diagram, heavy atom count, count of the molecules in the corresponding bin (N_{mol}), and bin range are shown on each $p^{\mathrm{nhb}}(\sigma)$, $p^{\mathrm{oh}}(\sigma)$, $p^{\mathrm{ot}}(\sigma)$ plot.

5 Evaluating the effect of using ML-generated COSMO descriptors on downstream thermodynamic modeling

5.1 Prediction of octanol–water partition coefficient

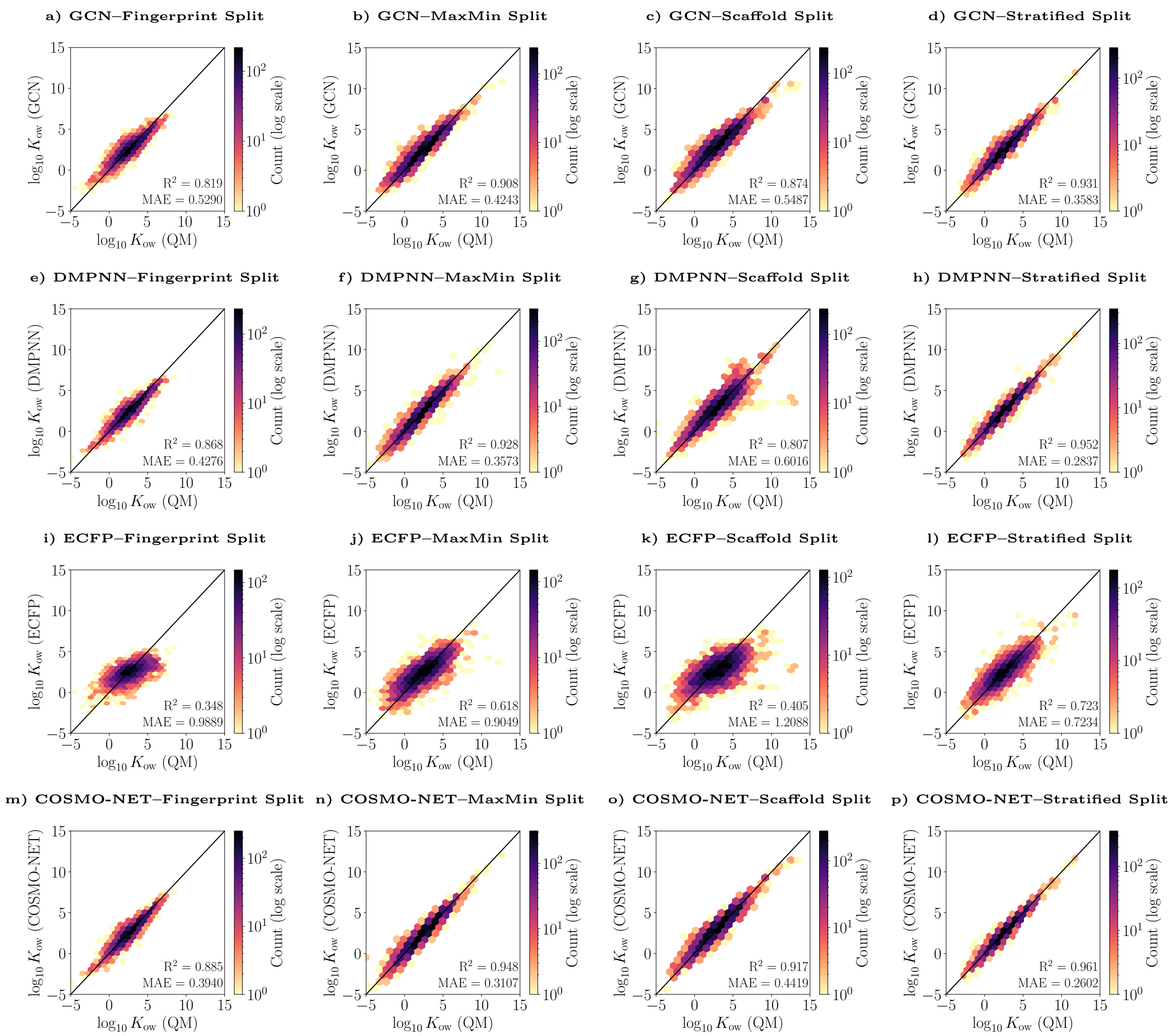

Figure S32: Parity plots for octanol–water partition coefficient ($\log K_{\text{ow}}$) predictions using COSMO-SAC 2002 with COSMO descriptors ($p(\sigma)$, A, V) from four models: GCN (a–d), DMPNN (e–h), ECFP-MLP (i–l), and COSMO-NET (m–p). Results are shown across four train-test splitting strategies (Fingerprint, MaxMin, Scaffold, and Stratified). Predictions are compared against reference values calculated from QM COSMO descriptors using only test set data. Hexagonal bin color indicates the number of data points.

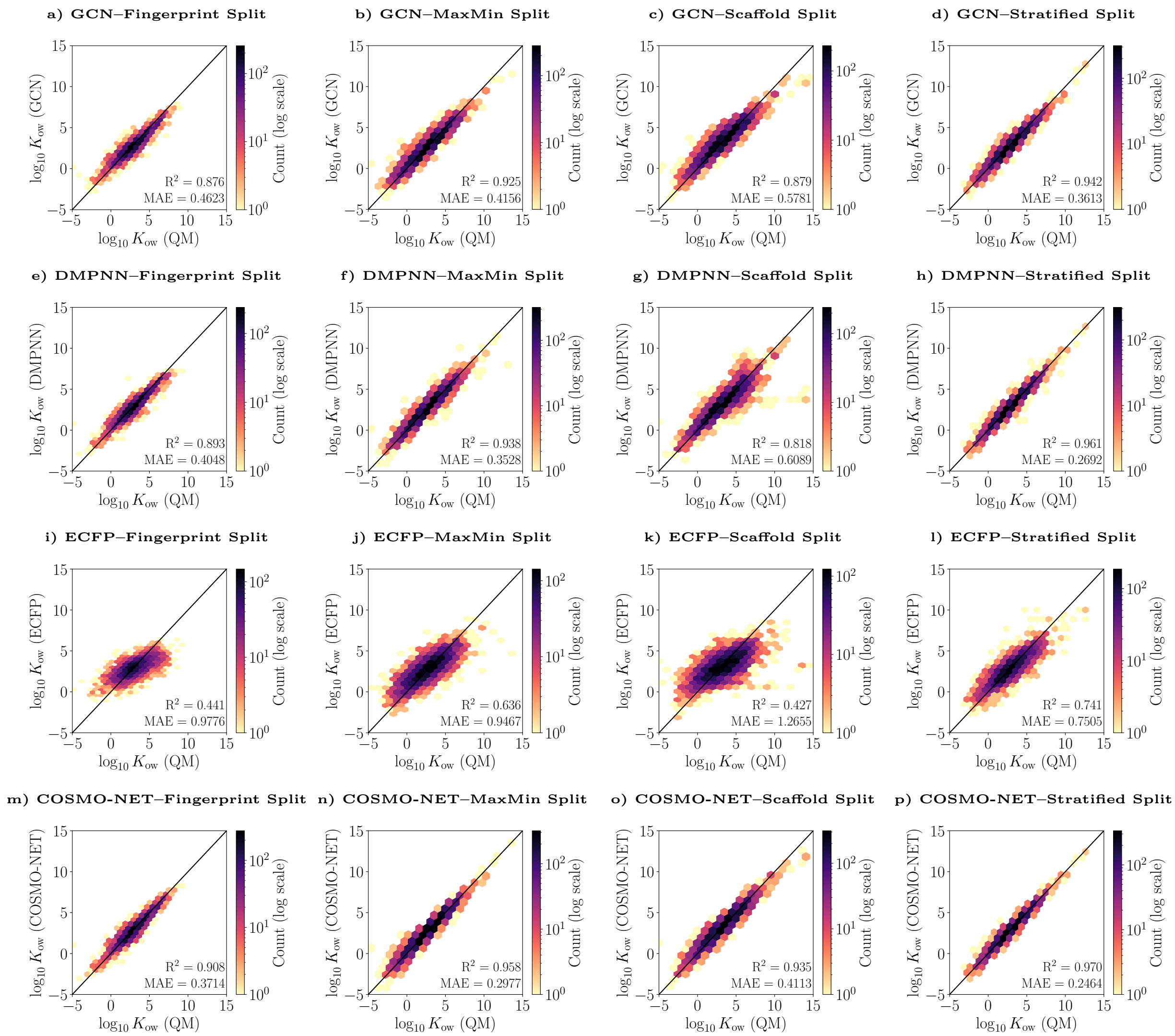

Figure S33: Parity plots for octanol–water partition coefficient ($\log K_{\mathrm{ow}}$) predictions using COSMO-SAC 2010 with COSMO descriptors ($p^{\mathrm{nhb}}(\sigma)$, $p^{\mathrm{oh}}(\sigma)$, $p^{\mathrm{ot}}(\sigma)$, A, V) from four models: GCN (a–d), DMPNN (e–h), ECFP-MLP (i–l), and COSMO-NET (m–p). Results are shown across four train-test splitting strategies (Fingerprint, MaxMin, Scaffold, and Stratified). Predictions are compared against reference values calculated from QM COSMO descriptors using only test set data. Hexagonal bin color indicates the number of data points.

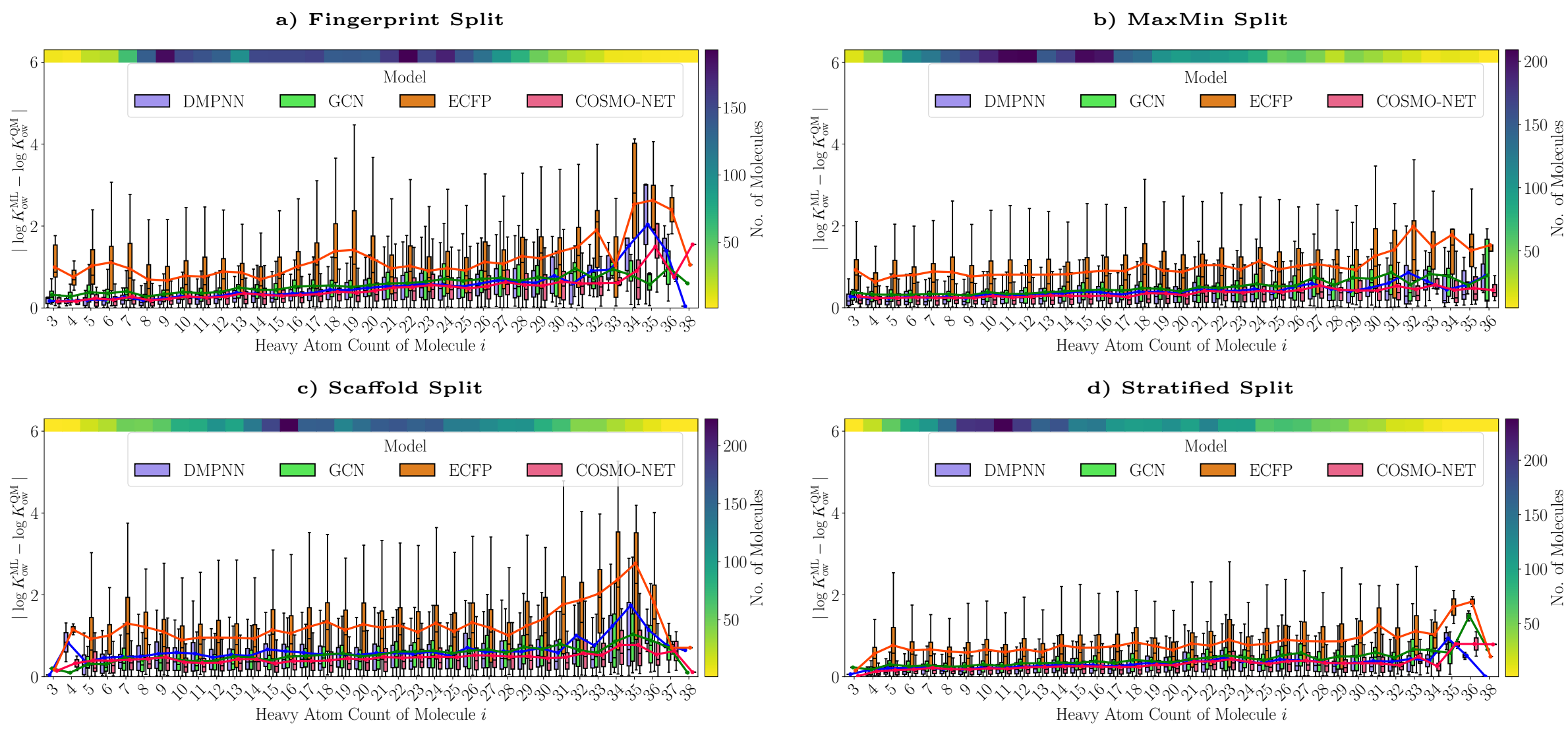

Figure S34: Errors of the predicted octanol-water partition coefficient using COSMO-SAC 2002 model where the corresponding probability densities, surface area and cavity volume are predicted by the DMPNN (blue box and curve), GCN (green box and curve), ECFP-MLP (orange box and curves), and COSMO-NET (red box and curve) models. The predictions are compared with the predictions of ocatnol-water partition coefficient using QM COSMO calculated probability densities, surface area and cavity volume. The heatmap on the top of the figure shows the number of compounds in each subset of the data according to the color map to the right of the figure. The points and continuous lines show the mean of the absolute error.

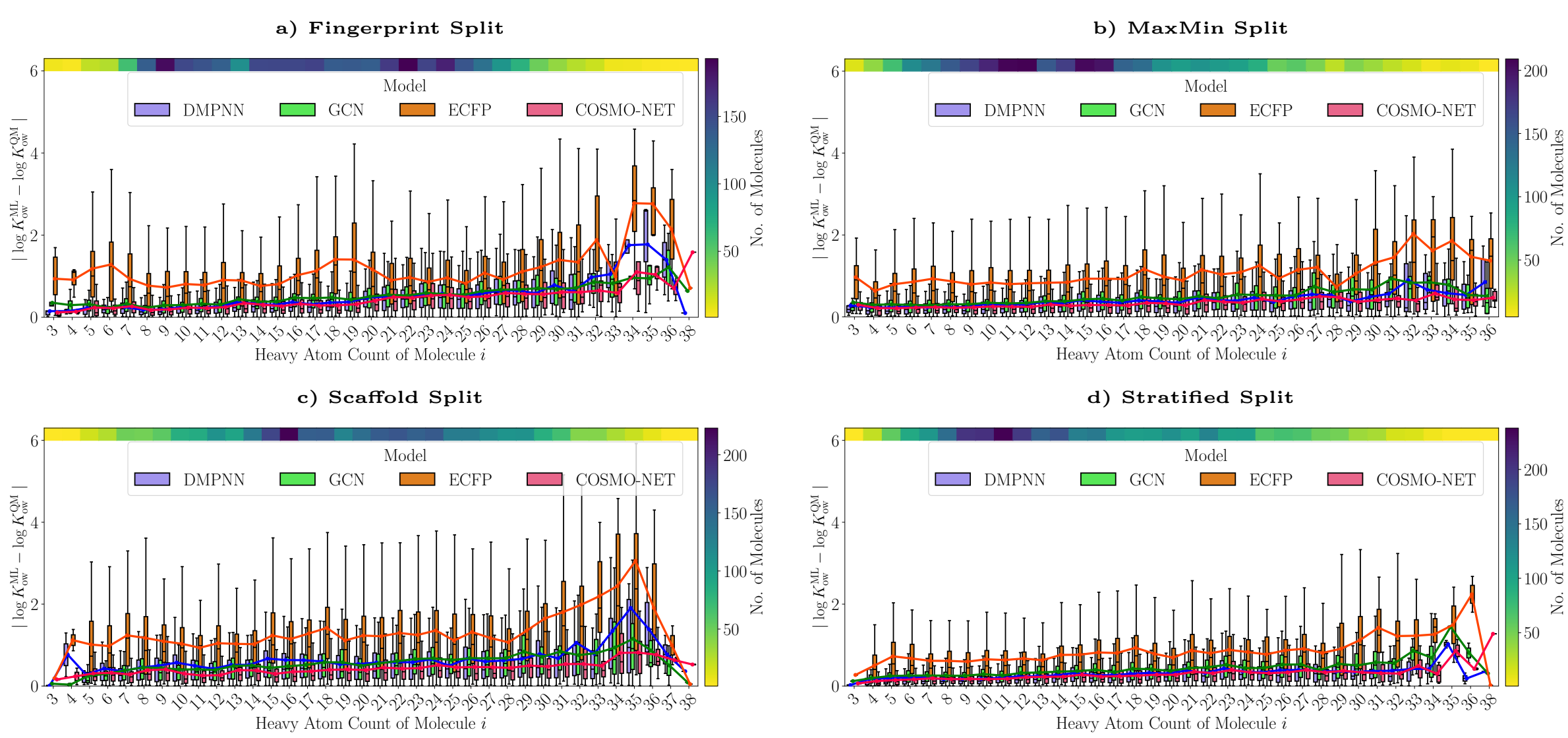

Figure S35: Errors of the predicted octanol-water partition coefficient using COSMO-SAC 2010 model where the corresponding probability densities, surface area and cavity volume are predicted by the DMPNN (blue box and curve), GCN (green box and curve), ECFP-MLP (orange box and curves), and COSMO-NET (red box and curve) models. The predictions are compared with the predictions of ocatnol-water partition coefficient using QM COSMO calculated probability densities, surface area and cavity volume. The heatmap on the top of the figure shows the number of compounds in each subset of the data according to the color map to the right of the figure. The points and continuous lines show the mean of the absolute error.

5.2 Prediction of solid–liquid solubility

Table S4: Fusion temperature and enthalpy of fusion of the solutes used for the solid–liquid solubility predictions.

**Formula**	**InChIKey**	Δh_i^{fus} **KJ/mol**	T_i^{fus} **/K**	**Phase**	**Source**
C3H9NO	HXKKHQJGJAFBHI-UHFFFAOYSA-N	12.31	274.9	cr to l	4
C6H3ClN2O4	VYZAHLCBVHPDDF-UHFFFAOYSA-N	20.17	325.2	unknown	[5, 6, 7]
C2H4Cl2	WSLDOOZREJYCGB-UHFFFAOYSA-N	8.74	237.7	cr,I to l	4
C6H3Cl3	PBKONEOXTCPAFI-UHFFFAOYSA-N	16.35	290.1	cr to l	4
C3H4O5	ROBFUDYVXSDBQM-UHFFFAOYSA-N	30.62	428.0	unknown	[5, 6, 7]
C5H6O5	KPGXRSRHYNQIFN-UHFFFAOYSA-N	28.59	388.7	unknown	[5, 6, 7]
C6H4Cl2O	RANCECPPZPIPNO-UHFFFAOYSA-N	22.43	331.0	cr to l	4
C7H6O4	YQUVCSBJEUQKSH-UHFFFAOYSA-N	32.60	474.5	unknown	[5, 6, 7]
C6H4N2	GZPHSAQLYPIAIN-UHFFFAOYSA-N	16.73	325.1	unknown	[5, 6, 7]
C7H6O2	IAVREABSGIHHMO-UHFFFAOYSA-N	24.85	376.8	unknown	[5, 6, 7]
C9H10O2	XMIIGOLPHOKFCH-UHFFFAOYSA-N	15.56	321.6	cr to l	4
C4H9NO2	BTCSSZJGUNDROE-UHFFFAOYSA-N	35.41	463.8	unknown	[5, 6, 7]
C7H6O2	RGHHSNMVTDWUBI-UHFFFAOYSA-N	21.37	390.9	unknown	[5, 6, 7]
C8H8O3	XQXPVVBIMDBYFF-UHFFFAOYSA-N	28.40	423.6	cr to l	4
C2H6O2	LYCAIKOWRPUZTN-UHFFFAOYSA-N	11.62	260.8	cr to l	4
C2H4O2	QTBSBXVTEAMEQO-UHFFFAOYSA-N	11.73	289.8	cr to l	4
C2H4O	IKHGUXGNUITLKF-UHFFFAOYSA-N	1.72	242.9	unknown	[5, 6, 7]
C2H5NO	DLFVBJFMPXGRIB-UHFFFAOYSA-N	15.59	353.3	cr to l	4
C3H6O	CSCPPACGZOOCGX-UHFFFAOYSA-N	4.76	178.6	cr,I to l	4
C6H10O4	WNLRTRBMVRJNCN-UHFFFAOYSA-N	34.85	425.5	cr to l	4
C7H7NO2	RWZYAGGXGHYGMB-UHFFFAOYSA-N	20.38	417.8	cr to l	4
C7H6O	HUMNYLRZRPPJDN-UHFFFAOYSA-N	9.32	216.0	cr to l	4
C6H6	UHOVQNZJYSORNB-UHFFFAOYSA-N	9.93	278.6	cr to l	4
C7H6O2	WPYMKLBDIGXBTP-UHFFFAOYSA-N	17.32	395.0	cr to l	4
C7H8O	WVDDGKGOMKODPV-UHFFFAOYSA-N	8.97	257.7	cr to l	4
C4H8O	ZTQSAGDEMFDKMZ-UHFFFAOYSA-N	11.11	176.8	cr to l	4
C4H10O2	OWBTYPJTUOEWEK-UHFFFAOYSA-N	11.20	299.0	unknown	[5, 6, 7]
C4H10O	LRHPLDYGYMQRHN-UHFFFAOYSA-N	9.28	183.9	cr to l	4
C4H8O2	FERIUCNNQQJTOY-UHFFFAOYSA-N	11.59	268.0	cr,I to l	4
C5H14N2	VHRGRCVQAFMJIZ-UHFFFAOYSA-N	29.82	285.0	unknown	[5, 6, 7]
CO2	CURLTUGMZLYLDI-UHFFFAOYSA-N	7.94	215.6	unknown	[5, 6, 7]
CH2O2	BDAGIHXWWSANSR-UHFFFAOYSA-N	12.70	34.5	cr to l	4
C6H6O2	YCIMNLLNPGFGHC-UHFFFAOYSA-N	22.76	377.5	cr to l	4
CH4	VNWKTOKETHGBQD-UHFFFAOYSA-N	0.90	89.8	cr to l	4
C2H3ClO2	FOCAUTSVDIKZOP-UHFFFAOYSA-N	16.30	334.3	cr,I to l	4
C6H8O7	KRKNYBCHXYNGOX-UHFFFAOYSA-N	48.51	429.5	unknown	[5, 6, 7]
C9H6O2	ZYGHJZDHTFUPRJ-UHFFFAOYSA-N	18.59	342.5	unknown	[5, 6, 7]
C7H8O	QWVGKYWNOKOFNN-UHFFFAOYSA-N	15.82	304.2	cr,I to l	4
C7H6O3	YGSDEFSMJLZEOE-UHFFFAOYSA-N	14.20	432.0	cr to l	4
C7H8O	RLSSMJSEOOYNOY-UHFFFAOYSA-N	10.71	285.4	cr,I to l	4
C8H18	TVMXDCGIABBOFY-UHFFFAOYSA-N	20.09	215.6	cr to l	4
C6H6O3	QCDYQQDYXPDABM-UHFFFAOYSA-N	34.50	491.8	unknown	[5, 6, 7]
C7H6O5	LNTHITQWFMADLM-UHFFFAOYSA-N	71.33	527.5	unknown	[5, 6, 7]
C8H16O2	WWZKQHOCKIZLMA-UHFFFAOYSA-N	21.38	289.5	cr to l	4
C7H12O4	WLJVNTCWHIRURA-UHFFFAOYSA-N	27.62	377.5	cr,I to l	4
C6H7NO	PLIKAWJENQZMHA-UHFFFAOYSA-N	31.20	459.5	cr to l	4
C3H10N2	XFNJVJPLKCPIBV-UHFFFAOYSA-N	32.60	383.1	cr to l	4
C7H5ClO2	LULAYUGMBFYYEX-UHFFFAOYSA-N	23.85	427.4	cr to l	4
C8H16O	NUJGJRNETVAIRJ-UHFFFAOYSA-N	26.10	247.7	cr to l	4
C7H8O2	LHGVFZTZFXWLCP-UHFFFAOYSA-N	13.51	299.2	unknown	[5, 6, 7]
C4H6O4	ZIYVHBGGAOATLY-UHFFFAOYSA-N	30.73	403.0	unknown	[5, 6, 7]
C4H6O5	BJEPYKJPYRNKOW-UHFFFAOYSA-N	28.11	390.2	unknown	[5, 6, 7]
C3H6O	NBBJYMSMWIIQGU-UHFFFAOYSA-N	8.59	171.3	cr to l	4
C7H5NO3	BXRFQSNOROATLV-UHFFFAOYSA-N	24.73	378.9	unknown	[5, 6, 7]
C12H8O	TXCDCPKCNAJMEE-UHFFFAOYSA-N	18.60	355.7	cr,I to l	4

Continued on next page

Continued from previous page

Formula	InChIKey	Δh_i^{fus} KJ/mol	T_i^{fus}/K	Phase	Source
C4H5N3O	OPTASPLRGRRNAP-UHFFFAOYSA-N	35.00	606.0	unknown	[5, 6, 7]
C3H6O3	JVTAAEKCZFNVCJ-UHFFFAOYSA-N	11.34	290.0	cr to l	4
C9H8O2	VMUXSMXIQBNMGZ-UHFFFAOYSA-N	16.70	312.3	unknown	[5, 6, 7]
C2H7N	ROSDSFDQCJNGOL-UHFFFAOYSA-N	5.94	181.0	cr to l	4
C2H6OS	IAZDPXIOMUYVGZ-UHFFFAOYSA-N	14.37	291.7	cr to l	4
C2H7NO	HZAXFHJVJLSVMW-UHFFFAOYSA-N	13.56	283.5	unknown	[5, 6, 7]
C2H6O	LFQSCWFLJHTTHZ-UHFFFAOYSA-N	4.63	156.2	cr,I to l	4
CH2O	WSFSSNUMVMOOMR-UHFFFAOYSA-N	7.53	155.0	unknown	[5, 6, 7]
CH3NO	ZHNUHDYFZUAESO-UHFFFAOYSA-N	7.98	275.7	cr to l	4
C4H4O4	VZCYOOQTPOCHFL-UHFFFAOYSA-N	19.35	453.0	unknown	[5, 6, 7]
C6H6Cl6	JLYXXMFPNIAWKQ-UHFFFAOYSA-N	22.18	410.2	cr to l	4
C5H8O4	JFCQEDHGNNZCLN-UHFFFAOYSA-N	20.90	371.0	cr,I to l	4
C3H8O3	PEDCQBHIVMGVHV-UHFFFAOYSA-N	16.40	286.1	cr to l	4
C2H4O3	AEMRFAOFKBGASW-UHFFFAOYSA-N	19.60	351.1	unknown	[5, 6, 7]
CHN	LELOWRISYMNNSU-UHFFFAOYSA-N	8.41	259.9	cr,I to l	4
CHNS	ZMZDMBWJUHKJPS-UHFFFAOYSA-N	10.87	163.2	unknown	[5, 6, 7]
C6H6O2	QIGBRXMKCJKVMJ-UHFFFAOYSA-N	27.11	445.5	cr to l	4
C3H4N2	RAXXELZNTBOGNW-UHFFFAOYSA-N	12.82	362.7	cr to l	4
C8H7N	SIKJAQJRHWYJAI-UHFFFAOYSA-N	10.75	325.8	unknown	[5, 6, 7]
C5H12O5	HEBKCHPVOIAQTA-UHFFFAOYSA-N	38.90	379.4	cr to l	4
C18H30O2	DTOSIQBPPRVQHS-UHFFFAOYSA-N	32.00	262.8	unknown	[5, 6, 7]
C3H4O4	OFOBLEOULBTSOW-UHFFFAOYSA-N	20.92	406.8	unknown	[5, 6, 7]
C5H6O4	HNEGQIOMVPPMNR-UHFFFAOYSA-N	21.50	360.9	unknown	[5, 6, 7]
CH4S	LSDPWZHWYPCBBB-UHFFFAOYSA-N	5.91	150.2	cr,I to l	4
CH4O	OKKJLVBELUTLKV-UHFFFAOYSA-N	2.20	176.0	cr,I to l	4
C6H12O6	CDAISMWEOUEBRE-UHFFFAOYSA-N	47.90	496.9	cr to l	4
C13H16N2O2	DRLFMBDRBRZALE-UHFFFAOYSA-N	34.90	390.6	unknown	[5, 6, 7]
C8H9NO	FZERHIULMFGESH-UHFFFAOYSA-N	22.50	387.6	cr to l	4
C10H8	UFWIBTONFRDIAS-UHFFFAOYSA-N	19.00	353.1	cr to l	4
C6H6N2O	DFPAKSUCGFBDDF-UHFFFAOYSA-N	22.84	402.0	cr to l	4
C6H5NO2	PVNIIMVLHYAWGP-UHFFFAOYSA-N	34.74	509.5	unknown	[5, 6, 7]
C8H11N	JLTDJTHDQAWBAV-UHFFFAOYSA-N	11.56	275.6	unknown	[5, 6, 7]
C8H18O	KBPLFHHGFOOTCA-UHFFFAOYSA-N	42.30	256.2	cr to l	4
C18H34O2	ZQPPMHVWECSIRJ-UHFFFAOYSA-N	61.55	320.1	cr to l	4
C4H4O5	KHPXUQMNIQBQEV-UHFFFAOYSA-N	50.38	437.0	unknown	[5, 6, 7]
C2H2O4	MUBZPKHOEPUJKR-UHFFFAOYSA-N	3.42	370.0	unknown	[5, 6, 7]
C7H7NO2	ALYNCZNDIQEVRV-UHFFFAOYSA-N	20.92	461.4	cr to l	4
C6H5NO3	BTJIUGUIPKRLHP-UHFFFAOYSA-N	24.27	387.0	cr to l	4
C16H32O2	IPCSVZSSVZVIGE-UHFFFAOYSA-N	53.10	328.2	cr to l	4
C10H14NO5PS	LCCNCVORNKJIRZ-UHFFFAOYSA-N	19.87	279.4	cr to l	4
C6HCl5O	IZUPBVBPLAPZRR-UHFFFAOYSA-N	17.15	462.5	cr to l	4
C14H10	YNPNZTXNASCQKK-UHFFFAOYSA-N	18.60	418.0	cr,I to l	4
C6H6O	ISWSIDIOOBJBQZ-UHFFFAOYSA-N	10.58	312.7	cr to l	4
C8H8O2	WLJVXDMOQOGPHL-UHFFFAOYSA-N	14.47	348.0	cr to l	4
C8H6O4	XNGIFLGASWRNHJ-UHFFFAOYSA-N	37.30	471.7	unknown	[5, 6, 7]
C6H5NO2	SIOXPEMLGUPBBT-UHFFFAOYSA-N	26.55	410.0	unknown	[5, 6, 7]
C3H8O2	DNIAPMSPPWPWGF-UHFFFAOYSA-N	8.40	240.0	unknown	[5, 6, 7]
C3H8O	BDERNNFJNOPAEC-UHFFFAOYSA-N	5.19	147.0	cr to l	4
C3H6O2	XBDQKXXYIPTUBI-UHFFFAOYSA-N	7.63	252.3	cr to l	4
C4H12N2	KIDHWZJUCRJVML-UHFFFAOYSA-N	28.06	295.1	unknown	[5, 6, 7]
C5H5N3O	IPEHBUMCGVEMRF-UHFFFAOYSA-N	28.40	463.0	cr to l	4
C3H4N2	WTKZEGDFNFYCGP-UHFFFAOYSA-N	13.80	333.1	cr to l	4
C5H5N	JUJWROOIHBZHMG-UHFFFAOYSA-N	7.06	233.1	cr to l	4
C2H6S	QMMFVYPAHWMCMS-UHFFFAOYSA-N	7.99	174.9	cr to l	4
C3H7NO2	FSYKKLYZXJSNPZ-UHFFFAOYSA-N	20.60	481.6	unknown	[5, 6, 7]
C4H6O4	KDYFGRWQOYBRFD-UHFFFAOYSA-N	33.36	457.0	cr,I to l	4
C2H7NO3S	XOAAWQZATWQOTB-UHFFFAOYSA-N	22.20	588.5	unknown	[5, 6, 7]
C4H8S	RAOIDOHSFRTOEL-UHFFFAOYSA-N	7.35	177.0	cr to l	4

Continued on next page

Continued from previous page

**Formula**	**InChIKey**	Δh_i^{fus} **KJ/mol**	T_i^{fus}/K	**Phase**	**Source**
C4H8OS	ISXOBTBCNRIIQO-UHFFFAOYSA-N	0.51	231.8	unknown	[5, 6, 7]
C5H6N2O2	RWQNBRDOKXIBIV-UHFFFAOYSA-N	17.50	321.3	cr to l	4
C7H8	YXFVVABEGXRONW-UHFFFAOYSA-N	6.62	178.0	cr to l	4
C3H9N	GETQZCLCWQTVFV-UHFFFAOYSA-N	7.00	156.1	cr to l	4
CH4N2O	XSQUKJJJFZCRTK-UHFFFAOYSA-N	13.60	406.2	cr to l	4
C8H8O3	MWOOGOJBHIARFG-UHFFFAOYSA-N	22.14	355.0	unknown	[5, 6, 7]
C8H8O3	IWYDHOAUDWTVEP-UHFFFAOYSA-N	25.86	397.8	unknown	[5, 6, 7]
C12H8N2	DGEZNRSVGBDHLK-UHFFFAOYSA-N	13.93	392.9	unknown	[5, 6, 7]
C2H8N2	DIIIISSCIXVANO-UHFFFAOYSA-N	13.64	264.3	cr to l	4
C4H6N2	MCTWTZJPVLRJOU-UHFFFAOYSA-N	12.21	270.4	unknown	[5, 6, 7]
C10H8N2	ROFVEXUMMXZLPA-UHFFFAOYSA-N	20.15	343.5	unknown	[5, 6, 7]
C8H5Cl3O3	SMYMJHWAQXWPDB-UHFFFAOYSA-N	34.90	428.0	cr to l	4
C6H3Br3O	BSWWXRFVMJHFBN-UHFFFAOYSA-N	18.50	366.1	cr to l	4
C8H6Cl2O3	OVSKIKFHRZPJSS-UHFFFAOYSA-N	38.10	412.8	cr to l	4
C9H8Cl2O3	HWIGZMADSFQMOI-UHFFFAOYSA-N	25.10	315.4	cr to l	4
C10H10Cl2O3	YIVXMZJTEQBPQO-UHFFFAOYSA-N	41.00	390.9	cr to l	4
C7H6O4	UIAFKZKHHVMJGS-UHFFFAOYSA-N	31.00	501.7	unknown	[5, 6, 7]
C6H4N2O5	UFBJCMHMOXMLKC-UHFFFAOYSA-N	24.17	388.0	cr to l	4
C4H11NO2	UXFQFBNBSPQBJW-UHFFFAOYSA-N	2.99	384.1	cr,I to l	4
C13H11N	CFRFHWQYWJMEJN-UHFFFAOYSA-N	23.81	400.9	unknown	[5, 6, 7]
C2H4N4	KLSJWNVTNUYHDU-UHFFFAOYSA-N	24.70	428.9	cr to l	4
C5H6N2	NUKYPUAOHBNCPY-UHFFFAOYSA-N	20.10	432.0	cr to l	4
C9H7NO	MCJGNVYPOGVAJF-UHFFFAOYSA-N	22.10	348.0	cr to l	4
C4H10NO3PS	YASYVMFAVPKPKE-UHFFFAOYSA-N	20.60	365.1	cr to l	4
C8H9NO2	RZVAJINKPMORJF-UHFFFAOYSA-N	26.02	441.2	cr to l	4
C4H6N4O3S2	BZKPWHYZMXOIDC-UHFFFAOYSA-N	28.60	532.2	unknown	[5, 6, 7]
C13H9NO	FZEYVTFCMJSGMP-UHFFFAOYSA-N	32.50	640.0	unknown	[5, 6, 7]
C12H15N3O2S	HXHWSAZORRCQMX-UHFFFAOYSA-N	44.71	483.2	unknown	[5, 6, 7]
C17H13ClN4	VREFGVBLTWBCJP-UHFFFAOYSA-N	31.34	501.0	unknown	[5, 6, 7]
C10H17N	DKNWSYNQZKUICI-UHFFFAOYSA-N	59.90	298.2	Crystal	[8]
C7H8N4O2	ZFXYFBGIUFBOJW-UHFFFAOYSA-N	30.90	544.7	cr to l	4
C15H10O2	XGWFJBFNAQHLEF-UHFFFAOYSA-N	34.70	495.6	unknown	[5, 6, 7]
C11H12N2O	VEQOALNAAJBPNY-UHFFFAOYSA-N	27.50	382.0	cr to l	4
C9H8O4	BSYNRYMUTXBXSQ-UHFFFAOYSA-N	24.30	411.1	cr to l	4
C9H16O4	BDJRBEYXGGNYIS-UHFFFAOYSA-N	32.68	380.0	cr to l	4
C12H10N2	DMLAVOWQYNRWNQ-UHFFFAOYSA-N	22.40	342.2	cr to l	4
C8H12N2O3	FTOAOBMCPZCFFF-UHFFFAOYSA-N	25.86	457.1	unknown	[5, 6, 7]
C11H13NO4	XEGGRYVFLWGFHI-UHFFFAOYSA-N	29.40	402.8	cr to l	4
C10H12N2O3S	ZOMSMJKLGFBRBS-UHFFFAOYSA-N	21.80	412.8	cr to l	4
C7H7NO	KXDAEFPNCMNJSK-UHFFFAOYSA-N	20.50	400.4	cr to l	4
C20H12	FMMWHPNWAFZXNH-UHFFFAOYSA-N	17.32	454.2	cr,I to l	4
C9H11NO2	BLFLLBZGZJTVJG-UHFFFAOYSA-N	19.75	363.0	cr to l	4
C14H12O2	SESFRYSPDFLNCH-UHFFFAOYSA-N	20.40	295.0	cr to l	4
C3H4O2	VEZXCJBBBCKRPI-UHFFFAOYSA-N	9.41	239.9	cr to l	4
C14H22O2	JZODKRWQWUWGCD-UHFFFAOYSA-N	44.14	495.3	unknown	[5, 6, 7]
C14H10BrN3O	VMIYHDSEFNYJSL-UHFFFAOYSA-N	44.67	523.6	unknown	[5, 6, 7]
C6H14O6S2	COVZYZSDYWQREU-UHFFFAOYSA-N	45.15	383.2	unknown	[5, 6, 7]
C11H15NO2	IUWVALYLNVXWKX-UHFFFAOYSA-N	24.60	329.0	cr to l	4
C9H8O4	QAIPRVGONGVQAS-UHFFFAOYSA-N	27.61	501.1	unknown	[5, 6, 7]
C8H10N4O2	RYYVLZVUVIJVGH-UHFFFAOYSA-N	20.96	508.0	cr,I to l	4
C21H26O2	VBGLYOIFKLUMQG-UHFFFAOYSA-N	17.00	352.2	unknown	[5, 6, 7]
C15H12N2O	FFGPTBGBLSHEPO-UHFFFAOYSA-N	26.74	463.5	unknown	[5, 6, 7]
C12H15NO3	DUEPRVBVGDRKAG-UHFFFAOYSA-N	30.30	426.4	cr to l	4
C17H14F3N3O2S	RZEKVGVHFLEQIL-UHFFFAOYSA-N	35.84	435.1	unknown	[5, 6, 7]
C16H34O	BXWNKGSJHAJOGX-UHFFFAOYSA-N	34.72	320.0	cr,I to l	4
C2H3Cl3O2	RNFNDJAIBTYOQL-UHFFFAOYSA-N	23.00	349.1	cr to l	4
C9H5Cl2NO	WDFKMLRRRCGAKS-UHFFFAOYSA-N	39.20	452.2	unknown	[5, 6, 7]
C10H12ClNO2	CWJSHJJYOPWUGX-UHFFFAOYSA-N	20.50	315.7	cr to l	4

Continued on next page

Continued from previous page

Formula	InChIKey	Δh_i^{fus} KJ/mol	T_i^{fus}/K	Phase	Source
C7H4ClNO2	TZFWDZFKRBELIQ-UHFFFAOYSA-N	25.65	464.0	unknown	[5, 6, 7]
C10H18O	WEEGYLXZBRQIMU-UHFFFAOYSA-N	13.80	274.1	unknown	[5, 6, 7]
C16H13ClN2O2	CXOXHMZGEKVPMT-UHFFFAOYSA-N	30.40	455.1	cr to l	4
C7H8O	IWDCLRJOBJJRNH-UHFFFAOYSA-N	12.20	309.0	cr to l	4
C5H11N	NISGSNTVMOOSJQ-UHFFFAOYSA-N	8.31	190.5	cr,I to l	4
C7H15Cl2N2O2P	CMSMOCZEIVJLDB-UHFFFAOYSA-N	33.10	323.5	cr to l	4
C10H6Cl4O4	NPOJQCVWMSKXDN-UHFFFAOYSA-N	30.50	431.3	cr to l	4
C14H8O4	QBPFLULOKWLNNW-UHFFFAOYSA-N	20.06	471.2	unknown	[5, 6, 7]
C10H20O2	GHVNFZFCNZKVNT-UHFFFAOYSA-N	29.20	300.1	cr to l	4
C15H11ClN2O	AKPLHCDWDRPJGD-UHFFFAOYSA-N	29.87	490.3	unknown	[5, 6, 7]
C16H13ClN2O	AAOVKJBEBIDNHE-UHFFFAOYSA-N	24.78	404.7	unknown	[5, 6, 7]
C8H7ClN2O2S	GDLBFKVLRPITMI-UHFFFAOYSA-N	34.10	600.4	unknown	[5, 6, 7]
C12H8S	IYYZUPMFVPLQIF-UHFFFAOYSA-N	22.35	372.4	cr to l	4
C8H6Cl2O3	IWEDIXLBFLAXBO-UHFFFAOYSA-N	22.59	386.5	cr to l	4
C7H3Cl2N	YOYAIZYFCNQIRF-UHFFFAOYSA-N	25.90	416.7	cr to l	4
C14H11Cl2NO2	DCOPUUMXTXDBNB-UHFFFAOYSA-N	39.40	453.5	unknown	[5, 6, 7]
C12H8Cl6O	DFBKLUNHFCTMDC-UHFFFAOYSA-N	2.72	452.0	cr,I to l	4
C13H8F2O3	HUPFGZXOMWLGNK-UHFFFAOYSA-N	36.00	484.2	unknown	[5, 6, 7]
C5H7N3O2	IBXPYPUJPLLOIN-UHFFFAOYSA-N	19.11	411.7	unknown	[5, 6, 7]
C13H10O	RWCCWEUUXYIKHB-UHFFFAOYSA-N	17.70	321.2	cr to l	4
C9H10Cl2N2O	XMTQQYYKAHVGBJ-UHFFFAOYSA-N	33.90	429.7	cr to l	4
C10H14N4O4	KSCFJBIXMNOVSH-UHFFFAOYSA-N	33.88	437.0	unknown	[5, 6, 7]
C9H6Cl6O3S	RDYMFSUJUZBWLH-UHFFFAOYSA-N	10.00	381.3	cr to l	4
C4H10O	RTZKZFJDLAIYFH-UHFFFAOYSA-N	8.49	156.1	cr,I to l	4
C9H10N2O3S2	OUZWUKMCLIBBOG-UHFFFAOYSA-N	39.20	464.0	cr to l	4
C2H8N2	PIICEJLVQHRZGT-UHFFFAOYSA-N	35.30	417.7	cr to l	4
C10H12O2	RRAFCDWBNXTKKO-UHFFFAOYSA-N	17.00	261.8	unknown	[5, 6, 7]
C14H12O2	QRZAKQDHEVVFRX-UHFFFAOYSA-N	29.76	437.0	unknown	[5, 6, 7]
C16H14O3	ZPAKPRAICRBAOD-UHFFFAOYSA-N	50.89	459.9	unknown	[5, 6, 7]
C15H13FO2	SYTBZMRGLBWNTM-UHFFFAOYSA-N	27.10	386.2	unknown	[5, 6, 7]
C11H11F3N2O3	MKXKFYHWDHIYRV-UHFFFAOYSA-N	30.94	383.4	unknown	[5, 6, 7]
C12H11ClN2O5S	ZZUFCTLCJUWOSV-UHFFFAOYSA-N	21.07	508.2	unknown	[5, 6, 7]
C7H6O4	WXTMDXOMEHJXQO-UHFFFAOYSA-N	25.00	476.9	unknown	[5, 6, 7]
C2HBrClF3	BCQZXOMGPXTTIC-UHFFFAOYSA-N	4.84	157.4	cr to l	4
C10H5Cl7	FRCCEHPWNOQAEU-UHFFFAOYSA-N	1.88	369.0	cr,I to l	4
C12H18O2	WFJIVOKAWHGMBH-UHFFFAOYSA-N	19.04	341.5	cr to l	4
C7H8ClN3O4S2	JZUFKLXOESDKRF-UHFFFAOYSA-N	33.03	543.2	unknown	[5, 6, 7]
C13H18O2	HEFNNWSXXWATRW-UHFFFAOYSA-N	24.47	341.3	unknown	[5, 6, 7]
C17H15NO3	RJMIEHBSYVWVIN-UHFFFAOYSA-N	38.17	484.8	unknown	[5, 6, 7]
C18H16O3	SFBODOKJTYAUCM-UHFFFAOYSA-N	43.64	389.9	unknown	[5, 6, 7]
C6H7N3O	QRXWMOHMRWLFEY-UHFFFAOYSA-N	28.88	445.8	unknown	[5, 6, 7]
C3H8O	KFZMGEQAYNKOFK-UHFFFAOYSA-N	5.30	184.6	cr to l	4
C15H13NO3	OZWKMVRBQXNZKK-UHFFFAOYSA-N	75.26	429.2	unknown	[5, 6, 7]
C14H12O5	HSMPDPBYAYSOBC-UHFFFAOYSA-N	30.11	425.0	unknown	[5, 6, 7]
C12H24O2	POULHZVOKOAJMA-UHFFFAOYSA-N	36.65	316.9	cr to l	4
C12H9F3N2O2	VHOGYURTWQBHIL-UHFFFAOYSA-N	31.54	440.9	unknown	[5, 6, 7]
C18H32O2	OYHQOLUKZRVURQ-UHFFFAOYSA-N	37.47	277.1	unknown	[5, 6, 7]
C15H10Cl2N2O2	DIWRORZWFLOCLC-UHFFFAOYSA-N	83.88	449.9	unknown	[5, 6, 7]
C16H15ClN2	YLCXGBZIZBEVPZ-UHFFFAOYSA-N	21.50	374.1	cr to l	4
C15H15NO2	HYYBABOKPJLUIN-UHFFFAOYSA-N	44.35	503.2	unknown	[5, 6, 7]
C7H17NO5	MBBZMMPHUWSWHV-UHFFFAOYSA-N	51.30	400.9	unknown	[5, 6, 7]
C11H8O2	MJVAVZPDRWSRRC-UHFFFAOYSA-N	21.46	376.0	cr to l	4
C10H14O3	JWDYCNIAQWPBHD-UHFFFAOYSA-N	32.10	353.7	unknown	[5, 6, 7]
C7H7NO3	KBOPZPXVLCULAV-UHFFFAOYSA-N	67.20	543.2	unknown	[5, 6, 7]
C4H6O2	CERQOIWHTDAKMF-UHFFFAOYSA-N	8.06	287.5	cr to l	4
C2H8NO2PS	NNKVPIKMPCQWCG-UHFFFAOYSA-N	13.30	319.9	cr to l	4
C5H10N2O2S	UHXUZOCRWCRNSJ-UHFFFAOYSA-N	24.30	351.7	cr to l	4
C8H8O3	OSWPMRLSEDHDFF-UHFFFAOYSA-N	16.29	271.5	unknown	[5, 6, 7]

Continued on next page

Continued from previous page

Formula	InChIKey	Δh_i^{fus} KJ/mol	T_i^{fus}/K	Phase	Source
C6H9N3O3	VAOCPAMSLUNLGC-UHFFFAOYSA-N	34.40	433.4	unknown	[5, 6, 7]
C13H11NO2	ZWJINEZUASEZBH-UHFFFAOYSA-N	39.70	458.2	unknown	[5, 6, 7]
C12H12N2O3	MHWLWQUZZRMNGJ-UHFFFAOYSA-N	35.91	501.5	unknown	[5, 6, 7]
C15H14N4O	NQDJXKOVJZTUJA-UHFFFAOYSA-N	23.14	516.2	unknown	[5, 6, 7]
C13H8Cl2N2O4	RJMUSRYZPJIFPJ-UHFFFAOYSA-N	40.70	505.4	unknown	[5, 6, 7]
C12H10F3N3O4	XWXYUMMDTVBTOU-UHFFFAOYSA-N	31.03	428.0	unknown	[5, 6, 7]
C13H12N2O5S	HYWYRSMBCFDLJT-UHFFFAOYSA-N	37.69	421.6	unknown	[5, 6, 7]
C3H5N3O9	SNIOPGDIGTZGOP-UHFFFAOYSA-N	21.00	280.7	unknown	[5, 6, 7]
C16H18FN3O3	OGJPXUAPXNRGGI-UHFFFAOYSA-N	35.63	491.0	unknown	[5, 6, 7]
C15H11ClN2O2	ADIMAYPTOBDMTL-UHFFFAOYSA-N	84.11	467.5	unknown	[5, 6, 7]
C14H12O3	DXGLGDHPHMLXJC-UHFFFAOYSA-N	21.77	336.7	unknown	[5, 6, 7]
C7H7NO3	WUBBRNOQWQTFEX-UHFFFAOYSA-N	55.00	409.3	unknown	[5, 6, 7]
C6H4O2	AZQWKYJCGOJGHM-UHFFFAOYSA-N	18.45	386.0	cr to l	4
C8H7NO4	YBADLXQNJCMBKR-UHFFFAOYSA-N	29.18	426.9	unknown	[5, 6, 7]
C6H5ClO	WXNZTHHGJRFXKQ-UHFFFAOYSA-N	14.07	316.0	cr to l	4
C6H4Cl2	OCJBOOLMMGQPQU-UHFFFAOYSA-N	18.39	325.7	cr,I to l	4
C9H6Cl2N2O3	LRUUNMYPIBZBQH-UHFFFAOYSA-N	29.50	397.0	cr to l	4
C13H18N4O3	BYPFEZZEUUWMEJ-UHFFFAOYSA-N	36.60	376.8	unknown	[5, 6, 7]
C10H13NO2	CPJSUEIXXCENMM-UHFFFAOYSA-N	31.30	410.0	cr to l	4
C12H8N2	PCNDJXKNXGMECE-UHFFFAOYSA-N	20.90	444.0	cr to l	4
C10H12O2	OBKXEAXTFZPCHS-UHFFFAOYSA-N	19.50	324.2	unknown	[5, 6, 7]
C14H17N5O3	JOHZPMXAZQZXHR-UHFFFAOYSA-N	32.85	529.9	unknown	[5, 6, 7]
C4H10N2	GLUUGHFHXGJENI-UHFFFAOYSA-N	22.10	384.6	cr to l	4
C6H10N2O2	GMZVRMREEHBGGF-UHFFFAOYSA-N	26.93	421.9	unknown	[5, 6, 7]
C10H19N5O	ISEUFVQQFVOBCY-UHFFFAOYSA-N	22.18	364.6	cr to l	4
C9H9Cl2NO	LFULEKSKNZEWOE-UHFFFAOYSA-N	20.08	364.5	cr to l	4
C12H18O	OLBCVFGFOZPWHH-UHFFFAOYSA-N	14.64	292.8	unknown	[5, 6, 7]
C11H15NO3	ISRUGXGCCGIOQO-UHFFFAOYSA-N	23.00	363.6	cr to l	4
C10H14N4O3	KYHQZNGJUGFTGR-UHFFFAOYSA-N	22.75	398.7	unknown	[5, 6, 7]
C6H6O2	GHMLBKRAJCXXBS-UHFFFAOYSA-N	21.30	382.8	cr,I to l	4
C8H5F3N2OS	FTALBRSUTCGOEG-UHFFFAOYSA-N	19.15	393.2	unknown	[5, 6, 7]
C7H5NO3S	CVHZOJJKTDOEJC-UHFFFAOYSA-N	27.40	499.0	cr to l	4
C7H8O2	CQRYARSYNCAZFO-UHFFFAOYSA-N	21.50	358.3	unknown	[5, 6, 7]
C7H7NO2	SKZKKFZAGNVIMN-UHFFFAOYSA-N	26.70	414.0	cr to l	4
C10H18O4	CXMXRPHRNRROMY-UHFFFAOYSA-N	40.81	404.0	cr to l	4
C10H10N4O2S	SEEPANYCNGTZFQ-UHFFFAOYSA-N	31.20	538.8	cr to l	4
C7H12ClN5	ODCWYMIRDDJXKW-UHFFFAOYSA-N	43.90	503.8	cr to l	4
C6H8O2	WSWCOQWTEOXDQX-UHFFFAOYSA-N	18.76	405.3	unknown	[5, 6, 7]
C18H36O2	QIQXTHQIDYTFRH-UHFFFAOYSA-N	56.70	337.2	cr to l	4
C8H10N2O3S	SKIVFJLNDNKQPD-UHFFFAOYSA-N	28.65	454.5	unknown	[5, 6, 7]
C11H12N4O2S	QPPBRPIAZZHUNT-UHFFFAOYSA-N	31.55	515.2	cr to l	4
C11H12N4O3S	GPTONYMQFTZPKC-UHFFFAOYSA-N	34.50	486.7	cr to l	4
C9H10N4O2S2	VACCAVUAMIDAGB-UHFFFAOYSA-N	33.30	482.2	unknown	[5, 6, 7]
C10H11N3O3S	JLKIGFTWXXRPMT-UHFFFAOYSA-N	28.67	439.5	cr to l	4
C11H12N4O3S	VLYWMPOKSSWJAL-UHFFFAOYSA-N	29.56	453.4	unknown	[5, 6, 7]
C6H8N2O2S	FDDDEECHVMSUSB-UHFFFAOYSA-N	23.28	438.0	unknown	[5, 6, 7]
C11H11N3O2S	GECHUMIMRBOMGK-UHFFFAOYSA-N	36.50	466.0	cr to l	4
C9H9N3O2S2	JNMRHUJNCSQMMB-UHFFFAOYSA-N	26.10	474.7	cr,I to l	4
C11H13N3O3S	NHUHCSRWZMLRLA-UHFFFAOYSA-N	29.20	468.2	cr to l	4
C9H16N4OS	HBPDKDSFLXWOAE-UHFFFAOYSA-N	29.50	436.6	cr to l	4
C16H13ClN2O2	SEQDDYPDSLOBDC-UHFFFAOYSA-N	25.63	428.6	unknown	[5, 6, 7]
C13H10N2O4	UEJJHQNACJXSKW-UHFFFAOYSA-N	37.97	548.6	unknown	[5, 6, 7]
C10H7N3S	WJCNZQLZVWNLKY-UHFFFAOYSA-N	36.00	574.8	cr to l	4
C10H12N2	JIVZKJJQOZQXQB-UHFFFAOYSA-N	16.20	337.4	unknown	[5, 6, 7]
C9H14O6	URAYPUMNDPQOKB-UHFFFAOYSA-N	25.80	275.3	cr to l	4
C12H11N7	FNYLWPVRPXGIIP-UHFFFAOYSA-N	73.40	602.2	unknown	[5, 6, 7]
CHBr3	DIKBFYAXUHHXCS-UHFFFAOYSA-N	11.05	281.8	cr to l	4
C12H7Cl3O2	XEFQLINVKFYRCS-UHFFFAOYSA-N	17.75	331.1	unknown	[5, 6, 7]

Continued on next page

Continued from previous page

**Formula**	**InChIKey**	Δh_i^{fus} **KJ/mol**	T_i^{fus}/K	**Phase**	**Source**
C14H12O3	FMHHVULEAZTJMA-UHFFFAOYSA-N	28.40	508.0	cr to l	4
C8H11NO	DZGWFCGJZKJUFP-UHFFFAOYSA-N	28.10	433.6	unknown	[5, 6, 7]
C3H7NO2	JOYRKODLDBILNP-UHFFFAOYSA-N	15.20	321.9	cr to l	4
C8H8N2O3S	UBQNRHZMVUUOMG-UHFFFAOYSA-N	35.78	435.2	unknown	[5, 6, 7]
C7H3Cl3O2	XZIDTOHMJBOSOX-UHFFFAOYSA-N	23.85	402.7	cr to l	4
C7H6N2	HYZJCKYKOHLVJF-UHFFFAOYSA-N	20.81	444.2	unknown	[5, 6, 7]
C6H7NO	CDAWCLOXVUBKRW-UHFFFAOYSA-N	34.00	447.4	cr to l	4
C7H4Cl2O2	CXKCZFDUOYMOOP-UHFFFAOYSA-N	23.00	460.5	cr to l	4
C4H8Cl3O4P	NFACJZMKEDPNKN-UHFFFAOYSA-N	20.40	355.4	cr to l	4
C22H14	LHRCREOYAASXPZ-UHFFFAOYSA-N	31.17	544.2	cr to l	4
C6H5NO2	TWBYWOBDOCUKOW-UHFFFAOYSA-N	135.00	593.0	unknown	[5, 6, 7]
C6H17N3	OTBHHUPVCYLGQO-UHFFFAOYSA-N	34.70	268.2	cr,I to l	4
CCl4	VZGDMQKNWNREIO-UHFFFAOYSA-N	2.65	248.5	cr,I to l	4
C18H12	DXBHBZVCASKNBY-UHFFFAOYSA-N	21.38	434.3	cr to l	4
C2H8N2	RHUYHJGZWVXEHW-UHFFFAOYSA-N	10.07	216.0	cr to l	4
C10H6Cl8	BIWJNBZANLAXMG-UHFFFAOYSA-N	28.50	374.3	cr to l	4
C27H46O	HVYWMOMLDIMFJA-DPAQBDIFSA-N	148.00	298.0	Crystal	[8]
C20H16	MKYQPGPNVYRMHI-UHFFFAOYSA-N	20.47	340.5	unknown	[5, 6, 7]
C12H11N3	QPQKUYVSJWQSDY-UHFFFAOYSA-N	19.90	397.7	unknown	[5, 6, 7]
CH6N2	HDZGCSFEDULWCS-UHFFFAOYSA-N	10.42	220.8	cr to l	4
C12H16N2	DMULVCHRPCFFGV-UHFFFAOYSA-N	17.90	325.2	unknown	[5, 6, 7]
C7H5NO4	OTLNPYWUJOZPPA-UHFFFAOYSA-N	35.90	512.4	cr to l	4
C6H7N	PAYRUJLWNCNPSJ-UHFFFAOYSA-N	10.56	266.9	cr to l	4
C12H11NO2	CVXBEEMKQHEXEN-UHFFFAOYSA-N	24.30	415.5	cr to l	4
C6H12O	JARKCYVAAOWBJS-UHFFFAOYSA-N	13.33	217.0	cr to l	4
C6H4N2O5	MHKBMNACOMRIAW-UHFFFAOYSA-N	26.24	417.0	cr to l	4
C5H8N2	SDXAWLJRERMRKF-UHFFFAOYSA-N	16.66	381.3	unknown	[5, 6, 7]
C4H4N2O3	HNYOPLTXPVRDBG-UHFFFAOYSA-N	20.60	526.3	unknown	[5, 6, 7]
CHCl3	HEDRZPFGACZZDS-UHFFFAOYSA-N	9.44	212.1	cr to l	4
C2H6O2S	HHVIBTZHLRERCL-UHFFFAOYSA-N	18.30	382.0	cr to l	4
C2Cl6	VHHHONWQHHHLTI-UHFFFAOYSA-N	9.75	458.0	cr,I to l	4
C2H3BrO2	KDPAWGWELVVRCH-UHFFFAOYSA-N	13.90	319.2	unknown	[5, 6, 7]
C3H7NO	ZMXDDKWLCZADIW-UHFFFAOYSA-N	7.90	212.7	cr to l	4
C7H9NO2S	LMYRWZFENFIFIT-UHFFFAOYSA-N	25.84	410.3	unknown	[5, 6, 7]
C5H12O	AMQJEAYHLZJPGS-UHFFFAOYSA-N	9.98	194.2	cr to l	4
C2H3Cl3	UOCLXMDMGBRAIB-UHFFFAOYSA-N	4.50	240.8	cr,I to l	4
C7H5ClO2	XRHGYUZYPHTUJZ-UHFFFAOYSA-N	32.26	512.9	cr to l	4
CH3Br	GZUXJHMPEANEGY-UHFFFAOYSA-N	5.98	179.5	cr,I to l	4
C2H6	OTMSDBZUPAUEDD-UHFFFAOYSA-N	0.58	90.3	cr,I to l	4
C2H4	VGGSQFUCUMXWEO-UHFFFAOYSA-N	2.93	103.7	cr to l	4
C2H2	HSFWRNGVRCDJHI-UHFFFAOYSA-N	3.76	192.4	unknown	[5, 6, 7]
CH3Cl	NEHMKBQYUWJMIP-UHFFFAOYSA-N	6.43	175.4	cr to l	4
CH5N	BAVYZALUXZFZLV-UHFFFAOYSA-N	6.13	179.7	cr,I to l	4
C2H5Br	RDHPKYGYEGBMSE-UHFFFAOYSA-N	7.47	154.6	cr to l	4
C3H8	ATUOYWHBWRKTHZ-UHFFFAOYSA-N	3.52	85.5	cr to l	4
C2H5Cl	HRYZWHHZPQKTII-UHFFFAOYSA-N	4.60	133.1	cr to l	4
C2H3Cl	BZHJMEDXRYGGRV-UHFFFAOYSA-N	4.75	119.4	cr to l	4
C2H3N	WEVYAHXRMPXWCK-UHFFFAOYSA-N	8.87	228.3	cr,I to l	4
C2H6S	DNJIEGIFACGWOD-UHFFFAOYSA-N	3.98	125.3	cr to l	4
CH2Cl2	YMWUJEATGCHHMB-UHFFFAOYSA-N	4.60	176.0	cr to l	4
CH2F2	RWRIWBAIICGTTQ-UHFFFAOYSA-N	4.36	136.4	unknown	[5, 6, 7]
CS2	QGJOPFRUJISHPQ-UHFFFAOYSA-N	2.76	162.3	cr to l	4
C3H6	LVZWSLJZHVFIQJ-UHFFFAOYSA-N	5.44	145.6	cr to l	4
C2H4O	IAYPIBMASNFSPL-UHFFFAOYSA-N	5.17	160.7	cr to l	4
C3H7Br	NAMYKGVDVNBCFQ-UHFFFAOYSA-N	6.53	184.2	cr to l	4
C4H10	NNPPMTNAJDCUHE-UHFFFAOYSA-N	4.50	113.2	cr to l	4
C3H7Cl	ULYZAYCEDJDHCC-UHFFFAOYSA-N	7.39	156.0	cr to l	4
C3H9N	JJWLVOIRVHMVIS-UHFFFAOYSA-N	7.33	178.0	cr to l	4

Continued on next page

Continued from previous page

Formula	InChIKey	Δh_i^{fus} KJ/mol	T_i^{fus}/K	Phase	Source
C3H8S	KJRCEJOSASVSRA-UHFFFAOYSA-N	5.75	142.6	cr,I to l	4
C2H4Cl2	SCYULBFZEHDVBN-UHFFFAOYSA-N	5.61	174.0	cr to l	4
C2H2Cl2	LGXVIGDEPROXKC-UHFFFAOYSA-N	6.51	150.6	cr to l	4
C2H4F2	NPNPZTNLOVBDOC-UHFFFAOYSA-N	1.57	154.6	unknown	[5, 6, 7]
CCl2O	YGYAWVDWMABLBF-UHFFFAOYSA-N	5.74	145.4	cr,I to l	4
CHClF2	VOPWNXZWBYDODV-UHFFFAOYSA-N	4.12	115.7	cr,I to l	4
CHF3	XPDWGBQVDMORPB-UHFFFAOYSA-N	4.06	118.0	cr to l	4
CH3NO2	LYGJENNIWJXYER-UHFFFAOYSA-N	9.70	244.8	cr to l	4
C3H6O	GOOHAUXETOMSMM-UHFFFAOYSA-N	6.53	161.2	cr to l	4
CBrCl3	XNNQFQFUQLJSQT-UHFFFAOYSA-N	2.53	267.5	cr to l	4
C4H11N	YBRBMKDOPFTVDT-UHFFFAOYSA-N	1.21	206.2	cr,I to l	4
C4H10O	DKGAVHZHDRPRBM-UHFFFAOYSA-N	6.78	298.5	cr to l	4
C4H10S	WMXCDAVJEZZYLT-UHFFFAOYSA-N	1.65	274.4	cr,I to l	4
C2H3ClF2	BHNZEZWIUMJCGF-UHFFFAOYSA-N	2.69	142.4	cr to l	4
CCl3F	CYRMSUTZVYGINF-UHFFFAOYSA-N	6.89	162.7	cr to l	4
CF4	TXEYQDLBPFQVAA-UHFFFAOYSA-N	0.69	88.4	cr,I to l	4
C2H2Br2Cl2	FIYBYNHDEOSJPL-UHFFFAOYSA-N	5.08	206.3	cr to l	4
C6H14	HNRMPXKDFBEGFZ-UHFFFAOYSA-N	0.46	172.1	cr,I to l	4
C5H12O	KPSSIOMAKSHJJG-UHFFFAOYSA-N	4.06	325.6	cr,I to l	4
C5H12O	MSXVEPNJUHWQHW-UHFFFAOYSA-N	4.46	264.0	cr,I to l	4
C6H12O	PJGSXYOJTGTZAV-UHFFFAOYSA-N	11.33	221.7	cr to l	4
C5H10O2	IUGYQRQAERSCNH-UHFFFAOYSA-N	2.48	307.1	cr,I to l	4
C2HCl5	BNIXVQGCZULYKV-UHFFFAOYSA-N	11.34	243.5	cr to l	4
C2HCl3O2	YNJBWRMUSHSURL-UHFFFAOYSA-N	5.90	332.3	cr to l	4
CCl3NO2	LFHISGNCFUNFFM-UHFFFAOYSA-N	11.54	203.7	cr to l	4
C6H14O2	IVDFJHOHABJVEH-UHFFFAOYSA-N	14.70	316.5	cr to l	4
C2Cl4F2	SLGOCMATMKJJCE-UHFFFAOYSA-N	3.99	314.2	cr to l	4
C2Cl4F2	UGCSPKPEHQEOSR-UHFFFAOYSA-N	3.67	297.9	cr to l	4
C2Cl3F3	AJDIZQLSFPQPEY-UHFFFAOYSA-N	2.47	236.9	cr,I to l	4
C2Cl2F4	DDMOUSALMHHKOS-UHFFFAOYSA-N	1.51	180.6	cr,I to l	4
C2ClF5	RFCAUADVODFSLZ-UHFFFAOYSA-N	1.88	173.7	cr,I to l	4
C2F6	WMIYKQLTONQJES-UHFFFAOYSA-N	2.69	173.1	cr,I to l	4
C3F8	QYSGYZVSCZSLHT-UHFFFAOYSA-N	0.48	125.5	cr,I to l	4
C4H9NO3	MVGJRISPEUZYAQ-UHFFFAOYSA-N	3.74	361.6	cr,I to l	4
C5H8N2O2	YIROYDNZEPTFOL-UHFFFAOYSA-N	22.20	448.9	unknown	[5, 6, 7]
C10H12	HECLRDQVFMWTQS-UHFFFAOYSA-N	2.22	304.8	cr,I to l	4
C4H6O2S	MBDNRNMVTZADMQ-UHFFFAOYSA-N	14.20	337.2	unknown	[5, 6, 7]
C5H12O3	QXJQHYBHAIHNGG-UHFFFAOYSA-N	5.38	470.1	cr,I to l	4
C4H11NO3	LENZDBCJOHFCAS-UHFFFAOYSA-N	3.04	440.6	cr,I to l	4
C6H14O3	ZJCCRDAZUWHFQH-UHFFFAOYSA-N	21.45	333.4	cr to l	4
C3H6O2S	DKIDEFUBRARXTE-UHFFFAOYSA-N	16.97	291.9	unknown	[5, 6, 7]
C5H8Cl2O	CXURGFRDGROIKG-UHFFFAOYSA-N	16.94	292.2	cr,I to l	4
C3H6Br2	XFNJYAKDBJUJAJ-UHFFFAOYSA-N	8.94	217.7	cr to l	4
C4H9Br	UPSXAPQYNGXVBF-UHFFFAOYSA-N	6.89	160.5	cr to l	4
C4H9Br	HLVFKOKELQSXIQ-UHFFFAOYSA-N	2.52	155.8	cr to l	4
C5H12	QWTDNUCVQCZILF-UHFFFAOYSA-N	5.11	112.6	cr to l	4
C5H8	RRHGJUQNOFWUDK-UHFFFAOYSA-N	4.83	126.4	cr to l	4
C4H10O	ZXEKIIBDNHEJCQ-UHFFFAOYSA-N	6.07	169.0	cr to l	4
C3H6Cl2	KNKRKFALVUDBJE-UHFFFAOYSA-N	6.40	172.6	cr to l	4
C3H10N2	AOHJOMMDDJHIJH-UHFFFAOYSA-N	18.42	236.5	cr,I to l	4
C4H10O	BTANRVKWQNVYAZ-UHFFFAOYSA-N	5.97	184.7	cr to l	4
C4H8O	ZWEHNKRNPOVVGH-UHFFFAOYSA-N	8.48	186.1	cr to l	4
C2H3Cl3	UBOXGVDOUJQMTN-UHFFFAOYSA-N	11.54	236.6	cr to l	4
C2HCl3	XSTXAVWGXDQKEL-UHFFFAOYSA-N	8.45	188.5	cr to l	4
C3H7NO	QLNJFJADRCOGBJ-UHFFFAOYSA-N	12.90	352.6	unknown	[5, 6, 7]
C3H5NO	HRPVXLWXLXDGHG-UHFFFAOYSA-N	15.33	358.0	unknown	[5, 6, 7]
C3H4O2	NIXOWILDQLNWCW-UHFFFAOYSA-N	11.20	286.1	cr to l	4
C3H7NO	OHLUUHNLEMFGTQ-UHFFFAOYSA-N	9.40	303.8	unknown	[5, 6, 7]

Continued on next page

Continued from previous page

**Formula**	**InChIKey**	Δh_i^{fus} **KJ/mol**	T_i^{fus}/K	**Phase**	**Source**
C3H6O2	KXKVLQRXCPHEJC-UHFFFAOYSA-N	7.49	174.9	cr,I to l	4
C2H5NO2	MCSAJNNLRCFZED-UHFFFAOYSA-N	9.85	183.7	cr to l	4
C6H14	ZFFMLCVRJBZUDZ-UHFFFAOYSA-N	0.81	145.2	cr,I to l	4
C2H2Cl4	QPFMBZIOSGYJDE-UHFFFAOYSA-N	9.17	230.8	cr,I to l	4
C2ClF3	UUAGAQFQZIEFAH-UHFFFAOYSA-N	5.55	115.0	cr to l	4
C4H7NO	FQPSGWSUVKBHSU-UHFFFAOYSA-N	15.00	385.1	unknown	[5, 6, 7]
C2H2Cl2O2	JXTHNDFMNIQAHM-UHFFFAOYSA-N	7.64	284.0	cr to l	4
C10H16	CRPUJAZIXJMDBK-UHFFFAOYSA-N	31.80	324.1	cr to l	4
C15H16O2	IISBACLAFKSPIT-UHFFFAOYSA-N	30.10	433.0	cr to l	4
C12H8Cl2O2S	GPAPPPVRLPGFEQ-UHFFFAOYSA-N	24.40	422.0	cr to l	4
C12H10O4S	VPWNQTHUCYMVMZ-UHFFFAOYSA-N	41.40	519.9	unknown	[5, 6, 7]
C18H22O2	XMNIXWIUMCBBBL-UHFFFAOYSA-N	28.51	312.5	unknown	[5, 6, 7]
C5H8O2	VVQNEPGJFQJSBK-UHFFFAOYSA-N	14.44	225.6	cr to l	4
C14H18N2O5	WXCMHFPAUCOJIG-UHFFFAOYSA-N	23.81	409.0	unknown	[5, 6, 7]
C14H8O4	GUEIZVNYDFNHJU-UHFFFAOYSA-N	24.20	473.0	cr to l	4
C12H6O3	GRSMWKLPSNHDHA-UHFFFAOYSA-N	23.30	545.7	cr to l	4
C17H10O	HUKPVYBUJRAUAG-UHFFFAOYSA-N	27.80	446.3	cr to l	4
C15H11NO2	SVTDYSXXLJYUTM-UHFFFAOYSA-N	28.81	443.2	unknown	[5, 6, 7]
C14H7ClO2	BOCJQSFSGAZAPQ-UHFFFAOYSA-N	29.24	435.2	unknown	[5, 6, 7]
C14H9NO2	KHUFHLFHOQVFGB-UHFFFAOYSA-N	27.54	524.5	unknown	[5, 6, 7]
C6Cl5NO2	LKPLKUMXSAEKID-UHFFFAOYSA-N	18.41	418.0	cr to l	4
C6H3N3O8	IXHMHWIBCIYOAZ-UHFFFAOYSA-N	33.50	454.9	cr to l	4
C14H14O3	RZKYEQDPDZUERB-UHFFFAOYSA-N	31.00	382.7	cr to l	4
C12H10	CWRYPZZKDGJXCA-UHFFFAOYSA-N	20.70	366.5	cr to l	4
C9H8O	QNXSIUBBGPHDDE-UHFFFAOYSA-N	17.69	313.5	unknown	[5, 6, 7]
C9H9N	ZFRKQXVRDFCRJG-UHFFFAOYSA-N	90.40	298.2	Crystal	[8]
C18H14	OIAQMFOKAXHPNH-UHFFFAOYSA-N	17.19	329.4	cr to l	4
C16H12O2	SJEBAWHUJDUKQK-UHFFFAOYSA-N	18.77	382.9	unknown	[5, 6, 7]
C14H8O2	RZVHIXYEVGDQDX-UHFFFAOYSA-N	32.57	555.0	cr to l	4
C12H14O4	FLKPEMZONWLCSK-UHFFFAOYSA-N	17.98	269.9	cr to l	4
C28H46O4	PGIBJVOPLXHHGS-UHFFFAOYSA-N	45.00	277.7	unknown	[5, 6, 7]
C8H5Cl3O2	QZXCCPZJCKEPSA-UHFFFAOYSA-N	22.40	433.5	cr to l	4
C8H5NO2	XKJCHHZQLQNZHY-UHFFFAOYSA-N	28.60	507.2	unknown	[5, 6, 7]
C8H4O3	LGRFSURHDFAFJT-UHFFFAOYSA-N	23.10	404.4	cr to l	4
C14H10O3	FGTYTUFKXYPTML-UHFFFAOYSA-N	26.90	402.3	cr to l	4
C10H7NO2	RJKGJBPXVHTNJL-UHFFFAOYSA-N	18.43	329.1	cr to l	4
C13H10	NIHNNTQXNPWCJQ-UHFFFAOYSA-N	19.87	387.0	cr to l	4
C12H9N	UJOBWOGCFQCDNV-UHFFFAOYSA-N	29.45	509.1	cr,I to l	4
C10H12O4	OPHQOIGEOHXOGX-UHFFFAOYSA-N	23.60	347.7	unknown	[5, 6, 7]
C12H11NO	XFNJVKMNNVCYEK-UHFFFAOYSA-N	32.80	457.1	cr to l	4
C12H10O2	PRPINYUDVPFIRX-UHFFFAOYSA-N	22.30	406.0	cr to l	4
C13H11NO2	WKEDVNSFRWHDNR-UHFFFAOYSA-N	45.08	413.2	unknown	[5, 6, 7]
C9H11NO2	TWLLPUMZVVGILS-UHFFFAOYSA-N	22.10	287.5	cr to l	4
C6H3Cl3	RELMFMZEBKVZJC-UHFFFAOYSA-N	20.50	326.9	cr to l	4
C6H4Cl2O	HOLHYSJJBXSLMV-UHFFFAOYSA-N	22.14	340.0	cr to l	4
C7H3Br5	OZHJEQVYCBTHJT-UHFFFAOYSA-N	29.30	559.0	unknown	[5, 6, 7]
C12H18	YUWFEBAXEOLKSG-UHFFFAOYSA-N	20.63	438.6	cr,I to l	4
C6H9NO	WHNWPMSKXPGLAX-UHFFFAOYSA-N	15.28	286.2	unknown	[5, 6, 7]
C6H6OS	WYJOVVXUZNRJQY-UHFFFAOYSA-N	15.00	283.6	cr to l	4
C7H9NO2S	YCMLQMDWSXFTIF-UHFFFAOYSA-N	29.43	427.4	unknown	[5, 6, 7]
C11H16O	XOUQAVYLRNOXDO-UHFFFAOYSA-N	14.85	296.1	unknown	[5, 6, 7]
C7H5BrO2	XRXMNWGCKISMOH-UHFFFAOYSA-N	24.67	422.0	unknown	[5, 6, 7]
C7H8N2O	PXBFMLJZNCDSMP-UHFFFAOYSA-N	21.40	384.0	unknown	[5, 6, 7]
C9H12O	CRBJBYGJVIBWIY-UHFFFAOYSA-N	12.49	290.0	cr to l	4
C6H4ClNO2	BFCFYVKQTRLZHA-UHFFFAOYSA-N	17.90	305.3	cr to l	4
C6H6N2O2	DPJCXCZTLWNFOH-UHFFFAOYSA-N	16.11	342.5	cr to l	4
C6H5NO3	IQUPABOKLQSFBK-UHFFFAOYSA-N	15.60	316.0	cr to l	4
C10H12N2O5	OWZPCEFYPSAJFR-UHFFFAOYSA-N	21.80	316.4	cr to l	4

Continued on next page

Continued from previous page

Formula	InChIKey	Δh_i^{fus} KJ/mol	T_i^{fus}/K	Phase	Source
C6H3N3O7	OXNIZHLAWKMVMX-UHFFFAOYSA-N	17.10	394.1	cr to l	4
C10H2O6	ANSXAPJVJOKRDJ-UHFFFAOYSA-N	32.39	558.9	cr,I to l	4
C7H8N2O2	DLURHXYXQYMPLT-UHFFFAOYSA-N	21.15	388.6	unknown	[5, 6, 7]
C6H5ClN2O2	PBGKNXWGYQPUJK-UHFFFAOYSA-N	22.28	388.9	unknown	[5, 6, 7]
C6H4ClNO3	NWSIFTLPLKCTSX-UHFFFAOYSA-N	22.69	360.3	unknown	[5, 6, 7]
C10H14O	MGSRCZKZVOBKFT-UHFFFAOYSA-N	17.30	321.7	cr to l	4
C7H5ClO	FPYUJUBAXZAQNL-UHFFFAOYSA-N	13.43	285.3	cr to l	4
C7H6O2	SMQUZDBALVYZAC-UHFFFAOYSA-N	13.30	278.7	unknown	[5, 6, 7]
C10H7Br	DLKQHBOKULLWDQ-UHFFFAOYSA-N	15.16	275.0	cr to l	4
C11H10	QPUYECUOLPXSFR-UHFFFAOYSA-N	9.62	242.7	cr,I to l	4
C10H7Cl	JTPNRXUCIXHOKM-UHFFFAOYSA-N	12.90	270.7	cr to l	4
C10H8O	KJCVRFUGPWSIIH-UHFFFAOYSA-N	23.47	368.2	cr to l	4
C10H9Cl3O2	JKRWZLOCPLZZEI-UHFFFAOYSA-N	30.23	360.6	unknown	[5, 6, 7]
C12H11N	TWBPWBPGNQWFSJ-UHFFFAOYSA-N	13.99	322.3	cr,I to l	4
C12H20O	UOBQDYFTAJKQAL-UHFFFAOYSA-N	18.00	277.0	cr to l	4
C12H10O	LLEMOWNGBBNAJR-UHFFFAOYSA-N	16.21	330.6	cr to l	4
C14H10O	RJGDLRCDCYRQOQ-UHFFFAOYSA-N	24.60	429.3	unknown	[5, 6, 7]
C13H8O2	JNELGWHKGNBSMD-UHFFFAOYSA-N	26.12	449.6	cr to l	4
C13H8Cl2O	OKISUZLXOYGIFP-UHFFFAOYSA-N	30.10	420.0	cr,I to l	4
C13H12O	QILSFLSDHQAZET-UHFFFAOYSA-N	23.00	338.5	cr to l	4
C12H9NO	GCSHUYKULREZSJ-UHFFFAOYSA-N	20.91	316.5	unknown	[5, 6, 7]
C8H10O3	KLIDCXVFHGNTTM-UHFFFAOYSA-N	50.30	326.4	unknown	[5, 6, 7]
C8H4N2	XQZYPMVTSDWCCE-UHFFFAOYSA-N	21.30	413.1	cr to l	4
C8H10O2	ABDKAPXRBAPSQN-UHFFFAOYSA-N	15.87	295.9	cr to l	4
C10H18	NNBZCPXTIHJBJL-UHFFFAOYSA-N	14.41	242.4	cr to l	4
C9H7N	SMWDFEZZVXVKRB-UHFFFAOYSA-N	10.80	258.5	cr,I to l	4
C8H5NO2	JXDYKVIHCLTXOP-UHFFFAOYSA-N	27.82	475.7	unknown	[5, 6, 7]
C11H10	QIMMUPPBPVKWKM-UHFFFAOYSA-N	11.97	307.2	cr,I to l	4
C10H7Cl	CGYGETOMCSJHJU-UHFFFAOYSA-N	14.70	332.0	cr to l	4
C10H9N	JBIJLHTVPXGSAM-UHFFFAOYSA-N	22.00	382.7	cr to l	4
C10H9N	SMUQFGGVLNAIOZ-UHFFFAOYSA-N	12.52	270.5	unknown	[5, 6, 7]
C18H14	YJTKZCDBKVTVBY-UHFFFAOYSA-N	26.36	361.0	unknown	[5, 6, 7]
C12H22	WVIIMZNLDWSIRH-UHFFFAOYSA-N	10.70	276.8	cr,I to l	4
C12H10	ZUOUZKKEUPVFJK-UHFFFAOYSA-N	18.90	314.3	cr,I to l	4
C12H9Br	PKJBWOWQJHHAHG-UHFFFAOYSA-N	16.30	362.1	unknown	[5, 6, 7]
C12H10O	YXVFYQXJAXKLAK-UHFFFAOYSA-N	31.55	441.1	unknown	[5, 6, 7]
C11H8O3	ALKYHXVLJMQRLQ-UHFFFAOYSA-N	27.70	494.4	unknown	[5, 6, 7]
C15H11NO	CNRNYORZJGVOSY-UHFFFAOYSA-N	22.90	344.2	unknown	[5, 6, 7]
C13H10O	GJCOSYZMQJWQCA-UHFFFAOYSA-N	19.20	373.7	cr to l	4
C12H9NS	WJFKNYWRSNBZNX-UHFFFAOYSA-N	30.50	457.5	cr to l	4
C12H8S2	GVIJJXMXTUZIOD-UHFFFAOYSA-N	25.44	428.4	cr to l	4
C12H8Br2	HQJQYILBCQPYBI-UHFFFAOYSA-N	28.38	440.7	unknown	[5, 6, 7]
C12H12N2	HFACYLZERDEVSX-UHFFFAOYSA-N	19.10	400.1	cr to l	4
C12H10O2	VCCBEIPGXKNHFW-UHFFFAOYSA-N	43.05	560.7	unknown	[5, 6, 7]
C18H14	XJKSTNDFUHDPQJ-UHFFFAOYSA-N	35.50	486.3	cr to l	4
C9H10O4	DAUAQNGYDSHRET-UHFFFAOYSA-N	32.10	453.3	unknown	[5, 6, 7]
C11H8O2	UOBYKYZJUGYBDK-UHFFFAOYSA-N	24.50	458.8	cr to l	4
C8H8O2	QPJVMBTYPHYUOC-UHFFFAOYSA-N	9.74	260.8	cr to l	4
C7H7NO2	YNBADRVTZLEFNH-UHFFFAOYSA-N	19.30	312.6	unknown	[5, 6, 7]
C9H7Cl3O3	ZLSWBLPERHFHIS-UHFFFAOYSA-N	39.60	453.8	cr to l	4
C14H10O3	CHIHQLCVLOXUJW-UHFFFAOYSA-N	17.15	313.2	unknown	[5, 6, 7]
C13H11NO	ZVSKZLHKADLHSD-UHFFFAOYSA-N	32.08	436.0	unknown	[5, 6, 7]
C10H12O3	QELSKZZBTMNZEB-UHFFFAOYSA-N	28.00	369.2	cr to l	4
C11H14O3	QFOHBWFCKVYLES-UHFFFAOYSA-N	26.69	341.5	unknown	[5, 6, 7]
C14H10O4	OMPJBNCRMGITSC-UHFFFAOYSA-N	22.59	378.0	unknown	[5, 6, 7]
C8H6O4	VDVJGIYXDVPQLP-UHFFFAOYSA-N	30.50	501.6	unknown	[5, 6, 7]
C9H9ClO3	WHKUVVPPKQRRBV-UHFFFAOYSA-N	31.40	392.7	cr to l	4
C11H13ClO3	LLWADFLAOKUBDR-UHFFFAOYSA-N	34.30	373.5	cr to l	4

Continued on next page

Continued from previous page

Formula	InChIKey	Δh_i^{fus} KJ/mol	T_i^{fus}/K	Phase	Source
C9H8	YBYIRNPNPLQARY-UHFFFAOYSA-N	10.20	271.7	cr to l	4
C6H5N3	QRUDEWIWKLJBPS-UHFFFAOYSA-N	11.46	369.6	unknown	[5, 6, 7]
C8H6S	FCEHBMOGCRZNNI-UHFFFAOYSA-N	11.80	304.5	cr to l	4
C7H5NS	IOJUPLGTWVMSFF-UHFFFAOYSA-N	13.30	276.1	cr,I to l	4
C9H9N	BHNHHSOHWZKFOX-UHFFFAOYSA-N	15.07	337.5	unknown	[5, 6, 7]
C8H7NO	DQSHFKPKFISSNM-UHFFFAOYSA-N	11.50	280.1	unknown	[5, 6, 7]
C11H14N2S2	IUJLOAKJZQBENM-UHFFFAOYSA-N	26.84	382.5	unknown	[5, 6, 7]
C7H7Br	QSSXJPIWXQTSIX-UHFFFAOYSA-N	10.14	245.4	cr to l	4
C8H10	CTQNGGLPUBDAKN-UHFFFAOYSA-N	13.03	247.8	cr to l	4
C7H7Cl	IBSQPLPBRSHTTG-UHFFFAOYSA-N	8.40	236.7	cr to l	4
C6H4Cl2	RFFLAFLAYFXFSW-UHFFFAOYSA-N	12.92	255.7	cr to l	4
C6H6ClN	AKCRQHGQIJBRMN-UHFFFAOYSA-N	11.88	271.1	cr to l	4
C7H9N	RNVCVTLRINQCPJ-UHFFFAOYSA-N	7.54	249.5	cr to l	4
C6H8N2	GEYOCULIXLDCMW-UHFFFAOYSA-N	23.10	373.9	cr to l	4
C6H5ClO	ISPYQTSUDJAMAB-UHFFFAOYSA-N	13.50	282.2	cr to l	4
C9H12	GWHJZXXIDMPWGX-UHFFFAOYSA-N	12.65	228.6	cr,I to l	4
C8H10O	YCOXTKKNXUZSKD-UHFFFAOYSA-N	18.13	334.0	cr to l	4
C7H8O2	CNHDIAIOKMXOLK-UHFFFAOYSA-N	27.60	404.2	unknown	[5, 6, 7]
C6H5Cl2N	SDYWXFYBZPNOFX-UHFFFAOYSA-N	21.69	344.5	unknown	[5, 6, 7]
C6H4Cl2O	WDNBURPWRNALGP-UHFFFAOYSA-N	20.93	341.0	cr to l	4
C8H10O	NKTOLZVEWDHZMU-UHFFFAOYSA-N	23.38	348.0	cr to l	4
C10H14	SQNZJJAZBFDUTD-UHFFFAOYSA-N	21.01	352.4	cr to l	4
C6H2Cl4	JHBKHLUZVFWLAG-UHFFFAOYSA-N	24.10	412.2	cr,I to l	4
C6H3Cl3O	LHJGJYXLEPZJPM-UHFFFAOYSA-N	23.85	341.7	cr to l	4
C6H8O4	JJTUDXZGHPGLLC-UHFFFAOYSA-N	19.28	378.2	unknown	[5, 6, 7]
C3H5Br3	FHCLGDLYRUPKAM-UHFFFAOYSA-N	23.78	289.3	cr to l	4
C6H14	PFEOZHBOMNWTJB-UHFFFAOYSA-N	5.30	110.3	cr to l	4
C5H10O	FDPIMTJIUBPUKL-UHFFFAOYSA-N	11.59	234.2	cr,I to l	4
C3H8N2O	MGJKQDOBUOMPEZ-UHFFFAOYSA-N	12.64	379.9	cr to l	4
C4H6O2	BAPJBEWLBFYGME-UHFFFAOYSA-N	9.73	197.5	unknown	[5, 6, 7]
C3H5ClO2	QABLOFMHHSOFRJ-UHFFFAOYSA-N	11.29	241.0	cr to l	4
C6H12	GDOPTJXRTPNYNR-UHFFFAOYSA-N	6.88	130.1	cr to l	4
C5H10O	XCIXKGXIYUWCLL-UHFFFAOYSA-N	1.54	257.4	cr,I to l	4
C4H6O2	YEJRWHAVMIAJKC-UHFFFAOYSA-N	9.57	229.8	cr to l	4
C3H4O3	KMTRUDSVKNLOMY-UHFFFAOYSA-N	13.30	309.5	cr to l	4
C5H7N	OXHNLMTVIGZXSG-UHFFFAOYSA-N	7.83	216.9	cr to l	4
C7H8N2O3	GVBHRNIWBGTNQA-UHFFFAOYSA-N	27.80	412.9	unknown	[5, 6, 7]
C8H14O2	SOGAXMICEFXMKE-UHFFFAOYSA-N	13.95	196.8	cr to l	4
C5H6O2	XPFVYQJUAUNWIW-UHFFFAOYSA-N	14.81	253.5	cr to l	4
C5H4O2	HYBBIBNJHNGZAN-UHFFFAOYSA-N	14.37	235.1	cr to l	4
C7H5Cl3	XEMRAKSQROQPBR-UHFFFAOYSA-N	13.95	236.0	cr to l	4
C7H5F3	GETTZEONDQJALK-UHFFFAOYSA-N	13.47	244.0	cr to l	4
C6H7NO2S	KHBQMWCZKVMBLN-UHFFFAOYSA-N	25.17	425.8	unknown	[5, 6, 7]
C10H14O2	XESZUVZBAMCAEJ-UHFFFAOYSA-N	9.80	328.7	unknown	[5, 6, 7]
C10H14O	QHPQWRBYOIRBIT-UHFFFAOYSA-N	14.52	373.2	unknown	[5, 6, 7]
C11H14O2	KDVYCTOWXSLNNI-UHFFFAOYSA-N	17.90	436.7	cr to l	4
C9H12	RWGFKTVRMDUZSP-UHFFFAOYSA-N	7.33	177.1	cr to l	4
C9H10	XYLMUPLGERFSHI-UHFFFAOYSA-N	10.66	249.9	cr to l	4
C8H8O	KWOLFJPFCHCOCG-UHFFFAOYSA-N	16.65	292.7	unknown	[5, 6, 7]
C7H5ClO	PASDCCFISLVPSO-UHFFFAOYSA-N	19.20	272.8	cr to l	4
C7H12O2	NZNMSOFKMUBTKW-UHFFFAOYSA-N	9.20	301.9	unknown	[5, 6, 7]
C6H5NO2	LQNUZADURLCDLV-UHFFFAOYSA-N	12.12	278.8	cr to l	4
C8H9NO	CKQHAYFOPRIUOM-UHFFFAOYSA-N	12.13	371.2	unknown	[5, 6, 7]
C8H8O2	GPSDUZXPYCFOSQ-UHFFFAOYSA-N	15.73	381.9	cr to l	4
C7H7NO2	XFDUHJPVQKIXHO-UHFFFAOYSA-N	21.84	452.9	cr to l	4
C7H6O3	IJFXRHURBJZNAO-UHFFFAOYSA-N	55.40	472.0	cr to l	4
C6H6N2O2	XJCVRTZCHMZPBD-UHFFFAOYSA-N	23.68	384.9	cr to l	4
C7H6O4	UYEMGAFJOZZIFP-UHFFFAOYSA-N	37.87	510.1	unknown	[5, 6, 7]

Continued on next page

Continued from previous page

Formula	InChIKey	Δh_i^{fus} KJ/mol	T_i^{fus}/K	Phase	Source
C8H8O5	FBSFWRHWHYMIOG-UHFFFAOYSA-N	35.80	477.1	unknown	[5, 6, 7]
C6H4Cl2N2O2	BIXZHMJUSMUDOQ-UHFFFAOYSA-N	32.60	466.8	cr to l	4
C7H4N2O6	VYWYYJYRVSBHJQ-UHFFFAOYSA-N	29.61	480.4	unknown	[5, 6, 7]
C6H3N3O6	UATJOMSPNYCXIX-UHFFFAOYSA-N	15.00	398.4	cr,I to l	4
C9H10O2	CPXCDEMFNPKOEF-UHFFFAOYSA-N	19.15	270.3	unknown	[5, 6, 7]
C6H3Cl2NO2	NTBYINQTYWZXLH-UHFFFAOYSA-N	17.77	315.2	unknown	[5, 6, 7]
C7H5NO3	ZETIVVHRRQLWFW-UHFFFAOYSA-N	21.01	328.9	unknown	[5, 6, 7]
C6H4N2O4	WDCYWAQPCXBPJA-UHFFFAOYSA-N	20.40	363.1	cr to l	4
C9H10O2	QSSJZLPUHJDYKF-UHFFFAOYSA-N	20.58	306.3	unknown	[5, 6, 7]
C8H8O3	LXCFILQKKLGQFO-UHFFFAOYSA-N	24.30	398.4	cr to l	4
C10H14	HFPZCAJZSCWRBC-UHFFFAOYSA-N	9.66	204.2	cr to l	4
C9H12O	YQUQWHNMBPIWGK-UHFFFAOYSA-N	13.60	336.2	cr to l	4
C8H7ClO	BUZYGTVTZYSBCU-UHFFFAOYSA-N	15.30	290.3	unknown	[5, 6, 7]
C8H9NO	GPRYKVSEZCQIHD-UHFFFAOYSA-N	18.01	379.2	unknown	[5, 6, 7]
C8H8O2	TXFPEBPIARQUIG-UHFFFAOYSA-N	18.07	381.0	unknown	[5, 6, 7]
C8H8O2	LPNBBFKOUUSUDB-UHFFFAOYSA-N	22.72	452.8	cr to l	4
C7H7NO2	ZPTVNYMJQHSSEA-UHFFFAOYSA-N	13.31	324.8	cr to l	4
C6H4ClNO2	CZGCEKJOLUNIFY-UHFFFAOYSA-N	14.11	355.1	cr to l	4
C6H6N2O2	TYMLOMAKGOJONV-UHFFFAOYSA-N	21.09	420.7	cr to l	4
C9H10O2	NTPLXRHDUXRPNE-UHFFFAOYSA-N	16.80	310.3	unknown	[5, 6, 7]
C8H8O3	ZEYHEAKUIGZSGI-UHFFFAOYSA-N	28.40	457.8	cr to l	4
C9H11NO	BGNGWHSBYQYVRX-UHFFFAOYSA-N	19.70	346.5	unknown	[5, 6, 7]
C8H7NO3	YQYGPGKTNQNXMH-UHFFFAOYSA-N	23.93	353.8	unknown	[5, 6, 7]
C8H4Cl2O2	LXEJRKJRKIFVNY-UHFFFAOYSA-N	21.10	356.1	unknown	[5, 6, 7]
C8H6O4	KKEYFWRCBNTPAC-UHFFFAOYSA-N	53.57	698.2	unknown	[5, 6, 7]
C6H4N2O4	FYFDQJRXFWGIBS-UHFFFAOYSA-N	28.12	446.7	cr to l	4
C7H7Br	AGEZXYOZHKGVCM-UHFFFAOYSA-N	13.70	271.8	cr to l	4
C8H10	YNQLUTRBYVCPMQ-UHFFFAOYSA-N	9.16	178.0	cr to l	4
C8H8	PPBRXRYQALVLMV-UHFFFAOYSA-N	10.95	242.5	cr to l	4
C7H7Cl	KCXMKQUNVWSEMD-UHFFFAOYSA-N	8.74	230.0	unknown	[5, 6, 7]
C7H5N	JFDZBHWFFUWGJE-UHFFFAOYSA-N	9.10	259.2	cr to l	4
C6H4N2	GPHQHTOMRSGBNZ-UHFFFAOYSA-N	18.09	353.6	unknown	[5, 6, 7]
C6H8N2	HKOOXMFOFWEVGF-UHFFFAOYSA-N	16.40	295.3	cr to l	4
C6H11NO	VEZUQRBDRNJBJY-UHFFFAOYSA-N	12.70	362.5	cr,I to l	4
C7H8O	RDOXTESZEPMUJZ-UHFFFAOYSA-N	17.00	293.2	cr to l	4
C7H8S	HNKJADCVZUBCPG-UHFFFAOYSA-N	14.84	256.4	cr to l	4
C9H5Cl3N4	IMHBYKMAHXWHRP-UHFFFAOYSA-N	31.50	432.5	cr to l	4
C9H9ClO3	YNTJKQDWYXUTLZ-UHFFFAOYSA-N	33.05	386.0	unknown	[5, 6, 7]
C13H9Cl3N2O	ICUTUKXCWQYESQ-UHFFFAOYSA-N	41.94	528.2	unknown	[5, 6, 7]
C9H12N2O	XXOYNJXVWVNOOJ-UHFFFAOYSA-N	24.30	406.0	cr to l	4
C12H9BrO	JDUYPUMQALQRCN-UHFFFAOYSA-N	15.64	291.9	cr to l	4
C15H10N2O2	UPMLOUAZCHDJJD-UHFFFAOYSA-N	27.30	313.6	cr to l	4
C13H14N2	YBRVSVVVWCFQMG-UHFFFAOYSA-N	9.20	363.7	cr to l	4
C12H12N2O	HLBLWEWZXPIGSM-UHFFFAOYSA-N	7.74	465.4	cr to l	4
C13H12	CZZYITDELCSZES-UHFFFAOYSA-N	17.70	299.2	cr to l	4
C12H10O	USIUVYZYUHIAEV-UHFFFAOYSA-N	17.22	300.0	cr to l	4
C10H12O2	DULCUDSUACXJJC-UHFFFAOYSA-N	18.00	244.0	unknown	[5, 6, 7]
C9H11NO2	LBKPGNUOUPTQKA-UHFFFAOYSA-N	16.27	326.0	cr to l	4
C13H12N2O	GWEHVDNNLFDJLR-UHFFFAOYSA-N	34.60	512.1	cr to l	4
C13H10O3	ROORDVPLFPIABK-UHFFFAOYSA-N	23.87	355.5	unknown	[5, 6, 7]
C5H6	ZSWFCLXCOIISFI-UHFFFAOYSA-N	8.01	176.6	cr to l	4
C8H11NO2	NAZDVUBIEPVUKE-UHFFFAOYSA-N	23.20	352.6	unknown	[5, 6, 7]
C11H12N2OS2	MHKLKWCYGIBEQF-UHFFFAOYSA-N	22.60	360.1	cr to l	4
C13H12O2	VYQNWZOUAUKGHI-UHFFFAOYSA-N	39.33	392.4	unknown	[5, 6, 7]
C13H10Cl2S	ZHLKXBJTJHRTTE-UHFFFAOYSA-N	32.20	343.8	cr to l	4
C14H14	QWUWMCYKGHVNAV-UHFFFAOYSA-N	23.65	324.1	cr,I to l	4
C9H12	ODLMAHJVESYWTB-UHFFFAOYSA-N	9.27	173.6	cr,I to l	4
C8H9NO	LSBDFXRDZJMBSC-UHFFFAOYSA-N	27.20	431.4	unknown	[5, 6, 7]

Continued on next page

Continued from previous page

Formula	InChIKey	Δh_i^{fus} KJ/mol	T_i^{fus}/K	Phase	Source
C9H10O3	NRPFNQUDKRYCNX-UHFFFAOYSA-N	21.80	358.1	cr to l	4
C9H11ClO3	MXOAEAUPQDYUQM-UHFFFAOYSA-N	40.95	351.3	unknown	[5, 6, 7]
C10H12O	RUVINXPYWBROJD-UHFFFAOYSA-N	16.00	294.7	cr to l	4
C10H14	OCKPCBLVNKHBMX-UHFFFAOYSA-N	10.98	184.6	cr to l	4
C16H26	UZILCZKGXMQEQR-UHFFFAOYSA-N	30.84	259.7	unknown	[5, 6, 7]
C8H8O	FXLOVSHXALFLKQ-UHFFFAOYSA-N	11.00	263.1	unknown	[5, 6, 7]
C4H5NO2	ANGDWNBGPBMQHW-UHFFFAOYSA-N	12.33	260.0	cr to l	4
C5H10O2	FKRCODPIKNYEAC-UHFFFAOYSA-N	12.29	199.8	unknown	[5, 6, 7]
C6H12O2	OBNCKNCVKJNDBV-UHFFFAOYSA-N	10.68	174.5	unknown	[5, 6, 7]
C5H7NO2	ZIUSEGSNTOUIPT-UHFFFAOYSA-N	11.78	246.8	cr to l	4
C6H14O2	DHKHKXVYLBGOIT-UHFFFAOYSA-N	10.95	167.0	unknown	[5, 6, 7]
C5H10O3	OIFBSDVPJOWBCH-UHFFFAOYSA-N	9.17	197.3	unknown	[5, 6, 7]
C6H11NO	JBKVHLHDHHXQEQ-UHFFFAOYSA-N	16.13	342.4	cr to l	4
C8H10O	KUFFULVDNCHOFZ-UHFFFAOYSA-N	12.76	297.3	unknown	[5, 6, 7]
C12H20O4	JBSLOWBPDRZSMB-UHFFFAOYSA-N	27.60	255.3	cr to l	4
C14H28O2	MMXKVMNBHPAILY-UHFFFAOYSA-N	31.17	272.3	unknown	[5, 6, 7]
C7H14O	NGAZZOYFWWSOGK-UHFFFAOYSA-N	17.53	236.0	unknown	[5, 6, 7]
C6H4Br2	SWJPEBQEEAHIGZ-UHFFFAOYSA-N	20.33	358.0	cr,I to l	4
C7H7Br	ZBTMRBYMKUEVEU-UHFFFAOYSA-N	14.93	300.8	cr to l	4
C6H4BrCl	NHDODQWIKUYWMW-UHFFFAOYSA-N	18.76	337.8	cr to l	4
C6H6BrN	WDFQBORIUYODSI-UHFFFAOYSA-N	15.06	336.0	unknown	[5, 6, 7]
C6H5BrO	GZFGOTFRPZRKDS-UHFFFAOYSA-N	13.00	337.0	cr to l	4
C8H10	URLKBWYHVLBVBO-UHFFFAOYSA-N	16.93	286.3	cr to l	4
C7H7Cl	NPDACUSDTOMAMK-UHFFFAOYSA-N	13.58	280.7	unknown	[5, 6, 7]
C6H6ClN	QSNSCYSYFYORTR-UHFFFAOYSA-N	19.85	342.2	cr to l	4
C7H9N	RZXMPPFPUUCRFN-UHFFFAOYSA-N	17.30	315.6	cr to l	4
C6H8N2	CBCKQZAAMUWICA-UHFFFAOYSA-N	30.00	414.4	cr to l	4
C6H10O4	MUXOBHXGJLMRAB-UHFFFAOYSA-N	21.84	291.1	cr to l	4
C7H14O2	NUKZAGXMHTUAFE-UHFFFAOYSA-N	17.20	203.6	cr to l	4
C8H16O2	XNCNNDVCAUWAIT-UHFFFAOYSA-N	20.70	217.4	cr to l	4
C4H8O	RBACIKXCRWGCBB-UHFFFAOYSA-N	6.70	143.9	cr to l	4
C2H4Br2	PAAZPARNPHGIKF-UHFFFAOYSA-N	10.63	282.7	cr,I to l	4
C3H7Br	CYNYIHKIEHGYOZ-UHFFFAOYSA-N	6.53	163.1	cr to l	4
C4H10	IJDNQMDRQITEOD-UHFFFAOYSA-N	4.37	134.1	cr,I to l	4
C4H8	VXNZUUAINFGPBY-UHFFFAOYSA-N	3.85	87.8	cr to l	4
C4H6	KAKZBPTYRLMSJV-UHFFFAOYSA-N	7.98	164.2	cr to l	4
C4H6	KDKYADYSIPSCCQ-UHFFFAOYSA-N	6.03	147.5	cr to l	4
C3H8S	SUVIGLJNEAMWEG-UHFFFAOYSA-N	5.50	160.0	cr,I to l	4
C2H4BrCl	IBYHHJPAARCAIE-UHFFFAOYSA-N	9.62	256.5	cr,I to l	4
C3H9N	WGYKZJWCGVVSQN-UHFFFAOYSA-N	10.63	188.4	cr to l	4
C3H5N	FVSKHRXBFJPNKK-UHFFFAOYSA-N	5.03	180.4	cr,I to l	4
C3H3N	NLHHRLWOUZZQLW-UHFFFAOYSA-N	8.10	189.6	cr,I to l	4
C8H16	FXNDIJDIPNCZQJ-UHFFFAOYSA-N	7.87	172.1	cr to l	4
C8H16	LAAVYEUJEMRIGF-UHFFFAOYSA-N	6.80	166.0	cr to l	4
C6H14	AFABGHUZZDYHJO-UHFFFAOYSA-N	0.37	119.7	cr to l	4
C5H10O	XNLICIUVMPYHGG-UHFFFAOYSA-N	10.59	194.0	cr,I to l	4
C3H4N2O	DGJMPUGMZIKDRO-UHFFFAOYSA-N	21.70	387.3	cr,I to l	4
C4H6O2	XTXRWKRVRITETP-UHFFFAOYSA-N	8.46	180.6	unknown	[5, 6, 7]
C7H16	BZHMBWZPUJHVEE-UHFFFAOYSA-N	6.70	152.5	cr to l	4
C3H6N2O2	WRIRWRKPLXCTFD-UHFFFAOYSA-N	28.69	441.3	unknown	[5, 6, 7]
C6H14O	ZAFNJMIOTHYJRJ-UHFFFAOYSA-N	11.02	186.3	cr to l	4
C4H6O3	WFDIJRYMOXRFFG-UHFFFAOYSA-N	10.50	199.1	cr to l	4
C5H8O2	GAEKPEKOJKCEMS-UHFFFAOYSA-N	7.10	238.3	unknown	[5, 6, 7]
C4H4O3	RINCXYDBBGOEEQ-UHFFFAOYSA-N	20.40	392.1	cr to l	4
C4H2O3	FPYJFEHAWHCUMM-UHFFFAOYSA-N	12.93	325.8	cr to l	4
C4H6O3	RUOJZAUFBMNUDX-UHFFFAOYSA-N	9.62	224.9	cr to l	4
C6H4Br2	JSRLURSZEMLAFO-UHFFFAOYSA-N	14.23	266.3	cr to l	4
C8H10	IVSZLXZYQVIEFR-UHFFFAOYSA-N	11.44	219.6	cr,I to l	4

Continued on next page

Continued from previous page

Formula	InChIKey	$\Delta h_i^{\mathrm{fus}}$ KJ/mol	T_i^{fus}/K	Phase	Source
C6H6ClN	PNPCRKVUWYDDST-UHFFFAOYSA-N	11.60	262.8	cr to l	4
C6H5ClO	HORNXRXVQWOLPJ-UHFFFAOYSA-N	14.90	305.8	cr to l	4
C7H9N	JJYPMNFTHPTTDI-UHFFFAOYSA-N	7.08	242.8	cr to l	4
C6H8N2	WZCQRUWWHSTZEM-UHFFFAOYSA-N	15.57	339.1	cr to l	4
C7H9N	JYYNAJVZFGKDEQ-UHFFFAOYSA-N	6.32	209.2	cr to l	4
C7H9N	OISVCGZHLKNMSJ-UHFFFAOYSA-N	10.05	267.1	cr to l	4
C6H8N2	HJFZAYHYIWGLNL-UHFFFAOYSA-N	18.60	310.3	unknown	[5, 6, 7]
C5H6O3	VANNPISTIUFMLH-UHFFFAOYSA-N	12.46	329.2	unknown	[5, 6, 7]
C9H12	AUHZEENZYGFFBQ-UHFFFAOYSA-N	9.52	228.4	cr,I to l	4
C8H10O	TUAMRELNJMMDMT-UHFFFAOYSA-N	17.42	336.6	cr to l	4
C6H3Cl3	XKEFYDZQGKAQCN-UHFFFAOYSA-N	18.20	336.7	cr to l	4
C8H11N	BWZVCCNYKMEVEX-UHFFFAOYSA-N	9.54	229.0	cr to l	4
C6H11Br	AQNQQHJNRPDOQV-UHFFFAOYSA-N	10.79	216.9	unknown	[5, 6, 7]
C6H5Br	QARVLSVVCXYDNA-UHFFFAOYSA-N	10.63	242.4	cr to l	4
C7H14	UAEPNZWRGJTJPN-UHFFFAOYSA-N	6.67	146.2	cr to l	4
C6H7N	FKNQCJSGGFJEIZ-UHFFFAOYSA-N	11.56	276.8	cr,I to l	4
C6H5Cl	MVPPADPHJFYWMZ-UHFFFAOYSA-N	9.56	227.9	cr to l	4
C6H13N	PAFZNILMFXTMIY-UHFFFAOYSA-N	17.47	255.4	cr to l	4
C6H12O	HPXRVTGHNJAIIH-UHFFFAOYSA-N	1.70	297.0	cr,I to l	4
C6H10O	JHIVVAPYMSGYDF-UHFFFAOYSA-N	1.51	241.8	cr to l	4
C6H6S	RMVRSNDYEFQCLF-UHFFFAOYSA-N	11.48	258.3	cr to l	4
C6H7N	ITQTTZVARXURQS-UHFFFAOYSA-N	10.31	255.0	cr to l	4
C6H13N	NNWUEBIEOFQMSS-UHFFFAOYSA-N	18.58	269.4	cr to l	4
C6H7N	BSKHPKMHTQYZBB-UHFFFAOYSA-N	9.82	206.4	cr to l	4
C8H16O2	XUPYJHCZDLZNFP-UHFFFAOYSA-N	14.93	181.7	cr to l	4
C16H30O2	LOKPJYNMYCVCRM-UHFFFAOYSA-N	7.70	309.2	unknown	[5, 6, 7]
C5H10O2	NQPDZGIKBAWPEJ-UHFFFAOYSA-N	13.90	239.7	cr to l	4
C5H14N2	IUNMPGNGSSIWFP-UHFFFAOYSA-N	12.39	194.4	cr to l	4
C3H6Br2	VEFLKXRACNJHOV-UHFFFAOYSA-N	14.64	238.6	cr to l	4
C4H9Br	MPPPKRYCTPRNTB-UHFFFAOYSA-N	9.23	160.4	cr to l	4
C5H12	OFBQJSOFQDEBGM-UHFFFAOYSA-N	8.38	143.4	cr to l	4
C5H10	YWAKXRMUMFPDSH-UHFFFAOYSA-N	4.94	107.9	cr to l	4
C3H6BrCl	MFESCIUQSIBMSM-UHFFFAOYSA-N	9.22	214.3	cr to l	4
C3H2N2	CUONGYYJJVDODC-UHFFFAOYSA-N	10.04	307.6	cr,I to l	4
C4H10S	WQAQPCDUOCURKW-UHFFFAOYSA-N	8.70	157.5	cr to l	4
C3H8O2	NKDDWNXOKDWJAK-UHFFFAOYSA-N	8.33	168.0	cr to l	4
C4H11N	HPNMFZURTQLUMO-UHFFFAOYSA-N	13.30	224.7	unknown	[5, 6, 7]
C4H6O	QYKIQEUNHZKYBP-UHFFFAOYSA-N	7.90	173.1	cr to l	4
C4H5N	KAESVJOAVNADME-UHFFFAOYSA-N	7.91	249.8	cr to l	4
C4H8O	WYURNTSHIVDZCO-UHFFFAOYSA-N	9.10	164.1	cr to l	4
C4H4O	YLQBMQCUIZJEEH-UHFFFAOYSA-N	3.80	187.6	cr,I to l	4
C4H4S	YTPLMLYBLZKORZ-UHFFFAOYSA-N	4.97	233.7	cr,I to l	4
C4H8N2O2	SNCZNSNPXMPCGN-UHFFFAOYSA-N	6.08	485.9	unknown	[5, 6, 7]
C12H24O2	RGXWDWUGBIJHDO-UHFFFAOYSA-N	32.29	253.6	unknown	[5, 6, 7]
C11H22O2	YRHYCMZPEVDGFQ-UHFFFAOYSA-N	34.30	260.4	cr to l	4
C7H14O	CATSNJVOTSVZJV-UHFFFAOYSA-N	19.71	237.7	unknown	[5, 6, 7]
C5H11Br	YZWKKMVJZFACSU-UHFFFAOYSA-N	14.37	185.1	cr to l	4
C6H14	VLKZOEOYAKHREP-UHFFFAOYSA-N	12.58	178.6	cr to l	4
C5H9N	RFFFKMOABOFIDF-UHFFFAOYSA-N	9.00	177.0	cr to l	4
C4H4N2	IAHFWCOBPZCAEA-UHFFFAOYSA-N	11.67	330.1	cr,I to l	4
C5H10O	HGBOYTHUEUWSSQ-UHFFFAOYSA-N	15.00	151.6	unknown	[5, 6, 7]
C4H10O2	WERYXYBDKMZEQL-UHFFFAOYSA-N	18.70	293.6	cr to l	4
C5H12S	ZRKMQKLGEQPLNS-UHFFFAOYSA-N	17.53	197.5	cr to l	4
C4H10O2	XTHFKEDIFFGKHM-UHFFFAOYSA-N	12.60	203.9	cr to l	4
C4H11NO	MIJDSYMOBYNHOT-UHFFFAOYSA-N	16.50	263.9	unknown	[5, 6, 7]
C4H10S2	CETBSQOFQKLHHZ-UHFFFAOYSA-N	5.90	171.7	cr to l	4
C6H12	XDTMQSROBMDMFD-UHFFFAOYSA-N	2.70	279.7	cr to l	4
C6H10	HGCIXCUEYOPUTN-UHFFFAOYSA-N	3.29	169.0	cr,I to l	4

Continued on next page

Continued from previous page

Formula	InChIKey	Δh_i^{fus} KJ/mol	T_i^{fus}/K	Phase	Source
C3H6O3	BGJSXRVXTHVRSN-UHFFFAOYSA-N	15.11	333.4	cr,I to l	4
C5H11N	NQRYJNQNLNOLGT-UHFFFAOYSA-N	11.42	262.4	cr to l	4
C4H9NO	YNAVUWVOSKDBBP-UHFFFAOYSA-N	14.50	268.4	cr to l	4
C6H15NO2	LVTYICIALWPMFW-UHFFFAOYSA-N	22.74	250.0	unknown	[5, 6, 7]
C9H18O2	JGHZJRVDZXSNKQ-UHFFFAOYSA-N	25.10	236.5	cr to l	4
C8H16O	ZPVFWPFBNIEHGJ-UHFFFAOYSA-N	24.42	252.9	cr to l	4
C7H14O2	MNWFXJYAOYHMED-UHFFFAOYSA-N	14.50	266.0	cr,I to l	4
C6H13Br	MNDIARAMWBIKFW-UHFFFAOYSA-N	18.05	188.1	cr to l	4
C6H15N	BMVXCPBXGZKUPN-UHFFFAOYSA-N	25.04	252.2	unknown	[5, 6, 7]
C6H14O	ZSIAUFGUXNUGDI-UHFFFAOYSA-N	15.38	225.8	cr to l	4
C5H12O2	ALQSHHUCVQOPAS-UHFFFAOYSA-N	15.73	248.0	cr to l	4
C6H14S	PMBXCGGQNSVESQ-UHFFFAOYSA-N	17.00	192.7	cr to l	4
C6H14O	POLCUAVZOMRGSN-UHFFFAOYSA-N	10.77	158.4	cr to l	4
C4H8Cl2O	ZNSMNVMLTJELDZ-UHFFFAOYSA-N	8.39	226.5	unknown	[5, 6, 7]
C4H10O3	MTHSVFCYNBDYFN-UHFFFAOYSA-N	18.10	265.1	unknown	[5, 6, 7]
C6H14S	ZERULLAPCVRMCO-UHFFFAOYSA-N	12.08	170.4	cr to l	4
C20H40O2	MVLVMROFTAUDAG-UHFFFAOYSA-N	60.10	306.5	unknown	[5, 6, 7]
C20H38O2	LVGKNOAMLMIIKO-UHFFFAOYSA-N	47.40	254.0	unknown	[5, 6, 7]
C8H16	KWKAKUADMBZCLK-UHFFFAOYSA-N	15.31	171.5	cr to l	4
C6H8N2	BTGRAWJCKBQKAO-UHFFFAOYSA-N	18.00	275.0	unknown	[5, 6, 7]
C7H16O	BBMCTIGTTCKYKF-UHFFFAOYSA-N	18.17	240.0	cr to l	4
C7H14O	FXHGMKSSBGDXIY-UHFFFAOYSA-N	23.59	229.8	cr to l	4
C6H14O2	POAOYUHQDCAZBD-UHFFFAOYSA-N	11.80	199.5	unknown	[5, 6, 7]
C8H12	VYXHVRARDIDEHS-UHFFFAOYSA-N	9.83	204.0	cr,I to l	4
C13H26O2	UQDUPQYQJKYHQI-UHFFFAOYSA-N	43.10	278.2	cr to l	4
C8H17Br	VMKOFRJSULQZRM-UHFFFAOYSA-N	24.70	218.2	cr to l	4
C9H20	BKIMMITUMNQMOS-UHFFFAOYSA-N	22.12	219.2	cr,I to l	4
C8H19N	IOQPZZOEVPZRBK-UHFFFAOYSA-N	34.74	273.4	unknown	[5, 6, 7]
C8H18S	KZCOBXFFBQJQHH-UHFFFAOYSA-N	24.27	224.0	unknown	[5, 6, 7]
C6H14O3	SBZXBUIDTXKZTM-UHFFFAOYSA-N	17.80	209.1	cr to l	4
C9H18O2	FBUKVWPVBMHYJY-UHFFFAOYSA-N	20.29	285.5	cr,I to l	4
C11H22O	KYWIYKKSMDLRDC-UHFFFAOYSA-N	36.34	285.3	unknown	[5, 6, 7]
C10H22O	MWKFXSUHUHTGQN-UHFFFAOYSA-N	43.00	279.6	cr to l	4
C10H20O	KSMVZQYAVGTKIV-UHFFFAOYSA-N	34.50	269.2	cr to l	4
C11H22O2	ZDPHROOEEOARMN-UHFFFAOYSA-N	25.10	301.4	cr,I to l	4
C17H34O2	FLIACVVOZYBSBS-UHFFFAOYSA-N	68.00	304.5	cr to l	4
C12H26	SNRUBQQJIBEYMU-UHFFFAOYSA-N	36.58	263.5	cr to l	4
C12H24	CRSBERNSMYQZNG-UHFFFAOYSA-N	19.91	237.9	cr,I to l	4
C11H24O	KJIOQYGWTQBHNH-UHFFFAOYSA-N	34.06	297.9	unknown	[5, 6, 7]
C8H18O4	YFNKIDBQEZZDLK-UHFFFAOYSA-N	23.72	229.3	cr to l	4
C12H26O	LQZZUXJYWNFBMV-UHFFFAOYSA-N	35.00	296.6	cr to l	4
C12H24O	HFJRKMMYBMWEAD-UHFFFAOYSA-N	43.38	286.5	unknown	[5, 6, 7]
C8H18O5	UWHCKJMYHZGTIT-UHFFFAOYSA-N	28.20	265.5	unknown	[5, 6, 7]
C19H38O2	HPEUJPJOZXNMSJ-UHFFFAOYSA-N	48.00	310.2	cr to l	4
C13H28O	XFRVVPUIAFSTFO-UHFFFAOYSA-N	41.40	304.9	cr,I to l	4
C14H30O	HLZKNKRTKFSKGZ-UHFFFAOYSA-N	39.70	310.0	cr,I to l	4
C22H42O2	DPUOLQHDNGRHBS-UHFFFAOYSA-N	54.00	307.2	unknown	[5, 6, 7]
C18H38O	GLDOVTGHNKAZLK-UHFFFAOYSA-N	39.20	331.7	cr,I to l	4
C20H42	CBFCDTFDPHXCNY-UHFFFAOYSA-N	61.47	309.7	cr,I to l	4
C3H6	QQONPFPTGQHPMA-UHFFFAOYSA-N	2.94	88.2	cr to l	4
C2H6O	LCGLNKUTAGEVQW-UHFFFAOYSA-N	4.94	131.7	cr to l	4
C4H8	VQTUBCCKSQIDNK-UHFFFAOYSA-N	5.92	132.4	cr to l	4
C5H10O	HNVRRHSXBLFLIG-UHFFFAOYSA-N	5.20	245.0	unknown	[5, 6, 7]
C2H3Cl3O	KPWDGTGXUYRARH-UHFFFAOYSA-N	10.05	290.6	unknown	[5, 6, 7]
C4F8	BCCOBQSFUDVTJQ-UHFFFAOYSA-N	2.77	233.0	cr,I to l	4
C9H2Cl6O3	FLBJFXNAEMSXGL-UHFFFAOYSA-N	2.67	506.0	cr,I to l	4
C5H12O4	WXZMFSXDPGVJKK-UHFFFAOYSA-N	7.11	538.7	cr,I to l	4
C18H15O4P	XZZNDPSIHUTMOC-UHFFFAOYSA-N	29.61	322.6	cr to l	4

Continued on next page

Continued from previous page

Formula	InChIKey	Δh_i^{fus} KJ/mol	T_i^{fus}/K	Phase	Source
C2F4	BFKJFAAPBSQJPD-UHFFFAOYSA-N	7.72	142.0	cr to l	4
C12H6Cl4O2S	MLGCXEBRWGEOQX-UHFFFAOYSA-N	29.90	420.1	cr to l	4
C3H4Cl2O2	HKMLRUAPIDAGIE-UHFFFAOYSA-N	8.17	221.2	cr to l	4
C6HCl4NO2	XQTLDIFVVHJORV-UHFFFAOYSA-N	19.50	373.6	cr to l	4
C14H12O2	PYHXGXCGESYPCW-UHFFFAOYSA-N	31.27	420.4	cr to l	4
C10H4Cl2O2	SVPKNMBRVBMTLB-UHFFFAOYSA-N	28.50	469.6	cr to l	4
C13H10O2	HJIAMFHSAAEUKR-UHFFFAOYSA-N	0.67	308.1	cr to l	4
C10H12O5	SJSOFNCYXJUNBT-UHFFFAOYSA-N	29.90	444.5	unknown	[5, 6, 7]
C13H10O3	ZQBAKBUEJOMQEX-UHFFFAOYSA-N	18.32	312.0	unknown	[5, 6, 7]
C9H10O3	GYCKQBWUSACYIF-UHFFFAOYSA-N	24.20	274.5	unknown	[5, 6, 7]
C7H6Cl2	DMEDNTFWIHCBRK-UHFFFAOYSA-N	10.70	272.0	unknown	[5, 6, 7]
C6H6O3	XPCTZQVDEJYUGT-UHFFFAOYSA-N	21.20	433.4	unknown	[5, 6, 7]
C6Cl6	CKAPSXZOOQJIBF-UHFFFAOYSA-N	23.85	505.0	cr to l	4
C6Cl4O2	UGNWTBMOAKPKBL-UHFFFAOYSA-N	30.90	568.1	cr to l	4
C8H8O2	ZWLPBLYKEWSWPD-UHFFFAOYSA-N	20.17	376.9	cr to l	4
C7H5ClO2	IKCLCGXPQILATA-UHFFFAOYSA-N	25.73	413.4	cr to l	4
C8H8O2	JECYUBVRTQDVAT-UHFFFAOYSA-N	13.00	278.5	unknown	[5, 6, 7]
C7H5N3O6	SPSSULHKWOKEEL-UHFFFAOYSA-N	22.41	355.1	cr to l	4
C7H6N2O5	CVYZVNVPQRKDLW-UHFFFAOYSA-N	20.23	369.4	unknown	[5, 6, 7]
C14H12O2	ISAOCJYIOMOJEB-UHFFFAOYSA-N	38.08	407.4	unknown	[5, 6, 7]
C10H12	CXWXQJXEFPUFDZ-UHFFFAOYSA-N	12.45	237.4	cr to l	4
C9H7N	AWJUIBRHMBBTKR-UHFFFAOYSA-N	13.54	299.6	cr,I to l	4
C12H10N2O2	RUKISNQKOIKZGT-UHFFFAOYSA-N	26.14	347.9	unknown	[5, 6, 7]
C14H10	MWPLVEDNUUSJAV-UHFFFAOYSA-N	28.87	489.7	cr to l	4
C9H10O3	WJUFSDZVCOTFON-UHFFFAOYSA-N	20.30	317.0	unknown	[5, 6, 7]
C12H10O3	RZCJYMOBWVJQGV-UHFFFAOYSA-N	38.50	429.4	cr to l	4
C9H10O3	NUVBSKCKDOMJSU-UHFFFAOYSA-N	27.99	388.5	unknown	[5, 6, 7]
C10H10O4	WOZVHXUHUFLZGK-UHFFFAOYSA-N	32.10	413.8	cr to l	4
C9H11NO	BPEXTIMJLDWDTL-UHFFFAOYSA-N	21.70	382.7	unknown	[5, 6, 7]
C6H4Cl2O	HFZWRUODUSTPEG-UHFFFAOYSA-N	28.00	323.0	cr to l	4
C5H8O	BGTOWKSIORTVQH-UHFFFAOYSA-N	11.40	221.2	unknown	[5, 6, 7]
C3H6N2O	YAMHXTCMCPHKLN-UHFFFAOYSA-N	5.11	397.3	cr,I to l	4
C7H6N2O4	RMBFBMJGBANMMK-UHFFFAOYSA-N	20.10	343.1	cr to l	4
C9H10O3	CBOQJANXLMLOSS-UHFFFAOYSA-N	23.48	351.3	unknown	[5, 6, 7]
C8H8O4	WKOLLVMJNQIZCI-UHFFFAOYSA-N	30.53	483.4	unknown	[5, 6, 7]
C7H8	SJYNFBVQFBRSIB-UHFFFAOYSA-N	7.00	205.1	cr to l	4
C8H8O2	LUJMEECXHPYQOF-UHFFFAOYSA-N	23.85	367.4	unknown	[5, 6, 7]
C6H4ClNO2	KMAQZIILEGKYQZ-UHFFFAOYSA-N	19.71	317.4	unknown	[5, 6, 7]
C3H6N6O6	XTFIVUDBNACUBN-UHFFFAOYSA-N	35.65	478.5	cr to l	4
C8H7NO3	ARKIFHPFTHVKDT-UHFFFAOYSA-N	17.96	351.2	unknown	[5, 6, 7]
C8H6O4	QQVIHTHCMHWDBS-UHFFFAOYSA-N	38.15	617.9	unknown	[5, 6, 7]
C7H5NO4	AFPHTEQTJZKQAQ-UHFFFAOYSA-N	19.33	414.3	cr to l	4
C9H10O3	DDIZAANNODHTRB-UHFFFAOYSA-N	19.90	321.2	unknown	[5, 6, 7]
C13H5N3O7	VHQGURIJMFPBKS-UHFFFAOYSA-N	23.50	449.0	cr,I to l	4
C12H14N2O5	QJYHUJAGJUHXJN-UHFFFAOYSA-N	28.00	378.7	cr to l	4
C14H7ClO2	FPKCTSIVDAWGFA-UHFFFAOYSA-N	33.00	483.0	cr to l	4
C10H10O4	NIQCNGHVCWTJSM-UHFFFAOYSA-N	16.94	274.2	cr to l	4
C14H12O4	MEZZCSHVIGVWFI-UHFFFAOYSA-N	22.00	343.0	unknown	[5, 6, 7]
C15H14O5	SODJJEXAWOSSON-UHFFFAOYSA-N	33.20	412.3	unknown	[5, 6, 7]
C13H10O5	WXNRYSGJLQFHBR-UHFFFAOYSA-N	28.00	472.0	unknown	[5, 6, 7]
C9H8Cl3NO2S	LDVVMCZRFWMZSG-UHFFFAOYSA-N	44.40	447.1	cr to l	4
C9H4Cl3NO2S	HKIOYBQGHSTUDB-UHFFFAOYSA-N	35.60	454.1	cr to l	4
C7H5Cl2NO2	HSSBORCLYSCBJR-UHFFFAOYSA-N	38.90	474.5	cr to l	4
C8H9NO2	VAMXMNNIEUEQDV-UHFFFAOYSA-N	11.84	297.6	cr to l	4
C10H9N	RUFPHBVGCFYCNW-UHFFFAOYSA-N	13.36	320.7	cr to l	4
C14H10O2	WURBFLDFSFBTLW-UHFFFAOYSA-N	22.69	368.1	cr,I to l	4
C8H8O2	PKZJLOCLABXVMC-UHFFFAOYSA-N	20.40	310.2	unknown	[5, 6, 7]
C10H8O	JWAZRIHNYRIHIV-UHFFFAOYSA-N	18.79	393.8	cr to l	4

Continued on next page

Continued from previous page

Formula	InChIKey	Δh_i^{fus} KJ/mol	T_i^{fus}/K	Phase	Source
C24H18	GPRIERYVMZVKTC-UHFFFAOYSA-N	37.80	587.2	cr,I to l	4
C7H6N2S	UHGULLIUJBCTEF-UHFFFAOYSA-N	18.88	402.3	unknown	[5, 6, 7]
C12H10S	LTYMSROWYAPPGB-UHFFFAOYSA-N	13.98	258.0	unknown	[5, 6, 7]
C8H7N	SUSQOBVLVYHIEX-UHFFFAOYSA-N	10.00	251.2	cr to l	4
C9H11ClN2O	BMLIZLVNXIYGCK-UHFFFAOYSA-N	3.35	441.5	cr to l	4
C7H12O2	CQEYYJKEWSMYFG-UHFFFAOYSA-N	17.31	209.5	cr to l	4
C4H8O2	XEKOWRVHYACXOJ-UHFFFAOYSA-N	10.48	189.3	cr to l	4
C3H6Cl2	YHRUOJUYPBUZOS-UHFFFAOYSA-N	13.60	239.0	cr to l	4
C5H8	LPIQUOYDBNQMRZ-UHFFFAOYSA-N	3.36	138.1	cr,I to l	4
C5H10O	DHXVGJBLRPWPCS-UHFFFAOYSA-N	1.78	224.1	cr,I to l	4
C7H16	IMNFDUFMRHMDMM-UHFFFAOYSA-N	14.16	182.2	cr to l	4
C8H16O2	AOGQPLXWSUTHQB-UHFFFAOYSA-N	19.83	212.1	cr to l	4
C9H20O	ZWRUINPWMLAQRD-UHFFFAOYSA-N	24.54	268.1	unknown	[5, 6, 7]
C10H23N	MHZGKXUYDGKKIU-UHFFFAOYSA-N	42.70	289.2	unknown	[5, 6, 7]
C10H22S	VTXVGVNLYGSIAR-UHFFFAOYSA-N	33.35	247.9	cr to l	4
C10H22O5	ZUHZGEOKBKGPSW-UHFFFAOYSA-N	29.70	244.5	unknown	[5, 6, 7]
C8H18O2	JCTXKRPTIMZBJT-UHFFFAOYSA-N	24.20	328.3	unknown	[5, 6, 7]
C6H4Br3N	GVPODVKBTHCGFU-UHFFFAOYSA-N	19.90	395.1	cr to l	4
C7H8O2	NWVVVBRKAWDGAB-UHFFFAOYSA-N	18.25	328.4	unknown	[5, 6, 7]
C8H10O2	OHBQPCCCRFSCAX-UHFFFAOYSA-N	20.10	333.0	unknown	[5, 6, 7]
C8H11NO	IMPPGHMHELILKG-UHFFFAOYSA-N	11.36	277.8	cr to l	4
C3H9NO	WUGQZFFCHPXWKQ-UHFFFAOYSA-N	19.70	285.6	cr to l	4
C24H14	TUGYIJVAYAHHHM-UHFFFAOYSA-N	27.90	556.8	cr to l	4
C24H12	VPUGDVKSAQVFFS-UHFFFAOYSA-N	19.20	710.5	cr,I to l	4
C22H12	GYFAGKUZYNFMBN-UHFFFAOYSA-N	17.36	554.2	cr to l	4
C24H14	JNTHRSHGARDABO-UHFFFAOYSA-N	24.69	501.2	cr to l	4
C24H14	KGHMWBNEMFNJFZ-UHFFFAOYSA-N	30.50	520.2	cr to l	4
C20H12	TXVHTIQJNYSSKO-UHFFFAOYSA-N	16.57	454.5	cr,I to l	4
C22H12	SXQBHARYMNFBPS-UHFFFAOYSA-N	16.30	437.7	cr to l	4
C20H13N	STJXCDGCXVZHDU-UHFFFAOYSA-N	20.10	429.8	unknown	[5, 6, 7]
C22H14	YZWGEMSQAMDWEM-UHFFFAOYSA-N	22.70	398.5	unknown	[5, 6, 7]
C18H12	TUAHORSUHVUKBD-UHFFFAOYSA-N	16.30	334.8	cr to l	4
C18H10	YEIHPPOCKIHUQJ-UHFFFAOYSA-N	11.80	424.4	cr,I to l	4
C20H12	KHNYNFUTFKJLDD-UHFFFAOYSA-N	17.90	438.3	unknown	[5, 6, 7]
C20H12	FTOVXSOBNPWTSH-UHFFFAOYSA-N	19.60	441.5	unknown	[5, 6, 7]
C16H10	GVEPBJHOBDJJJI-UHFFFAOYSA-N	18.65	383.3	cr to l	4
C20H12	HAXBIWFMXWRORI-UHFFFAOYSA-N	29.95	490.2	unknown	[5, 6, 7]
C12H8	HXGDTGSAIMULJN-UHFFFAOYSA-N	6.94	362.6	cr to l	4
C22H14	GBROPGWFBFCKAG-UHFFFAOYSA-N	35.20	637.2	cr to l	4
C22H14	RAASUWZPTOJQAY-UHFFFAOYSA-N	25.82	553.5	cr to l	4
C18H12	SLGBZMMZGDRARJ-UHFFFAOYSA-N	24.74	471.0	cr to l	4
C18H12	WDECIBYCCFPHNR-UHFFFAOYSA-N	26.15	531.4	cr,I to l	4
C21H13N	ANUCHZVCBDOPOX-UHFFFAOYSA-N	25.50	492.7	unknown	[5, 6, 7]
C17H11N	OAPPEBNXKAKQGS-UHFFFAOYSA-N	20.30	381.4	unknown	[5, 6, 7]
C21H13N	JNCSIWAONQTVCF-UHFFFAOYSA-N	30.60	499.7	unknown	[5, 6, 7]
C13H9N	RDOWQLZANAYVLL-UHFFFAOYSA-N	22.83	379.7	cr,I to l	4
C13H9N	WZJYKHNJTSNBHV-UHFFFAOYSA-N	14.10	324.1	cr to l	4
C17H12	HKMTVMBEALTRRR-UHFFFAOYSA-N	18.40	462.8	cr,I to l	4
C17H12	HAPOJKSPCGLOOD-UHFFFAOYSA-N	23.40	489.7	cr to l	4
C8H6N2	LFSXCDWNBUNEEM-UHFFFAOYSA-N	13.32	364.5	unknown	[5, 6, 7]
C13H9N	DZBUGLKDJFMEHC-UHFFFAOYSA-N	18.60	384.0	cr to l	4
C12H8O2	NFBOHOGPQUYFRF-UHFFFAOYSA-N	23.55	392.8	unknown	[5, 6, 7]
C7H5NO	BCMCBBGGLRIHSE-UHFFFAOYSA-N	16.78	302.5	cr,I to l	4
C10H8	CUFNKYGDVFVPHO-UHFFFAOYSA-N	17.53	373.5	unknown	[5, 6, 7]
C7H12	UMRZSTCPUPJPOJ-UHFFFAOYSA-N	4.45	360.8	unknown	[5, 6, 7]
C8H14	GPRLTFBKWDERLU-UHFFFAOYSA-N	8.35	447.5	cr,I to l	4
C6H12N2	IMNIMPAHZVJRPE-UHFFFAOYSA-N	7.43	433.0	cr,I to l	4
C10H16	ORILYTVJVMAKLC-UHFFFAOYSA-N	8.03	552.0	cr,I to l	4

Continued on next page

Continued from previous page

Formula	InChIKey	Δh_i^{fus} KJ/mol	T_i^{fus}/K	Phase	Source
C4H8	PMPVIKIVABFJJI-UHFFFAOYSA-N	1.04	182.9	cr,I to l	4
C3H6S	XSROQCDVUIHRSI-UHFFFAOYSA-N	8.25	199.9	cr,I to l	4
C5H10	RGSFGYAAUTVSQA-UHFFFAOYSA-N	0.61	179.0	cr,I to l	4
C3H3NS	FZWLAAWBMGSTSO-UHFFFAOYSA-N	9.59	239.5	cr to l	4
C2H3N3	NSPMIYGKQJPBQR-UHFFFAOYSA-N	6.24	392.2	cr to l	4
C4H4N2	CZPWVGJYEJSRLH-UHFFFAOYSA-N	13.00	294.3	unknown	[5, 6, 7]
C4H4N2	KYQCOXFCLRTKLS-UHFFFAOYSA-N	12.95	324.2	cr,I to l	4
C3H6S3	LORRLQMLLQLPSJ-UHFFFAOYSA-N	32.22	488.4	unknown	[5, 6, 7]
C7H14	DMEGYFMYUHOHGS-UHFFFAOYSA-N	1.89	265.0	cr,I to l	4
C8H16	WJTCGQSWYFHTAC-UHFFFAOYSA-N	2.51	288.0	cr,I to l	4
C12H24	DDTBPAQBQHZRDW-UHFFFAOYSA-N	14.80	333.8	cr,I to l	4
C9H4Cl8O	LRWHHSXTGZSMSN-UHFFFAOYSA-N	25.90	395.4	cr to l	4
C7H6O4	AKEUNCKRJATALU-UHFFFAOYSA-N	25.15	442.0	unknown	[5, 6, 7]
C2HCl2F3	OHMHBGPWCHTMQE-UHFFFAOYSA-N	5.51	145.7	unknown	[5, 6, 7]
C8F18	YVBBRRALBYAZBM-UHFFFAOYSA-N	9.58	254.2	cr,I to l	4
C9H13BrN2O2	CTSLUCNDVMMDHG-UHFFFAOYSA-N	22.00	430.9	cr to l	4
C12H18N2O2	YNEVBPNZHBAYOA-UHFFFAOYSA-N	18.40	362.1	cr to l	4
C6H4N2O5	UWEZBKLLMKVIPI-UHFFFAOYSA-N	23.73	381.0	cr to l	4
C9H10Cl2N2O2	XKJMBINCVNINCA-UHFFFAOYSA-N	28.70	365.9	cr to l	4
C7F16	LGUZHRODIJCVOC-UHFFFAOYSA-N	6.95	221.9	cr,I to l	4
C13H8F2O	LSQARZALBDFYQZ-UHFFFAOYSA-N	18.08	382.0	unknown	[5, 6, 7]
C6H4ClF	RJCGZNCCVKIBHO-UHFFFAOYSA-N	13.90	245.0	unknown	[5, 6, 7]
C7H7F	BTQZKHUEUDPRST-UHFFFAOYSA-N	8.30	184.0	cr to l	4
C4H10S	LJSQFQKUNVCTIA-UHFFFAOYSA-N	13.30	169.2	cr to l	4
CF2O	IYRWEQXVUNLMAY-UHFFFAOYSA-N	6.70	161.9	unknown	[5, 6, 7]
C2HF5	GTLACDSXYULKMZ-UHFFFAOYSA-N	2.25	172.6	unknown	[5, 6, 7]
C2Cl3F3	BOSAWIQFTJIYIS-UHFFFAOYSA-N	4.11	287.5	cr,I to l	4
C4F10	KAVGMUDTWQVPDF-UHFFFAOYSA-N	7.66	144.0	cr,I to l	4
C6F14	ZJIJAJXFLBMLCK-UHFFFAOYSA-N	6.61	186.9	cr,I to l	4
C6HF5	WACNXHCZHTVBJM-UHFFFAOYSA-N	10.85	225.8	cr to l	4
C6H4F2	GOYDNIKZWGIXJT-UHFFFAOYSA-N	11.05	226.0	cr to l	4
C6H5FO	RHMPLDJJXGPMEX-UHFFFAOYSA-N	14.50	320.4	unknown	[5, 6, 7]
C6H4F2	UEMGWPRHOOEKTA-UHFFFAOYSA-N	8.58	204.0	cr,I to l	4
C6F6	ZQBFAOFFOQMSGJ-UHFFFAOYSA-N	11.59	278.3	cr to l	4
C8H7FO	ZDPAWHACYDRYIW-UHFFFAOYSA-N	14.20	269.0	unknown	[5, 6, 7]
CH2N2	XZMCDFZZKTWFGF-UHFFFAOYSA-N	7.27	318.7	cr to l	4
C2H3F3	UJPMYEOUBPIPHQ-UHFFFAOYSA-N	6.19	161.8	cr,I to l	4
C8BrF17	WTWWXOGTJWMJHI-UHFFFAOYSA-N	12.13	278.9	unknown	[5, 6, 7]
C7F8	USPWUOFNOTUBAD-UHFFFAOYSA-N	11.49	207.7	cr to l	4
C7H5FO2	NSTREUWFTAOOKS-UHFFFAOYSA-N	20.10	397.4	unknown	[5, 6, 7]
C10H10	KEIFWROAQVVDBN-UHFFFAOYSA-N	10.53	264.4	unknown	[5, 6, 7]
C7H5FO2	BBYDXOIZLAWGSL-UHFFFAOYSA-N	20.90	451.2	unknown	[5, 6, 7]
C2N2	JMANVNJQNLATNU-UHFFFAOYSA-N	8.11	245.3	cr to l	4
C2H4N4	QGBSISYHAICWAH-UHFFFAOYSA-N	23.98	485.2	unknown	[5, 6, 7]
C3H4N2O2	WJRBRSLFGCUECM-UHFFFAOYSA-N	22.83	494.1	unknown	[5, 6, 7]
C6H5F	PYLWMHQQBFSUBP-UHFFFAOYSA-N	10.40	231.1	cr to l	4
C5H6N2	CUYKNJBYIJFRCU-UHFFFAOYSA-N	14.40	335.5	unknown	[5, 6, 7]
C3H4	IYABWNGZIDDRAK-UHFFFAOYSA-N	4.40	136.6	cr to l	4
COS	JJWKPURADFRFRB-UHFFFAOYSA-N	4.73	134.3	cr to l	4
C5H12	CRSOQBOWXPBRES-UHFFFAOYSA-N	3.26	256.5	cr,I to l	4
C7H16	ZISSAWUMDACLOM-UHFFFAOYSA-N	2.20	247.7	cr,I to l	4
C6H14O	DFOXKPDFWGNLJU-UHFFFAOYSA-N	8.40	278.4	cr to l	4
C7H5N5O8	AGUIVNYEYSCPNI-UHFFFAOYSA-N	25.86	400.5	cr to l	4
C18H18	NXLOLUFNDSBYTP-UHFFFAOYSA-N	18.03	369.0	cr to l	4
C15H18N2O6	ZRDUSMYWDRPZRM-UHFFFAOYSA-N	18.90	343.8	cr to l	4
C13H8O	YLQWCDOCJODRMT-UHFFFAOYSA-N	16.73	355.6	unknown	[5, 6, 7]
C10H14	UOHMMEJUHBCKEE-UHFFFAOYSA-N	11.23	265.4	cr to l	4
C10H12O5	KVZUCOGWKYOPID-UHFFFAOYSA-N	31.15	417.9	unknown	[5, 6, 7]

Continued on next page

Continued from previous page

Formula	InChIKey	Δh_i^{fus} KJ/mol	T_i^{fus}/K	Phase	Source
C13H8OS	YRHRIQCWCFGUEQ-UHFFFAOYSA-N	31.95	487.3	unknown	[5, 6, 7]
C8H7ClO3	BWSFWXSSALIZAU-UHFFFAOYSA-N	27.20	394.0	unknown	[5, 6, 7]
C8H8O3	BHUIUXNAPJIDOG-UHFFFAOYSA-N	18.05	327.1	unknown	[5, 6, 7]
C9H10	PQNFLJBBNBOBRQ-UHFFFAOYSA-N	8.60	221.8	cr to l	4
C4H4O2	VIHAEDVKXSOUAT-UHFFFAOYSA-N	10.85	272.2	unknown	[5, 6, 7]
C7H10O	KPMKEVXVVHNIEY-UHFFFAOYSA-N	3.39	368.7	unknown	[5, 6, 7]
C5H8O4	WXUAQHNMJWJLTG-UHFFFAOYSA-N	9.98	383.0	unknown	[5, 6, 7]
C7H10	JFNLZVQOOSMTJK-UHFFFAOYSA-N	3.48	319.5	unknown	[5, 6, 7]
C14H10	JRXXLCKWQFKACW-UHFFFAOYSA-N	21.38	333.1	cr to l	4
C7H12O	CGZZMOTZOONQIA-UHFFFAOYSA-N	1.39	259.3	unknown	[5, 6, 7]
C6H10O2	PAPBSGBWRJIAAV-UHFFFAOYSA-N	13.82	271.8	cr to l	4
C9H18O	WSGCRAOTEDLMFQ-UHFFFAOYSA-N	24.93	269.3	cr,I to l	4
C15H28O	OSOIQJGOYGSIMF-UHFFFAOYSA-N	8.80	338.4	unknown	[5, 6, 7]
C4H6	XNMQEEKYCVKGBD-UHFFFAOYSA-N	9.24	240.9	cr to l	4
C3H6O	AHHWIHXENZJRFG-UHFFFAOYSA-N	6.50	176.1	cr to l	4
C9H14O	MTZWHHIREPJPTG-UHFFFAOYSA-N	17.07	299.0	cr to l	4
C5H6N2	ICSNLGPSRYBMBD-UHFFFAOYSA-N	16.10	331.9	unknown	[5, 6, 7]
C3H8O2	YPFDHNVEDLHUCE-UHFFFAOYSA-N	7.10	245.5	cr to l	4
C4H8S2	WQADWIOXOXRPLN-UHFFFAOYSA-N	14.40	327.2	cr,I to l	4
C4H8S2	LOZWAPSEEHRYPG-UHFFFAOYSA-N	21.60	384.6	cr to l	4
C8H14O4	TYFQFVWCELRYAO-UHFFFAOYSA-N	29.16	415.5	cr,I to l	4
C17H34O2	KEMQGTRYUADPNZ-UHFFFAOYSA-N	51.50	334.4	cr,I to l	4
C20H40O2	VKOBVWXKNCXXDE-UHFFFAOYSA-N	72.00	348.4	cr to l	4
CClN	QPJDMGCKMHUXFD-UHFFFAOYSA-N	11.38	266.3	unknown	[5, 6, 7]
C4H9Br	RKSOPLXZQNSWAS-UHFFFAOYSA-N	1.97	256.2	cr,I to l	4
C4H9Cl	NBRKLOOSMBRFMH-UHFFFAOYSA-N	2.09	246.1	cr,I to l	4
C5H10	BKOOMYPCSUNDGP-UHFFFAOYSA-N	7.44	138.9	cr to l	4
C4H10S	BDFAOUQQXJIZDG-UHFFFAOYSA-N	4.98	128.3	cr to l	4
C4H10S	LOCHFZBWPCLPAN-UHFFFAOYSA-N	6.47	133.0	cr to l	4
C6H8O3	OMQHDIHZSDEIFH-UHFFFAOYSA-N	15.30	342.9	unknown	[5, 6, 7]
C19H16	AAAQKTZKLRYKHR-UHFFFAOYSA-N	18.16	365.5	cr to l	4
C15H10O2	VHBFFQKBGNRLFZ-UHFFFAOYSA-N	20.32	369.9	unknown	[5, 6, 7]
C9H12	FYGHSUNMUKGBRK-UHFFFAOYSA-N	8.40	247.8	cr,I to l	4
C8H10O	QWBBPBRQALCEIZ-UHFFFAOYSA-N	21.02	346.0	cr to l	4
C6H2Cl5N	KHCZSJXTDDHLGJ-UHFFFAOYSA-N	18.70	507.1	cr to l	4
C10H14	BFIMMTCNYPIMRN-UHFFFAOYSA-N	10.67	249.2	cr to l	4
C9H12O	BPRYUXCVCCNUFE-UHFFFAOYSA-N	21.40	345.1	unknown	[5, 6, 7]
C5H4O2S	QERYCTSHXKAMIS-UHFFFAOYSA-N	20.30	401.2	unknown	[5, 6, 7]
C8H9NO	XXUNIGZDNWWYED-UHFFFAOYSA-N	22.25	415.0	unknown	[5, 6, 7]
C6H4N2O4	IZUKQUVSCNEFMJ-UHFFFAOYSA-N	22.84	390.1	cr to l	4
C9H6O6	ARCGXLSVLAOJQL-UHFFFAOYSA-N	30.80	499.6	unknown	[5, 6, 7]
C7H4N2O6	OMVRRHJJQILIJX-UHFFFAOYSA-N	24.60	438.2	unknown	[5, 6, 7]
C8H7N	NWPNXBQSRGKSJB-UHFFFAOYSA-N	14.40	262.8	cr,I to l	4
C8H8O	BTFQKIATRPGRBS-UHFFFAOYSA-N	10.20	233.4	unknown	[5, 6, 7]
C9H10O5	JMSVCTWVEWCHDZ-UHFFFAOYSA-N	33.88	482.4	unknown	[5, 6, 7]
C10H8O3	LIIALPBMIOVAHH-UHFFFAOYSA-N	23.16	391.0	unknown	[5, 6, 7]
C8H8ClNO	KNVQTRVKSOEHPU-UHFFFAOYSA-N	25.30	359.9	cr to l	4
C6H6O3	GGNQRNBDZQJCCN-UHFFFAOYSA-N	28.80	413.2	unknown	[5, 6, 7]
C5H6O	VQKFNUFAXTZWDK-UHFFFAOYSA-N	8.55	181.9	cr to l	4
C7H6N2O5	ZXVONLUNISGICL-UHFFFAOYSA-N	20.50	359.3	cr to l	4
C8H6	UEXCJVNBTNXOEH-UHFFFAOYSA-N	9.46	228.0	cr to l	4
C9H12O3	FNQIYTUXOKTMDM-UHFFFAOYSA-N	29.90	336.6	unknown	[5, 6, 7]
C13H11N	UVEWQKMPXAHFST-UHFFFAOYSA-N	20.42	329.7	unknown	[5, 6, 7]
C8H8ClNO	GGUOCFNAWIODMF-UHFFFAOYSA-N	40.80	451.6	cr to l	4
C7H14O2	ICMAFTSLXCXHRK-UHFFFAOYSA-N	12.97	181.3	unknown	[5, 6, 7]
C9H18O2	CFNJLPHOBMVMNS-UHFFFAOYSA-N	20.01	200.5	cr to l	4
C3H7Cl	SNMVRZFUUCLYTO-UHFFFAOYSA-N	5.54	150.2	cr to l	4
C2H2Cl2	KFUSEUYYWQURPO-UHFFFAOYSA-N	12.00	223.4	cr,I to l	4

Continued on next page

Continued from previous page

Formula	InChIKey	Δh_i^{fus} KJ/mol	T_i^{fus}/K	Phase	Source
C8H18	NHTMVDHEPJAVLT-UHFFFAOYSA-N	9.04	165.3	cr to l	4
C5H12S	GIJGXNFNUUFEGH-UHFFFAOYSA-N	7.41	139.6	cr to l	4
C4H9NO	DNSISZSEWVHGLH-UHFFFAOYSA-N	18.85	336.2	unknown	[5, 6, 7]
C6H4Cl2	ZPQOPVIELGIULI-UHFFFAOYSA-N	12.59	248.8	cr to l	4
C6H11Cl	UNFUYWDGSFDHCW-UHFFFAOYSA-N	2.77	230.0	cr,I to l	4
C3H4ClN	GNHMRTZZNHZDDM-UHFFFAOYSA-N	12.30	221.7	cr to l	4
C5H6N2	ZTOMUSMDRMJOTH-UHFFFAOYSA-N	9.54	243.6	cr to l	4
C7H8	CHVJITGCYZJHLR-UHFFFAOYSA-N	1.16	197.9	cr,I to l	4
C8H18S	HTIRHQRTDBPHNZ-UHFFFAOYSA-N	18.30	198.1	cr to l	4
C14H28O2	TUNFSRHWOTWDNC-UHFFFAOYSA-N	44.70	327.4	cr to l	4
C16H34	DCAYPVUWAIABOU-UHFFFAOYSA-N	51.86	291.3	cr to l	4
C32H66	QHMGJGNTMQDRQA-UHFFFAOYSA-N	69.00	342.6	cr,I to l	4
C6H2F4	SOZFIIXUNAKEJP-UHFFFAOYSA-N	10.93	233.3	cr,I to l	4
C7H5NO4	SLAMLWHELXOEJZ-UHFFFAOYSA-N	27.99	419.0	cr to l	4
C9H4O5	SRPWOOOHEPICQU-UHFFFAOYSA-N	10.46	385.0	cr to l	4
C7H5NO3	CMWKITSNTDAEDT-UHFFFAOYSA-N	20.43	315.3	unknown	[5, 6, 7]
C10H8N2	MWVTWFVJZLCBMC-UHFFFAOYSA-N	20.40	381.3	unknown	[5, 6, 7]
C4H6O4	LOMVENUNSWAXEN-UHFFFAOYSA-N	21.05	322.6	cr to l	4
C5H6S	XQQBUAPQHNYYRS-UHFFFAOYSA-N	9.10	209.8	cr to l	4
C6H5NO3	RTZZCYNQPHTPPL-UHFFFAOYSA-N	21.30	364.0	cr to l	4
C12H16Cl2N2O	CCGPUGMWYLICGL-UHFFFAOYSA-N	29.70	374.6	cr to l	4
C6H12O2	VKONPUDBRVKQLM-UHFFFAOYSA-N	1.90	381.7	unknown	[5, 6, 7]
CH4N4O2	IDCPFAYURAQKDZ-UHFFFAOYSA-N	33.46	505.2	unknown	[5, 6, 7]
C4H10O	VNKYTQGIUYNRMY-UHFFFAOYSA-N	7.67	134.0	cr to l	4
CBr4	HJUGFYREWKUQJT-UHFFFAOYSA-N	3.95	363.3	cr,I to l	4
C6H12	PKXHXOTZMFCXSH-UHFFFAOYSA-N	1.10	158.4	cr,I to l	4
C8H18	OKVWYBALHQFVFP-UHFFFAOYSA-N	1.52	171.5	cr to l	4
C7H16	AEXMKKGTQYQZCS-UHFFFAOYSA-N	7.07	138.2	cr,I to l	4
C8H18	KUMXLFIBWFCMOJ-UHFFFAOYSA-N	8.91	147.0	cr to l	4
C5H10	YHQXBTXEYZIYOV-UHFFFAOYSA-N	5.35	104.7	cr to l	4
C5H10	MHNNAWXXUZQSNM-UHFFFAOYSA-N	7.91	135.6	cr to l	4
C6H12	OWWIWYDDISJUMY-UHFFFAOYSA-N	5.45	115.9	cr to l	4
C6H12	WGLLSSPDPJPLOR-UHFFFAOYSA-N	5.46	198.5	cr,I to l	4
C5H10O	SYBYTAAJFKOIEJ-UHFFFAOYSA-N	9.34	180.0	cr to l	4
C8H18	XTDQDBVBDLYELW-UHFFFAOYSA-N	8.63	160.9	cr to l	4
C7H16	WGECXQBGLLYSFP-UHFFFAOYSA-N	5.89	149.4	cr to l	4
C8H18	RLPGDEORIPLBNF-UHFFFAOYSA-N	9.27	163.6	cr to l	4
C7H14O	HXVNBWAKAOHACI-UHFFFAOYSA-N	11.18	204.8	cr to l	4
C12H12	APQSQLNWAIULLK-UHFFFAOYSA-N	10.60	279.9	cr to l	4
C12H12	SDDBCEWUYXVGCQ-UHFFFAOYSA-N	20.00	355.2	unknown	[5, 6, 7]
C6H4N2O5	JCRIDWXIBSEOEG-UHFFFAOYSA-N	19.58	336.0	cr to l	4
C12H12	CBMXCNPQDUJNHT-UHFFFAOYSA-N	8.50	257.0	unknown	[5, 6, 7]
C6H4Cl2O	UMPSXRYVXUPCOS-UHFFFAOYSA-N	21.36	330.0	cr to l	4
C8H10O	NXXYKOUNUYWIHA-UHFFFAOYSA-N	18.90	318.9	cr to l	4
C15H10O3	HVQAJTFOCKOKIN-UHFFFAOYSA-N	32.45	443.6	unknown	[5, 6, 7]
C8H8O3	ILUJQPXNXACGAN-UHFFFAOYSA-N	23.10	374.0	cr to l	4
C10H7Br	APSMUYYLXZULMS-UHFFFAOYSA-N	14.40	329.0	cr,I to l	4
C12H12	WWGUMAYGTYQSGA-UHFFFAOYSA-N	15.90	378.4	cr,I to l	4
C12H12	YGYNBBAUIYTWBF-UHFFFAOYSA-N	23.85	383.3	cr to l	4
C10H7NO2	ZJYJZEAJZXVAMF-UHFFFAOYSA-N	14.50	348.2	unknown	[5, 6, 7]
C12H12	LRQYSMQNJLZKPS-UHFFFAOYSA-N	24.18	368.8	cr to l	4
C6H4Br2	WQONPSCCEXUXTQ-UHFFFAOYSA-N	13.59	275.0	cr to l	4
C7H9N	NURQLCJSMXZBPC-UHFFFAOYSA-N	8.04	262.1	cr,I to l	4
C7H9N	HPYNZHMRTTWQTB-UHFFFAOYSA-N	11.84	257.9	cr to l	4
C5H12O	AQIXEPGDORPWBJ-UHFFFAOYSA-N	9.08	204.2	unknown	[5, 6, 7]
C4H5NO2	KZNICNPSHKQLFF-UHFFFAOYSA-N	16.10	401.0	unknown	[5, 6, 7]
C7H5BrO2	VOIZNVUXCQLQHS-UHFFFAOYSA-N	23.11	429.9	unknown	[5, 6, 7]
C8H8O3	XHQZJYCNDZAGLW-UHFFFAOYSA-N	23.23	377.0	unknown	[5, 6, 7]

Continued on next page

Continued from previous page

Formula	InChIKey	Δh_i^{fus} KJ/mol	T_i^{fus}/K	Phase	Source
C9H11Br	MOZHUOIQYVYEPN-UHFFFAOYSA-N	8.79	250.7	cr to l	4
C7H5BrO2	TUXYZHVUPGXXQG-UHFFFAOYSA-N	30.16	526.9	unknown	[5, 6, 7]
C8H10O	JJCKHVUTVOPLBV-UHFFFAOYSA-N	13.56	264.3	unknown	[5, 6, 7]
C12H11N	DMBHHRLKUKUOEG-UHFFFAOYSA-N	16.97	327.1	cr to l	4
C8H8ClNO	MUUQHCOAOLLHIL-UHFFFAOYSA-N	18.10	349.8	cr to l	4
C14H12	PJANXHGTPQOBST-UHFFFAOYSA-N	30.20	397.1	cr to l	4
C8H10O	KMTDMTZBNYGUNX-UHFFFAOYSA-N	20.17	331.9	unknown	[5, 6, 7]
C6H12O	PFCHFHIRKBAQGU-UHFFFAOYSA-N	13.49	217.7	cr,I to l	4
C8H18	CHBAWFGIXDBEBT-UHFFFAOYSA-N	10.84	152.2	cr to l	4
C7H16O	YVBCULSIZWMTFY-UHFFFAOYSA-N	8.30	235.1	unknown	[5, 6, 7]
C8H18	LAIUFBWHERIJIH-UHFFFAOYSA-N	11.37	152.7	cr to l	4
C8H16	QRMPKOFEUHIBNM-UHFFFAOYSA-N	9.31	195.7	cr to l	4
C7H9N	XWKFPIODWVPXLX-UHFFFAOYSA-N	11.76	257.6	cr to l	4
C4H6	QNRMTGGDHLBXQZ-UHFFFAOYSA-N	6.96	137.0	cr to l	4
C7H16	CXOWYJMDMMMMJO-UHFFFAOYSA-N	5.86	148.1	cr to l	4
C8H16	QEGNUYASOUJEHD-UHFFFAOYSA-N	2.03	239.8	cr,I to l	4
C7H14O	VTBOTOBFGSVRMA-UHFFFAOYSA-N	12.59	299.3	unknown	[5, 6, 7]
C8H18	FLTJDUOFAQWHDF-UHFFFAOYSA-N	6.80	152.0	cr to l	4
C6H6BrN	DHYHYLGCQVVLOQ-UHFFFAOYSA-N	14.68	291.0	unknown	[5, 6, 7]
C7H9N	HWWYDZCSSYKIAD-UHFFFAOYSA-N	12.58	266.7	cr to l	4
C6H7NO	CWLKGDAVCFYWJK-UHFFFAOYSA-N	24.70	394.2	cr to l	4
C6H4Cl2O	VPOMSPZBQMDLTM-UHFFFAOYSA-N	20.51	341.0	cr to l	4
C7H12	CTMHWPIWNRWQEG-UHFFFAOYSA-N	8.30	152.8	cr to l	4
C5H8O3	JOOXCMJARBKPKM-UHFFFAOYSA-N	9.22	306.1	cr to l	4
C7H16	GXDHCNNESPLIKD-UHFFFAOYSA-N	8.87	154.0	cr to l	4
C6H12O	QQZOPKMRPOGIEB-UHFFFAOYSA-N	14.90	217.7	cr to l	4
C5H8	QYZLKGVUSQXAMU-UHFFFAOYSA-N	6.14	124.3	cr to l	4
C5H8	LVMTVPFRTKXRPH-UHFFFAOYSA-N	7.56	135.9	cr to l	4
C8H18	UWNADWZGEHDQAB-UHFFFAOYSA-N	12.85	182.0	cr to l	4
C8H18	JVSWJIKNEAIKJW-UHFFFAOYSA-N	10.26	164.1	cr to l	4
C5H12N2O	CNWSQCLBDWYLAN-UHFFFAOYSA-N	14.55	369.3	cr,I to l	4
C5H11NO2	SKKTUOZKZKCGTB-UHFFFAOYSA-N	17.45	326.2	unknown	[5, 6, 7]
C6H12	LIKMAJRDDDTEIG-UHFFFAOYSA-N	9.34	133.4	cr to l	4
C6H12	ZQDPJFUHLCOCRG-UHFFFAOYSA-N	11.08	159.7	cr to l	4
C6H8	MGNZXYYWBUKAII-UHFFFAOYSA-N	4.21	161.0	cr to l	4
C7H14	ZGEGCLOFRBLKSE-UHFFFAOYSA-N	12.66	153.4	cr,I to l	4
C18H38	RZJRJXONCZWCBN-UHFFFAOYSA-N	60.50	300.9	cr to l	4
C27H56	BJQWYEJQWHSSCJ-UHFFFAOYSA-N	60.42	332.0	cr,I to l	4
C2H3Br	INLLPKCGLOXCIV-UHFFFAOYSA-N	5.12	133.6	cr to l	4
C4H8	VNXBKJFUJUWOCW-UHFFFAOYSA-N	2.80	95.8	cr to l	4
C3H6Cl2	ZEOVXNVKXIPWMS-UHFFFAOYSA-N	3.31	238.5	cr,I to l	4
C4H8O3	BWLBGMIXKSTLSX-UHFFFAOYSA-N	17.60	348.0	unknown	[5, 6, 7]
C4H9NO2	AIMREYQYBFBEGQ-UHFFFAOYSA-N	2.58	298.9	unknown	[5, 6, 7]
C3H6ClNO2	JQYFSFNSNVRUPY-UHFFFAOYSA-N	1.34	251.6	cr,I to l	4
C8H18	OMMLUKLXGSRPHK-UHFFFAOYSA-N	7.11	377.1	cr,I to l	4
C7H16O	XKIRHOWVQWCYBT-UHFFFAOYSA-N	9.50	259.4	unknown	[5, 6, 7]
C5H8	PAKGDPSCXSUALC-UHFFFAOYSA-N	7.80	159.5	cr to l	4
C2H6N2O	XGEGHDBEHXKFPX-UHFFFAOYSA-N	13.97	375.3	cr to l	4
C4H10O	RMGHERXMTMUMMV-UHFFFAOYSA-N	5.85	127.9	cr to l	4
C2H5NO2	GTCAXTIRRLKXRU-UHFFFAOYSA-N	16.30	328.6	cr to l	4
CH3NO3	LRMHVVPPGGOAJQ-UHFFFAOYSA-N	8.24	190.2	cr to l	4
C3H8N2O	YBBLOADPFWKNGS-UHFFFAOYSA-N	22.97	455.2	cr to l	4
C15H16O	QBDSZLJBMIMQRS-UHFFFAOYSA-N	21.68	346.4	cr to l	4
C5H8O2	TZMFJUDUGYTVRY-UHFFFAOYSA-N	7.84	221.2	unknown	[5, 6, 7]
C7H16O	BAYAKMPRFGNNFW-UHFFFAOYSA-N	5.90	260.7	unknown	[5, 6, 7]
C7H6N2O4	DYSXLQBUUOPLBB-UHFFFAOYSA-N	17.57	329.8	cr to l	4
C20H14	LUBXLGUQZVKOFP-UHFFFAOYSA-N	25.50	427.6	cr to l	4
C14H9NO2	LSIKFJXEYJIZNB-UHFFFAOYSA-N	20.10	420.4	unknown	[5, 6, 7]

Continued on next page

Continued from previous page

Formula	InChIKey	Δh_i^{fus} KJ/mol	T_i^{fus}/K	Phase	Source
C18H15P	RIOQSEWOXXDEQQ-UHFFFAOYSA-N	19.69	354.4	cr to l	4
C9H9N	BLRHMMGNCXNXJL-UHFFFAOYSA-N	10.90	276.5	unknown	[5, 6, 7]
C16H12	IYDMICQAKLQHLA-UHFFFAOYSA-N	15.55	297.5	unknown	[5, 6, 7]
C14H8Cl2	FKDIWXZNKAZCBY-UHFFFAOYSA-N	26.25	485.8	unknown	[5, 6, 7]
C7H6N2O4	XTRDKALNCIHHNI-UHFFFAOYSA-N	16.10	339.2	cr to l	4
C6H3ClN2O4	BPPMIQPXQVIZNJ-UHFFFAOYSA-N	18.95	361.2	unknown	[5, 6, 7]
C9H6O2	UHKAJLSKXBADFT-UHFFFAOYSA-N	21.80	401.5	unknown	[5, 6, 7]
C13H9NO2	XFOHWECQTFIEIX-UHFFFAOYSA-N	24.75	429.9	unknown	[5, 6, 7]
C6HBr5O	SVHOVVJFOWGYJO-UHFFFAOYSA-N	21.75	489.4	unknown	[5, 6, 7]
C6HCl5	CEOCDNVZRAIOQZ-UHFFFAOYSA-N	20.60	357.7	cr to l	4
C8H18	DUPUVYJQZSLSJB-UHFFFAOYSA-N	11.31	158.1	cr to l	4
C7H4N2O7	LWFUFLREGJMOIZ-UHFFFAOYSA-N	35.72	445.6	unknown	[5, 6, 7]
C7H6N2O3	KLGQWSOYKYFBTR-UHFFFAOYSA-N	27.75	446.0	unknown	[5, 6, 7]
C7H6N2O4	INYDMNPNDHRJQJ-UHFFFAOYSA-N	18.83	329.5	cr to l	4
C9H12	HYFLWBNQFMXCPA-UHFFFAOYSA-N	9.96	193.3	cr,I to l	4
C10H9N	JRLTTZUODKEYDH-UHFFFAOYSA-N	10.73	246.9	unknown	[5, 6, 7]
C9H11NO	IMNDHOCGZLYMRO-UHFFFAOYSA-N	21.22	316.9	unknown	[5, 6, 7]
C14H14	BSZXAFXFTLXUFV-UHFFFAOYSA-N	17.60	255.1	cr to l	4
C24H18	SXWIAEOZZQADEY-UHFFFAOYSA-N	33.40	446.0	cr to l	4
C16H12	TURIHPLQSRVWHU-UHFFFAOYSA-N	20.26	374.2	unknown	[5, 6, 7]
C14H12	WPDAVTSOEQEGMS-UHFFFAOYSA-N	23.84	382.2	cr to l	4
C8H9NO	NCCHARWOCKOHIH-UHFFFAOYSA-N	18.62	347.4	unknown	[5, 6, 7]
C7H8N2O	WARCRYXKINZHGQ-UHFFFAOYSA-N	25.70	388.2	unknown	[5, 6, 7]
C9H8O	UMJJFEIKYGFCAT-UHFFFAOYSA-N	16.89	330.0	unknown	[5, 6, 7]
C8H8N2	LDZYRENCLPUXAX-UHFFFAOYSA-N	20.49	451.4	unknown	[5, 6, 7]
C6H6BrN	AOPBDRUWRLBSDB-UHFFFAOYSA-N	18.09	304.6	unknown	[5, 6, 7]
C3H6O3	IEJIGPNLZYLLBP-UHFFFAOYSA-N	11.74	277.9	unknown	[5, 6, 7]
C5H6S	QENGPZGAWFQWCZ-UHFFFAOYSA-N	10.54	204.2	cr to l	4
C4H7NO	HNJBEVLQSNELDL-UHFFFAOYSA-N	13.92	299.1	cr to l	4
C6H10O4	AQYCMVICBNBXNA-UHFFFAOYSA-N	30.26	349.0	unknown	[5, 6, 7]
C7H16	AORMDLNPRGXHHL-UHFFFAOYSA-N	9.46	154.3	cr to l	4
C6H4ClNO3	BOFRXDMCQRTGII-UHFFFAOYSA-N	20.88	380.7	unknown	[5, 6, 7]
C7H4N2O2	RUSAWEHOGCWOPG-UHFFFAOYSA-N	20.49	389.7	unknown	[5, 6, 7]
C8H7BrO2	CZNGTXVOZOWWKM-UHFFFAOYSA-N	25.80	351.2	unknown	[5, 6, 7]
C8H6O3	GOUHYARYYWKXHS-UHFFFAOYSA-N	26.28	487.1	unknown	[5, 6, 7]
C9H11NO2	YDIYEOMDOWUDTJ-UHFFFAOYSA-N	34.10	512.5	unknown	[5, 6, 7]
C6H6O2	OUDFNZMQXZILJD-UHFFFAOYSA-N	11.56	240.5	unknown	[5, 6, 7]
C9H12	ZLCSFXXPPANWQY-UHFFFAOYSA-N	7.60	177.6	cr,I to l	4
C13H12O2	PXKLMJQFEQBVLD-UHFFFAOYSA-N	36.80	436.2	unknown	[5, 6, 7]
C8H9NO2	QLNWXBAGRTUKKI-UHFFFAOYSA-N	24.63	411.7	unknown	[5, 6, 7]
C8H8O3	JVTZFYYHCGSXJV-UHFFFAOYSA-N	31.83	385.9	unknown	[5, 6, 7]
C8H14O2	YYLLIJHXUHJATK-UHFFFAOYSA-N	5.69	223.0	unknown	[5, 6, 7]
C9H12	JRLPEMVDPFPYPJ-UHFFFAOYSA-N	12.70	210.8	cr to l	4
C7H4ClN	GJNGXPDXRVXSEH-UHFFFAOYSA-N	18.50	363.5	unknown	[5, 6, 7]
C8H8Cl2	ZZHIDJWUJRKHGX-UHFFFAOYSA-N	23.95	373.1	cr to l	4
C8H4N2	BHXFKXOIODIUJO-UHFFFAOYSA-N	22.90	499.0	unknown	[5, 6, 7]
C8H6O2	KUCOHFSKRZZVRO-UHFFFAOYSA-N	24.13	387.3	unknown	[5, 6, 7]
C5H10O2	UUIQMZJEGPQKFD-UHFFFAOYSA-N	11.36	190.1	unknown	[5, 6, 7]
C5H12N2O	ZWAVGZYKJNOTPX-UHFFFAOYSA-N	13.10	385.0	cr to l	4
C6H12O2	HNBDRPTVWVGKBR-UHFFFAOYSA-N	12.60	182.5	cr to l	4
C6H8O4	LDCRTTXIJACKKU-UHFFFAOYSA-N	14.64	254.0	cr to l	4
C4H8	IAQRGUVFOMOMEM-UHFFFAOYSA-N	9.86	167.3	cr to l	4
C2H3NO	HAMGRBXTJNITHG-UHFFFAOYSA-N	7.45	178.5	unknown	[5, 6, 7]
C3H8S	WXEHBUMAEPOYKP-UHFFFAOYSA-N	9.30	167.2	cr to l	4
C2H6S2	WQOXQRCZOLPYPM-UHFFFAOYSA-N	9.19	188.4	cr to l	4
C6H14O	DUXCSEISVMREAX-UHFFFAOYSA-N	9.54	235.7	unknown	[5, 6, 7]
C3H8N2O	RYECOJGRJDOGPP-UHFFFAOYSA-N	13.94	367.8	cr to l	4
C2H5NO3	IDNUEBSJWINEMI-UHFFFAOYSA-N	8.53	178.6	cr to l	4

Continued on next page

Continued from previous page

Formula	InChIKey	Δh_i^{fus} KJ/mol	T_i^{fus}/K	Phase	Source
C6H14S	XYWDPYKBIRQXQS-UHFFFAOYSA-N	10.80	195.1	cr to l	4
C6H9N	PAPNRQCYSFBWDI-UHFFFAOYSA-N	9.22	279.9	cr to l	4
C8H4N2	LAQPNDIUHRHNCV-UHFFFAOYSA-N	24.29	433.5	unknown	[5, 6, 7]
C6H5Cl2N	UQRLKWGPEVNVHT-UHFFFAOYSA-N	19.30	323.2	unknown	[5, 6, 7]
C6H10O4	XJMMNTGIMDZPMU-UHFFFAOYSA-N	29.20	357.3	unknown	[5, 6, 7]
C5H11NO	IPWFJLQDVFKJDU-UHFFFAOYSA-N	17.90	377.2	unknown	[5, 6, 7]
C6H13NO	ALBYIUDWACNRRB-UHFFFAOYSA-N	19.53	373.1	unknown	[5, 6, 7]
C5H12O	CXBDYQVECUFKRK-UHFFFAOYSA-N	10.85	157.5	cr to l	4
C5H12S	WCXXISMIJBRDQK-UHFFFAOYSA-N	11.50	175.3	cr to l	4
C5H12O	NVJUHMXYKCUMQA-UHFFFAOYSA-N	8.40	145.7	cr to l	4
C6H8	UVJHQYIOXKWHFD-UHFFFAOYSA-N	5.72	224.0	cr,I to l	4
C8H18O	SZNYYWIUQFZLLT-UHFFFAOYSA-N	11.33	190.4	unknown	[5, 6, 7]
C7H12	ZXIJMRYMVAMXQP-UHFFFAOYSA-N	0.82	217.6	cr,I to l	4
C6H12N2O2	GVNWZKBFMFUVNX-UHFFFAOYSA-N	52.72	499.1	unknown	[5, 6, 7]
C18H36O2	XIRNKXNNONJFQO-UHFFFAOYSA-N	53.45	297.2	unknown	[5, 6, 7]
C7H15Br	LSXKDWGTSHCFPP-UHFFFAOYSA-N	21.80	217.1	cr to l	4
C6H14O2	XXMIOPMDWAUFGU-UHFFFAOYSA-N	25.50	320.6	cr to l	4
C6H14S2	ALVPFGSHPUPROW-UHFFFAOYSA-N	5.60	187.6	cr to l	4
C8H8	KDUIUFJBNGTBMD-UHFFFAOYSA-N	11.27	268.5	cr to l	4
C13H28	IIYFAKIEWZDVMP-UHFFFAOYSA-N	28.55	267.8	cr,I to l	4
C14H30	BGHCVCJVXZWKCC-UHFFFAOYSA-N	45.93	278.6	cr to l	4
C15H32	YCOZIPAWZNQLMR-UHFFFAOYSA-N	46.97	283.1	cr,I to l	4
C16H32	GQEZCXVZFLOKMC-UHFFFAOYSA-N	28.30	277.1	cr,I to l	4
C15H32O	REIUXOLGHVXAEO-UHFFFAOYSA-N	30.35	316.9	cr,I to l	4
C17H36	NDJKXXJCMXVBJW-UHFFFAOYSA-N	39.10	295.1	cr,I to l	4
C19H40	LQERIDTXQFOHKA-UHFFFAOYSA-N	39.40	304.9	cr,I to l	4
C21H44	FNAZRRHPUDJQCJ-UHFFFAOYSA-N	55.60	313.5	cr,I to l	4
C20H42O	BTFJIXJJCSYFAL-UHFFFAOYSA-N	41.80	338.1	cr,I to l	4
C22H46	HOWGUJZVBDQJKV-UHFFFAOYSA-N	49.00	317.0	cr,I to l	4
C25H52	YKNWIILGEFFOPE-UHFFFAOYSA-N	57.74	326.7	cr,I to l	4
C26H54	HMSWAIKSFDFLKN-UHFFFAOYSA-N	58.70	329.3	cr,I to l	4
C28H58	ZYURHZPYMFLWSH-UHFFFAOYSA-N	62.89	333.9	cr,I to l	4
C29H60	IGGUPRCHHJZPBS-UHFFFAOYSA-N	66.11	336.6	cr,I to l	4
C5H9N	JAMNHZBIQDNHMM-UHFFFAOYSA-N	9.29	292.1	cr,I to l	4
C5H10O	FJJYHTVHBVXEEQ-UHFFFAOYSA-N	2.52	272.1	cr,I to l	4
C25H20	PEQHIRFAKIASBK-UHFFFAOYSA-N	48.28	554.2	unknown	[5, 6, 7]
C5H12N2O	AVQQQNCBBIEMEU-UHFFFAOYSA-N	14.10	272.0	cr to l	4
C9H12O3	CRUILBNAQILVHZ-UHFFFAOYSA-N	20.20	318.3	unknown	[5, 6, 7]
C6H2Cl4	GBDZXPJXOMHESU-UHFFFAOYSA-N	17.00	320.0	cr to l	4
C6H2Cl4	QZYNWJQFTJXIRN-UHFFFAOYSA-N	19.00	323.9	cr to l	4
C8H8O3	HMMBJOWWRLZEMI-UHFFFAOYSA-N	11.88	343.5	unknown	[5, 6, 7]
C12H14O4	ONIHPYYWNBVMID-UHFFFAOYSA-N	24.60	316.4	cr to l	4
C6H8O2	DCZFGQYXRKMVFG-UHFFFAOYSA-N	11.32	351.6	cr,I to l	4
C6H8S	GWQOOADXMVQEFT-UHFFFAOYSA-N	8.19	210.6	cr to l	4
C13H26O2	SZHOJFHSIKHZHA-UHFFFAOYSA-N	34.20	315.1	cr,I to l	4
C23H48	FIGVVZUWCLSUEI-UHFFFAOYSA-N	58.60	320.5	cr,I to l	4
C30H62	JXTPJDDICSTXJX-UHFFFAOYSA-N	68.80	338.7	cr,I to l	4
C13H12	ALLIZEAXNXSFGD-UHFFFAOYSA-N	13.10	273.0	cr to l	4
C13H12	ZZLCFHIKESPLTH-UHFFFAOYSA-N	12.29	320.3	unknown	[5, 6, 7]
C5H10	QMMOXUPEWRXHJS-UHFFFAOYSA-N	8.34	133.0	cr to l	4
C3H6O2	WNXJIVFYUVYPPR-UHFFFAOYSA-N	6.57	175.9	cr,I to l	4
C19H38O2	ISYWECDDZWTKFF-UHFFFAOYSA-N	57.80	341.3	cr,I to l	4
C24H50	POOSGDOYLQNASK-UHFFFAOYSA-N	81.20	320.5	cr,I to l	4
C22H46O	NOPFSRXAKWQILS-UHFFFAOYSA-N	46.60	343.5	cr,I to l	4
C6N4	NLDYACGHTUPAQU-UHFFFAOYSA-N	24.31	473.7	unknown	[5, 6, 7]
C5H9NO	XUWHAWMETYGRKB-UHFFFAOYSA-N	10.50	311.9	cr to l	4
CH3Cl2OP	SCLFRABIDYGTAZ-UHFFFAOYSA-N	18.08	306.1	cr to l	4
C5F12	NJCBUSHGCBERSK-UHFFFAOYSA-N	6.80	147.8	unknown	[5, 6, 7]

Continued on next page

Continued from previous page

Formula	InChIKey	Δh_i^{fus} KJ/mol	T_i^{fus}/K	Phase	Source
C3F6O	VBZWSGALLODQNC-UHFFFAOYSA-N	8.38	147.7	cr to l	4
C6H12	WSSSPWUEQFSQQG-UHFFFAOYSA-N	4.93	118.9	unknown	[5, 6, 7]
C12H22O4	TVIDDXQYHWJXFK-UHFFFAOYSA-N	50.60	402.5	cr to l	4
C11H23Br	IKPSIIAXIDAQLG-UHFFFAOYSA-N	33.50	263.3	cr to l	4
C6H10	ATQUFXWBVZUTKO-UHFFFAOYSA-N	7.60	146.6	cr to l	4
C4H5NS	QMHIMXFNBOYPND-UHFFFAOYSA-N	8.90	229.1	unknown	[5, 6, 7]
C4H6N2	LXBGSDVWAMZHDD-UHFFFAOYSA-N	13.68	417.9	unknown	[5, 6, 7]
C5H5NO	ILVXOBCQQYKLDS-UHFFFAOYSA-N	9.68	337.0	unknown	[5, 6, 7]
C11H16	BEZDDPMMPIDMGJ-UHFFFAOYSA-N	12.34	327.5	cr,I to l	4
C12H20	CWNOIUTVJRWADX-UHFFFAOYSA-N	1.13	245.6	unknown	[5, 6, 7]
C11H13N3O3S	CYFLXLSBHQBMFT-UHFFFAOYSA-N	11.60	481.1	cr,I to l	4
C5H11Cl	JEKYMVBQWWZVHO-UHFFFAOYSA-N	1.81	250.5	unknown	[5, 6, 7]
C6F5OH	XBNGYFFABRKICK-UHFFFAOYSA-N	16.41	310.6	cr,I to l	4
C14H12	XXPBFNVKTVJZKF-UHFFFAOYSA-N	13.25	367.1	cr to l	4
C15H16	MILSYCKGLDDVLM-UHFFFAOYSA-N	18.00	302.3	cr to l	4
C2H2F4	LVGUZGTVOIAKKC-UHFFFAOYSA-N	2.01	169.4	unknown	[5, 6, 7]
C14H26O4	HQHCYKULIHKCEB-UHFFFAOYSA-N	56.70	397.7	unknown	[5, 6, 7]
C11H22	DCTOHCCUXLBQMS-UHFFFAOYSA-N	16.99	224.0	cr,I to l	4
C8H12N2O2	RRAMGCGOFNQTLD-UHFFFAOYSA-N	18.64	206.1	cr to l	4
C12H16	IGARGHRYKHJQSM-UHFFFAOYSA-N	16.00	280.2	cr to l	4
C11H16O2	JIMXXGFJRDUSRO-UHFFFAOYSA-N	16.90	448.1	unknown	[5, 6, 7]
C12H22O	SXVPOSFURRDKBO-UHFFFAOYSA-N	16.85	335.6	unknown	[5, 6, 7]
C12H10O2	VGKONPUVOVVNSU-UHFFFAOYSA-N	20.21	319.2	unknown	[5, 6, 7]
C12H10O2	ZSBDGXGICLIJGD-UHFFFAOYSA-N	24.50	356.7	unknown	[5, 6, 7]
C15H12	LOCGAKKLRVLQAM-UHFFFAOYSA-N	14.04	324.9	cr,I to l	4
C9H17N5S	RQVYBGPQFYCBGX-UHFFFAOYSA-N	26.00	359.1	unknown	[5, 6, 7]
C10H22	SGVYKUFIHHTIFL-UHFFFAOYSA-N	17.49	198.8	cr to l	4
C10H20	AFFLGGQVNFXPEV-UHFFFAOYSA-N	13.81	206.9	cr,I to l	4
C5H9NO	SECXISVLQFMRJM-UHFFFAOYSA-N	14.57	248.8	unknown	[5, 6, 7]
C7H4ClN	NHWQMJMIYICNBP-UHFFFAOYSA-N	15.40	323.2	unknown	[5, 6, 7]
C6H6N2O	MTFJSAGADRTKCI-UHFFFAOYSA-N	19.98	388.0	unknown	[5, 6, 7]
C10H14	MEMBJMDZWKVOTB-UHFFFAOYSA-N	12.94	210.3	cr to l	4
C11H11N	JJPSZKIOGBRMHK-UHFFFAOYSA-N	20.40	330.8	unknown	[5, 6, 7]
C12H10S2	GUUVPOWQJOLRAS-UHFFFAOYSA-N	27.99	331.9	unknown	[5, 6, 7]
C9H4Cl4O4	SXINVWXSZUQKSW-UHFFFAOYSA-N	30.10	450.5	cr to l	4
C16H9NO2	PIHGQKMEAMSUNA-UHFFFAOYSA-N	22.60	435.0	unknown	[5, 6, 7]
C11H22O	ZPQAKYPOZRXKFA-UHFFFAOYSA-N	30.04	288.3	unknown	[5, 6, 7]
C12H27N	JRBPAEWTRLWTQC-UHFFFAOYSA-N	118.30	298.2	Crystal	[8]
C8H14	URYYVOIYTNXXBN-UHFFFAOYSA-N	1.81	259.2	cr,I to l	4
C10H14	QUBBAXISAHIDNM-UHFFFAOYSA-N	13.66	206.2	cr to l	4
C7H7N3	JWYUFVNJZUSCSM-UHFFFAOYSA-N	24.70	505.1	cr to l	4
C7H5NOS	YEDUAINPPJYDJZ-UHFFFAOYSA-N	19.60	410.8	unknown	[5, 6, 7]
C10H14	SBUYFICWQNHBCM-UHFFFAOYSA-N	12.06	206.2	cr to l	4
C11H9N	JVZRCNQLWOELDU-UHFFFAOYSA-N	19.95	346.9	unknown	[5, 6, 7]
C12H13N	XKLNOVWDVMWTOB-UHFFFAOYSA-N	17.85	391.3	unknown	[5, 6, 7]
C12H23NO	JHWNWJKBPDFINM-UHFFFAOYSA-N	16.10	425.3	unknown	[5, 6, 7]
C14H11N	KLLLJCACIRKBDT-UHFFFAOYSA-N	25.62	461.7	unknown	[5, 6, 7]
C12H9N3O4	RHTVQEPJVKUMPI-UHFFFAOYSA-N	14.37	431.4	unknown	[5, 6, 7]
CH5O3P	YACKEPLHDIMKIO-UHFFFAOYSA-N	10.41	382.1	unknown	[5, 6, 7]
C15H30O2	WQEPLUUGTLDZJY-UHFFFAOYSA-N	42.70	325.8	cr,I to l	4
C4H3BrS	TUCRZHGAIRVWTI-UHFFFAOYSA-N	7.58	203.9	cr to l	4
C6H7NO	IWYYIZOHWPCALJ-UHFFFAOYSA-N	18.90	457.8	unknown	[5, 6, 7]
C14H22	OOWNNCMFKFBNOF-UHFFFAOYSA-N	21.90	350.8	cr to l	4
C8H15N5S	MGLWZSOBALDPEK-UHFFFAOYSA-N	24.00	353.2	unknown	[5, 6, 7]
C8H18	GIEZWIDCIFCQPS-UHFFFAOYSA-N	10.83	182.3	cr to l	4
C9H20	BGXXXYLRPIRDHJ-UHFFFAOYSA-N	10.03	240.1	cr,I to l	4
C9H20	VLHAGZNBWKUMRW-UHFFFAOYSA-N	7.20	150.8	cr,I to l	4
C9H15NO5	ISOLMABRZPQKOV-UHFFFAOYSA-N	34.30	368.7	unknown	[5, 6, 7]

Continued on next page

Continued from previous page

Formula	InChIKey	Δh_i^{fus} KJ/mol	T_i^{fus}/K	Phase	Source
C9H20	GUMULFRCHLJNDY-UHFFFAOYSA-N	9.74	206.6	cr to l	4
C10H22	HXQDUXXBVMMIKL-UHFFFAOYSA-N	9.83	260.6	cr to l	4
C6H7NO	IGJQUJNPMOYEJY-UHFFFAOYSA-N	14.08	363.0	unknown	[5, 6, 7]
C10H14	YQZBFMJOASEONC-UHFFFAOYSA-N	14.77	212.8	cr to l	4
C10H14	QUEBYVKXYIKVSO-UHFFFAOYSA-N	10.57	190.6	cr to l	4
C10H14	JXFVMNFKABWTHD-UHFFFAOYSA-N	11.49	209.5	cr to l	4
C11H12O2	MNALUTYMBUBKNX-UHFFFAOYSA-N	22.80	351.3	unknown	[5, 6, 7]
C11H24	RSJKGSCJYJTIGS-UHFFFAOYSA-N	22.32	247.2	cr,I to l	4
C21H42O2	QGBRLVONZXHAKJ-UHFFFAOYSA-N	74.00	319.2	unknown	[5, 6, 7]
C3H6O3S	FSSPGSAQUIYDCN-UHFFFAOYSA-N	9.96	304.3	cr to l	4
C8H12N2	FINHMKGKINIASC-UHFFFAOYSA-N	18.91	358.1	unknown	[5, 6, 7]
C5H4N2O3	RXKNNAKAVAHBNK-UHFFFAOYSA-N	17.48	434.0	unknown	[5, 6, 7]
C12H12	ZMXIYERNXPIYFR-UHFFFAOYSA-N	16.32	259.3	cr to l	4
C13H9Br	FXSCJZNMWILAJO-UHFFFAOYSA-N	16.52	384.9	unknown	[5, 6, 7]
C13H10O2	NPFYZDNDJHZQKY-UHFFFAOYSA-N	24.40	407.7	unknown	[5, 6, 7]
C24H18	OWPJBAYCIXEHFA-UHFFFAOYSA-N	27.70	360.0	unknown	[5, 6, 7]
C6H14O	XRMVWAKMXZNZIL-UHFFFAOYSA-N	4.42	233.6	unknown	[5, 6, 7]
C4H6O	SHQSVMDWKBRBGB-UHFFFAOYSA-N	10.80	220.5	unknown	[5, 6, 7]
C6H6O2	IEMMBWWQXVXBEU-UHFFFAOYSA-N	15.50	301.6	unknown	[5, 6, 7]
C8H9NO2	CSEWAUGPAQPMDC-UHFFFAOYSA-N	42.70	468.2	unknown	[5, 6, 7]
C15H9N	KEQZHLAEKAVZLY-UHFFFAOYSA-N	25.19	445.2	unknown	[5, 6, 7]
C13H8Br3NO2	KVSKGMLNBAPGKH-UHFFFAOYSA-N	28.70	499.6	cr to l	4
C6H6N2O	VFQXVTODMYMSMJ-UHFFFAOYSA-N	24.12	429.3	unknown	[5, 6, 7]
C17H36O	GOQYKNQRPGWPLP-UHFFFAOYSA-N	50.03	327.0	unknown	[5, 6, 7]
C9H20S	ZVEZMVFBMOOHAT-UHFFFAOYSA-N	33.50	267.7	unknown	[5, 6, 7]
C10H10O4	VNGOYPQMJFJDLV-UHFFFAOYSA-N	25.30	340.7	cr to l	4
C3H6N2	OAGOUCJGXNLJNL-UHFFFAOYSA-N	12.50	232.1	cr to l	4
C26H18	FCNCGHJSNVOIKE-UHFFFAOYSA-N	30.60	523.3	unknown	[5, 6, 7]
C4H10S	ROSSIHMZZJOVOU-UHFFFAOYSA-N	10.50	171.7	cr to l	4
C6H12S	CMKBCTPCXZNQKX-UHFFFAOYSA-N	10.00	189.6	cr to l	4
C9H8O3	FEIOASZZURHTHB-UHFFFAOYSA-N	21.40	335.2	unknown	[5, 6, 7]
C5H8	PMJHHCWVYXUKFD-UHFFFAOYSA-N	7.14	185.7	cr to l	4
C6H12N2O3	NOQGZXFMHARMLW-UHFFFAOYSA-N	41.00	425.8	cr to l	4
C5H10S	YPWFISCTZQNZAU-UHFFFAOYSA-N	2.45	292.3	cr,I to l	4
C5H12O	BZLVMXJERCGZMT-UHFFFAOYSA-N	7.60	164.6	cr to l	4
C14H16	URGSMJLDEFDWNX-UHFFFAOYSA-N	25.10	253.4	cr to l	4
C7H14	QWHNJUXXYKPLQM-UHFFFAOYSA-N	1.08	203.7	cr,I to l	4
C7H16S	VPIAKHNXCOTPAY-UHFFFAOYSA-N	25.50	229.9	cr to l	4
C7H14	IFTRQJLVEBNKJK-UHFFFAOYSA-N	6.86	134.7	cr to l	4
C12H15NO3	IMWZZHHPURKASS-UHFFFAOYSA-N	28.35	395.3	unknown	[5, 6, 7]
C8H16	IIEWJVIFRVWJOD-UHFFFAOYSA-N	8.33	161.8	cr to l	4
C9H18	DEDZSLCZHWTGOR-UHFFFAOYSA-N	10.37	178.3	cr to l	4
C10H20	GGBJHURWWWLEQH-UHFFFAOYSA-N	14.16	198.4	cr to l	4
C5H10S	WVDYBOADDMMFIY-UHFFFAOYSA-N	7.83	155.4	cr to l	4
C7H3Br2NO	UPMXNNIRAGDFEH-UHFFFAOYSA-N	31.80	463.6	cr to l	4
C10H8ClN3O	WYKYKTKDBLFHCY-UHFFFAOYSA-N	26.60	480.2	cr to l	4
C3H7NO3	GAPFWGOSHOCNBM-UHFFFAOYSA-N	10.01	190.8	cr to l	4
C10H22	DIOQZVSQGTUSAI-UHFFFAOYSA-N	28.78	243.1	cr to l	4
C10H20O2	IJXHLVMUNBOGRR-UHFFFAOYSA-N	27.60	238.8	cr to l	4
C18H36O2	HUEBIMLTDXKIPR-UHFFFAOYSA-N	48.10	304.2	unknown	[5, 6, 7]
C5H10N2	MTPJEFOSTIKRSS-UHFFFAOYSA-N	12.20	228.9	cr to l	4
C12H4Cl4O2	HGUFODBRKLSHSI-UHFFFAOYSA-N	38.90	578.2	unknown	[5, 6, 7]
C9H11ClN2O2	LKJPSUCKSLORMF-UHFFFAOYSA-N	22.50	353.9	cr to l	4
C5H10S	AJPGNQYBSTXCJE-UHFFFAOYSA-N	8.88	172.4	cr to l	4
C16H32	STWFZICHPLEOIC-UHFFFAOYSA-N	38.64	271.4	cr to l	4
C12H10O2	IMHDGJOMLMDPJN-UHFFFAOYSA-N	25.36	386.7	unknown	[5, 6, 7]
C12H7Cl2NO3	XITQUSLLOSKDTB-UHFFFAOYSA-N	26.40	344.7	cr to l	4
C8Cl4N2	CRQQGFGUEAVUIL-UHFFFAOYSA-N	31.40	526.2	cr to l	4

Continued on next page

Continued from previous page

**Formula**	**InChIKey**	Δh_i^{fus} **KJ/mol**	T_i^{fus}/K	**Phase**	**Source**
C8H7Cl2NO2	WOZQBERUBLYCEG-UHFFFAOYSA-N	23.20	382.7	cr to l	4
C6H3Cl4N	DCUJJWWUNKIJPH-UHFFFAOYSA-N	20.30	337.2	cr to l	4
C10H14O2	BGNXCDMCOKJUMV-UHFFFAOYSA-N	27.74	350.9	unknown	[5, 6, 7]
C15H15ClN2O2	IVUXTESCPZUGJC-UHFFFAOYSA-N	38.90	425.1	cr to l	4
C14H31N	PLZVEHJLHYMBBY-UHFFFAOYSA-N	134.50	298.2	Crystal	[8]
C13H14	TVYVQNHYIHAJTD-UHFFFAOYSA-N	15.90	288.3	cr,I to l	4
C11H16N2O2	IMIDOCRTMDIQIJ-UHFFFAOYSA-N	26.80	368.2	cr to l	4
C11H15NO2S	YFBPRJGDJKVWAH-UHFFFAOYSA-N	30.10	394.8	cr to l	4
C9H18	ZAGHKONXGGSVDV-UHFFFAOYSA-N	11.31	165.2	cr to l	4
C8H16	KDIAMAVWIJYWHN-UHFFFAOYSA-N	10.03	155.8	cr to l	4
C12H8Cl2	YTBRNEUEFCNVHC-UHFFFAOYSA-N	22.80	421.1	unknown	[5, 6, 7]
C12H9Cl	FPWNLURCHDRMHC-UHFFFAOYSA-N	13.32	348.6	cr to l	4
C6H16N2	NAQMVNRVTILPCV-UHFFFAOYSA-N	41.20	312.7	unknown	[5, 6, 7]
C6H10Cl2	LQRDJCBZCLUBRY-UHFFFAOYSA-N	1.47	236.0	unknown	[5, 6, 7]
C13H22N2O	YGLMVCVJLXREAK-UHFFFAOYSA-N	21.70	441.9	cr to l	4
C13H18N2O2	ZTMKADLOSYKWCA-UHFFFAOYSA-N	42.30	584.6	cr to l	4
C10H11F3N2O	RZILCCPWPBTYDO-UHFFFAOYSA-N	29.70	434.0	cr to l	4
C5Cl5N	DNDPLEAVNVOOQZ-UHFFFAOYSA-N	27.70	398.2	unknown	[5, 6, 7]
C14H22	CDKDZKXSXLNROY-UHFFFAOYSA-N	29.96	234.2	unknown	[5, 6, 7]
C9H20	SEEOMASXHIJCDV-UHFFFAOYSA-N	16.99	165.6	unknown	[5, 6, 7]
C11H16O	BKZXZGWHTRCFPX-UHFFFAOYSA-N	17.32	302.5	unknown	[5, 6, 7]
C13H18ClNO	WGVWLKXZBUVUAM-UHFFFAOYSA-N	16.30	355.9	cr to l	4
C9H8O4	GDBUZIKSJGRBJP-UHFFFAOYSA-N	28.43	466.0	unknown	[5, 6, 7]
C10H10N2	FKHNZQFCDGOQGV-UHFFFAOYSA-N	22.35	379.5	unknown	[5, 6, 7]
C11H16O	IKEHOXWJQXIQAG-UHFFFAOYSA-N	19.40	325.5	cr to l	4
C9H12O	QQOMQLYQAXGHSU-UHFFFAOYSA-N	22.05	331.2	unknown	[5, 6, 7]
C10H9Cl4NO2S	JHRWWRDRBPCWTF-UHFFFAOYSA-N	43.10	432.0	cr to l	4
C10H18O	WUOACPNHFRMFPN-UHFFFAOYSA-N	15.78	308.5	unknown	[5, 6, 7]
C10H6N2OS2	FBQQHUGEACOBDN-UHFFFAOYSA-N	29.90	443.7	cr to l	4
C7H7NO3	PIIZYNQECPTVEO-UHFFFAOYSA-N	27.41	401.0	cr to l	4
C12H17NO2	DTAPQAJKAFRNJB-UHFFFAOYSA-N	23.00	362.5	cr to l	4
C11H15NO2	QBSJMKIUCUGGNG-UHFFFAOYSA-N	26.10	369.9	cr to l	4
C7H5NOS	DMSMPAJRVJJAGA-UHFFFAOYSA-N	20.87	428.8	unknown	[5, 6, 7]
C12H10O2S	VWGKEVWFBOUAND-UHFFFAOYSA-N	31.04	432.9	unknown	[5, 6, 7]
C8H8Cl2O2	PFIADAMVCJPXSF-UHFFFAOYSA-N	30.50	404.4	cr to l	4
C4H8N8O8	UZGLIIJVICEWHF-UHFFFAOYSA-N	69.87	553.2	unknown	[5, 6, 7]
C10H9Cl2NO	PLQDLOBGKJCDSZ-UHFFFAOYSA-N	25.10	403.4	cr to l	4
C5H12N2S	MNOILHPDHOHILI-UHFFFAOYSA-N	20.17	350.7	unknown	[5, 6, 7]
C8H9NO3	FDGAEAYZQQCBRN-UHFFFAOYSA-N	25.34	477.9	unknown	[5, 6, 7]
C8H15NO2	JKNCOURZONDCGV-UHFFFAOYSA-N	16.85	237.7	cr to l	4
C15H12N2O3	TUXJTJITXCHUEL-UHFFFAOYSA-N	35.29	515.2	unknown	[5, 6, 7]
C9H11BrN2O2	WLFDQEVORAMCIM-UHFFFAOYSA-N	25.50	369.2	cr to l	4
C4H2Cl2S	FGYBDASKYMSNCX-UHFFFAOYSA-N	11.93	232.1	unknown	[5, 6, 7]
C6H3Cl2NO2	CMVQZRLQEOAYSW-UHFFFAOYSA-N	22.31	334.9	unknown	[5, 6, 7]
C9H20	ZUBZATZOEPUUQF-UHFFFAOYSA-N	17.99	192.8	unknown	[5, 6, 7]
C12Cl8O2	FOIBFBMSLDGNHL-UHFFFAOYSA-N	40.30	596.7	unknown	[5, 6, 7]
C8H10N2O4S	VGPYEHKOIGNJKV-UHFFFAOYSA-N	27.20	417.3	cr to l	4
C6H4ClN3O4	LHRIICYSGQGXSX-UHFFFAOYSA-N	22.65	434.2	unknown	[5, 6, 7]
C9H9NO3	QCXJEYYXVJIFCE-UHFFFAOYSA-N	38.30	533.5	unknown	[5, 6, 7]
C6H8O3	INAXVXBDKKUCGI-UHFFFAOYSA-N	19.00	354.5	unknown	[5, 6, 7]
C19H14	GOHBXWHNJHENRX-UHFFFAOYSA-N	19.00	390.7	unknown	[5, 6, 7]
C4H6O2	LDHQCZJRKDOVOX-UHFFFAOYSA-N	13.81	333.7	cr to l	4
C10H5Cl9	OCHOKXCPKDPNQU-UHFFFAOYSA-N	26.40	400.6	cr to l	4
C8H9O3PS	OUNSASXJZHBGAI-UHFFFAOYSA-N	16.90	329.5	cr to l	4
C4H10S	ZOASGOXWEHUTKZ-UHFFFAOYSA-N	8.50	160.2	cr to l	4
C8H8ClNO2	ZAGNMMRDHSEOPE-UHFFFAOYSA-N	21.81	362.7	cr to l	4
C6H12	RYPKRALMXUUNKS-UHFFFAOYSA-N	8.26	140.2	cr to l	4
C5H12S	ZDDDFDQTSXYYSE-UHFFFAOYSA-N	10.50	156.1	cr to l	4

Continued on next page

Continued from previous page

Formula	InChIKey	Δh_i^{fus} KJ/mol	T_i^{fus}/K	Phase	Source
C12H24	QHWAQXOSHHKCFK-UHFFFAOYSA-N	20.53	224.9	unknown	[5, 6, 7]
C7H12O2	VUXKVKAHWOVIDN-UHFFFAOYSA-N	10.49	201.3	unknown	[5, 6, 7]
C10H12O4	IAJBQAYHSQIQRE-UHFFFAOYSA-N	30.59	395.8	unknown	[5, 6, 7]
C11H10O	PBLNHHSDYFYZNC-UHFFFAOYSA-N	15.57	333.0	unknown	[5, 6, 7]
C12H13NO2S	GYSSRZJIHXQEHQ-UHFFFAOYSA-N	22.18	371.5	cr to l	4
C12H13NO4S	AMEKQAFGQBKLKX-UHFFFAOYSA-N	26.70	403.2	cr to l	4
C8H14O2	LJOODBDWMQKMFB-UHFFFAOYSA-N	13.80	302.6	unknown	[5, 6, 7]
C24H14	JHOWUOKQHJHGMU-UHFFFAOYSA-N	24.00	505.5	unknown	[5, 6, 7]
C6H4ClN3O4	CLMQUEQFVUMDPC-UHFFFAOYSA-N	105.20	298.2	Crystal	[8]
C8H3NO5	ROFZMKDROVBLNY-UHFFFAOYSA-N	19.35	435.3	unknown	[5, 6, 7]
C10H12O2	NJGBTKGETPDVIK-UHFFFAOYSA-N	23.73	356.3	unknown	[5, 6, 7]
C16H9NO2	ALRLPDGCPYIVHP-UHFFFAOYSA-N	18.90	425.9	unknown	[5, 6, 7]
C9H11Cl3NO4P	OTMOUPHCTWPNSL-UHFFFAOYSA-N	15.60	314.3	cr to l	4
C9H13ClN2O2	NBQCNZYJJMBDKY-UHFFFAOYSA-N	12.51	448.0	unknown	[5, 6, 7]
C10H22	PLZDDPSCZHRBOY-UHFFFAOYSA-N	18.70	188.5	cr to l	4
C10H16	XMGQYMWWDOXHJM-UHFFFAOYSA-N	11.38	199.2	unknown	[5, 6, 7]
C5H12O	JYVLIDXNZAXMDK-UHFFFAOYSA-N	8.48	200.0	unknown	[5, 6, 7]
C10H13ClN2	STUSTWKEFDQFFZ-UHFFFAOYSA-N	20.60	305.8	cr to l	4
C6H5ClN2O2	KWIXNFOTNVKIGM-UHFFFAOYSA-N	27.03	393.4	unknown	[5, 6, 7]
C5H2Cl3NO	WCYYAQFQZQEUEN-UHFFFAOYSA-N	25.80	447.9	cr to l	4
C6H6O2S	RCNOGGGBSSVMAS-UHFFFAOYSA-N	18.80	353.3	unknown	[5, 6, 7]
C11H24	CNPVJWYWYZMPDS-UHFFFAOYSA-N	25.09	224.3	cr to l	4
C11H13NO4	SDKQRNRRDYRQKY-UHFFFAOYSA-N	23.80	388.3	cr to l	4
C12H7Cl3	BZTYNSQSZHARAZ-UHFFFAOYSA-N	19.70	329.3	unknown	[5, 6, 7]
C10H20	QTYARKOMFKRPSY-UHFFFAOYSA-N	8.07	178.4	unknown	[5, 6, 7]
C16H32O2	XIUXKAZJZFLLDQ-UHFFFAOYSA-N	13.81	380.3	cr to l	4
C12H22S	FTAORUVBXKFVDA-UHFFFAOYSA-N	5.68	284.2	unknown	[5, 6, 7]
C8H7Cl2NO2	DTSSCQVCVYZGSI-UHFFFAOYSA-N	16.30	335.1	cr to l	4
C11H24O	JPEWDCTZJFUITH-UHFFFAOYSA-N	31.72	243.4	cr to l	4
C18H11NO2	UAWLTQJFZUYROA-UHFFFAOYSA-N	28.40	486.6	unknown	[5, 6, 7]
C13H12O	CDMGNVWZXRKJNS-UHFFFAOYSA-N	20.20	307.0	unknown	[5, 6, 7]
C8H20N2	PWGJDPKCLMLPJW-UHFFFAOYSA-N	51.30	325.1	unknown	[5, 6, 7]
C9H10O3	MWSMNBYIEBRXAL-UHFFFAOYSA-N	25.80	345.4	unknown	[5, 6, 7]
C10H13NO2	VXPLXMJHHKHSOA-UHFFFAOYSA-N	23.01	357.7	cr to l	4
C9H14ClN5	OOHIAOSLOGDBCE-UHFFFAOYSA-N	28.80	443.5	cr to l	4
C16H16N2O4	WZJZMXBKUWKXTQ-UHFFFAOYSA-N	32.70	394.8	cr to l	4
C16H16N2O4	IDOWTHOLJBTAFI-UHFFFAOYSA-N	42.30	423.9	cr to l	4
C6H12O6	GZCGUPFRVQAUEE-UHFFFAOYSA-N	39.01	435.1	unknown	[5, 6, 7]
C11H15NO2	FZUGPQWGEGAKET-UHFFFAOYSA-N	20.76	336.8	unknown	[5, 6, 7]
C12H8Cl2	JAYCNKDKIKZTAF-UHFFFAOYSA-N	16.60	330.9	unknown	[5, 6, 7]
C20H12O	SPUUWWRWIAEPDB-UHFFFAOYSA-N	24.10	469.6	unknown	[5, 6, 7]
C9H10BrClN2O2	NLYNUTMZTCLNOO-UHFFFAOYSA-N	27.60	370.3	cr to l	4
C10H22	RMQHJMMCLSJULX-UHFFFAOYSA-N	12.38	219.2	cr to l	4
C8H7ClO3	SODPIMGUZLOIPE-UHFFFAOYSA-N	36.30	431.5	cr to l	4
C3H6N6O3	HFWOSHMLDRSIDN-UHFFFAOYSA-N	3.77	376.0	cr,I to l	4
C13H7F3N2O5	HHMCAJWVGYGUEF-UHFFFAOYSA-N	18.40	364.9	cr to l	4
C6H15P	RXJKFRMDXUJTEX-UHFFFAOYSA-N	10.73	188.2	unknown	[5, 6, 7]
C10H22	TYSIILFJZXHVPU-UHFFFAOYSA-N	16.64	186.7	cr to l	4
C2H6ClO3P	UDPGUMQDCGORJQ-UHFFFAOYSA-N	14.80	349.9	cr to l	4
C9H20	CLZCPQKGOAXOJT-UHFFFAOYSA-N	10.20	173.7	cr,I to l	4
C7H10ClN3O3	IPWKIXLWTCNBKN-UHFFFAOYSA-N	21.38	358.6	unknown	[5, 6, 7]
C10H22	IALRSQMWHFKJJA-UHFFFAOYSA-N	15.19	174.7	cr to l	4
C20H13NO4	MHXFWEJMQVIWDH-UHFFFAOYSA-N	30.79	458.2	unknown	[5, 6, 7]
C12H24O6	XEZNGIUYQVAUSS-UHFFFAOYSA-N	37.21	312.3	unknown	[5, 6, 7]
C19H38	NHLUYCJZUXOUBX-UHFFFAOYSA-N	34.90	296.5	unknown	[5, 6, 7]
C8H7ClO3	ONIVKFDMLVBDRK-UHFFFAOYSA-N	39.40	442.6	unknown	[5, 6, 7]
C10H13ClN2O2	DSRNRYQBBJQVCW-UHFFFAOYSA-N	27.50	399.5	cr to l	4
C10H4Cl4	NAQWICRLNQSPPW-UHFFFAOYSA-N	11.54	470.8	unknown	[5, 6, 7]

Continued on next page

Continued from previous page

Formula	InChIKey	Δh_i^{fus} KJ/mol	T_i^{fus}/K	Phase	Source
C13H10S	NICUQYHIOMMFGV-UHFFFAOYSA-N	90.30	298.2	Crystal	[8]
C8H14N4OS	FOXFZRUHNHCZPX-UHFFFAOYSA-N	18.00	399.9	cr to l	4
C6H6N2O	DBZAKQWXICEWNW-UHFFFAOYSA-N	21.21	347.9	unknown	[5, 6, 7]
C9H10O2	FQYUMYWMJTYZTK-UHFFFAOYSA-N	17.32	279.8	unknown	[5, 6, 7]
C12H12N2	YBQZXXMEJHZYMB-UHFFFAOYSA-N	17.63	407.1	cr to l	4
C8H8O2	IPBVNPXQWQGGJP-UHFFFAOYSA-N	12.80	266.0	unknown	[5, 6, 7]
C18H30	KWKXNDCHNDYVRT-UHFFFAOYSA-N	43.10	274.6	unknown	[5, 6, 7]
C7H14O	HCFAJYNVAYBARA-UHFFFAOYSA-N	16.16	240.2	unknown	[5, 6, 7]
C2H5NO	ATHHXGZTWNVVOU-UHFFFAOYSA-N	10.44	270.6	unknown	[5, 6, 7]
C5H8O2	YRKCREAYFQTBPV-UHFFFAOYSA-N	14.50	254.8	cr to l	4
C6H12O3	SQYNKIJPMDEDEG-UHFFFAOYSA-N	13.83	285.8	cr,I to l	4
C4H9N	RWRDLPDLKQPQOW-UHFFFAOYSA-N	8.52	215.3	cr,I to l	4
C6H12O2	DKPFZGUDAPQIHT-UHFFFAOYSA-N	14.59	196.1	cr to l	4
C4H8O2	RYHBNJHYFVUHQT-UHFFFAOYSA-N	11.88	283.2	cr,I to l	4
C22H44O2	ULBTUVJTXULMLP-UHFFFAOYSA-N	37.48	299.7	cr,I to l	4
C16H32O2	MMKRHZKQPFCLLS-UHFFFAOYSA-N	45.00	287.4	unknown	[5, 6, 7]
C15H30O2	ZAZKJZBWRNNLDS-UHFFFAOYSA-N	50.20	292.1	cr to l	4
C9H18	JRZJOMJEPLMPRA-UHFFFAOYSA-N	19.08	191.8	cr,I to l	4
C9H18O	GYHFUZHODSMOHU-UHFFFAOYSA-N	30.50	253.8	cr to l	4
C2Br2F4	KVBKAPANDHPRDG-UHFFFAOYSA-N	7.04	162.8	cr,I to l	4
C5H12O2	SLCVBVWXLSEKPL-UHFFFAOYSA-N	4.71	398.6	cr,I to l	4
C4H8O2S	HXJUTPCZVOIRIF-UHFFFAOYSA-N	3.74	296.7	unknown	[5, 6, 7]
C2Cl4	CYTYCFOTNPOANT-UHFFFAOYSA-N	10.56	250.6	cr,I to l	4
C4H9NO	FXHOOIRPVKKKFG-UHFFFAOYSA-N	10.06	252.8	unknown	[5, 6, 7]
C12H10O2S	KZTYYGOKRVBIMI-UHFFFAOYSA-N	21.29	399.5	unknown	[5, 6, 7]
C15H24O	NLZUEZXRPGMBCV-UHFFFAOYSA-N	22.50	343.0	cr to l	4
C14H22O	DKCPKDPYUFEZCP-UHFFFAOYSA-N	16.57	310.7	unknown	[5, 6, 7]
C14H10N2O2	FBMQNRKSAWNXBT-UHFFFAOYSA-N	24.20	484.2	unknown	[5, 6, 7]
C16H10	BBEAQIROQSPTKN-UHFFFAOYSA-N	17.36	423.8	cr,I to l	4
C7H13N3O3S	KZAUOCCYDRDERY-UHFFFAOYSA-N	30.20	373.0	cr to l	4
C12H11Cl2NO	PHNUZKMIPFFYSO-UHFFFAOYSA-N	28.70	429.7	cr to l	4
C16H20	GWLLTEXUIOFAFE-UHFFFAOYSA-N	18.84	343.0	unknown	[5, 6, 7]
C9H10O2	WVWZECQNFWFVFW-UHFFFAOYSA-N	12.49	228.8	unknown	[5, 6, 7]
C13H14	HMAMGXMFMCAOPV-UHFFFAOYSA-N	17.57	264.6	cr to l	4
C15H12N2O2	CTRLABGOLIVAIY-UHFFFAOYSA-N	33.22	491.1	unknown	[5, 6, 7]
C14H10O	MNQGEQSXFDKAPY-UHFFFAOYSA-N	22.00	357.6	unknown	[5, 6, 7]
C9H18	ODNRTOSCFYDTKF-UHFFFAOYSA-N	9.80	229.9	cr to l	4
C12H5Cl5	IUTPYMGCWINGEY-UHFFFAOYSA-N	26.60	382.3	unknown	[5, 6, 7]
C12H6Cl4	RKLLTEAEZIJBAU-UHFFFAOYSA-N	27.90	398.4	unknown	[5, 6, 7]
C12H6Cl4	KENZYIHFBRWMOD-UHFFFAOYSA-N	27.50	376.7	unknown	[5, 6, 7]
C12H6Cl4	UQMGJOKDKOLIDP-UHFFFAOYSA-N	34.50	451.2	unknown	[5, 6, 7]
C7H6Cl2O	SSNXYMVLSOMJLU-UHFFFAOYSA-N	23.68	310.5	unknown	[5, 6, 7]
C12H4Cl6	ICOAEPDGFWLUTI-UHFFFAOYSA-N	17.50	386.7	cr to l	4
C12H18N2O	PUIYMUZLKQOUOZ-UHFFFAOYSA-N	33.90	430.8	cr to l	4
C12H8Cl2	UFNIBRDIUNVOMX-UHFFFAOYSA-N	18.00	316.7	unknown	[5, 6, 7]
C12H4Cl6	MVWHGTYKUMDIHL-UHFFFAOYSA-N	22.90	375.3	unknown	[5, 6, 7]
C12H3Cl7	WBHQEUPUMONIKF-UHFFFAOYSA-N	24.80	383.4	unknown	[5, 6, 7]
C12H3Cl7	RMPWIIKNWPVWNG-UHFFFAOYSA-N	26.90	412.2	unknown	[5, 6, 7]
C14H9ClF2N2O2	QQQYTWIFVNKMRW-UHFFFAOYSA-N	56.00	501.2	cr to l	4
C10H22O2	FOTKYAAJKYLFFN-UHFFFAOYSA-N	41.70	345.4	cr to l	4
C12H6Cl4	HCWZEPKLWVAEOV-UHFFFAOYSA-N	20.80	357.1	unknown	[5, 6, 7]
C12H4Cl6	CKLLRBPBZLTGDJ-UHFFFAOYSA-N	21.50	352.6	unknown	[5, 6, 7]
C12H7Cl3	DCMURXAZTZQAFB-UHFFFAOYSA-N	18.90	316.6	unknown	[5, 6, 7]
C12H5Cl5	LAHWLEDBADHJGA-UHFFFAOYSA-N	18.80	350.1	cr to l	4
C12H5Cl5	GXNNLIMMEXHBKV-UHFFFAOYSA-N	23.30	367.7	unknown	[5, 6, 7]
C12H5Cl5	ARXHIJMGSIYYRZ-UHFFFAOYSA-N	16.20	322.4	unknown	[5, 6, 7]
C12H4Cl6	LKHLFUVHHXCNJH-UHFFFAOYSA-N	20.20	348.8	unknown	[5, 6, 7]
C12H4Cl6	BTAGRXWGMYTPBY-UHFFFAOYSA-N	29.20	424.9	cr to l	4

Continued on next page

Continued from previous page

Formula	InChIKey	Δh_i^{fus} KJ/mol	T_i^{fus}/K	Phase	Source
C12H4Cl6	FZFUUSROAHKTTF-UHFFFAOYSA-N	21.10	385.2	cr to l	4
C12H3Cl7	ZDLMBNHYTPHDLF-UHFFFAOYSA-N	24.40	400.5	unknown	[5, 6, 7]
C12H7Cl3	ZMHWQAHZKUPENF-UHFFFAOYSA-N	20.20	344.0	unknown	[5, 6, 7]
C12H7Cl3	RIMXLXBUOQMDHV-UHFFFAOYSA-N	19.50	332.3	unknown	[5, 6, 7]
C12Cl8O	RHIROFAGUQOFLU-UHFFFAOYSA-N	28.00	526.3	unknown	[5, 6, 7]
C12H6Cl4	ALDJIKXAHSDLLB-UHFFFAOYSA-N	18.30	320.4	unknown	[5, 6, 7]
C12H6Cl4	ZWPVHELAQPIZHO-UHFFFAOYSA-N	23.40	339.1	cr to l	4
C9H7N3S	DQJCHOQLCLEDLL-UHFFFAOYSA-N	24.10	460.5	cr to l	4
C14H9Cl2NO5	SUSRORUBZHMPCO-UHFFFAOYSA-N	26.30	359.3	cr to l	4
C15H11ClF3NO4	OQMBBFQZGJFLBU-UHFFFAOYSA-N	30.10	359.8	cr to l	4
C10H11F3N2O3S	CUYDUAXYJJPSRK-UHFFFAOYSA-N	40.50	456.3	cr to l	4
C12H9Cl2NO3	FSCWZHGZWWDELK-UHFFFAOYSA-N	27.50	383.7	cr to l	4
C14H9Cl	SRIHSAFSOOUEGL-UHFFFAOYSA-N	14.14	355.2	unknown	[5, 6, 7]
C12H20N4O2	CAWXEEYDBZRFPE-UHFFFAOYSA-N	20.40	390.4	cr to l	4
C12H3Cl7	TZMHVHLTPWKZCI-UHFFFAOYSA-N	20.30	395.4	cr to l	4
C14H18O4	GLEVLJDDWXEYCO-UHFFFAOYSA-N	38.70	465.5	unknown	[5, 6, 7]
C11H13F3N2O3S	OKIBNKKYNPBDRS-UHFFFAOYSA-N	37.70	457.5	cr to l	4
C7H4Cl3NO3	REEQLXCGVXDJSQ-UHFFFAOYSA-N	31.20	423.9	cr to l	4
C12H9N3O5S	YQNQNVDNTFHQSW-UHFFFAOYSA-N	52.85	475.0	unknown	[5, 6, 7]
C8H9NO4S	VSOOBQALJVLTBH-UHFFFAOYSA-N	30.20	398.2	unknown	[5, 6, 7]
C8H8Cl3N3O4S2	QOVTVIYTBRHADL-UHFFFAOYSA-N	33.00	471.2	unknown	[5, 6, 7]
C14H7ClF3NO5	NUFNQYOELLVIPL-UHFFFAOYSA-N	37.67	436.6	unknown	[5, 6, 7]
C20H11NO2	NMMAFYSZGOFZCM-UHFFFAOYSA-N	30.20	528.4	unknown	[5, 6, 7]
C6H10N6	LVQDKIWDGQRHTE-UHFFFAOYSA-N	24.10	491.7	unknown	[5, 6, 7]
C14H9Cl3N2OS	NQPDXQQQCQDHHW-UHFFFAOYSA-N	35.13	446.6	unknown	[5, 6, 7]
C4H7NO	OOWFYDWAMOKVSF-UHFFFAOYSA-N	12.00	210.2	cr to l	4
C9H20O2	DSKYSDCYIODJPC-UHFFFAOYSA-N	20.80	317.3	unknown	[5, 6, 7]
C9H18O2	OKJADYKTJJGKDX-UHFFFAOYSA-N	17.60	189.4	cr to l	4
C9H10O3	PFYHAAAQPNMZHO-UHFFFAOYSA-N	16.60	276.4	unknown	[5, 6, 7]
C14H12O4	GYUVMLBYMPKZAZ-UHFFFAOYSA-N	38.40	464.4	cr to l	4
C6H9NS	BAMPVSWRQZNDQC-UHFFFAOYSA-N	9.00	240.7	unknown	[5, 6, 7]
C3H6O2S	PMNLUUOXGOOLSP-UHFFFAOYSA-N	16.97	291.9	cr to l	4
C12H15N3O6	XMWRWTSZNLOZFN-UHFFFAOYSA-N	22.03	386.8	unknown	[5, 6, 7]
C8H10O3	ZENOXNGFMSCLLL-UHFFFAOYSA-N	30.74	389.0	unknown	[5, 6, 7]
C9H12O2	AYJXHIDNNLJQDT-UHFFFAOYSA-N	7.20	297.3	unknown	[5, 6, 7]
C12H5Cl5	REHONNLQRWTIFF-UHFFFAOYSA-N	27.10	430.7	unknown	[5, 6, 7]
C8H6N2O2	SDQJTWBNWQABLE-UHFFFAOYSA-N	128.30	298.2	Crystal	[8]
C10H18O	DTGKSKDOIYIVQL-UHFFFAOYSA-N	7.95	480.5	unknown	[5, 6, 7]
C7H8N2O	ZYVXHFWBYUDDBM-UHFFFAOYSA-N	22.00	379.1	unknown	[5, 6, 7]
C11H8N2	AIFRHYZBTHREPW-UHFFFAOYSA-N	25.50	471.5	unknown	[5, 6, 7]
C7H7NO2	QXSAKPUBHTZHKW-UHFFFAOYSA-N	25.30	433.5	unknown	[5, 6, 7]
C14H12N2	IYRGXJIJGHOCFS-UHFFFAOYSA-N	17.60	435.9	unknown	[5, 6, 7]
C13H13N	GTWJETSWSUWSEJ-UHFFFAOYSA-N	16.75	309.1	cr to l	4
C5H8N2	PQAMFDRRWURCFQ-UHFFFAOYSA-N	8.90	358.7	unknown	[5, 6, 7]
C5H5ClN2	MAXBVGJEFDMHNV-UHFFFAOYSA-N	27.86	407.7	unknown	[5, 6, 7]
C14H18	LFAYMJXHGYUQNV-UHFFFAOYSA-N	17.91	346.0	cr,I to l	4
C9H11N	YQDGQEKUTLYWJU-UHFFFAOYSA-N	9.08	222.6	cr to l	4
C8H9NO2	ZVIDMSBTYRSMAR-UHFFFAOYSA-N	21.00	435.9	unknown	[5, 6, 7]
C12H12O3	NKRISXMDKXBVRJ-UHFFFAOYSA-N	25.81	387.5	unknown	[5, 6, 7]
C10H11NO2	ASLSUMISAQDOOB-UHFFFAOYSA-N	24.72	336.8	unknown	[5, 6, 7]
C3H6O3	GSJFXBNYJCXDGI-UHFFFAOYSA-N	11.40	272.8	unknown	[5, 6, 7]
C6H8	NFWSQSCIDYBUOU-UHFFFAOYSA-N	6.86	130.2	cr to l	4
C9H11NO2	NEGFNJRAUMCZMY-UHFFFAOYSA-N	21.80	423.3	unknown	[5, 6, 7]
C8H18N2O2	VARKIGWTYBUWNT-UHFFFAOYSA-N	25.90	405.0	cr to l	4
C12H8N2	OZKOMUDCMCEDTM-UHFFFAOYSA-N	25.60	351.7	unknown	[5, 6, 7]
C13H10S	PQJUJGAVDBINPI-UHFFFAOYSA-N	26.10	401.7	cr to l	4
CH2N4	KJUGUADJHNHALS-UHFFFAOYSA-N	18.40	430.7	cr,I to l	4
C14H28	KATXJJSCAPBIOB-UHFFFAOYSA-N	9.30	329.3	cr,I to l	4

Continued on next page

Continued from previous page

Formula	InChIKey	Δh_i^{fus} KJ/mol	T_i^{fus}/K	Phase	Source
C16H32	JJWIOXUMXIOXQN-UHFFFAOYSA-N	7.60	333.7	cr,I to l	4
C10F8	JDCMOHAFGDQQJX-UHFFFAOYSA-N	17.55	358.8	unknown	[5, 6, 7]
C2F3N	SFFUEHODRAXXIA-UHFFFAOYSA-N	4.97	128.7	cr to l	4
C2H2F3NO	NRKYWOKHZRQRJR-UHFFFAOYSA-N	1.34	388.9	unknown	[5, 6, 7]
C5H3F7O2	MRPUVAKBXDBGJQ-UHFFFAOYSA-N	11.77	191.4	cr to l	4
C7HF13O2	ZWBAMYVPMDSJGQ-UHFFFAOYSA-N	18.00	295.7	unknown	[5, 6, 7]
C5F10	PWMJXZJISGDARB-UHFFFAOYSA-N	2.99	283.5	unknown	[5, 6, 7]
C12F10	ONUFSRWQCKNVSL-UHFFFAOYSA-N	20.50	339.6	unknown	[5, 6, 7]
C7H6FNO	KGGHWIKBOIQEAJ-UHFFFAOYSA-N	23.80	388.5	unknown	[5, 6, 7]
C10H10N2	YFOOEYJGMMJJLS-UHFFFAOYSA-N	16.15	339.8	unknown	[5, 6, 7]
C8H6S2	OHZAHWOAMVVGEL-UHFFFAOYSA-N	16.50	304.2	unknown	[5, 6, 7]
C14H8Br2	BRUOAURMAFDGLP-UHFFFAOYSA-N	27.85	497.9	unknown	[5, 6, 7]
C10H12O2	WAMKWBHYPYBEJY-UHFFFAOYSA-N	18.47	384.5	unknown	[5, 6, 7]
C7H6O3	LUSZGTFNYDARNI-UHFFFAOYSA-N	16.96	337.7	unknown	[5, 6, 7]
C4H9NO	WFKAJVHLWXSISD-UHFFFAOYSA-N	19.24	400.8	unknown	[5, 6, 7]
C10H8N2	RMHQDKYZXJVCME-UHFFFAOYSA-N	17.40	332.8	unknown	[5, 6, 7]
C7H11N3O3	DUOHVNSMLSPTMI-UHFFFAOYSA-N	25.65	333.0	unknown	[5, 6, 7]
C8H18O2S	AIDFJGKWTOULTC-UHFFFAOYSA-N	30.90	316.2	unknown	[5, 6, 7]
C14H14O	GIMDPFBLSKQRNP-UHFFFAOYSA-N	26.49	357.9	unknown	[5, 6, 7]
C8H5NO6	KFIRODWJCYBBHY-UHFFFAOYSA-N	55.30	496.4	unknown	[5, 6, 7]
C6H5Br2N	XIRRDAWDNHRRLB-UHFFFAOYSA-N	21.79	355.5	unknown	[5, 6, 7]
C7H6ClNO	RBGDLYUEXLWQBZ-UHFFFAOYSA-N	21.30	410.6	unknown	[5, 6, 7]
C7H7NO3	JKTYGPATCNUWKN-UHFFFAOYSA-N	20.97	336.4	unknown	[5, 6, 7]
C9H12O3	LKUDPHPHKOZXCD-UHFFFAOYSA-N	28.00	329.0	unknown	[5, 6, 7]
C7H14O3	VUPKGFBOKBGHFZ-UHFFFAOYSA-N	17.50	215.4	unknown	[5, 6, 7]
C8H17NO	LTHCSWBWNVGEFE-UHFFFAOYSA-N	25.43	377.2	unknown	[5, 6, 7]
C8H18O2	OEIJHBUUFURJLI-UHFFFAOYSA-N	36.10	332.7	cr to l	4
C26H20	JLZUZNKTTIRERF-UHFFFAOYSA-N	37.45	496.1	unknown	[5, 6, 7]
C14H14O8	QVEIFJBUBJUUMB-UHFFFAOYSA-N	35.70	418.0	cr to l	4
C18H30	ROXLYQQDLJJEBE-UHFFFAOYSA-N	19.60	393.0	unknown	[5, 6, 7]
C9H11N	LBUJPTNKIBCYBY-UHFFFAOYSA-N	11.81	289.9	cr,I to l	4
C7H7NO4	KXKCTSZYNCDFFG-UHFFFAOYSA-N	21.43	377.6	unknown	[5, 6, 7]
C15H10O	YMNKUHIVVMFOFO-UHFFFAOYSA-N	17.76	378.3	unknown	[5, 6, 7]
CH3F2OP	PQIOSYKVBBWRRI-UHFFFAOYSA-N	11.88	236.3	cr to l	4
C14H14	PQTAUFTUHHRKSS-UHFFFAOYSA-N	20.90	279.8	cr to l	4
C14H9Cl	KULLJOPUZUWTMF-UHFFFAOYSA-N	20.33	377.9	unknown	[5, 6, 7]
C3H7N	HTJDQJBWANPRPF-UHFFFAOYSA-N	13.18	237.8	cr to l	4
C15H14	PJQCANLCUDUPRF-UHFFFAOYSA-N	23.33	348.0	unknown	[5, 6, 7]
C3H6N2O5	IPLRZPREFHIGIB-UHFFFAOYSA-N	2.85	366.7	unknown	[5, 6, 7]
C4H6N2	UQFQONCQIQEYPJ-UHFFFAOYSA-N	11.40	229.2	unknown	[5, 6, 7]
C7H5BrO	ZRYZBQLXDKPBDU-UHFFFAOYSA-N	22.60	334.2	unknown	[5, 6, 7]
C18H13N	VIJYEGDOKCKUOL-UHFFFAOYSA-N	19.40	367.9	unknown	[5, 6, 7]
C5H10	FOTXAJDDGPYIFU-UHFFFAOYSA-N	5.60	123.6	cr to l	4
C7H12O2	YVHAIVPPUIZFBA-UHFFFAOYSA-N	12.55	284.7	unknown	[5, 6, 7]
C15H10O3	GPZYYYGYCRFPBU-UHFFFAOYSA-N	32.41	509.3	unknown	[5, 6, 7]
C11H20O2	YLCHTSSXNSNXSW-UHFFFAOYSA-N	18.32	222.4	unknown	[5, 6, 7]
C6H12O	CAKWRXVKWGUISE-UHFFFAOYSA-N	8.41	310.2	unknown	[5, 6, 7]
C13H10O4	OKJFKPFBSPZTAH-UHFFFAOYSA-N	31.30	482.6	unknown	[5, 6, 7]
C8H14	IFVMAGPISVKRAR-UHFFFAOYSA-N	9.70	163.2	cr to l	4
C3H5NO2	IZXIZTKNFFYFOF-UHFFFAOYSA-N	16.80	361.5	unknown	[5, 6, 7]
C14H9Br	ZIRVQSRSPDUEOJ-UHFFFAOYSA-N	19.20	375.9	unknown	[5, 6, 7]
C5H5N	STSRVFAXSLNLLI-UHFFFAOYSA-N	12.70	210.6	cr to l	4
C13H10O	AFMVESZOYKHDBJ-UHFFFAOYSA-N	24.20	428.7	unknown	[5, 6, 7]
C11H20O2	MVOSYKNQRRHGKX-UHFFFAOYSA-N	12.61	275.3	cr,I to l	4
C16H16	MBAIEZXRGAOPKH-UHFFFAOYSA-N	18.09	407.6	cr,I to l	4
C16H6O7	QQGYZOYWNCKGEK-UHFFFAOYSA-N	35.77	501.8	unknown	[5, 6, 7]
C4H8N2O	NQPJDJVGBDHCAD-UHFFFAOYSA-N	30.60	523.0	unknown	[5, 6, 7]
C18H22	HGTUJZTUQFXBIH-UHFFFAOYSA-N	25.52	392.0	unknown	[5, 6, 7]

Continued on next page

Continued from previous page

**Formula**	**InChIKey**	Δh_i^{fus} **KJ/mol**	T_i^{fus}/K	**Phase**	**Source**
C15H24O2	FLLRQABPKFCXSO-UHFFFAOYSA-N	26.90	374.4	unknown	[5, 6, 7]
C11H12O3	IHMQOBPGHZFGLC-UHFFFAOYSA-N	25.50	392.4	unknown	[5, 6, 7]
C10H21NO	TUTWLYPCGCUWQI-UHFFFAOYSA-N	15.10	370.6	unknown	[5, 6, 7]
C6H5N3O	ASOKPJOREAFHNY-UHFFFAOYSA-N	20.05	424.3	unknown	[5, 6, 7]
C7H5NO4	SNWQAKNKGGOVMO-UHFFFAOYSA-N	28.20	420.2	unknown	[5, 6, 7]
C7H8N2O	QIKYZXDTTPVVAC-UHFFFAOYSA-N	30.17	455.5	unknown	[5, 6, 7]
C10H10O3	HGVWMTAIIYNQSI-UHFFFAOYSA-N	23.49	356.0	unknown	[5, 6, 7]
C4H8O	KTHXBEHDVMTNOH-UHFFFAOYSA-N	8.53	228.4	unknown	[5, 6, 7]
C13H12O	VKTQADPEPIVMHK-UHFFFAOYSA-N	19.10	325.4	unknown	[5, 6, 7]
C8H8O3	WWYFPDXEIFBNKE-UHFFFAOYSA-N	26.83	454.5	unknown	[5, 6, 7]
C6H4O5	CHTHALBTIRVDBM-UHFFFAOYSA-N	55.13	614.5	unknown	[5, 6, 7]
C7H7NO4	IZLVFLOBTPURLP-UHFFFAOYSA-N	21.69	374.4	unknown	[5, 6, 7]
C14H20O2	NOUZOVBGCDDMSX-UHFFFAOYSA-N	26.37	388.2	unknown	[5, 6, 7]
C8H9NO2	GUCPYIYFQVTFSI-UHFFFAOYSA-N	29.04	440.6	unknown	[5, 6, 7]
C4H8N2O6	QELUAJBXJAWSRC-UHFFFAOYSA-N	26.70	285.5	cr to l	4
C8H7NO4	WMUZDBZPDLHUMW-UHFFFAOYSA-N	26.86	412.8	unknown	[5, 6, 7]
C5H5N3O2	UGSBCCAHDVCHGI-UHFFFAOYSA-N	29.20	461.4	unknown	[5, 6, 7]
C10H10N2	KKKDZZRICRFGSD-UHFFFAOYSA-N	21.50	343.9	unknown	[5, 6, 7]
C8H8S	YJUFGFXVASPYFQ-UHFFFAOYSA-N	14.84	269.8	unknown	[5, 6, 7]
C10H12O4	UUQHKWMIDYRWHH-UHFFFAOYSA-N	28.40	416.5	unknown	[5, 6, 7]
C16H10O3	OUJCFCNZIUTYBH-UHFFFAOYSA-N	27.30	430.7	unknown	[5, 6, 7]
C11H14	UTTMVTDJCFSOFF-UHFFFAOYSA-N	11.99	227.4	cr to l	4
C6F15N	CBEFDCMSEZEGCX-UHFFFAOYSA-N	5.56	156.2	cr,I to l	4
C6H10O5	TWNIBLMWSKIRAT-UHFFFAOYSA-N	3.20	454.9	unknown	[5, 6, 7]
C10H15Br	VQHPRVYDKRESCL-UHFFFAOYSA-N	5.00	372.7	unknown	[5, 6, 7]
C19H40O	XGFDHKJUZCCPKQ-UHFFFAOYSA-N	57.86	334.2	unknown	[5, 6, 7]
C6H7NO2	FGBJXOREULPLGL-UHFFFAOYSA-N	12.86	243.2	unknown	[5, 6, 7]
C8H6Cl2O4	XYHBJALHMANCCC-UHFFFAOYSA-N	29.00	410.4	cr to l	4
C13H10O2	SHULEACXTONYPS-UHFFFAOYSA-N	27.40	390.5	unknown	[5, 6, 7]
C10H10O3	SMIOEQSLJNNKQF-UHFFFAOYSA-N	20.80	324.1	unknown	[5, 6, 7]
C8H10O3	MUTGBJKUEZFXGO-UHFFFAOYSA-N	7.78	307.2	unknown	[5, 6, 7]
C9H10ClN5O2	YWTYJOPNNQFBPC-UHFFFAOYSA-N	26.40	416.8	unknown	[5, 6, 7]
C8H8O3	YKUCHDXIBAQWSF-UHFFFAOYSA-N	20.12	344.3	unknown	[5, 6, 7]
C9H6O3	ZOXBWJMCXHTKNU-UHFFFAOYSA-N	19.25	361.5	unknown	[5, 6, 7]
C15H22O3	ZWQBZEFLFSFEOS-UHFFFAOYSA-N	22.92	437.5	unknown	[5, 6, 7]
C14H12O	XXSCONYSQQLHTH-UHFFFAOYSA-N	27.09	376.9	unknown	[5, 6, 7]
C14H9Cl5O	LUXSISXJGNCOKN-UHFFFAOYSA-N	25.20	397.1	cr to l	4
C12H13N3	ZLIBICFPKPWGIZ-UHFFFAOYSA-N	21.23	370.8	unknown	[5, 6, 7]
C8H10Cl2O2	LLMLSUSAKZVFOA-UHFFFAOYSA-N	15.04	338.2	unknown	[5, 6, 7]
C10H9NO2	GLNDAGDHSLMOKX-UHFFFAOYSA-N	32.09	499.9	unknown	[5, 6, 7]
C16H20	JDBFIFNXALGSOF-UHFFFAOYSA-N	28.60	320.7	unknown	[5, 6, 7]
C9H20	QUKOJKFJIHSBKV-UHFFFAOYSA-N	2.33	263.4	cr,I to l	4
C20H14	NGDCLPXRKSWRPY-UHFFFAOYSA-N	30.28	527.2	cr to l	4
C9H17N	POTIYWUALSJREP-UHFFFAOYSA-N	25.72	321.3	cr to l	4
C12H6Br4O	XYBSIYMGXVUVGY-UHFFFAOYSA-N	17.30	356.9	unknown	[5, 6, 7]
C8H7ClO3	RWOLDZZTBNYTMS-UHFFFAOYSA-N	22.13	361.7	unknown	[5, 6, 7]
C10H6F3NO2	JBNOVHJXQSHGRL-UHFFFAOYSA-N	31.70	494.8	unknown	[5, 6, 7]
C12H18O	GVNVAWHJIKLAGL-UHFFFAOYSA-N	19.70	280.8	cr to l	4
C8HF17	KBHBUUBXEQUIMV-UHFFFAOYSA-N	9.87	254.3	unknown	[5, 6, 7]
C12H10F3NO2	GZTMNDOZYLMFQE-UHFFFAOYSA-N	30.47	432.5	unknown	[5, 6, 7]
C12H10F3NO2	KDTAEYOYAZPLIC-UHFFFAOYSA-N	26.25	420.5	unknown	[5, 6, 7]
C8H18O	UPOMCDPCTBJJDA-UHFFFAOYSA-N	8.65	162.3	unknown	[5, 6, 7]
C6H9N3O2	LVFRCHIUUKWBLR-UHFFFAOYSA-N	29.85	371.0	unknown	[5, 6, 7]
C16H14N2O3S	LNPDTQAFDNKSHK-UHFFFAOYSA-N	30.35	446.4	unknown	[5, 6, 7]
C14H20	ZICQBHNGXDOVJF-UHFFFAOYSA-N	8.65	517.9	cr,I to l	4
C15H16	HKTCLPBBJDIBGF-UHFFFAOYSA-N	18.80	297.6	cr to l	4
C9H18	YWYCGTZNHWYQBD-UHFFFAOYSA-N	7.04	184.7	cr to l	4
C18H15ClN2O2S	MNJVRJDLRVPLFE-UHFFFAOYSA-N	29.55	409.5	unknown	[5, 6, 7]

Continued on next page

Continued from previous page

Formula	InChIKey	Δh_i^{fus} KJ/mol	T_i^{fus}/K	Phase	Source
C12H14N2O2	LYONXVJRBWWGQO-UHFFFAOYSA-N	25.83	391.8	unknown	[5, 6, 7]
C5H8	PODAMDNJNMAKAZ-UHFFFAOYSA-N	6.63	147.5	cr to l	4
C3H6N2O4	DHPRWWYQIUXCQM-UHFFFAOYSA-N	2.41	326.0	cr,I to l	4
C7H15NO	AEDIXYWIVPYNBI-UHFFFAOYSA-N	16.20	368.6	unknown	[5, 6, 7]
C3H5NO	MNFORVFSTILPAW-UHFFFAOYSA-N	17.50	349.2	unknown	[5, 6, 7]
C20H18	KIIBETRYVBIAOO-UHFFFAOYSA-N	24.39	328.2	unknown	[5, 6, 7]
C5H10N2O6	MIYIEPHJPVBSEV-UHFFFAOYSA-N	15.48	256.6	cr to l	4
C6H14S2	CLMXSBZIYJITQU-UHFFFAOYSA-N	9.90	206.4	cr to l	4
C5H12S	CJFVCTVYZFTORU-UHFFFAOYSA-N	8.45	190.9	cr to l	4
C9F16	NLQOEOVORMKOIY-UHFFFAOYSA-N	2.68	269.7	unknown	[5, 6, 7]
C9H10ClF3O2	SPVZAYWHHVLPBN-UHFFFAOYSA-N	33.37	383.3	unknown	[5, 6, 7]
C11H16N4O3	GDEAMEURJBBCOQ-UHFFFAOYSA-N	24.70	353.5	cr to l	4
C10H12ClN3O2	QCQPGRMMDFIQMB-UHFFFAOYSA-N	27.60	431.4	cr to l	4
C18H12Cl2N2O	WYEMLYFITZORAB-UHFFFAOYSA-N	37.20	419.9	unknown	[5, 6, 7]
C7H7NO2	OLXYLDUSSBULGU-UHFFFAOYSA-N	14.60	283.4	unknown	[5, 6, 7]
C17H10O	RCYFOPUXRMOLQM-UHFFFAOYSA-N	11.00	399.9	unknown	[5, 6, 7]
C15H28O2	FKUPPRZPSYCDRS-UHFFFAOYSA-N	6.98	308.5	cr,I to l	4
C6H6O3	NOEGNKMFWQHSLB-UHFFFAOYSA-N	19.80	308.5	unknown	[5, 6, 7]
C10H18O2	VZHUBBUZNIULNM-UHFFFAOYSA-N	16.57	219.6	unknown	[5, 6, 7]
C9H8O3	CWKXDPPQCVWXAG-UHFFFAOYSA-N	19.44	324.4	unknown	[5, 6, 7]
C12H9Cl	LAXBNTIAOJWAOP-UHFFFAOYSA-N	14.52	304.9	cr to l	4
C6H11FO5	ZCXUVYAZINUVJD-UHFFFAOYSA-N	38.20	427.2	unknown	[5, 6, 7]
C8H7NO	JYGFTBXVXVMTGB-UHFFFAOYSA-N	17.60	396.4	unknown	[5, 6, 7]
C6H8N2O2	OVBFMUAFNIIQAL-UHFFFAOYSA-N	20.76	231.2	unknown	[5, 6, 7]
C15H18	FDHDUXOBMHHFFJ-UHFFFAOYSA-N	23.40	248.6	cr to l	4
C5H12S	BFLXFRNPNMTTAA-UHFFFAOYSA-N	0.61	146.1	cr,I to l	4
C10H5Cl3	QEPTXDCPBXMWJC-UHFFFAOYSA-N	18.44	354.7	unknown	[5, 6, 7]
C6H3ClF3N	RXATZPCCMYMPME-UHFFFAOYSA-N	19.46	312.2	unknown	[5, 6, 7]
C14H14O2	HCNHNBLSNVSJTJ-UHFFFAOYSA-N	27.90	399.3	unknown	[5, 6, 7]
C7H10N2OS	KNAHARQHSZJURB-UHFFFAOYSA-N	40.86	493.1	unknown	[5, 6, 7]
C9H12N2S	VRDIULHPQTYCLN-UHFFFAOYSA-N	23.21	414.1	unknown	[5, 6, 7]
C7H5NS2	YXIWHUQXZSMYRE-UHFFFAOYSA-N	21.43	454.7	unknown	[5, 6, 7]
C7H6N2S	YHMYGUUIMTVXNW-UHFFFAOYSA-N	24.11	589.4	unknown	[5, 6, 7]
C7H5NOS	FLFWJIBUZQARMD-UHFFFAOYSA-N	22.60	468.3	unknown	[5, 6, 7]
C7H7ClN2S	YZUKKTCDYSIWKJ-UHFFFAOYSA-N	22.30	416.4	cr to l	4
C4H6N2S	PMRYVIKBURPHAH-UHFFFAOYSA-N	19.60	418.0	unknown	[5, 6, 7]
C3H4N2S2	FPVUWZFFEGYCGB-UHFFFAOYSA-N	26.10	457.3	unknown	[5, 6, 7]
C4H4N2O2S	RVBUGGBMJDPOST-UHFFFAOYSA-N	14.80	516.4	unknown	[5, 6, 7]
C3H6N2S	PDQAZBWRQCGBEV-UHFFFAOYSA-N	21.99	468.5	unknown	[5, 6, 7]
C3H5NS2	WGJCBBASTRWVJL-UHFFFAOYSA-N	16.80	377.0	unknown	[5, 6, 7]
CH4N2S	UMGDCJDMYOKAJW-UHFFFAOYSA-N	13.81	453.3	cr to l	4
C2H5NS	YUKQRDCYNOVPGJ-UHFFFAOYSA-N	18.40	386.4	cr to l	4
C5H12N2S	FLVIGYVXZHLUHP-UHFFFAOYSA-N	16.60	350.6	cr,I to l	4
C4H10N2S	JAEZSIYNWDWMMN-UHFFFAOYSA-N	18.30	357.7	unknown	[5, 6, 7]
C9H7NS2	CYCKHTAVNBPQDB-UHFFFAOYSA-N	16.20	403.8	unknown	[5, 6, 7]
C14H16	CJJFFBINNGWEBO-UHFFFAOYSA-N	22.36	322.0	unknown	[5, 6, 7]
C3H8N2S	GMEHFXXZSWDEDB-UHFFFAOYSA-N	21.34	380.8	unknown	[5, 6, 7]
C2H2N2S2	JLAMDELLBBZOOX-UHFFFAOYSA-N	20.50	414.5	unknown	[5, 6, 7]
C6H6N4O4	HORQAOAYAYGIBM-UHFFFAOYSA-N	18.89	474.1	unknown	[5, 6, 7]
C6H7N3O2	RAUWPNXIALNKQM-UHFFFAOYSA-N	36.28	472.4	unknown	[5, 6, 7]
C15H10O7	REFJWTPEDVJJIY-UHFFFAOYSA-N	43.43	591.7	unknown	[5, 6, 7]
C15H10O5	KZNIFHPLKGYRTM-UHFFFAOYSA-N	49.70	639.7	unknown	[5, 6, 7]
C15H10O6	IQPNAANSBPBGFQ-UHFFFAOYSA-N	42.68	614.6	unknown	[5, 6, 7]
C10H8O3	HSHNITRMYYLLCV-UHFFFAOYSA-N	30.28	461.4	unknown	[5, 6, 7]
C9H6O3	ORHBXUUXSCNDEV-UHFFFAOYSA-N	31.25	503.7	unknown	[5, 6, 7]
C15H10O4	RTIXKCRFFJGDFG-UHFFFAOYSA-N	39.10	558.8	unknown	[5, 6, 7]
C15H10O4	ZQSIJRDFPHDXIC-UHFFFAOYSA-N	35.05	588.8	unknown	[5, 6, 7]
C15H10O3	WMKOZARWBMFKAS-UHFFFAOYSA-N	27.35	487.7	unknown	[5, 6, 7]

Continued on next page

Continued from previous page

**Formula**	**InChIKey**	Δh_i^{fus} **KJ/mol**	T_i^{fus}/K	**Phase**	**Source**
C4H9NO2	BTCSSZJGUNDROE-UHFFFAOYSA-N	35.41	463.8	unknown	[5, 6, 7]
C3H7NO2	FSYKKLYZXJSNPZ-UHFFFAOYSA-N	20.60	481.6	unknown	[5, 6, 7]
C9H6F3N3O	RLQJEEJISHYWON-UHFFFAOYSA-N	31.18	426.4	unknown	[5, 6, 7]
C9H6O3	VXIXUWQIVKSKSA-UHFFFAOYSA-N	23.83	486.0	unknown	[5, 6, 7]
CH4N4O2	IDCPFAYURAQKDZ-UHFFFAOYSA-N	33.46	505.2	unknown	[5, 6, 7]
C9H10ClN5O2	YWTYJOPNNQFBPC-UHFFFAOYSA-N	26.40	416.8	unknown	[5, 6, 7]
C8H11N5O3	MKUXAQIIEYXACX-UHFFFAOYSA-N	30.44	528.2	unknown	[5, 6, 7]
C9H13N5O4	IRSCQMHQWWYFCW-UHFFFAOYSA-N	37.88	509.2	unknown	[5, 6, 7]
C10H15N5O3	JNTOCHDNEULJHD-UHFFFAOYSA-N	35.74	551.2	unknown	[5, 6, 7]
C11H19N3O	BBXXLROWFHWFQY-UHFFFAOYSA-N	20.30	434.4	cr to l	4

Table S5: List of solvents and their identifiers used for the solid–liquid solubility predictions.

Name	Formula	InChIKey	SMILES
Water	H2O	XLYOFNOQVPJJNP-UHFFFAOYSA-N	O
Methanol	CH4O	OKKJLVBELUTLKV-UHFFFAOYSA-N	CO
Ethanol	C2H6O	LFQSCWFLJHTTHZ-UHFFFAOYSA-N	CCO
n-Propanol	C3H8O	BDERNNFJNOPAEC-UHFFFAOYSA-N	CCCO
Isopropanol	C3H8O	KFZMGEQAYNKOFK-UHFFFAOYSA-N	CC(C)O
n-Butanol	C4H10O	LRHPLDYGYMQRHN-UHFFFAOYSA-N	CCCCO
Isobutanol	C4H10O	ZXEKIIBDNHEJCQ-UHFFFAOYSA-N	CC(C)CO
tert-Butanol	C4H10O	DKGAVHZHDRPRBM-UHFFFAOYSA-N	CC(C)(C)O
Isoamyl alcohol	C5H12O	PHTQWCKDNZKARW-UHFFFAOYSA-N	CC(C)CCO
Benzyl alcohol	C7H8O	WVDDGKGOMKODPV-UHFFFAOYSA-N	OCc1ccccc1
Ethylene glycol	C2H6O2	LYCAIKOWRPUZTN-UHFFFAOYSA-N	OCCO
1,3-Propane diol	C3H8O2	YPFDHNVEDLHUCE-UHFFFAOYSA-N	OCCCO
Glycerol	C3H8O3	PEDCQBHIVMGVHV-UHFFFAOYSA-N	OCC(O)CO
Acetone	C3H6O	CSCPPACGZOOCGX-UHFFFAOYSA-N	CC(C)=O
MEK	C4H8O	ZWEHNKRNPOVVGH-UHFFFAOYSA-N	CCC(C)=O
MIBK	C6H12O	NTIZESTWPVYFNL-UHFFFAOYSA-N	CC(=O)CC(C)C
Cyclohexanone	C6H10O	JHIVVAPYMSGYDF-UHFFFAOYSA-N	O=C1CCCCC1
Methyl acetate	C3H6O2	KXKVLQRXCPHEJC-UHFFFAOYSA-N	COC(C)=O
Ethyl acetate	C4H8O2	XEKOWRVHYACXOJ-UHFFFAOYSA-N	CCOC(C)=O
n-Propyl acetate	C5H10O2	YKYONYBAUNKHLG-UHFFFAOYSA-N	CCCOC(C)=O
Isopropyl acetate	C5H10O2	JMMWKPVZQRWMSS-UHFFFAOYSA-N	CC(=O)OC(C)C
n-Butyl acetate	C6H12O2	DKPFZGUDAPQIHT-UHFFFAOYSA-N	CCCCOC(C)=O
Isobutyl acetate	C6H12O2	GJRQTCIYDGXPES-UHFFFAOYSA-N	CC(=O)OCC(C)C
Isoamyl acetate	C7H14O2	MLFHJEHSLIIPHL-UHFFFAOYSA-N	CC(=O)OCCC(C)C
gamma-Valerolactone	C5H8O2	GAEKPEKOJKCEMS-UHFFFAOYSA-N	CC1CCC(=O)O1
Glycol diacetate	C6H10O4	JTXMVXSTHSMVQF-UHFFFAOYSA-N	CC(=O)OCCOC(C)=O
Diethyl succinate	C8H14O4	DKMROQRQHGEIOW-UHFFFAOYSA-N	CCOC(=O)CCC(=O)OCC
Diethyl ether	C4H10O	RTZKZFJDLAIYFH-UHFFFAOYSA-N	CCOCC
Diisopropyl ether	C6H14O	ZAFNJMIOTHYJRJ-UHFFFAOYSA-N	CC(C)OC(C)C
MTBE	C5H12O	BZLVMXJERCGZMT-UHFFFAOYSA-N	COC(C)(C)C
ETBE	C6H14O	NUMQCACRALPSHD-UHFFFAOYSA-N	CCOC(C)(C)C
THF	C4H8O	WYURNTSHIVDZCO-UHFFFAOYSA-N	C1CCOC1
1,4-Dioxane	C4H8O2	RYHBNJHYFVUHQT-UHFFFAOYSA-N	C1COCCO1
Anisole	C7H8O	RDOXTESZEPMUJZ-UHFFFAOYSA-N	COc1ccccc1
Pentane	C5H12	OFBQJSOFQDEBGM-UHFFFAOYSA-N	CCCCC
Hexane	C6H14	VLKZOEOYAKHREP-UHFFFAOYSA-N	CCCCCC
Heptane	C7H16	IMNFDUFMRHMDMM-UHFFFAOYSA-N	CCCCCCC
Cyclohexane	C6H12	XDTMQSROBMDMFD-UHFFFAOYSA-N	C1CCCCC1
Methylcyclohexane	C7H14	UAEPNZWRGJTJPN-UHFFFAOYSA-N	CC1CCCCC1
Benzene	C6H6	UHOVQNZJYSORNB-UHFFFAOYSA-N	c1ccccc1
Toluene	C7H8	YXFVVABEGXRONW-UHFFFAOYSA-N	Cc1ccccc1
p-Cymene	C10H14	HFPZCAJZSCWRBC-UHFFFAOYSA-N	Cc1ccc(C(C)C)cc1
Dichloromethane	CH2Cl2	YMWUJEATGCHHMB-UHFFFAOYSA-N	ClCCl
Chloroform	CHCl3	HEDRZPFGACZZDS-UHFFFAOYSA-N	ClC(Cl)Cl
Carbon tetrachloride	CCl4	VZGDMQKNWNREIO-UHFFFAOYSA-N	ClC(Cl)(Cl)Cl
1,2-Dichloroethane	C2H4Cl2	WSLDOOZREJYCGB-UHFFFAOYSA-N	ClCCCl
Chlorobenzene	C6H5Cl	MVPPADPHJFYWMZ-UHFFFAOYSA-N	Clc1ccccc1
Acetonitrile	C2H3N	WEVYAHXRMPXWCK-UHFFFAOYSA-N	CC#N
DMF	C3H7NO	ZMXDDKWLCZADIW-UHFFFAOYSA-N	CN(C)C=O
DMAc	C4H9NO	FXHOOIRPVKKKFG-UHFFFAOYSA-N	CC(=O)N(C)C
NMP	C5H9NO	SECXISVLQFMRJM-UHFFFAOYSA-N	CN1CCCC1=O
DMPU	C6H12N2O	GUVUOGQBMYCBQP-UHFFFAOYSA-N	CN1CCCN(C)C1=O
DMSO	C2H6OS	IAZDPXIOMUYVGZ-UHFFFAOYSA-N	CS(C)=O
Sulfolane	C4H8O2S	HXJUTPCZVOIRIF-UHFFFAOYSA-N	O=S1(=O)CCCC1
Dimethyl carbonate	C3H6O3	IEJIGPNLZYLLBP-UHFFFAOYSA-N	COC(=O)OC
Ethylene carbonate	C3H4O3	KMTRUDSVKNLOMY-UHFFFAOYSA-N	O=C1OCCO1
Propylene carbonate	C4H6O3	RUOJZAUFBMNUDX-UHFFFAOYSA-N	CC1COC(=O)O1

Continued on next page

Continued from previous page

**Name**	**Formula**	**InChIKey**	**SMILES**
HMPA	C6H18N3OP	GNOIPBMMFNIUFM-UHFFFAOYSA-N	CN(C)P(=O)(N(C)C)N(C)C
Nitromethane	CH3NO2	LYGJENNIWJXYER-UHFFFAOYSA-N	C[N+](=O)[O-]
Formic acid	CH2O2	BDAGIHXWWSANSR-UHFFFAOYSA-N	O=CO
Acetic acid	C2H4O2	QTBSBXVTEAMEQO-UHFFFAOYSA-N	CC(=O)O
Acetic anhydride	C4H6O3	WFDIJRYMOXRFFG-UHFFFAOYSA-N	CC(=O)OC(C)=O
Pyridine	C5H5N	JUJWROOIHBZHMG-UHFFFAOYSA-N	c1ccncc1
Triethylamine	C6H15N	ZMANZCXQSJIPKH-UHFFFAOYSA-N	CCN(CC)CC
Methoxyethanol	C3H8O2	XNWFRZJHXBZDAG-UHFFFAOYSA-N	COCCO
Lactic acid	C3H6O3	JVTAAEKCZFNVCJ-UHFFFAOYSA-N	CC(O)C(=O)O
Tetrahydrofurfuryl alcohol	C5H10O2	BSYVTEYKTMYBMK-UHFFFAOYSA-N	OCC1CCCO1
Carbon disulfide	CS2	QGJOPFRUJISHPQ-UHFFFAOYSA-N	S=C=S

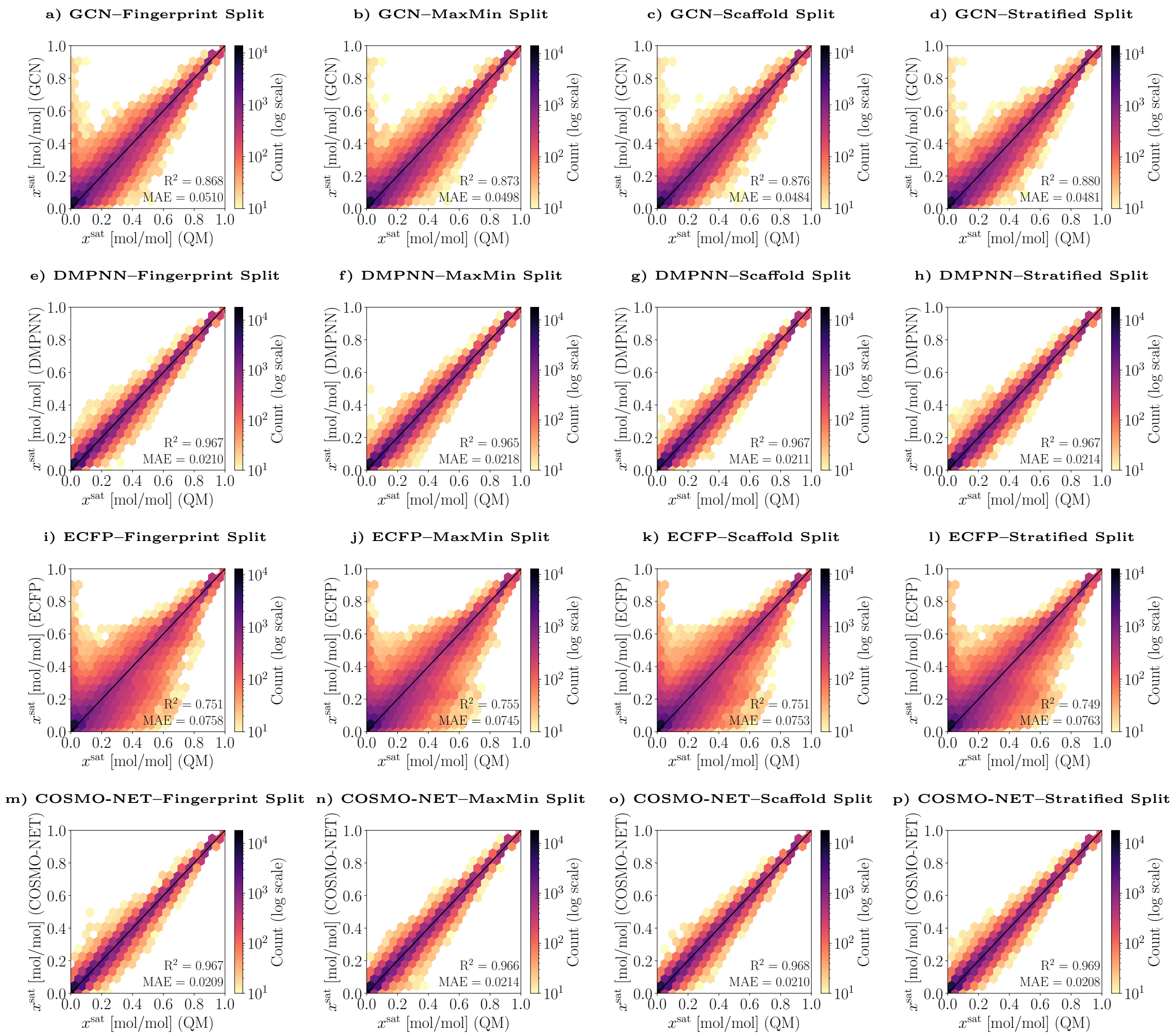

Figure S36: Parity plots for solid–liquid solubility (x^{sat} [mol/mol]) predictions using COSMO-SAC 2002 with COSMO descriptors ($p(\sigma)$, A, V) from four models: GCN (a–d), DMPNN (e–h), ECFP-MLP (i–l), and COSMO-NET (m–p). Results are shown across four train-test splitting strategies (Fingerprint, MaxMin, Scaffold, and Stratified). Predictions are compared against reference values calculated from QM COSMO descriptors. Hexagonal bin color indicates the number of data points.

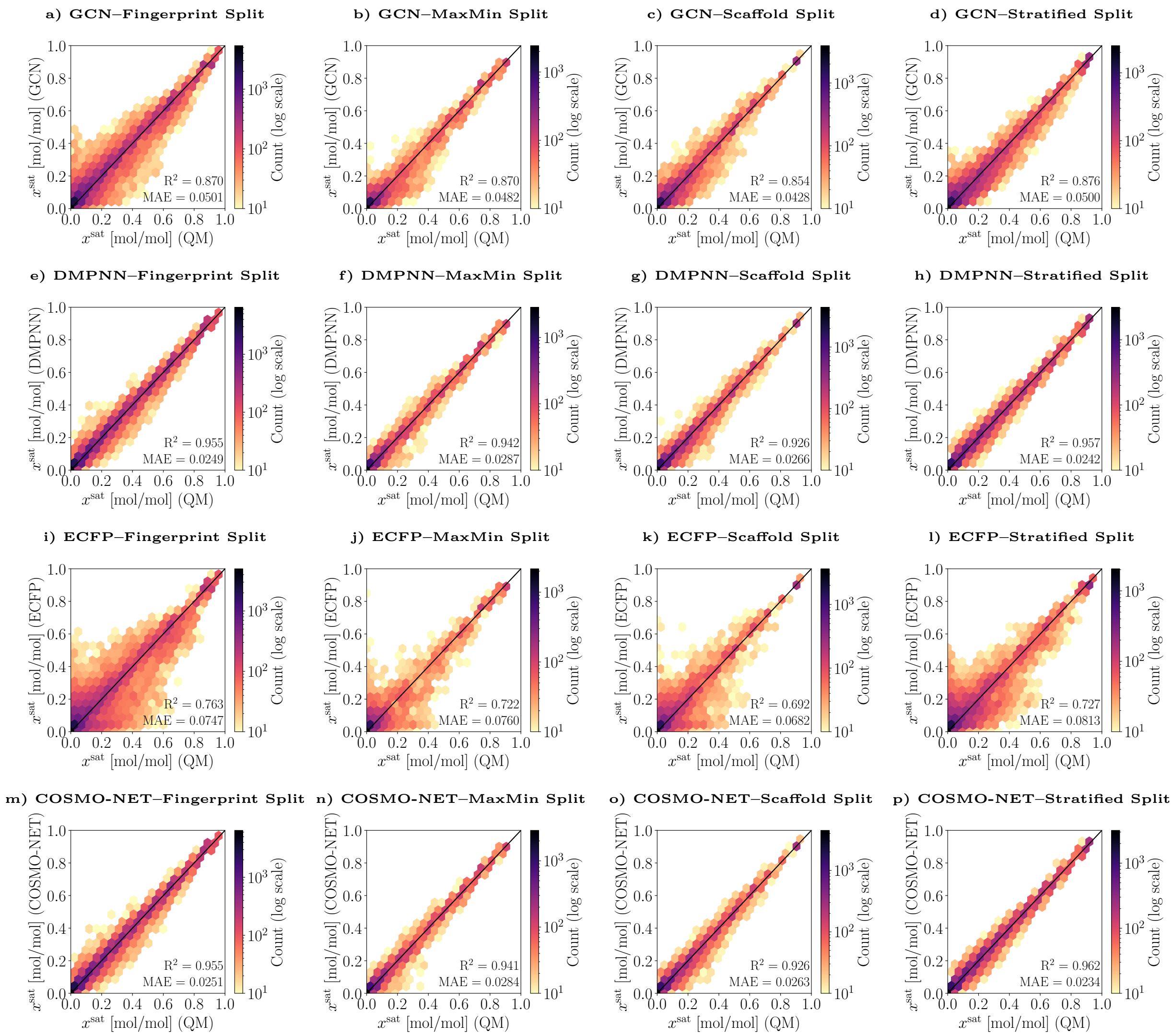

Figure S37: Parity plots for solid–liquid solubility (x^{sat} [mol/mol]) predictions using COSMO-SAC 2002 with COSMO descriptors ($p(\sigma)$, A, V) from four models: GCN (a–d), DMPNN (e–h), ECFP-MLP (i–l), and COSMO-NET (m–p). Results are shown across four train-test splitting strategies (Fingerprint, MaxMin, Scaffold, and Stratified). Predictions are compared against reference values calculated from QM COSMO descriptors **using only test set data**. Hexagonal bin color indicates the number of data points.

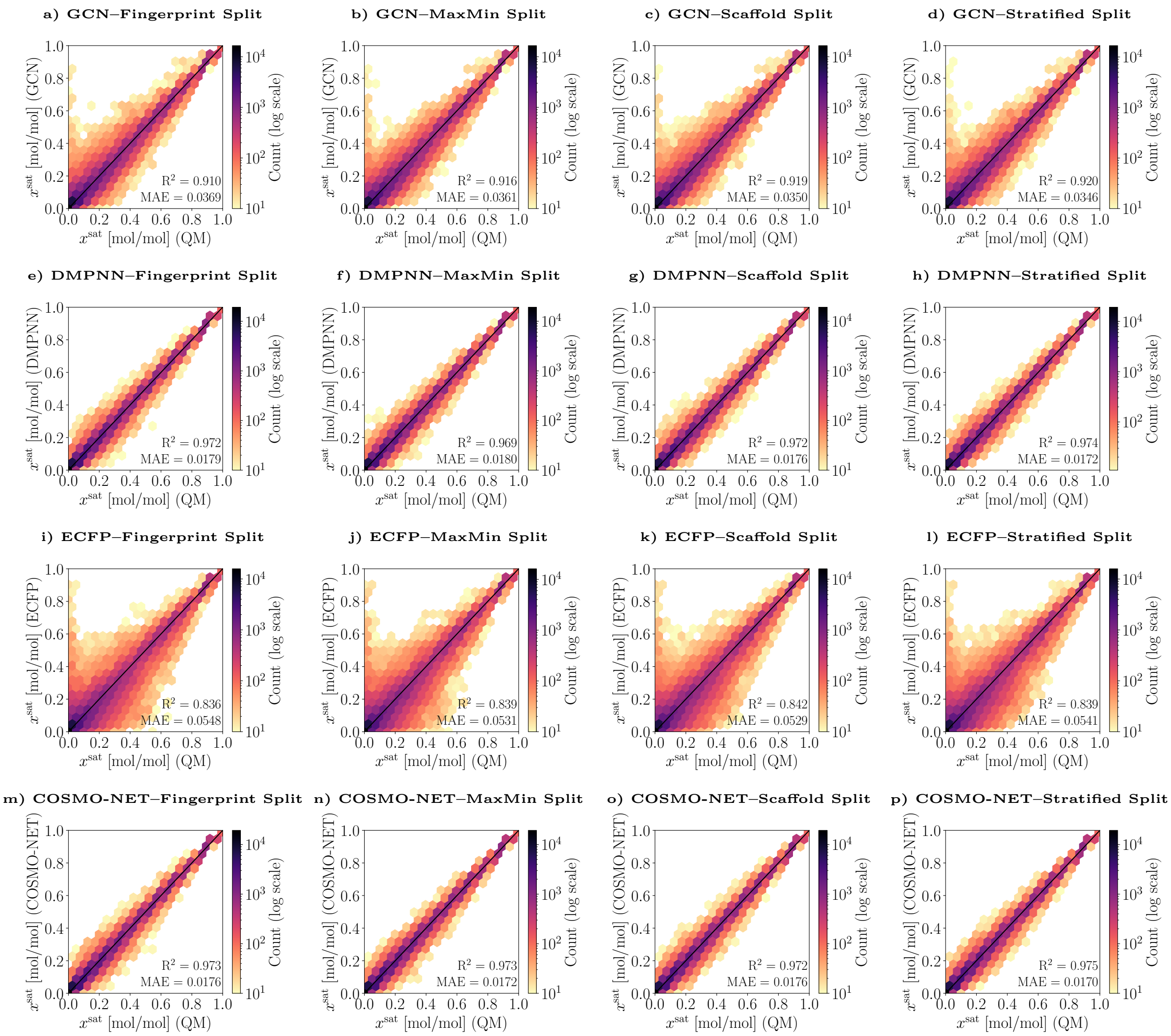

Figure S38: Parity plots for solid–liquid solubility (x^{sat} [mol/mol]) predictions using COSMO-SAC 2010 with COSMO descriptors ($p(\sigma)$, A, V) from four models: GCN (a–d), DMPNN (e–h), ECFP-MLP (i–l), and COSMO-NET (m–p). Results are shown across four train-test splitting strategies (Fingerprint, MaxMin, Scaffold, and Stratified). Predictions are compared against reference values calculated from QM COSMO descriptors. Hexagonal bin color indicates the number of data points.

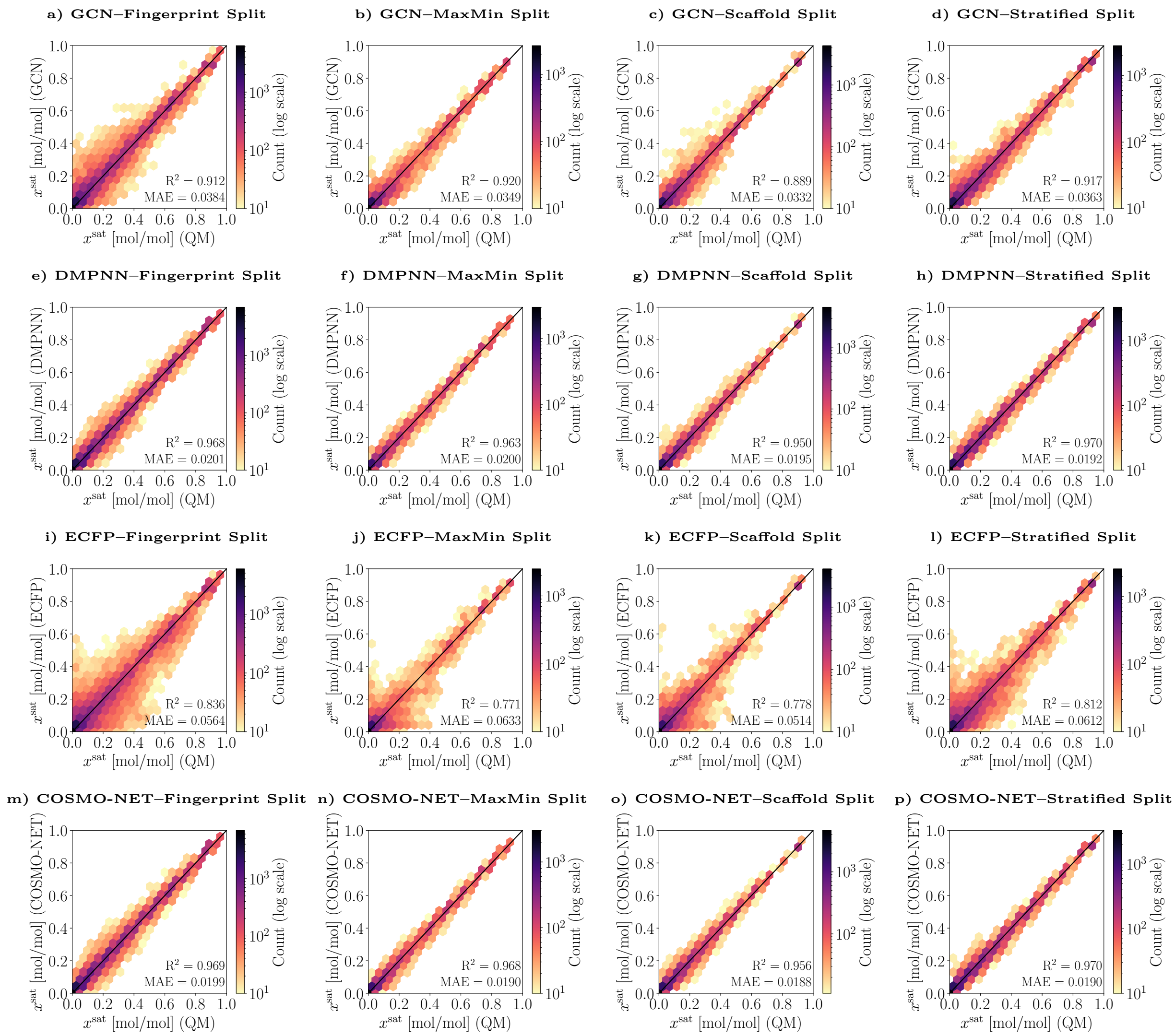

Figure S39: Parity plots for solid–liquid solubility (x^{sat} [mol/mol]) predictions using COSMO-SAC 2010 with COSMO descriptors ($p(\sigma)$, A, V) from four models: GCN (a–d), DMPNN (e–h), ECFP-MLP (i–l), and COSMO-NET (m–p). Results are shown across four train-test splitting strategies (Fingerprint, MaxMin, Scaffold, and Stratified). Predictions are compared against reference values calculated from QM COSMO descriptors **using only test set data**. Hexagonal bin color indicates the number of data points.

References

[1] Shiang-Tai Lin and Stanley I. Sandler. A priori phase equilibrium prediction from a segment contribution solvation model. *Industrial & Engineering Chemistry Research*, 41(5):899–913, 2002.

[2] Chieh-Ming Hsieh, Stanley I. Sandler, and Shiang-Tai Lin. Improvements of cosmo-sac for vapor–liquid and liquid–liquid equilibrium predictions. *Fluid Phase Equilibria*, 297(1):90–97, 2010.

[3] Wei-Lin Chen, Chieh-Ming Hsieh, Li Yang, Chan-Chia Hsu, and Shiang-Tai Lin. A critical evaluation on the performance of cosmo-sac models for vapor–liquid and liquid–liquid equilibrium predictions based on different quantum chemical calculations. *Industrial & Engineering Chemistry Research*, 55(34):9312–9322, 2016.

[4] Z.-Y. Zhang, M. Frenkel, K. N. Marsh, and R. C. Wilhoit. Datasheet from landolt-börnstein - group iv physical chemistry · volume 8a: "enthalpies of fusion and transition of organic compounds" in springermaterials. Copyright 1995 Springer-Verlag Berlin Heidelberg.

[5] William Acree and James S Chickos. Phase transition enthalpy measurements of organic and organometallic compounds. sublimation, vaporization and fusion enthalpies from 1880 to 2015. part 1. c1- c10. *Journal of Physical and Chemical Reference Data*, 45(3), 2016.

[6] William Acree and James S Chickos. Phase transition enthalpy measurements of organic and organometallic compounds and ionic liquids. sublimation, vaporization, and fusion enthalpies from 1880 to 2015. part 2. c11–c192. *Journal of Physical and Chemical Reference Data*, 46(1), 2017.

[7] William Acree and James S Chickos. Phase transition enthalpy measurements of organic compounds. an update of sublimation, vaporization, and fusion enthalpies from 2016 to 2021. *Journal of Physical and Chemical Reference Data*, 51(4), 2022.

[8] Michael Frenkel, Robert D. Chirico, Vladimir Diky, Qian Dong, Kenneth N. Marsh, John H. Dymond, William A. Wakeham, Stephen E. Stein, Erich Königsberger, and Anthony R. H. Goodwin. Xml-based iupac standard for experimental, predicted, and critically evaluated thermodynamic property data storage and capture (thermoml) (iupac recommendations 2006). *Pure and Applied Chemistry*, 78(3):541–612, 2006.
